# 7′,5′-alpha-bicyclo-DNA: new chemistry for oligonucleotide exon splicing modulation therapy

**DOI:** 10.1093/nar/gkab1097

**Published:** 2021-11-26

**Authors:** Damien Evéquoz, Ingrid E C Verhaart, Davy van de Vijver, Wolfgang Renner, Annemieke Aartsma-Rus, Christian J Leumann

**Affiliations:** Alpha Anomeric, 140 Bis, Rue de Rennes, 75006 Paris, France; Department of Human Genetics, Leiden University Medical Center, 2333 ZA Leiden, The Netherlands; Department of Human Genetics, Leiden University Medical Center, 2333 ZA Leiden, The Netherlands; Alpha Anomeric, 140 Bis, Rue de Rennes, 75006 Paris, France; Department of Human Genetics, Leiden University Medical Center, 2333 ZA Leiden, The Netherlands; Department of Chemistry and Biochemistry, University of Bern, Freiestrasse 3, CH-3012 Bern, Switzerland

## Abstract

Antisense oligonucleotides are small pieces of modified DNA or RNA, which offer therapeutic potential for many diseases. We report on the synthesis of 7′,5′-α-bc-DNA phosphoramidite building blocks, bearing the A, G, T and ^Me^C nucleobases. Solid-phase synthesis was performed to construct five oligodeoxyribonucleotides containing modified thymidine residues, as well as five fully modified oligonucleotides. Incorporations of the modification inside natural duplexes resulted in strong destabilizing effects. However, fully modified strands formed very stable duplexes with parallel RNA complements. In its own series, 7′,5′-α-bc-DNA formed duplexes with a surprising high thermal stability. CD spectroscopy and extensive molecular modeling indicated the adoption by the homo-duplex of a ladder-like structure, while hetero-duplexes with DNA or RNA still form helical structure. The biological properties of this new modification were investigated in animal models for Duchenne muscular dystrophy and spinal muscular atrophy, where exon splicing modulation can restore production of functional proteins. It was found that the 7′,5′-α-bc-DNA scaffold confers a high biostability and a good exon splicing modulation activity *in vitro* and *in vivo*.

## Graphical Abstract

**Graphical Abstract:**
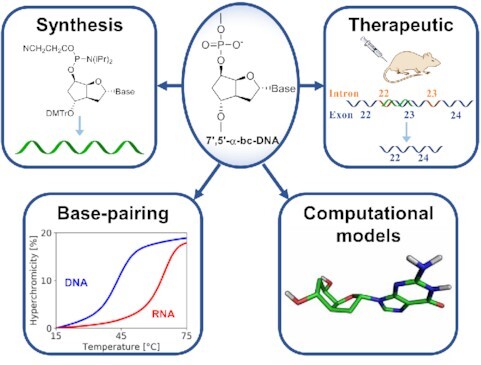
General scheme illustrating the synthesis, base-pairing characterization, modeling and potential therapeutic application of 7?,5?-?-bc-oligonucleotides.

## INTRODUCTION

Antisense oligonucleotide-based therapeutics efficiently interfere with the expression of genetic information by binding to target RNAs via Watson–Crick base-pairing, inhibiting gene expression via distinct biochemical mechanisms such as steric block, splice switching, RNase H mediated decay or the siRNA and miRNA pathways. At the beginning of 2020, 13 antisense oligonucleotide drugs have been approved to market (1). However, clinical applications are still limited to local delivery to brain or systemic delivery to liver, due to their poor pharmacokinetic properties and their dose-limiting toxicity, often triggered by the use of phosphorothioate (PS) internucleosidic linkages (2).

**Figure 1.**
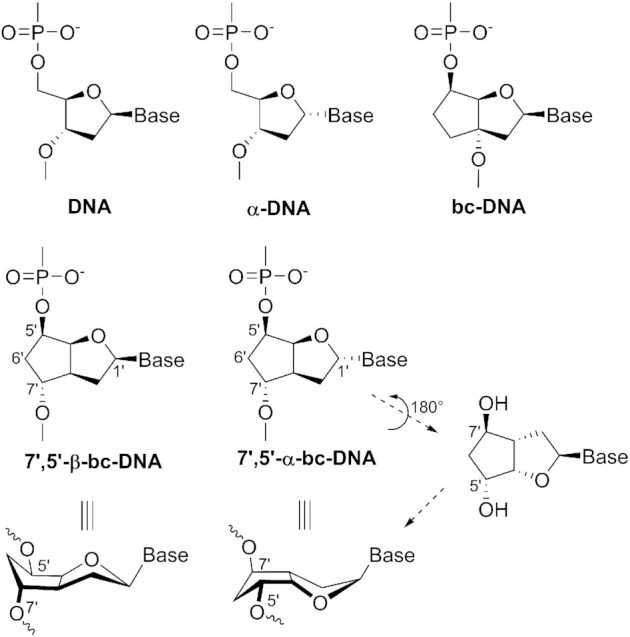
Schematic comparison of 7′,5′-α-bc-DNA with DNA analogs. Chemical structures of DNA, α-DNA and bc-DNA (top), the diastereomeric 7′,5′-β-bc-DNA (center left) and 7′,5′-α-bc-DNA (center right) as well as the 3D structural relationship arising from a 180° rotation around the pseudo C2-axis running through the centers C1′ and C6′ (bottom).

Chemical modification of oligonucleotides is therefore required for improved therapeutic activity. One of the first sugar modified oligonucleotide analogues to be investigated as antisense agents consisted of 2′deoxy-α-ribofuranosyl nucleosides (α-DNA, Figure 1). This modification rapidly attracted interest due to its substantially increased resistance to both, endo- and exo-nucleases (3), and therefore prompted further investigation on its pairing properties. In its own series, α-DNA forms anti-parallel B-like duplexes, via Watson–Crick base pairing (4). Furthermore, α-DNA is able to form duplexes with natural DNA also via Watson–Crick base pairing, resulting in B-like helices with an unusual parallel strand orientation (5,6), as anticipated from very early work based on Dreiding models (7). The thermodynamic duplex stability was found to be similar to natural DNA (8). More interestingly, α-DNA appears to bind more strongly to RNA than its natural counterpart, but the resulting duplexes are not substrate to RNase H, an enzyme that is responsible for the cleavage of RNA in hybrid DNA/RNA duplexes (9–11). The RNase H activity could be restored by using mixed α/β-oligodeoxynucleotides, in which the α-nucleotides are inserted with a polarity reversal resulting in 3′-3′ and 5′-5′ internucleotide linkages (12,13). Modern biological applications often rely on the use of conformationally restrained bicyclic sugar, such as LNA or its analogs, to favor RNA binding and thus, improves the overall efficacy (14).

In our laboratory, we undertook a structure-activity relationship study of alteration of the DNA backbone geometry. In this context, we were using the scaffold of bc-DNA (Figure 1), a conformationally constrained mimic of DNA (15), to attach the internucleotide linkage at different position on the carbocyclic ring. This research led us to the synthesis and characterization of 7′,5′-β-bc-DNA (Figure 1), a bc-DNA analog in which the internucleosidic hydroxyl group has been shifted from the 3′ to the 7′ position (16). This shift resulted in a nucleoside structure with a pseudo C_2_-axis running through the centers C6′ and C1′. In consequence, its α-anomer (7′,5′-α-bc-DNA, Figure 1) could be seen as a close mimic of its β counterpart. A simplistic model showed that the α-anomers with 7′, 5′ polarity reversal will orient the internucleosidic oxy-groups and the nucleobase in a similar fashion as the β-anomers. Previous work showed that single incorporation of 7′,5′-β-bc-DNA methylcytosine inside DNA duplexes has a substantial stabilizing effect, indicating that this backbone alteration could be well accommodated inside natural helices. However, due to an atypical sugar conformation pattern, fully modified 7′,5′-β-bc-DNA binds poorly with natural nucleic acids (16). Therefore, we became interested in testing whether oligonucleotides with 7′,5′-α-bc-DNA units in polarity reversal could also be well tolerated in duplexes and whether this could be extended also to fully modified 7′,5′-α-bc-oligonucleotides. In this article we report on the synthesis of the adenine, guanine, thymine and 5-methylcytosine phosphoramidite building blocks, the incorporation with reverse polarity of thymine nucleosides into DNA strands and the synthesis, characterization and biological activity of fully modified oligonucleotides. Molecular mechanics was performed to gain insight on the structures formed by this system.

## MATERIALS AND METHODS

### Temperature of melting

UV melting experiments were recorded on a Varian Cary Bio 100 UV/vis spectrophotometer. Experiments were performed at 2 μM duplex concentration, 10 mM NaH_2_PO_4_, between 0 M and 150 mM NaCl and the pH value was adjusted to 7.0. The samples were protected from evaporation by a covering layer of dimethylpolysiloxane. The absorbance was monitored at λ = 260 nm. For every experiment, three cooling-heating cycles were performed with a temperature gradient of 0.5°C min^−1^. The maxima of the curves first derivative were extracted with the Varian WinUV software and *T*_m_ values were reported as the average of the six ramps.

### Circular dichroism spectroscopy

Circular dichroism (CD) spectra were recorded on a Jasco J-715 spectropolarimeter equipped with a Jasco PFO-350S temperature controller. Sample conditions were the same as for UV melting experiments. Spectra were recorded between λ = 210 and 320 nm at a 50 nm min^−1^ rate and the temperature was measured directly from the sample. For each experiment, a blank containing the same salt concentration as the sample was recorded. The reported spectra were obtained by taking a smoothed average of three scans and subtracting the corresponding blank spectrum.

### Oligonucleotide synthesis, deprotection and purification

Oligonucleotides syntheses were performed on a Pharmacia-Gene-Assembler-Plus DNA synthesizer on a 1.3 μmol scale. The natural DNA phosphoramidites (dT, dC4bz, dG2DMF, dA6Bz) and the solid support (dA-Q-CPG 500, dmf-dG-Q-CPG 500, Glen Unysupport 500) were purchased from Glen Research. The 5′-Palmitate-C6-CE phosphoramidite and the 5′-Fmoc-Amino C6 Modifier phosphoramidite were purchased from LGC LINK. The natural DNA phosphoramidites were prepared as a 0.1 M solution in MeCN and were coupled using a 1.5 min step. The 7′,5′-α-bc-DNA phosphoramidites were prepared as a 0.1 M solution in 1,2-dichloroethane and were coupled using an extended 12 min step. 5-(Ethylthio)-1H-tetrazole (0.25 M in MeCN) was used as coupling agent. Detritylation of the modified nucleosides was performed with a solution of 5% dichloroacetic acid in 1,2-dichloroethane. For oligonucleotides containing PS-linkages (**ON9** and **12**, Table 2), sulfurization was performed with a solution of 0.2 M phenylacetyl disulfide in MeCN/pyridine (1:1) and with a reaction time of 3.5 min. For oligonucleotides containing PO-linkages (**ON1-8,10,11,13**), oxidation was performed with a solution of 0.02 M iodine in water/collidine/MeCN. Capping was performed with standard conditions. Cleavage from the solid support and deprotection of the oligonucleotides was achieved by treatment with concentrated ammonia at 55°C for 16 h. After centrifugation, the supernatants were collected, the beads were further washed with H_2_O (2 × 0.5 ml) and the resulting solutions were evaporated to dryness. The crude oligonucleotides were purified by ion-exchange HPLC (Dionex-DNAPac PA200). Buffer solutions of 25 mM Trizma in H_2_O, pH 8.0, were used as the mobile phase ‘A’ and 25 mM Trizma, 1.25 M NaCl in H_2_O, pH 8.0, was used as the mobile phase ‘B’. For the phosphorothioate strand, a buffer solution of 10 mM NaOH in H_2_O, pH 12.0, was used as the mobile phase ‘A’ and 10 mM NaOH, 2.50 M NaCl in H_2_O, pH 12.0, was used as the mobile phase ‘B’. The purified oligonucleotides were then desalted with Sep-pack C-18 cartridges. The GalNac conjugate (**ON13**, Table 2) was obtained by preparing a 7′-hexylamino oligonucleotide, followed by post synthesis conjugation of the triantennary GalNAc cluster and subsequent HPLC purification following described protocols (17). The MOE conjugated to a GalNac (**MOE1**, Table 2) was directly purchased from Axolabs GmbH. Concentrations were determined by measuring the absorbance at λ = 260 nm with a Nanodrop spectrophotometer by using the extinction coefficient of the corresponding natural DNA oligonucleotides. Characterizations of the oligonucleotides were performed by ESI- mass spectrometry or by LC-MS (Supplementary Table S1).

### Molecular modeling

Conformational analyses of the monomers were carried out with the Gaussian 09 software package (18). Stepwise potential energy surface scans were performed by varying the ν1 and ν3 angles. These calculations were performed with the Hartree-Fock methodology and a 6–31G* basis set. The atomic charges of the four nucleobase units were then calculated using the R.E.D. III.5 tools package (19). The duplex structures have been constructed with Discovery Studio 4.1 (20), starting from a natural DNA duplex in A conformation. Molecular dynamics simulations were carried out with the GROMACS 5.0.6 simulation package (21). The Amber94 force field was manually implemented with parameters for 7′,5′-α-bc-DNA and was used for the simulations. The duplexes were solvated in a rhombic dodecahedron with at least 1.0 nm to the border. The systems were filled with water (TIP3P) and neutralization was performed by addition of Na + counterions. Cutoffs of 1.4 nm were applied for short-range electrostatic and van der Waals forces. Long-range electrostatic interactions were calculated by using the particle mesh Ewald method (22). The neutral structures were then relaxed by energy minimization (steepest descent minimization). The simulations were run with a time step of 2 fs computed by leap-frog integrator, in periodic boundary conditions and by using the LINCS algorithm (23). Water molecules were equilibrated by a 100 ps run at 300 K in NVT conditions, followed by a 100 ps run at 300 K in NPT conditions. Atomic positions of the duplexes were restrained during these water equilibration steps. Simulations were then carried out under NPT conditions at 300 K and without restrains. The systems were equilibrated during 10 ns and then data were collected over 50 ns runs. MD trajectories were analyzed with the GROMACS package and with 3dna package (24).

### Biostability in serum

**ON6** and its corresponding natural oligonucleotide were diluted to 10 μM in a 1:1 mixture of H_2_O and human serum [human male AB plasma, USA origin, sterile-filtered (Sigma)]. The reactions were performed at a 20 μl scale and were incubated at 37°C. Control reactions (Supplementary Figure S1a,f) were performed by incubating the oligonucleotides at 10 μM in H_2_O at 37°C for 24 h. The reactions were stopped at specific times by addition of formamide (20 μl). The resulting mixtures were stored at −20°C before being heat denaturated for 5 min at 90°C and then analyzed by 20% denaturing PAGE. Visualization was performed with a stains-all solution (Supplementary Figure S1).

### Biostability in acidic conditions

**ON10** was diluted to 20 μM in an acetate buffer solution (0.1 M AcOH in H_2_O, pH adjusted to 4.5). The reactions were performed at a 100 μl scale and were incubated at 37°C. The reactions were stopped at 0, 2 and 24 h, by filtrating the solution with a Spin column (Amicon® Ultra 0.5 ml, Sigma-Aldrich) followed by buffer exchange with a Trizma buffer solution (2 × 200 μl; 25 mM Trizma in H_2_O, pH 8.0). The reaction outcome was analyzed by LC-MS (Supplementary Figure S2).

### General cell culture and skipping analyses

Experiments were conducted in mouse control immortalized myoblast cultures (C2C12). The cells were propagated and differentiated into myotubes using standard culturing techniques as described previously. The cells were treated with the splice-switching oligonucleotides (SSOs) by using a transfection reagent or by naked delivery (gymnosis). Each experiment was performed at least in duplicate.

After SSO treatment, total RNA was extracted, and molecular analysis was conducted. Reverse transcriptase amplification (RT-PCR), using a two-step (nested) PCR reaction, was undertaken to study the targeted regions of the dystrophin pre-mRNA or induced exonic rearrangements.

For analyzing the SSOs aiming to induce skipping of exon 23 and of exon 22 + 23, the RT-PCR was conducted on the region spanning exon 22 and 23. After cDNA synthesis, first round PCR was performed using specific primers in mouse exons 20 and 26 (region 20–26) and the second round PCR was performed using specific primers in mouse exons 21 and 24 (region 21–24). The reactions were analyzed on an agarose gel, including a size standard. Skipping efficacy was quantified with an image processing program (ImageJ).

### *In vitro* transfection experiments

The cells were transfected with 3 μg SSOs by using Lipofectamine 2000 as a transfection reagent. Cell were harvested after 24 h treatment and mRNA skipping efficacy was analyzed as indicated previously (25).

### *In vitro* gymnotic experiments

The cells were treated with SSOs at concentration ranging from 5 to 40 μM without any delivery reagent. Cell were harvested after 74 h treatment and mRNA skipping efficacy was analyzed as indicated previously (25). Gymnotic experiments were performed for SSOs at concentrations increasing from 5 to 40 μM.

### *In vivo* intra-muscular injections

The animal experiments were carried out at the animal facility of the Leiden University Medical Center following the guidelines of and were approved by the Animal Ethics Committee (DEC) of the Leiden University Medical Center. Nine- to thirteen-week-old male *mdx* mice were anesthetized and then injected intra-muscularly in the gastrocnemius right (GR) and left (GL) and the triceps right (TR) and left (TL) with **ON9-12** or positive controls. Each SSO was injected two times over two consecutive days, at an injection dose of 50 μg dissolved in 40 μl saline solution. Ten days after the last injection mice were killed by cervical dislocation and muscles tissues were isolated and stored at −80°C.

Total RNA was extracted from tested muscle tissues with Trizol reagent as per manufacturer’s protocol. cDNA synthesis was performed with 400 ng RNA and then analysis was performed by RT-PCR following the protocol described previously. Precise quantification of the skipped product was performed with a Lab-on-a-chip. For **ON9**, an outlier sample which rendered no skipped product was excluded.

### *In vivo* subcutaneous injections

The animal experiments were carried out at the animal facility of Charles River Discovery Services Finland following the European and international legislation and guidelines and were approved by the Animal Welfare Team. Seven-week-old SMA mice (FVB.Cg-Smn1tm1Hung Tg (SMN2)2Hung/J mouse model of Spinal Muscular Atrophy (SMA) Type III, Jackson Laboratory, 005058) were subcutaneously injected with a solution of **ON13** or **MOE1** in PBS, at doses of 10, 33 and 100 mg/kg. A negative control was performed by subcutaneously injecting a PBS solution. Each cohort is composed of three animals. About 72 h after treatment, mice were killed by pentobarbital (180 mg/kg) injection. About 20 mg from the liver left lobe was taken, immerged in RNAlater and frozen with liquid nitrogen. Total RNA was extracted with RNeasy-Plus Mini kit. About 500 ng mRNA was reverse transcribed using Superscript IV first-strand RT kit with random hexamers. The ddPCR was performed on a QX200TM Droplet Digital PCR system (Bio-Rad), with a 900 nM:250 nM primer:probe ratio. For SMN-fl quantification, an exon 7/8 primer (5′-GCTCACATTCCTTAAATTAAGGAGAAA-3′), an exon 8 primer (5′-TCCAGATCTGTCTGATCGTTTCTT-3′) and an exon 8 probe (5′-6FAM-CTGGCATAGAGCAGCACTAAATGACACCAC-TAMRA-3′) were used. For SMN-Δ7 quantification, an exon 6/8 primer (5′-TGGCTATCATACTGGCTATTATATGGAA-3′), an exon 8 primer (5′- TCCAGATCTGTCTGATCGTTTCTT-3′) and an exon 8 probe (5′-6FAM-CTGGCATAGAGCAGCACTAAATGACACCAC-TAMRA-3′) were used. The relative transcript levels were normalized by taking the negative control cohort as reference.

## RESULTS

### Synthesis of phosphoramidite building blocks

**Figure 2.**
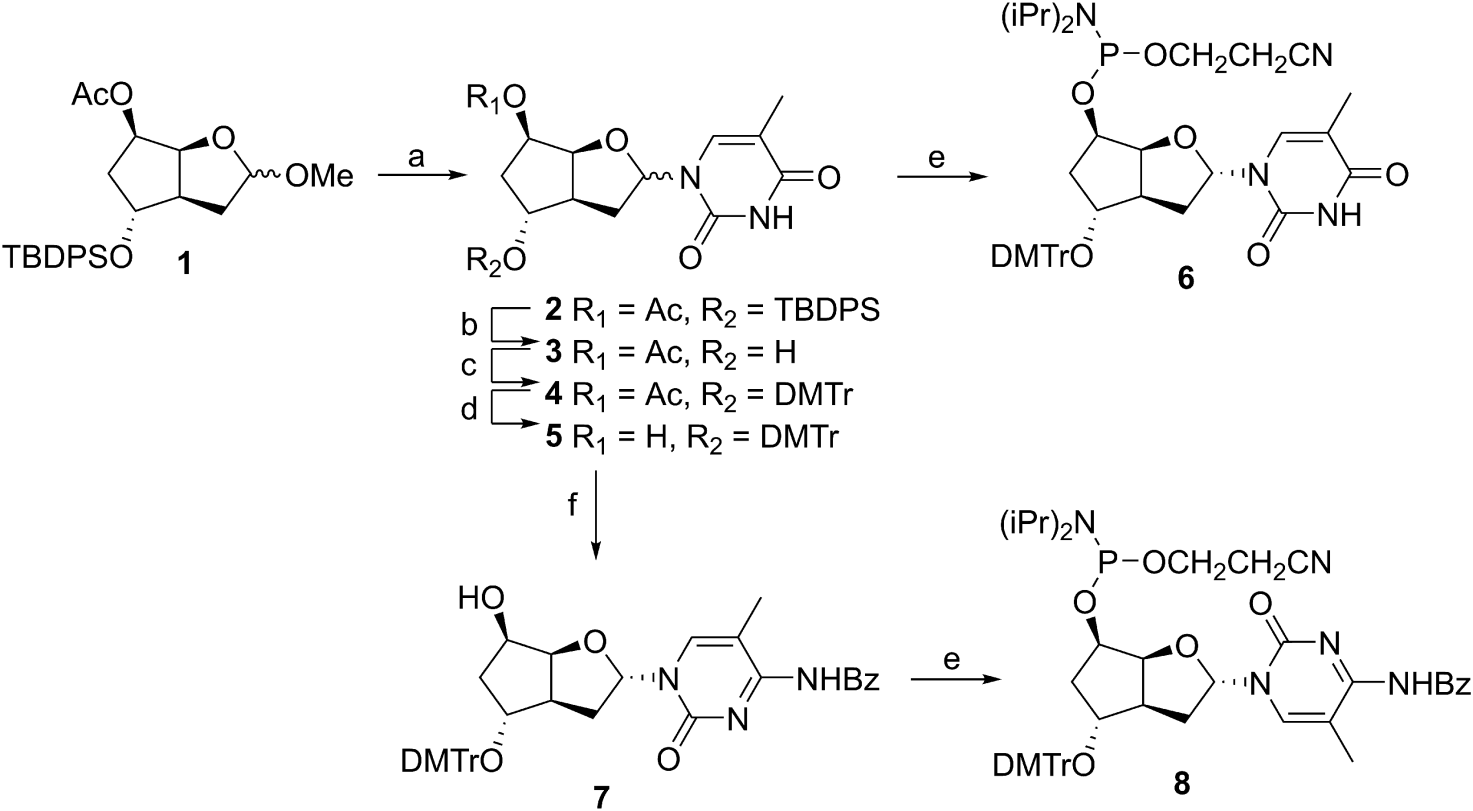
Synthesis of pyrimidine building blocks. (**a**) Thymine, BSA, TMSOTf, MeCN, rt, 18 h, 82%; (**b**) TBAF, THF, 2 h, 75%; (**c**) DMTr-Cl, pyridine, rt, 24 h, 96%; (**d**) K_2_CO_3_, MeOH, 3 h, 86%; (**e**) 2-cyanoethyl N,N,N′,N′-tetraisopropylphosphordiamidite, ETT, DCM, rt, 1 h, 81% for **6**, 30 min, 80% for **8**; (**f**) (i) BSA, 1,2,4-triazole, POCl_3_, Et_3_N, MeCN, rt, 7 h, (ii) 1,4-dioxane/NH_4_OH, rt, 3 h, (iii) Bz_2_O, Et_3_N, DMF, rt, 18 h, 83%.

The protected sugar **1**, which we developed in a previous study (16), represents a suitable starting point for the construction of the four phosphoramidite building blocks. We reasoned that the concave conformation of the bicyclic scaffold should favor formation of the α-anomer under Vorbrüggen conditions. Indeed, treatment of a mixture of sugar **1** and *in situ* silylated thymine with TMSOTf resulted in the smooth formation of the nucleoside **2**, with a favorable anomeric ratio α/β of approximately 85:15 (determined by ^1^H-NMR) (Figure 2). Based on our previous experience, the chemical pathway leading to the thymidine phosphoramidite bearing the DMTr group on the 5′ position does not allow the separation of anomers by standard chromatography. Therefore, and in order to introduce the modification with polarity reversal into DNA strands, we decided to introduce the DMTr group on the 7′ position. To this end, the silyl group of **2** was removed by short treatment with TBAF (→ **3**) followed by standard dimethoxytritylation (→ **4**). Separation of the two anomers by column chromatography was possible only after standard deacetylation, leading to the pure α-anomer **5** (for details on anomer separations, see Supplementary Data – 6. Anomer separation). The thymidine building block **6** was finally obtained by phosphitylation with 2-cyanoethyl N,N,N′,N′-tetraisopropylphosphorodiamidite in the presence of 5-(ethylthio)-1H-tetrazole.

The pure α-intermediate **5** also offered us a short access to the 5-methylcytosine nucleoside, by conversion of the *in situ* TMS protected nucleoside **5** to the corresponding triazolide with POCl_3_ and 1,2,4-triazole, followed by treatment in a mixture of ammonia and 1,4-dioxane. Direct protection with Bz_2_O in DMF resulted in the efficient formation of nucleoside **7**, the labile silyl protecting group being cleaved during the process. Final phosphitylation in conditions as described above afforded the 5-methylcytidine phosphoramidite **8**.

**Figure 3.**
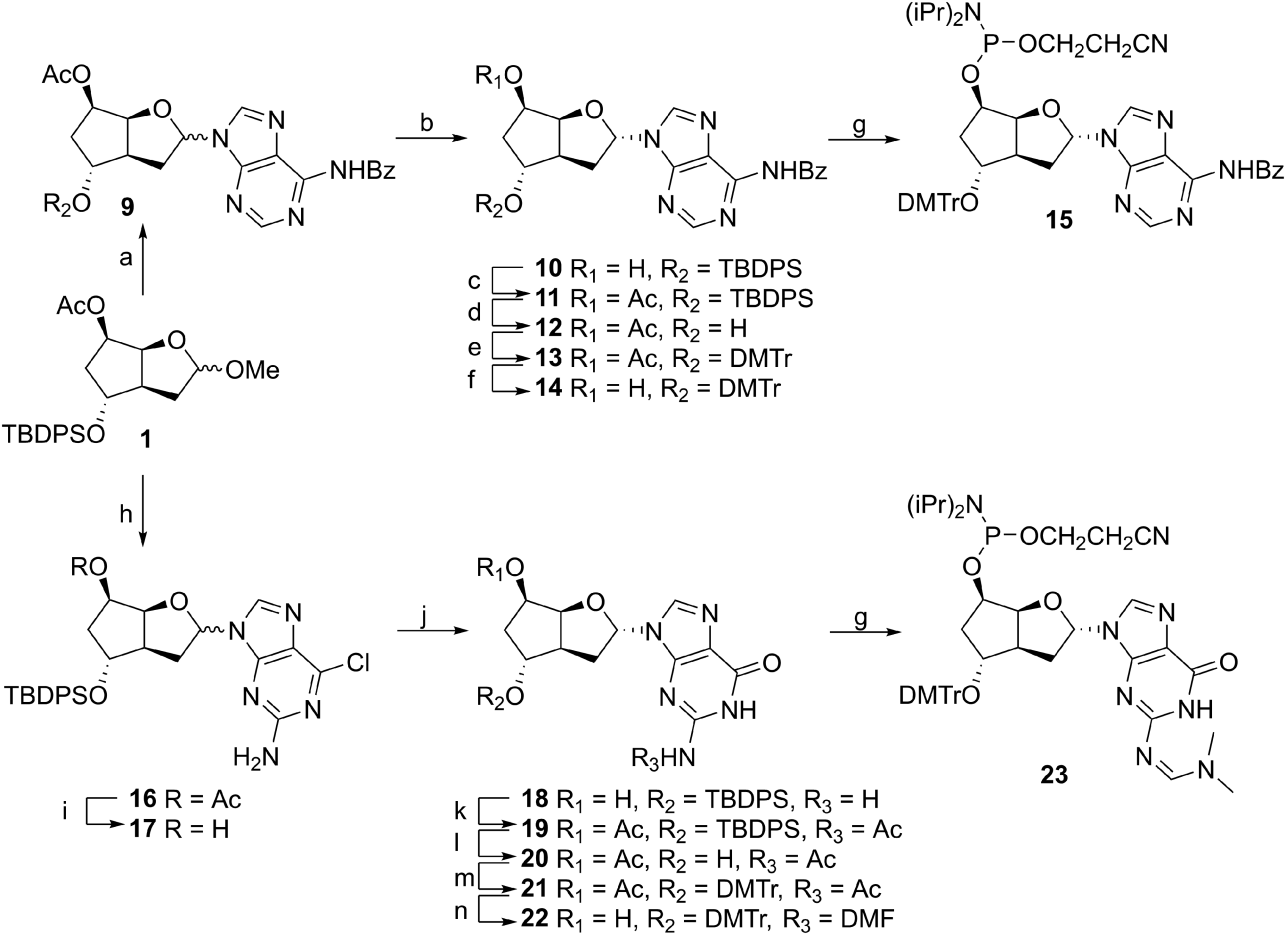
Synthesis of purine building blocks. (**a**) N^6^-Benzoyladenine, BSA, TMSOTf, MeCN, 70°C, 20 min, 64%; (**b**) NaOH, THF/MeOH/H_2_O, 0°C, 20 min, 51% α-anomer, 18% β-anomer; (**c**) Ac_2_O, DMAP, DCM, rt, 18 h, 90%; (**d**) TBAF, THF, rt, 3.5 h, 90%; (**e**) DMTr-Cl, pyridine, rt, 24 h, 89%; (**f**) NaOH, THF/MeOH/H_2_O, 0°C, 30 min, 94%; (**g**) 2-cyanoethyl N,N,N′,N′-tetraisopropylphosphordiamidite, ETT, DCM, rt, 1 h, 77% for **15**, 50 min, 67% for **23**; (**h**) 2-amino-6-chloropurine, BSA, TMSOTf, MeCN, 55°C, 50 min, 77%; (**i**) NaOH, THF/MeOH/H_2_O, 0°C, 20 min, 85%; (**j**) TBD, 3-hydroxypropionitrile, DCM, 48 h, 87%; (**k**) Ac_2_O, DMAP, DCM, rt, 48 h, 76%; (**l**) TBAF, THF, rt, 4 h, 87%; (**m**) DMTr-Cl, pyridine, rt, 48 h, 99%; (**n**) (i) K_2_CO_3_, MeOH, rt, 7 h, (ii) N,N-dimethylformamide dimethylacetal, DMF, 55°C, 2 h, 77%.

For the purine nucleosides, our synthetic strategy was dictated by our previous interest in the β-anomers. The introduction of the purines was optimized for α-nucleoside formation by a short reaction time at slightly elevated temperature with either N^6^-benzoyladenine or 2-amino-6-chloropurine, leading to the nucleoside **9** and **16**, respectively, in α/β ratios of 4:1 and 7:3 (Figure 3). In order to be able to separate the anomers, acetyl groups were removed under mild conditions, yielding the pure α-anomers **10** (used for the adenosine building block) and **17** (used for the guanosine building block), after standard column chromatography purification. The formation of the adenosine building block continues with the reintroduction of the acetyl protecting group (→ **11**), removal of the TPDPS protecting group with TBAF (→ **12**) followed by standard dimethoxytritylation (→ **13**). Selective deprotection of the acetyl group (→ **14**) followed by phosphitylation in conditions as described above yielded the adenine building block **15**.

To obtain the guanosine building block, compound **17** was converted to the guanosine nucleoside **18** by treatment with TBD and 3-hydroxypropionitrile. Acetylation over 48 h allowed the concomitant protection of the 5′-hydroxy and 2-amino groups, yielding the protected nucleoside **19**. The DMTr group was introduced by removal of the silyl protecting group with TBAF (→ **20**) followed by dimethoxytritylation (→ **21**). The two acetyl groups were removed by treatment with K_2_CO_3_ and the resulting polar product was directly protected with DMF to afford the guanosine nucleoside **22**. Final phosphitylation yielded the building block **23** (for the synthetic protocols and characterizations, see Supplementary Data)

### X-ray structures

**Figure 4.**
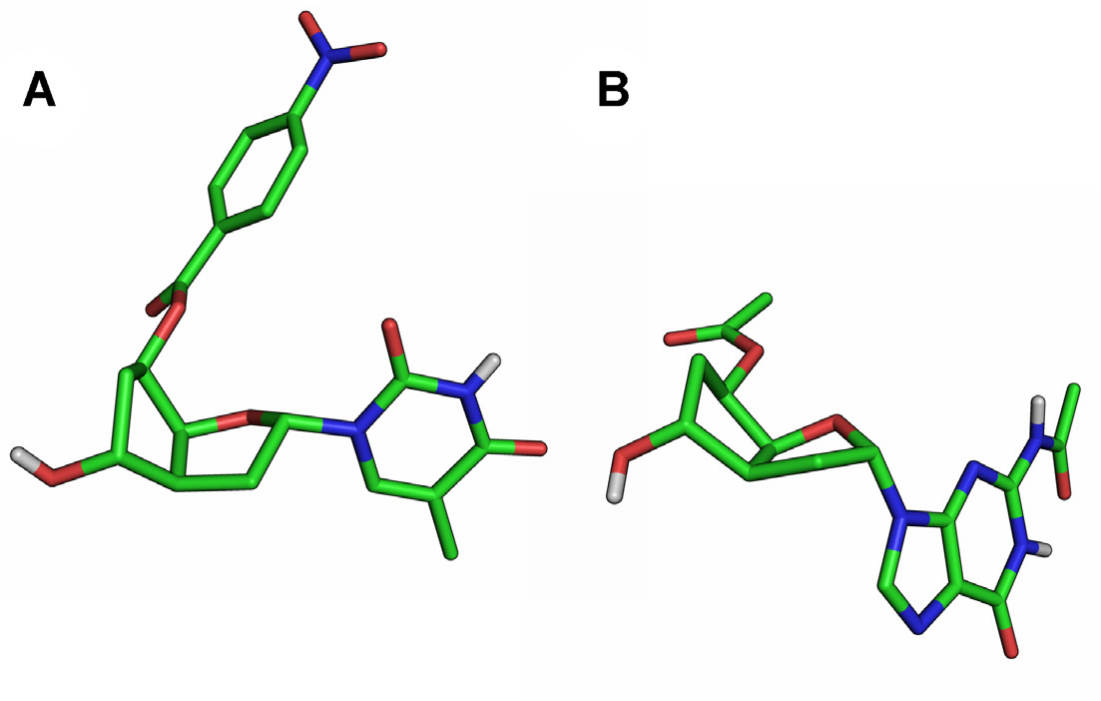
X-ray structure. (**A**) 5′-O-p-nitrobenzoyl-7′,5′-α-bc-T. (**B**) 5′-O-acetyl-7′,5′-α-bc-G^Ac^. Non-polar hydrogen atoms are omitted for clarity.

We were interested in obtaining crystals of the monomers, mainly to confirm the relative configuration of the 7′,5′-α-bc-DNA series but also to compare this structure with those found by *ab initio* calculations (see *Molecular modeling*). We succeeded to obtain crystals for the thymidine and the guanosine monomers. However, we had to introduce a p-nitrobenzoate at O5′ to be able to crystallize the T-nucleoside (for the synthesis, see Supplementary Data). This molecule co-crystallized with EtOAc giving rise to crystals of low quality. The resulting structure (Figure 4A, Supplementary Tables S3–5) adopts a C1′-endo, O4′-exo sugar pucker and a C6′-endo conformation in the carbocyclic ring. This conformation orients the C5′ substituent in a pseudoequatorial position and the C7′ hydroxyl group in a pseudoaxial position. This structure deviates in the sugar pucker from the minima predicted by *ab initio* calculations. However, further analysis demonstrated that this was due to the p-nitrobenzoate substituent (see *Molecular modeling*).

On the other hand, the protected guanosine **20** gave rise to crystals of good quality. In this case (Figure 4B and Supplementary Tables S6–10), the furanose adopts an almost perfect C1′-exo conformation, while the carbocyclic ring adopts a geometry as described above. This time, the structure matches perfectly with one of the minimal conformers predicted by *ab initio* calculation.

### Design and synthesis of oligomers

**Figure 5.**
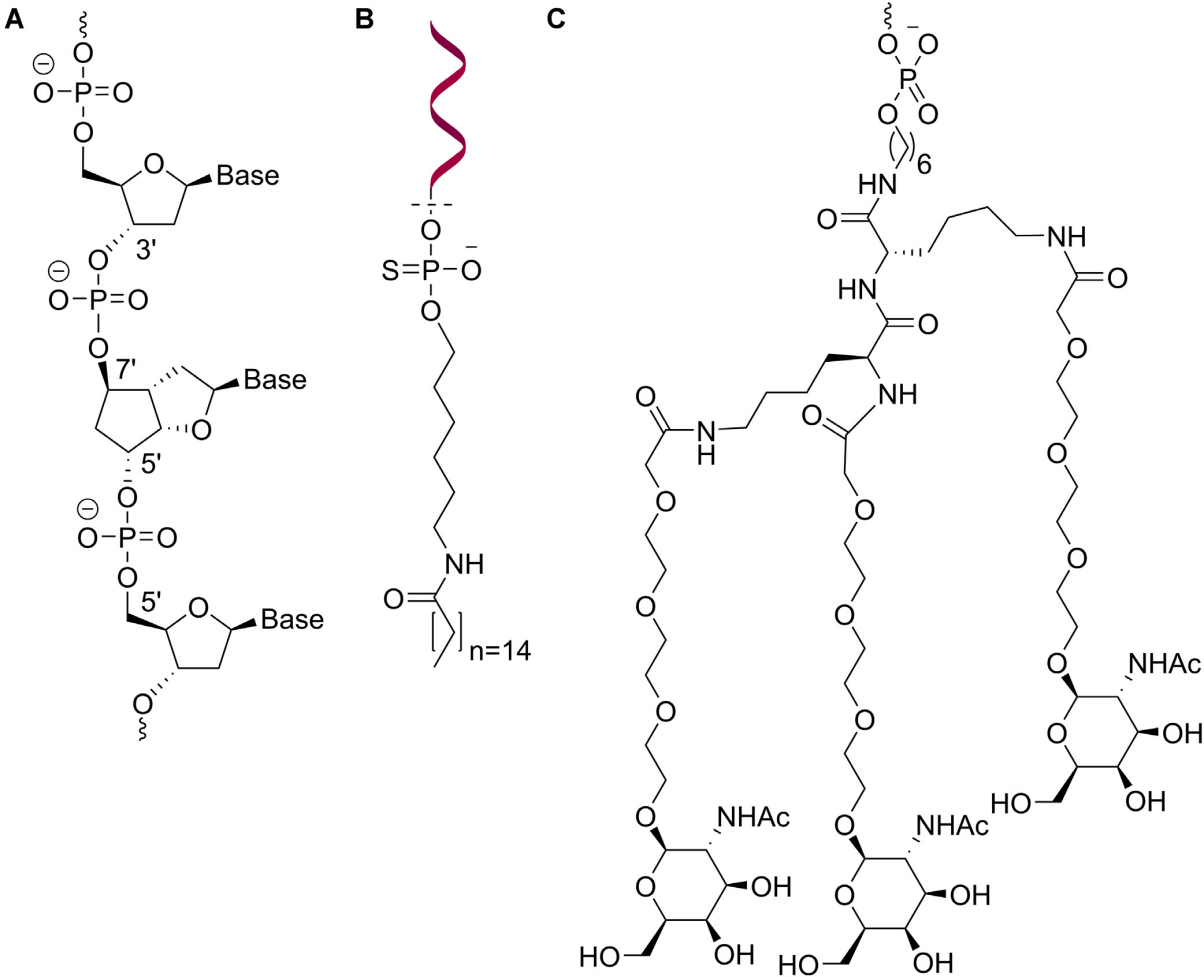
Oligonucleotide design. (**A**) Insertion of 7′,5′-α-bc-DNA with polarity reversal inside β-DNA. (**B**) Representation of the palmitic acid conjugate. (**C**) Representation of the GalNAc conjugate.

To assess the accommodation of the modification inside a natural β-DNA strand, five oligodeoxynucleotides (**ON1-5**, Table 1) with single or multiple insertions of the thymidine building block **6** were prepared. In order to fit the geometry of β-DNA, the modification was inserted with polarity reversal, resulting in 3′-7′ and 5′-5′ internucleosidic linkages (Figure 5A). To test the pairing properties of this new system with natural nucleic acid, but also toward itself, the fully modified **ON6** containing all four nucleobases, as well as its antiparallel (**ON7**) and parallel (**ON8**) fully modified complements were prepared (Table 2). In addition, two fully modified strands with either PS-linkages (**ON9**) or phosphodiester- (PO-) linkages (**ON11**), and two corresponding fully modified strands conjugated via one PS-linkage to a palmitic acid at the 7′ end (Figure 5B) and with either PS-linkages (**ON12**) or PO-linkages (**ON10**) were synthesized (Table 2), with the aim to screen their ability to induce exon skipping. Finally, a fully modified strand (**ON13**, Table 2), with PO-linkages and the sequence of the approved drug Nusinersen, was conjugated at the 7′ end to a triantennary GalNAc cluster (Figure 5C). This last oligonucleotide was designed to evaluate the *in vivo* efficacy at inducing exon inclusion in liver. The syntheses were performed using classical automated solid phase phosphoramidite chemistry. Fully modified strands were synthesized in a 5′ → 7′ direction. Complete cleavage of the DMTr protecting group from the 7′ position required a solution of 5% dichloroacetic acid in dichloroethane. In these conditions, coupling yield were > 98% based on trityl assay. Fully modified strands could be completely cleaved from universal solid support by a smooth treatment in concentrated ammonia at 55°C overnight.

**Table 1.** *T*_m_ and Δ*T*_m_/mod data from UV-melting curves (260 nm) of **ON1-5** in duplex with complementary DNA and RNA.

Entry	Sequence^a,b,c^	*T*_m_ versus DNA [°C]	Δ*T*_m_/mod [°C]	*T*_m_ versus RNA [°C]	Δ*T*_m_/mod [°C]
**ON1**	5′-d(GGA TGT TC**t** CGA)-3′	40.0	-9.1	42.6	-6.8
**ON2**	5′-d(GGA **t** GT TCT CGA)-3′	43.0	-6.1	45.8	-3.6
**ON3**	5′-d(GGA **t**GT TC**t** CGA)-3′	32.8	-8.1	38.0	-5.7
**ON4**	5′-d(GGA TG**t t**CT CGA)-3′	42.9	-3.1	47.0	-1.2
**ON5**	5′-d(GCA **ttt tt**A CCG)-3′^d^	34.0	-2.7	37.2	-1.4

^a^Total strand conc. 2 μM in 10 mM NaH_2_PO_4_, 150 mM NaCl, pH 7.0.

^b^A, G, T, C denote natural 2‘-deoxynucleosides; **t** corresponds to modified nucleosides.

^c^ *T*_m_ of unmodified duplexes, DNA/DNA: 49.1°C, DNA/RNA: 49.4°C, RNA/RNA: 60.3°C.

^d^ *T*_m_ of unmodified duplexes, DNA/DNA: 47.5°C, DNA/RNA: 44.0°C.

**Table 2.** *T*_m_ and Δ*T*_m_/mod data from UV-melting curves (260 nm) of **ON6-13** in duplex with complementary parallel DNA and RNA.

Entry	Sequence^a,b^	*T*_m_ versus parallel DNA [°C]	Δ*T*_m_/mod [°C]	*T*_m_ versus parallel RNA [°C]	Δ*T*_m_/mod [°C]
**ON6**	5′-d(**agc tct tgt agg**)-7′^c^	43.2	-0.5	65.0	1.3
**ON7**	5′-d(**cct aca aga gct**)-7′^d^	43.8	-0.4	58.6	1.3
**ON8**	5′-d(**tcg aga aca tcc**)-7′^e^	47.7	-0.1	61.6	1.5
**ON9**	5′-d(t*c*c*a*t*t*c*g*g*c*t*c*c*a*a)-7′^f^	43.2	-1.3	77.0	0.6
**ON10**	5′-d(**tccattcggctccaa**)-7′*P^f^	54.3	n.d.	76.8	n.d.
**ON11**	5′-d(**tccattcggctccaa**)-7′^f^	55.9	-0.4	79.2	0.8
**ON12**	5′-d(t*c*c*a*t*t*c*g*g*c*t*c*c*a*a)-7′*P^f^	42.6	n.d.	69.1	n.d.
**ON13**	5′-d(**ggtcgtaatactttcact**)7′-GalNAc	n.d.	n.d.	66.0	n.d.
**MOE1**	GalNAc-5′-(t*c*a*c*t*t*t*c*a*t*a*a*t*g*c*t*g*g)-3′	n.d.	n.d.	67.4	n.d

n.d. = not determined

^a^Total strand conc. 2μM in 10 mM NaH_2_PO_4_, 150 mM NaCl, pH 7.0.

^b^ **a**, **g**, **t**, **c** corresponds to modified adenine, guanine, thymine and methylcytosine respectively, a, g, t, c corresponds to 2‘-O-(2-methoxyethyl) adenine, guanine, thymine and methylcytosine respectively, * denotes a phosphorothioate linkage, P corresponds to palmitic acid, GalNac corresponds to a *N*-acetylgalactosamine conjugate.

^c^ *T*_m_ of unmodified duplexes, DNA/DNA: 49.1°C, DNA/RNA: 49.4°C, RNA/RNA: 60.3°C

^d^ *T*_m_ of unmodified duplexes, DNA/DNA: 49.1°C, DNA/RNA: 43.0°C, RNA/RNA: 60.3°C

^e^ *T*_m_ of unmodified duplexes, DNA/DNA: 49.0°C, DNA/RNA: 43.3°C, RNA/RNA: 57.3°C

^f^ *T*_m_ of unmodified duplexes, DNA/DNA: 62.0°C, DNA/RNA: 67.4°C, RNA/RNA: 74.3°C

### Pairing properties of modified oligodeoxynucleotides with complementary DNA and RNA

The duplex stabilities of the modified oligonucleotides were assessed by UV melting curves at 260 nm, and their *T*_m_s were compared to their natural DNA analogs (Table 1). Oligonucleotides **ON1-2** with a single incorporation resulted in a strong destabilization with DNA complements and slightly lower destabilization with RNA. This penalty appears to be cumulative as **ON3**, with the two previous positions modified, further decreased the *T*_m_s. However, when two modifications were introduced consecutively (**ON4**), the destabilization per modification is reduced to −3.1°C versus DNA and −1.2°C versus RNA. Similarly, when five modifications were introduced consecutively in different sequence context (**ON5**), the destabilization per modification remained at −2.7°C versus DNA and −1.4°C versus RNA, when compared to its respective natural DNA analog. These data suggest that a junction between the DNA and the 7′,5′-α-bc-DNA backbone induces a strong destabilization, with a reduction of *T*_m_ between −4 and −9°C, depending on the sequence context. Such destabilizations by heterobackbone junctions have already been observed for α-DNA (26,27) and for α-LNA (28). This destabilization could be compensated by inserting multiple consecutive modifications.

### Pairing properties of fully modified oligonucleotides

**Figure 6.**
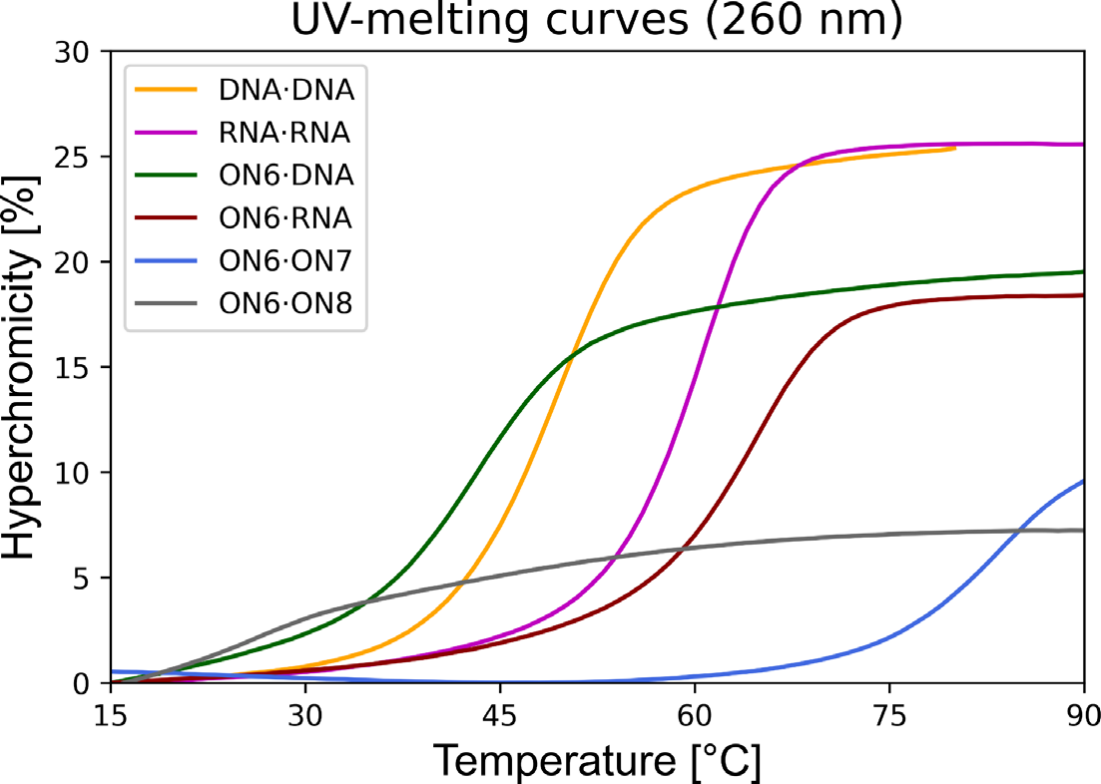
UV-melting curves (260 nm) of **ON6** with fully modified parallel (**ON7**) and antiparallel (**ON8**) complement, parallel DNA and parallel RNA, in comparison with the corresponding natural DNA and RNA duplexes. Total strand conc. 2μM in 10 mM NaH_2_PO_4_, 150 mM NaCl, pH 7.0

All three fully modified sequences **ON6-8** exhibit a cooperative and reversible melting behavior with their parallel DNA and RNA complements (Figure 6) but not with their antiparallel complements. The resulting 7′,5′-α-bc-DNA/DNA duplexes are slightly less stable than their natural counterparts, with a destabilization between −0.1 and −0.5°C per modification (Table 2). On the other hand, we were pleased to see that **ON6-8** formed very stable duplexes with RNA, resulting in a stabilization between 1.3 and 1.5°C per modification.

To test the mismatch discrimination, we performed UV melting experiments with **ON6** and its parallel DNA complements possessing all three alternative nucleobases at the position 4 (Supplementary Table S2). Such mispairings had a strong destabilizing effect and reduced the *T*_m_s by −9.6 to −14.3°C. The discrimination in the hybrid duplexes was higher by −1.0 to −2.4°C as compared to the corresponding mismatched DNA duplexes, indicating higher base pairing selectivity for the 7′,5′-α-bc-DNA modification.

**ON9-13**, used for further exon splicing modulation experiments, also displayed high affinity for their RNA complement. The presence of the terminal palmitic acid moiety induced a slight destabilization (−2.4°C) for the oligonucleotides with PO-linkages (**ON11** versus **ON10**) but a substantial destabilization (-7.9°C) for the oligonucleotides with PS-linkages (**ON9** versus **ON12**).

In its own series, **ON6** formed a very stable duplex toward its antiparallel complement **ON7**, resulting in an unexpected *T*_m_ of 83.6°C. Due to this high *T*_m_, the complete classical sigmoidal transition could be observed only in the absence of sodium chloride, decreasing the *T*_m_ to 68.6°C (Table 3 and Supplementary Figure S3). The formation of stable secondary structures due to self-complementarity would result in a reduced ΔG for the hetero-duplex formation with complementary RNA, consequently impairing any potential biological applications. Thus, any sequences prone to form secondary structures should be avoided during the drug design.

**Table 3.** *T*_m_ values from UV-melting curves (260 nm) of **ON7-8** and **Tc1** in duplex with **ON6**.

Entry	Sequence^a,b^	NaCl concentration [mM]	Δ*T*_m_ versus **ON6**^c^ [°C]
**ON7**	5’-d(**cct aca aga gct**)-7′	150	83.6
**ON7**	5′-d(**cct aca aga gct**)-7′	50	79.6
**ON7**	5′-d(**cct aca aga gct**)-7′	0	68.6
**ON8**	5′-d(**tcg aga aca tcc**)-7′	150	<10
**Tc1**	5′-d(**tcg agaacatcc**)-3′	150	81.0

^a^Total strand conc. 2μM in 10 mM NaH_2_PO_4_, pH 7.0.

^b^ **a**, **g**, **t**, **c** corresponds to modified adenine, guanine, thymine and methylcytosine, respectively, **a**, **g**, **t**, **c** corresponds to tricyclo-DNA adenine, guanine, thymine and methylcytosine, respectively.

^c^ **ON6** sequence: 5′-d(**agc tct tgt agg**)-7′.

Interestingly, duplex formation resulted in a low hypochromicity of only 10% (Figure 6). This is an indication of a base stacking geometry differing from that of a classical helix, pointing to a structure deviating from canonical A- or B-DNA. On the other hand, no sigmoidal melting transition has been observed between **ON6** and its parallel complement **ON8** (Figure 6). The change in hypochromicity occurring does not differ from the UV melting experiments performed on the two single strands separately. To test the ability of 7′,5′-α-bc-DNA to fit inside an A-like helix, we also performed a melting experiment with tricyclo-DNA, a conformationally constrained mimic of RNA, as a partner (29). When **ON6** is mixed with complementary parallel tricyclo-DNA strand (**Tc1**), a surprisingly high *T*_m_ of 81°C was observed, demonstrating the ability of 7′,5′-α-bc-DNA to recognize also this helix geometry.

### Thermodynamic data of duplex formation

The thermodynamic data for duplex formation of **ON6** with DNA and RNA and their natural counterpart have been extracted by curve fitting to the experimental melting curves, following an established methodology (30) (Table 4). As expected, the free energy Δ*G* at 25°C follows the same trend as the *T*_m_ data, with **ON6·RNA** being the most favored duplex. **ON6** binds to natural nucleic acids with a less favorable enthalpy change compared to the natural system. However, this is compensated by concomitant less entropic loss. This behavior is typical in the bc-DNA series and arises from the conformational rigidity added by the ethylene bridge. Interestingly, the selectivity of 7′,5′-α-bc-DNA for RNA over DNA is mostly enthalpically driven.

**Table 4.** Thermodynamic data of duplex formation.

Duplex	Sequences^a^	Δ*H* [kcal mol^−1^]	Δ*S* [cal mol^−1^ K^−1^]	Δ*G*^25°C^ [kcal mol^−1^]
**DNA·DNA**	5′-GGA TGT TCT CGA-3′ 3′-CCT ACA AGA GCT-5′	−91.7	−257.9	−14.8
**DNA·RNA**	5′-GGA TGT TCT CGA-3′ 3′-CCU ACA AGA GCU-5′	−92.1	−258.3	−15.0
**ON6·DNA**	7′-**gga tgt tct cga-**5′ 3′-CCT ACA AGA GCT-5′	−79.7	−224.2	−12.9
**ON6·RNA**	7′-**gga tgt tct cga-**5′ 3′-CCU ACA AGA GCU-5′	−83.9	−222.0	−17.7

^a^A, G, T, U, C denote natural nucleosides; **a**, **g**, **t**, **c** corresponds to modified adenine, guanine, thymine and methylcytosine, respectively.

### CD spectroscopy

**Figure 7.**
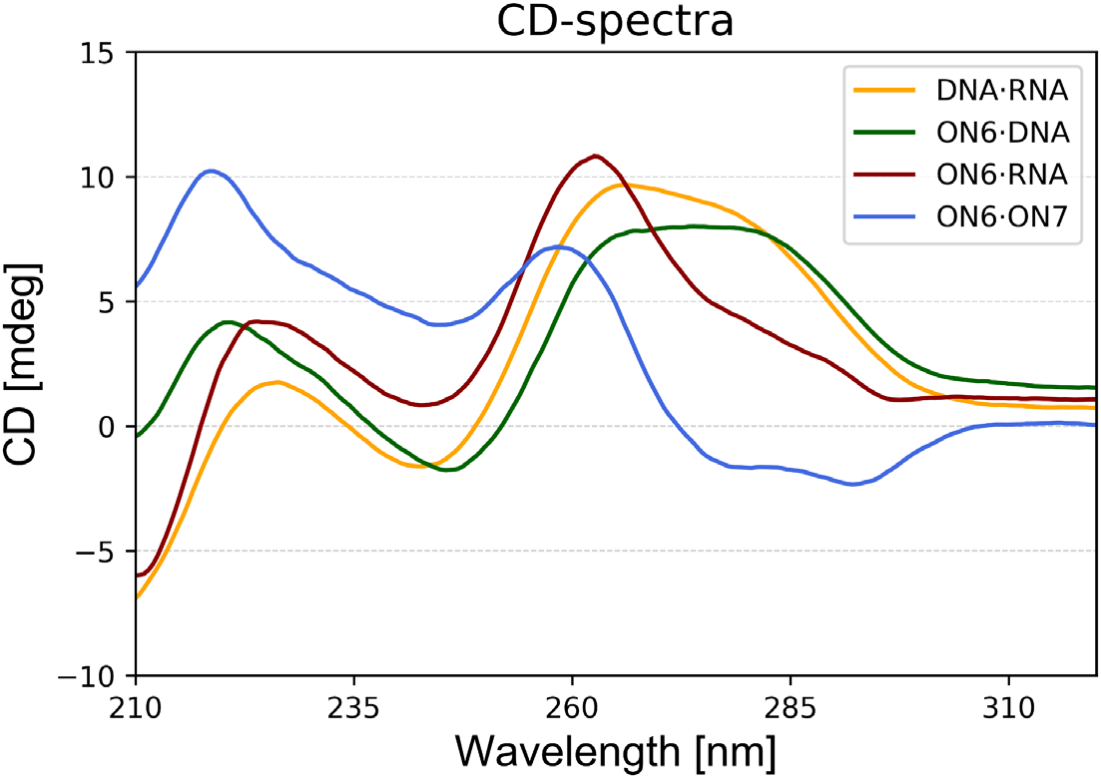
CD-spectra at 20°C of **ON6** with parallel DNA, parallel RNA and fully modified parallel complement (**ON7**), in comparison with the corresponding natural DNA·RNA duplex. Experimental conditions: Total strand conc. 2 μM in 10 mM NaH_2_PO_4_, 150 mM NaCl, pH 7.0.

The CD spectra of **ON6** in duplex with DNA, RNA or **ON7** have been measured and compared with the corresponding natural DNA/RNA duplex (Figure 7). Both duplexes of **ON6** with DNA or RNA have a CD signature relatively close the natural A/B-helix. However, the **ON6**/DNA duplex doesn′t display a negative signal at 210 nm and has the ellipticity at 226 nm blue shifted by 5 nm and associated with a gain in amplitude. The **ON6**/RNA duplex also has a peak of higher positive amplitude at 226 nm, and the positive ellipticity at 266 nm is blue shifted by 4 nm and formed a sharper peak. On the other hand, the modified homo-duplex has a very atypical CD signature, characterized by a broad negative ellipticity between 275 and 300 nm of small amplitude and two positive peaks at 259 and 218 nm. In agreement with the low hypochromicity change upon duplex formation, the CD spectra of the homo-duplex indicates the formation of a structure deviating from canonical A- or B-DNA.

### Molecular modeling

**Figure 8.**
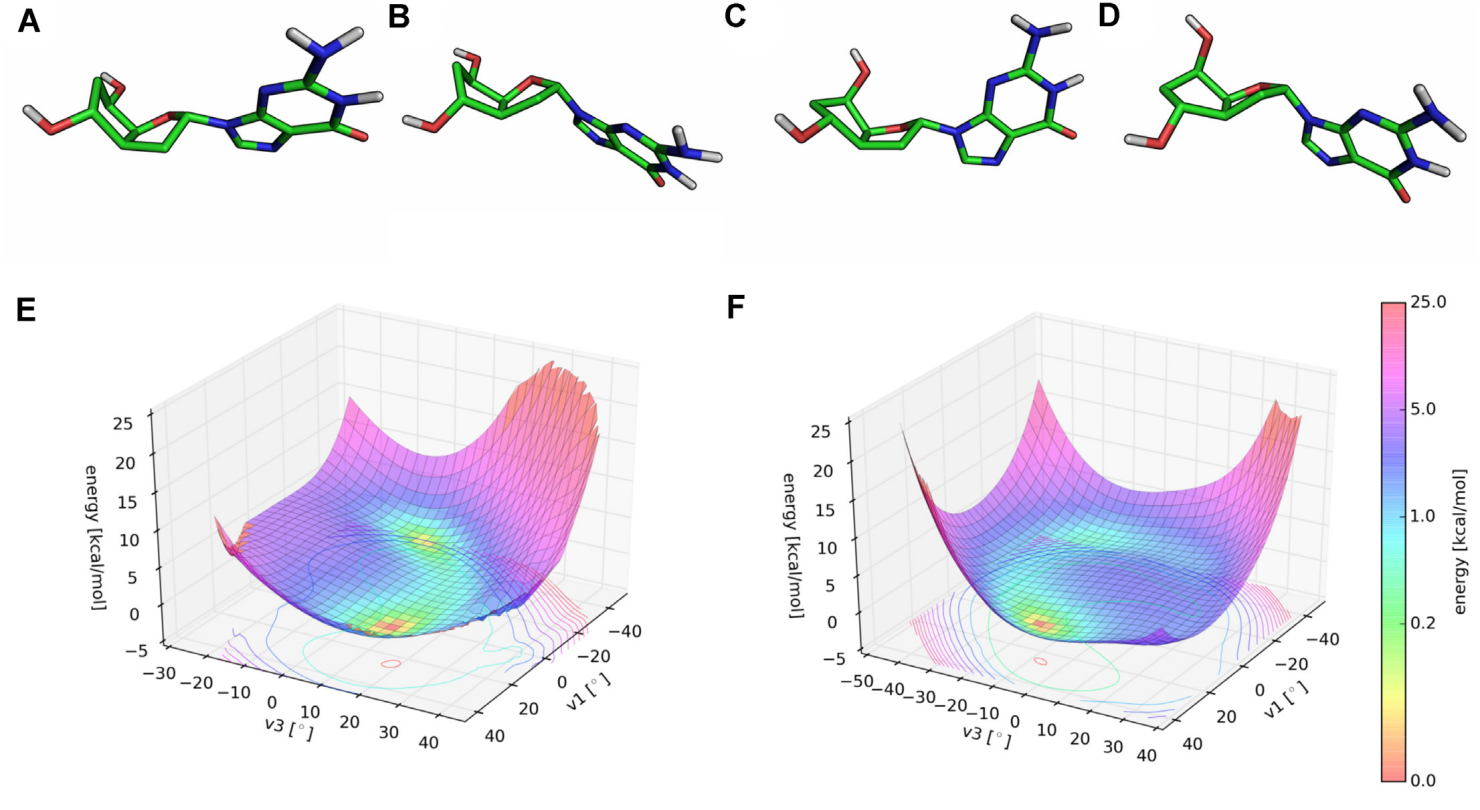
Conformational analyses. The four minimum energy conformers of 7′,5′-α-bc-DNA-G nucleoside: (**A**) conformer a, (**B**) conformer b, (**C**), conformer c, (**D**) conformer d. Potential energy surface starting from (**E**) C6′-endo conformation and (**F**) C6′-exo conformation.

To gain a deeper understanding on the 7′,5′-α-bc-DNA system and the structure of its duplexes, we performed extensive molecular modeling. We first analyzed the potential energy surfaces (PES) of the four monomers. The conformational analyses were done at the Hartree-Fock *ab initio* level, by varying ν_1_ and ν_3_ in a stepwise manner. For each monomer, two scans were performed, starting from a conformation with C6′ in an endo or exo conformation. These calculations showed the existence of four distinct minimal conformations close in energy for each nucleoside (Figure 8A−D, relevant parameters are summarized in Table 5). The PES were similar for the four nucleobases (Figure 8E and F), however, each nucleoside had different absolute minima. Despite the rigidity added by the ethylene bridge, the scaffold of 7′,5′-α-bc-DNA seems to allow a certain degree of residual flexibility. This is reflected by the flat region of the PES, indicating that the sugar could easily shift between different conformations. Therefore, all conformers are relevant structures and have been used to calculate the charges for the four nucleobases using the R.E.D. III.5 tools package (19). These charges were then used to modified the Amber94 force field for 7′,5′-α-bc-DNA.

**Table 5.** Summary of the relevant parameters for each conformer. Δ*E* have been calculated taking the absolute minima for each nucleoside as reference.

Nucleoside		Phase [°]	Carbocyclic ring conformation	γ [°]	χ [°]	Δ*E* in vacuo [kcal mol^−1^]
Thymine	Conformer a	349	C5′-exo,C6′-endo	162	161	0.409
	Conformer b	110	C6′-endo	153	175	0.884
	Conformer c	301	C6′-exo	89	141	0
	Conformer d	176	C5′-endo	77	166	0.469
Methylcytosine	Conformer a	350	C5′-exo,C6′-endo	162	167	0
	Conformer b	107	C6′-endo	154	174	0.604
	Conformer c	303	C6′-exo	90	163	0.393
	Conformer d	176	C5′-endo	77	167	0.608
Adenine	Conformer a	349	C5′-exo,C6′-endo	162	165	0.669
	Conformer b	114	C6′-endo	153	-173	0
	Conformer c	303	C6′-exo	89	144	0.136
	Conformer d	163	C5′-endo	78	175	0.021
Guanine	Conformer a	349	C5′-exo,C6′-endo	162	155	0.881
	Conformer b	114	C6′-endo	152	-177	0.382
	Conformer c	300	C6′-exo	88	139	0.056
	Conformer d	164	C5′-endo	78	171	0

**Figure 9.**
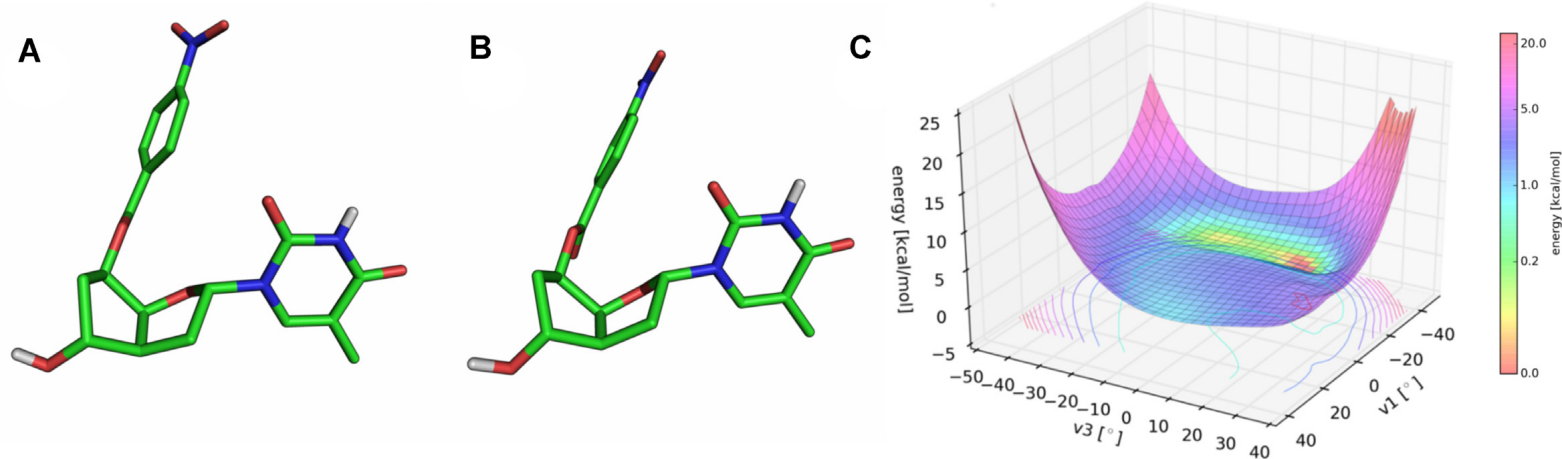
Comparison between X-ray structure and molecular model. (**A**) X-ray structure of the 5′-O-p-nitrobenzoyl-7′,5′-α-bc-T. (**B**) Minimal conformation calculated for the 5′-O-p-nitrobenzoyl-7′,5′-α-bc-T and (**C**) its corresponding PES.

To understand the deviation observed between the X-ray structure of the o-paranitrobenzoyl thymidine nucleoside (Figure 9A) and the *ab initio* calculations of the unprotected nucleoside, a conformational search was performed for the protected T nucleoside. The resulting PES differs from the PES of the free nucleoside, indicating that the 5′-substitution strongly influences the sugar pucker (Figure 9C). In this case, the minimal energy conformer obtained (Figure 9B) perfectly matches with the X-ray structure (Figure 9A), demonstrating the relevance of the *ab initio* calculations.

**Figure 10.**
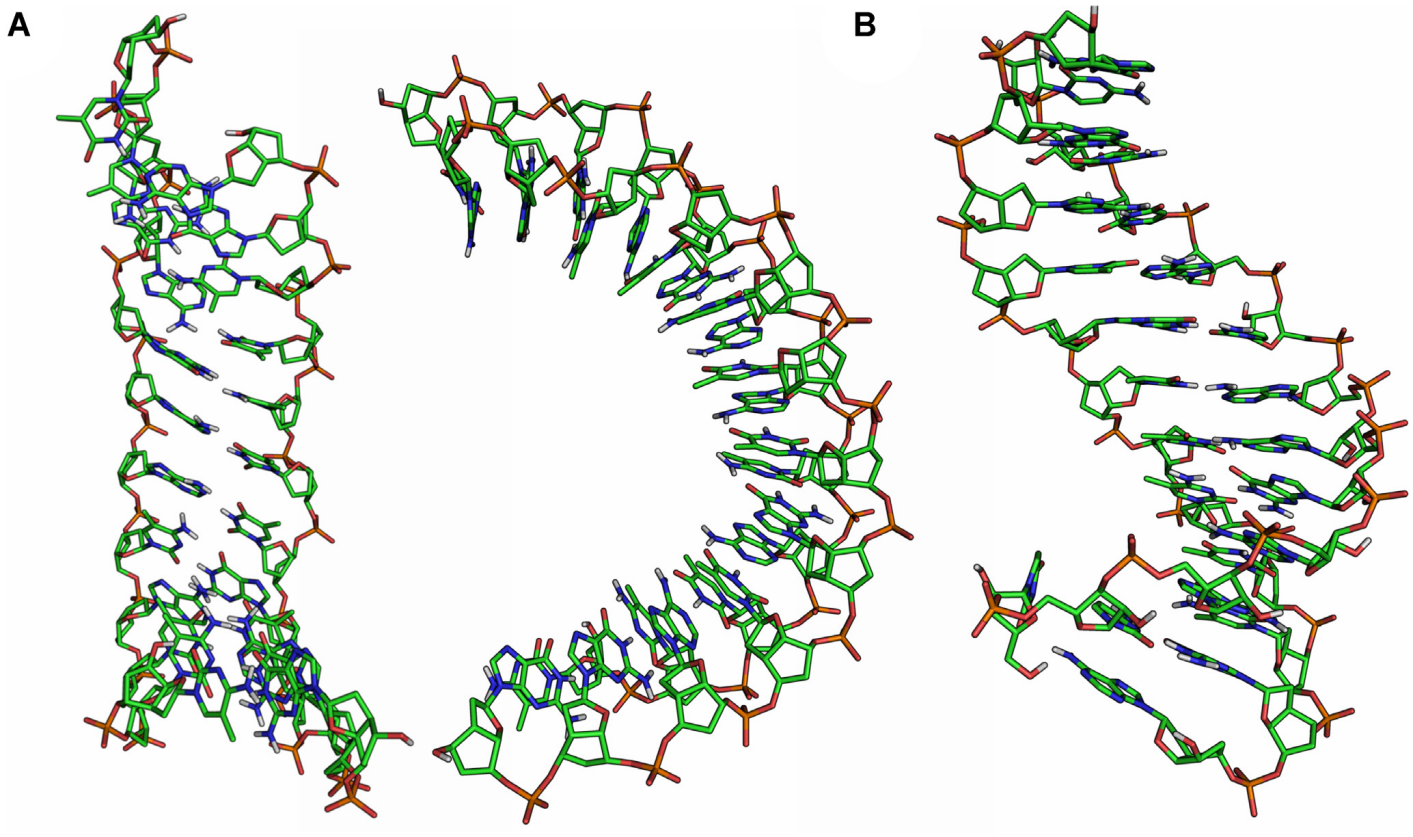
Last frame of the 60 ns simulation for (**A**) 7′,5′-α-bc-DNA homo-duplex and **(B**) 7′,5′-α-bc-DNA/RNA duplex.

**Figure 11.**
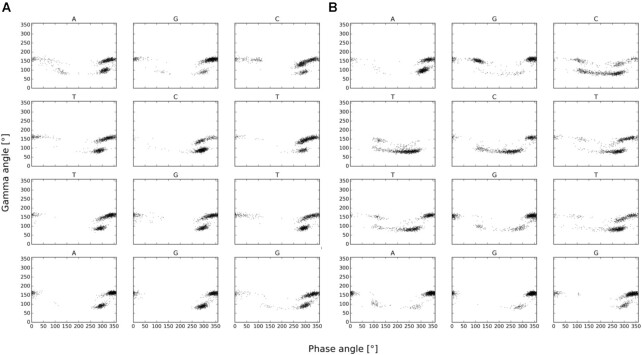
Plots of the phase angle versus γ angle for each snapshot during the last 50 ns of the simulation for (**A**) **ON6** in the context of 7′,5′-α-bc-DNA homo-duplex and (**B**) **ON6** in the context 7′,5′-α-bc-DNA/RNA duplex.

The solvated duplex structures formed by **ON6**/**ON7** and **ON6**/RNA were simulated on a 60 ns molecular dynamics trajectory with the GROMACS 5.0.6 simulation package. Due to the higher affinity of 7′,5′-α-bc-DNA for RNA, the duplexes were constructed starting from an A-helix. However, the structure of the homo-duplex rapidly converged toward a ladder-like structure with a strong inclination between backbone and nucleobase axes (Figure 10A). The duplex adopts an overall curved shape, with the minor groove oriented toward the convex side. This atypical structure possesses some striking similarities with the NMR-structure of an α-L-arabinopyranosyl-(4′→2′)-NA homo-duplex (31). Interestingly, complementary α-L-arabinopyranosyl-(4′→2′)-DNA strands with all four nucleobases also form homo-duplexes with low hypochromicity change and a CD signature characterized by a negative band of small amplitude between 280 and 300 nm and two positive peaks at 210 and 260 nm (32). Obviously, there seem to be substantial similarities between the arabinopyranosyl-(4′→2′)-NA and the 7′,5′-α-bc-DNA structures. To visualize the furanose conformations in the **ON6/ON7** homo-duplex, we plotted the phase angles in function the gamma angles for each snapshot during the last 50 ns of the simulation (Figure 11A). We found that the furanose units adopt a North conformation, while the γ angles alternate between a + *sc* and *ap* arrangement, corresponding to the conformer a and c (Table 5).

As suggested by CD spectroscopy, the **ON6**/RNA forms a more conventional helical structure (Figure 10B), with an average of approximately 14 bases per turn. As before, the γ angles alternate between a + *sc* and *ap* arrangement (Figure 11B). But in this case, the furanose puckers of 7′,5′-α-bc-DNA span a surprisingly wide spectrum of P-angles, indicating that the sugars can easily adopt the four minimal conformers (Table 5). The RNA residues remain in a natural conformation usually founded in A-helix, with the sugars oriented in a C3′-endo puckering. In agreement with *ab initio* calculation, the molecular dynamics simulation indicates that the scaffold of 7′,5′-α-bc-DNA allows a certain degree of structural adaptability. Therefore, the 7′,5′-α-bc-DNA system is able to properly fit into helical geometry and thus to cross-pair efficiently with natural nucleic acids.

### Biological stability

An important prerequisite for an effective oligonucleotide drug is to remain stable in biological media. Thus, we decided to test the biostability of the 7′,5′-α-bc-DNA chemistry by incubating **ON6** with PO-internucleosidic linkages in human serum. Analysis by gel electrophoresis (Supplementary Figure S1) demonstrated that **ON6** remained completely stable even after 24 h, while its corresponding natural DNA was substantial digested already after 1 h and complete digested after 4 h. We then tested the ability of the 7′,5′-α-bc-DNA chemistry to remain stable in the acidic endosomal conditions. **ON10** was incubated in phosphate buffer (pH 4.5) at 37°C for 24 h. Analysis by LC-MS showed no substantial decomposition of **ON10** (Supplementary Figure S2). Thus, the 7′,5′-α-bc-DNA modification appears to confer a sufficient biostability for biological applications.

### Exon splicing modulation activity

The splice-switching activity of the 7′,5′-α-bc-DNA scaffold was assessed *in vitro* by transfection and gymnotic delivery in C2C12 cells and *in vivo* by intra-muscular injections in the *mdx* mouse. This Duchenne muscular dystrophy animal has a nonsense mutation in exon 23 of the *Dmd* gene, which abolishes dystrophin production. Exon 23 skipping would allow the production of internally deleted dystrophin. This model was chosen as it is commonly used to evaluate splice-switching oligonucleotides (33). Our design involved a fully modified 15-mer sequence targeting exon 23 that was successfully tested *in vivo* with tricyclo-DNA chemistry in the recent past (34). Thus, **ON9** composed of a PS-backbone and **ON10** composed of a normal PO-backbone were initially investigated *in vitro*. We were interested to include the PO-backbone, as our modified scaffold confers already sufficient biostability. However, to overcome the poor cellular uptake of the PO-backbone (35), **ON10** was conjugated to palmitic acid in order to assure efficient complexation with the transfection reagent or to improve the cellular uptake in naked delivery (36). The positive control was a 2′-OMe-RNA with PS-backbone and an extended sequence of 20-nt (3′-uccauucggcuccaaaccgg-5′), which has previously demonstrated good efficacy *in vitro* and *in vivo* (37,38). The negative control was a 2′-OMe-RNA oligonucleotide with PS-backbone targeting an unrelated gene transcript (ALK4).

**Figure 12.**
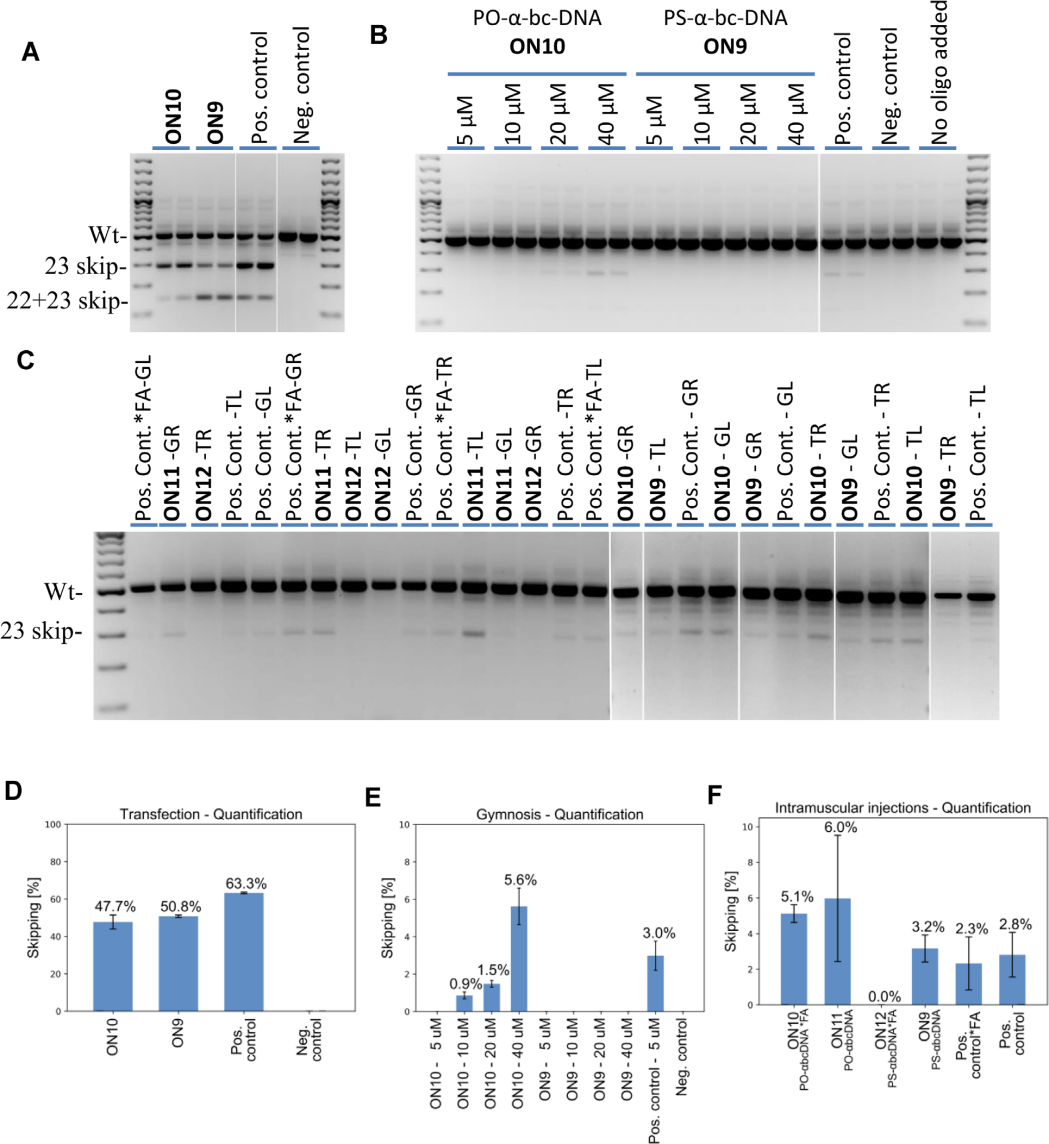
(**A**) Cropped agarose gel for mouse exon 23 and exon 22 + 23 skipping efficacy after transfection detected by nested RT-PCR. (**B**) Combined agarose gel for mouse exon 23 and exon 22 + 23 skipping efficacy after gymnosis detected by nested RT-PCR. (**C**) Combined agarose gels for mouse exon 23 and exon 22 + 23 skipping efficacy after intramuscular injections in gastrocnemius right (GR) and left (GL) and the triceps right (TR) and left (TL) to mice detected by nested RT-PCR. Quantification of antisense activity. (**D**) Quantification (*n*= 2) with ImageJ software for mouse exon 23 and exon 22 + 23 skipping efficacy after transfection detected by nested RT-PCR. (**E**) Quantification (*n*= 2) with ImageJ software for mouse exon 23 and exon 22 + 23 skipping efficacy after gymnosis detected by nested RT-PCR. (**F**) Quantification of skipped products with a lab-on-a-chip for intramuscular injections in mdx mice (*n*= 4 for **ON10-12** and positive controls, *n*= 3 for **ON9**). *FA denotes a conjugation to a fatty acid (palmitic acid). The skipping efficiencies are reported as the average of the quantified values and the error bars represent the standard deviations.

When **ON9-10** were delivered into cells with the help of a transfecting reagent, both induced robust exon 23 skipping (Figure 12A,D). Interestingly, we detected for all oligonucleotides the formation of a product corresponding to exon 23 skipping and also a fragment corresponding to exon 22 and 23 double skipping, with a ratio depending on the chemistry. Overall, **ON9-10** have a similar exon skipping activity (50.8% and 47.7%, respectively), slightly lower than the positive control (63.3%) that is longer by five nucleotides. This transfection experiment clearly shows that the 7′,5′-α-bc-DNA scaffold has the capacity to induce exon skipping and works as an efficient splice-switching oligonucleotide when delivered into cells.

We then evaluated the ability of the 7′,5′-α-bc-DNA scaffold to induce *in vitro* exon skipping without any transfecting reagent by performing gymnotic experiments at escalating doses (Figure 12B,E). In such conditions, the PS-7′,5′-α-bc-DNA (**ON9**) was not able to induce any detectable exon skipping, even at a high dose of 40 μM. The PO-7′,5′-α-bc-DNA (**ON10**) induced detectable but weak exon skipping at 10 and 20 μM (<2%), and relevant exon sipping only at 40 μM (5.6%). In comparison, the PS-2′-OMe positive control induced relevant exon skipping (3.0%) already at a dose of 5 μM. In overall, we did not observe any signs of cellular toxicity in our *in vitro* experiments.

We were interested to test our new scaffold *in vivo* by intramuscular injections. To screen the impact of the backbone chemistry and of the fatty acid (FA) conjugation, we included **ON11** (unconjugated PO-7′,5′-α-bc-DNA), **ON12**, (PS-7′,5′-α-bc-DNA conjugated to FA), as well as the positive control conjugated at the 5′-end to a palmitic acid (Pos. control*FA). Fifty micrograms of splice-switching oligonucleotide was injected twice on two consecutive days in the gastrocnemius and triceps and the skipped product was quantified after 10 days (Figure 12C,F). Interestingly, the two PO-7′,5′-α-bc-DNA constructs (**ON10-11**) displayed the strongest exon skipping activities (5.1 and 6.0%), demonstrating that a PO-backbone is a valuable option for our new modification. In the context of local delivery, the fatty acid conjugation seems to negatively impact the activity. This is surprisingly evident for PS-7′,5′-α-bc-DNA, as the unconjugated construct (**ON9**) displayed a similar efficacy as the PS-2′-OMe positive control (3.2% and 2.8% respectively), but the conjugate (**ON12**) rendered no activity. We have shown previously that the presence of the fatty acid decreases the *T*_m_s with complementary RNA, especially for PS-7′,5′-α-bc-DNA. Thus, this decreased activity might in part be explained by a lower target affinity.

**Figure 13.**
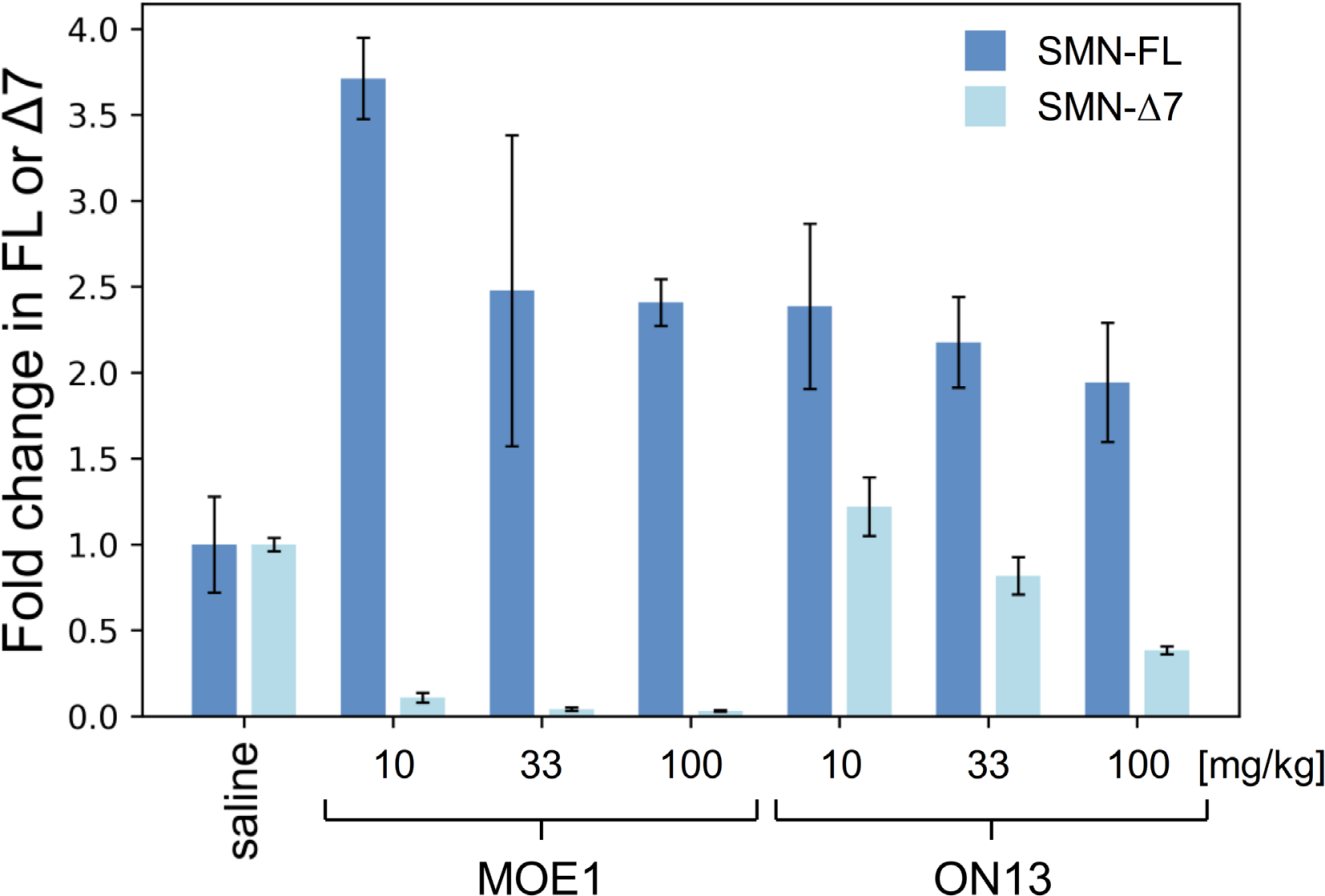
Quantification by ddPCR of the level of SMN transcript including exon 7 (SMN-fl) or excluding exon 7 (SMN-Δ7), after subcutaneous injection with **ON13** or **MOE1** at 10, 33 or 100 mg/kg, or with a saline solution (*n*= 3 for each cohort, all ddPCR experiment were performed in duplicate). The transcripts levels are reported as the average of the quantified values and the error bars represent the standard deviations. The transcript levels were normalized by taking the negative control cohort as reference.

Next, we evaluated the efficacy of oligonucleotide to stimulate exon 7 inclusion of survival motor neuron (SMN) in the liver of transgenic SMA mice, after subcutaneous injection. We opted for this alternative and previously described model (17), as there is still no robust strategy to efficiently deliver oligonucleotides to muscular tissues. The positive control (**MOE1**, Table 2) corresponds to Nusinersen, an approved drug for SMA, conjugated to a GalNAc. Our test compound is a PO-7′,5′-α-bc-DNA GalNAc-conjugate (**ON13**), with the sequence of Nusinersen. Seven-week-old mice were injected subcutaneously with **ON13** or **MOE1** (single dose of 10, 33 or 100 mg/kg) and were sacrificed 72 h after treatment. The levels of the SMN-fl and SMN-Δ7 were quantified in the liver by ddPCR (Figure 13). Both **ON13** and **MOE1** were efficiently able to induce the formation of the SMN-fl, the therapeutic transcript, at all tested doses. This indicates that our modification might have achieved a therapeutically relevant efficacy even with a PO-backbone. However, a discrepancy can be observed on SMN-Δ7 levels. Already at the lowest dose, **MOE1** induced a strong down-regulation of SMN-Δ7, contrasting with the data reported for similar constructs (17). On the other hand, **ON13** led to a significant down-regulation of SMN-Δ7 only at the highest dose. In overall, our results indicate that our new modification can have an efficacy by systemic treatment.

## DISCUSSION

Epimerization at the C1′ position of the 7′,5′-bc-nucleosides strikingly impacts the structural and the pairing properties of the 7′,5′-bc-DNA systems. Based on molecular modeling, we previously showed that the β-nucleosides steeply converged toward two minimums, giving rise to rigid oligonucleotides. Single insertion of the β-nucleosides can still stabilize DNA duplexes, but fully modified strands are constrained in a non-canonical structure and thus hardly cross-pair with their natural complement (16). Conversely, the α-nucleosides converged toward four minimums separated by low energy barriers, resulting in oligonucleotides able to adapt to different duplex geometries. Thus, as indicated by molecular modeling and corroborated by spectroscopic data, the 7′,5′-α-bc-DNA system can from a ladder-like structure in its own series or stable helical structures with natural nucleic acids. The higher flexibility of the α-anomers probably results from the nucleobase orientation pointing toward the convex side of the bicyclic sugar scaffold, leading to reduced steric interactions and thus access to a larger conformational space. But despite this relative furanose flexibility, the bicyclic structure still confers a certain level of preorganization, resulting in favored entropic terms upon duplex formation. Therefore, the 7′,5′-α-bc-DNA scaffold seems to represent an interesting combination between flexibility and rigidity.

This modification also shares the interesting properties of classical α-DNA, namely high biostability and increased affinity to complementary RNA. The selectivity for RNA seems to be a conserved characteristic within the α-oligonucleotide family. Besides the 7′,5′-α-bc-DNA and α-DNA, this characteristic has been observed for α-homo-DNA (39), α-LNA (28,40), α-bc-DNA (41,42) and α-tc-DNA (43). Therefore, epimerization at the C1′ position represents a potentially efficient strategy to increase the drug-like properties of oligonucleotides.

Currently, the design of negatively charged antisense oligonucleotides primarily relies on the sulfurization of the internucleosidic linkage to improve biostability (44) and to enhance protein binding, thus assuring sufficient blood retention and efficient cellular uptake (45). However, this strategy comes at the expense of increased systemic (46) and cellular (47) toxicity. Alternative and safer strategies can be identified through the exhaustive investigation of novel base-pairing systems. Here, we showed that our 7′,5′-α-bc-DNA modified scaffold could confer sufficient biostability for *in vivo* applications, even with a phosphodiester linkage. In addition, we showed that the *in vivo* delivery of PO-7′,5′-α-bc-DNA oligonucleotides can be efficiently driven by conjugation to a targeting ligand, resulting in exon splicing modulation activity similar to state-of-the-art chemistry. In overall, our primary biological investigation reveals that the 7′,5′-α-bc-DNA scaffold is a promising option to develop efficient therapeutic drugs with a natural PO-backbone and thus to overcome the dose limiting toxicity associated with PS linkage. However, efficient delivery to muscular tissues after systemic delivery still needs to be developed.

## DATA AVAILABILITY

The X-ray crystallographic coordinate for structure reported in this study have been deposited at the Cambridge Crystallographic Data Centre (CCDC) under deposition numbers 2045356 (**20**) and 2045359 (**27**). The coordinate for last frames of the 60 ns simulation reported in this study have been deposited at ModelArchive under ID ma-o98f7 (7′,5′-α-bc-DNA homo-duplex) and ma-ijfsc (7′,5′-α-bc-DNA/RNA duplex).

## Supplementary Material

gkab1097_Supplemental_File
