# Supplementary material for: 7′,5′-alpha-bicyclo-DNA: new chemistry for oligonucleotide exon splicing modulation therapy
Source: Nucleic Acids Res. 2021 Nov 26;49(21):12089–105. doi: 10.1093/nar/gkab1097 (PMC8643641; doi:10.1093/nar/gkab1097)
Supplement: gkab1097_Supplemental_File [file gkab1097_supplemental_file.pdf]

Table of Contents

1. Supplementary Tables 2

1.1. Characterizations of oligonucleotides 2

1.2. Mismatch discrimination 2

2. Supplementary figures 3

2.1. Biostability 3

2.2 UV-melting curves of ON6 with fully modified parallel ON7 5

3. Synthesis 5

3.1. Synthesis of phosphoramidite building blocks 6

3.2. Synthesis of 5’-O-p-nitrobenzoyl-7’,5’-α-bc-T 52

4. NOESY spectra 57

5. HMBC spectra 58

6. Anomer separations 59

7. Crystal-structure determination 63

8. References 80

1. Supplementary Tables

1.1. Characterizations of oligonucleotides

Table S1: Characterizations of oligonucleotides by HPLC and mass spectroscopy (LC-MS or ESI- mass spectrometry)

Entry	Sequence[a]	HPLC Gradient[b]	Retention time [min]	Purity [%]	Experimental mass
**ON1**	5′-d(GGA TGT TC**t** CGA)-3′	B: 15 → 55%	12.44	95.8	3701.64
**ON2**	5′-d(GGA **t**GT TCT CGA)-3′	B: 15 → 55%	12.33	99.8	3701.64
**ON3**	5′-d(GGA **t**GT TC**t** CGA)-3′	B: 15 → 55%	12.25	95.4	3727.66
**ON4**	5′-d(GGA TG**t** **t**CT CGA)-3′	B: 15 → 55%	12.25	97.8	3727.66
**ON5**	5′-d(GCA **ttt** **tt**A CCG)-3′	B: 15 → 55%	12.40	97.1	3739.65
**ON6**	5′-d(**agc tct tgt agg**)-7′	B: 30 → 50%	11.01	93.5	4015.86
**ON7**	5′-d(**cct aca aga gct**)-7′	B: 18 → 32%	17.27	98.7	3981.90
**ON8**	5′-d(**tcg aga aca tcc**)-7′	B: 22 → 30%	12.38	96.1	3981.90
**ON9**[c]	5′-d(**t*****c*****c*****a*****t*****t*****c*****g*****g*****c*****t*****c*****c*****a*****a**)-7′	B: 25 → 58%	15.67	99.0	5186.81
**ON10**[d]	5’-d(**tccattcggctccaa**)-7’*P	B: 5 → 40%	17.62	91.9	5397.1
**ON11**[e]	5’-d(**tccattcggctccaa**)-7’			96.9	4953.1
**ON12**[e]	5’-d(t*c*c*a*t*t*c*g*g*c*t*c*c*a*a)-7’*P			92.3	5613.1
**ON13**[e]	5'-d(**ggtcgtaatactttcact**)7’-GalNAc			82.4	7603.9

[a] A, G, T, C denote natural 2′-deoxynucleosides; **a**, **g**, **t**, **c** corresponds to modified adenine, guanine, thymine and methylcytosine respectively, * denotes a phosphorothioate linkage, P corresponds to palmitic acid, GalNac corresponds to a N-acetylgalactosamine conjugate.

[b] Mobile phase “A”: 25 mM Trizma in H_2O, pH 8.0; Mobile phase “B”: 25 mM Trizma, 1.25 M NaCl in H_2O, pH 8.0; Analyses were performed by using a linear gradient of B in A, over 20 min, with a flow of 1 ml/min.

[c] Mobile phase “A”: 10 mM NaOH in H_2O, pH 12.0; Mobile phase “B”: 10 mM NaOH, 2.50 M NaCl in H_2O, pH 12.0.

[d] Mobile phase “A”: 50 mM TEAA in H_2O, pH 8.0; Mobile phase “B”: 50 mM TEAA, 2.0 M NaCl in H_2O, pH 8.0, 10% MeCN

[e] LC-analyses performed by third-party

1.2. Mismatch discrimination

Table S2: T_m values from UV-melting curves (260 nm) of **ON6** and **DNA1** in duplex with complementary DNA incorporating one mismatch.

Entry	Duplex[a]	X=A	X=T	X=G	X=C
**ON6**	5’-d(**agc tct tgt agg**)-7’	43.2	30.0	33.5	28.9
**DNA-X**	5´-d(TCG XGA ACA TCC)-3'				
**DNA**	5’-d(GGA TGT TCT CGA)-3’	49.1	38.3	40.5	36.7
**DNA-X**	5´-d(TCG XGA ACA TCC)-3'				

Experimental conditions: total strand conc. 2 µM in 10 mM NaH_2PO_4, 150 mM NaCl, pH 7.0

[a] A, G, T, C denote natural 2‘-deoxynucleosides; **a**, **g**, **t**, **c** corresponds to modified adenine, guanine, thymine and methylcytosine respectively.

2. Supplementary figures

2.1. Biostability

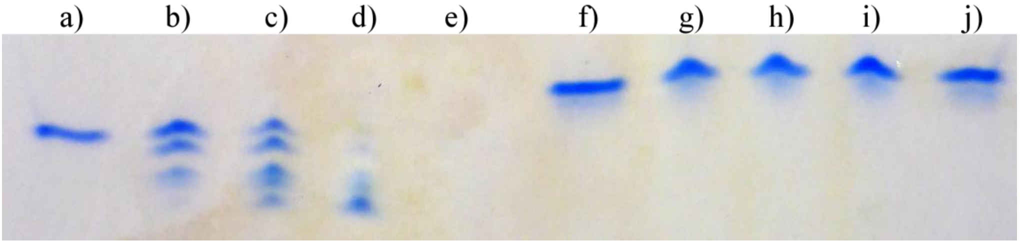

Figure S1: Biostability in serum. Cropped picture of the gel. a) **DNA** control experiment; **DNA** digestion reaction after b) 1 hour c) 2 hours d) 4 hours e) 24 hours; f) **ON6** control experiment; **ON6** digestion reaction after g) 1 hour h) 2 hours i) 4 hours j) 24 hours

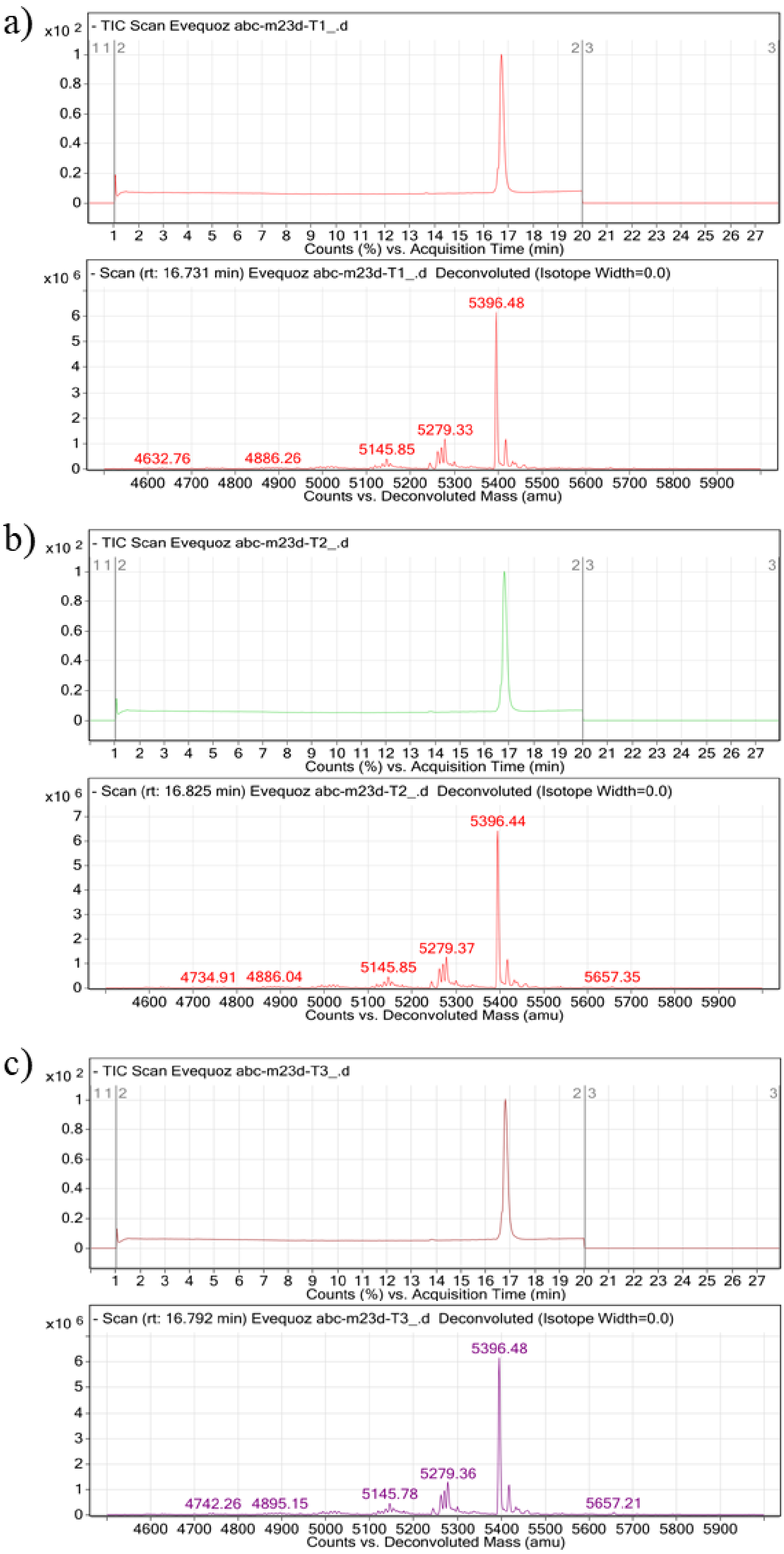

Figure S2: Biostability in acidic conditions: Characterization by LC-MS of **ON10** after incubation in acidic condition; a) chromatogram and deconvoluted mass spectrum at t=0h; b) chromatogram and deconvoluted mass spectrum at t=2h; c) chromatogram and deconvoluted mass spectrum at t=24h.

2.2 UV-melting curves of ON6 with fully modified parallel ON7

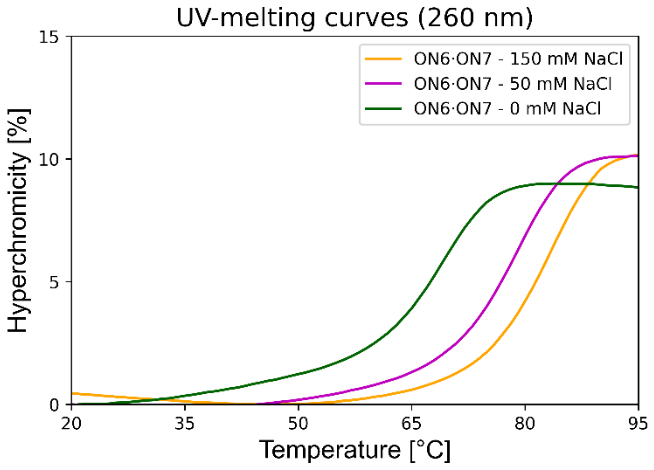

Figure S3: UV-melting curves (260 nm) of **ON6** with fully modified parallel **ON7**. total strand conc. 2µM in 10 mM NaH2PO4, 150, 50 or 0 mM NaCl, pH 7.0

3. Synthesis

General procedures

All reactions were performed in dried glassware and under an inert atmosphere of argon. Anhydrous solvents for reactions were obtained by filtration through activated alumina or by storage over molecular sieves (4 Å). Colum chromatography (CC) was performed on silica gel (SiliaFlash P60, 40-63 um, 60 Å). Methanol used for CC was of HPLC grade, all other solvents used for CC were of technical grade and distilled prior to use. Thin-layer chromatography was performed on silica gel plates (Macherey-Nagel, pre-coated TLC-plates sil G-25 UV254). Compounds were visualized under UV light or by dipping in a *p*-anisaldehyde staining solution [*p*-anisaldehyde (3.7 mL), glacial acetic acid (3.7 mL), concentrated sulfuric acid (5 mL), ethanol (135 mL)] followed by heating with a heat gun. NMR spectra were recorded at 300 or 400 MHz (^{1}H), at 75 or 101 MHz (^{13}C) and at 122 MHz (^{31}P) in either $CDCl_3$, CD_3OD, $(CD_3)_2SO$ or CD_3CN. Chemical shifts (δ) are reported relative to the undeuterated residual solvent peak [$CDCl_3$: 7.26 ppm (^{1}H), 77.16 ppm (^{13}C); CD_3OD: 3.31 ppm (^{1}H); $(CD_3)_2SO$: 2.50 ppm (^{1}H), 39.52 ppm (^{13}C); CD_3CN: 1.94 ppm (^{1}H)]. Signal assignments are based APT and DEPT and on ^{1}H,^{1}H and ^{1}H,^{13}C correlation experiments (COSY, HSQC, HMBC). High resolution mass detections were performed by electrospray ionization in the positive mode (ion trap, ESI^{+}).

3.1. Synthesis of phosphoramidite building blocks

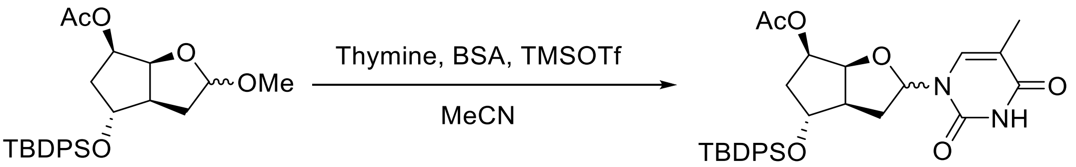

***(3'R,5'R,7'R)-1-{5'-O-acetyl-7'-[(tert-butyldiphenylsilyl)oxy]-2',3'-dideoxy-3',5'-ethano-α,β-D-ribofuranosyl} thymine** (2)* :

To a solution of the sugar **1** (933 mg, 2.05 mmol) and thymine (372 mg, 3.08 mmol) in dry MeCN (12 mL) was added dropwise BSA (1.5 mL, 6.15 mmol) at rt. After stirring for 50 min at rt, the solution was cooled down to 0°C and TMSOTf (0.45 mL, 2.5 mmol) was added dropwise. After further stirring for 3 h at 0° C and for 15 h at rt, the reaction mixture was diluted with satd $NaHCO_3$ (100 mL) and extracted with DCM (4 X 40 mL). The combined organic phases were dried over $MgSO_4$, filtered and evaporated. The crude product was purified by CC (2.5% isopropanol in DCM) to yield a mixture of **2** (924 mg, 82%) in an anomeric ratio α/β ≈ 85:15 as a white foam.

Data for **2**: R_f = 0.56 (7% MeOH in DCM);

^{1}H NMR (300 MHz, $CDCl_3$) δ 9.14 (*br*, 1H, H-N(3)), 7.53 (dd, *J* = 7.7, 1.6 Hz, 4H, H-arom), 7.39 – 7.23 (m, 6H, H-arom), 7.09 (d, *J* = 1.0 Hz, 0.15H, H-C(6)), 6.87 (d, *J* = 1.0 Hz, 0.85H, H-C(6)), 5.83 (t, *J* = 6.2 Hz, 0.85H, H-C(1')), 5.80 – 5.70 (m, 0.15H, H-C(1')), 5.36 – 5.04 (m, 1H, H-C(5')), 4.89 (dd, *J* = 6.3, 5.2 Hz, 0.85H, H-C(4')), 4.62 (dd, *J* = 7.1, 5.6 Hz, 0.15H, H-C(4')), 4.01 – 3.85 (m, 1H, H-C(7')), 2.76 – 2.55 (m, 1H, H-C(3')), 2.09 – 1.91 (m, 4H, H-C(6'), *Me*CO_2), 1.90 – 1.58 (m, 6H, H-C(6'), H-C(2'), *Me*-C(5)), 0.96 (s, 9H, $(CH_3)_3$-C-Si).

^{13}C NMR (75 MHz, $CDCl_3$) δ 170.70 (Me*C*O_2), 163.87 (C(4)), 150.29 (C(2)), 135.69, 135.67 (CH-arom), 134.99 (C(6)), 133.58, 133.18 (C-arom), 130.03, 127.87 (CH-arom), 111.05 (C(5)), 87.56 (C(1')), 82.85 (C(4')), 76.50 (C(7')), 74.76 (C(5')), 50.72 (C(3')), 37.79 (C(6')), 36.94 (C(2')), 26.88 ((CH_3)$_3$-C-Si), 20.95 (*Me*CO_2), 19.01 ($(CH_3)_3$-*C*-Si), 12.63 (*Me*-C(5)).

ESI$^+$-HRMS *m/z* calcd for $C_{30}H_{37}O_6N_2Si$ ($[M + H]^+$) 549.2415, found 549.2401.

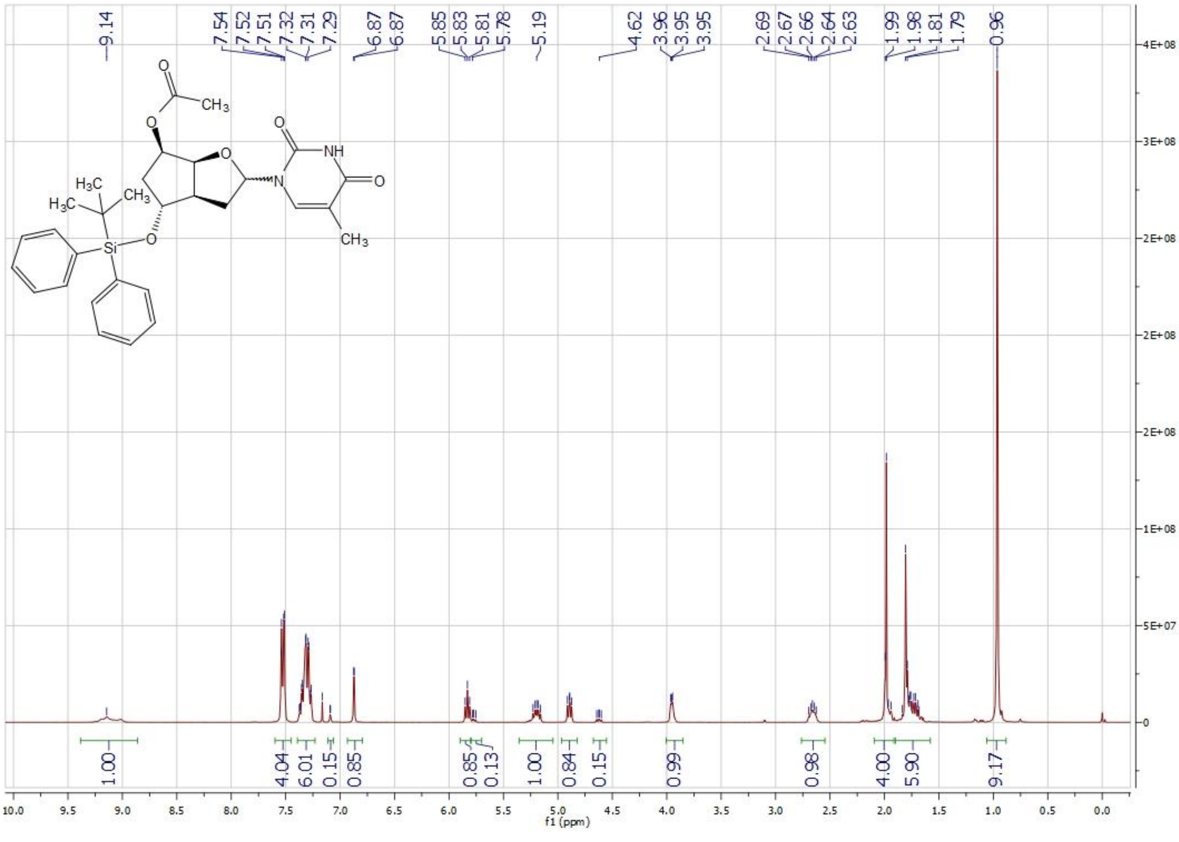
9.14
7.54 7.52 7.51 7.32 7.31 7.29
6.87 6.87
5.85 5.83 5.81 5.78
5.19
4.62 3.96 3.95 3.95
2.69 2.67 2.66 2.64 2.63
1.99 1.98 1.81 1.79
0.96
CH_3
O
NH
N
H_3C
CH_3
Si
1.00 4.04 6.01 0.15 0.85 0.85 0.13 1.00 0.84 0.15 0.99 0.98 4.00 5.90 9.17
f1 (ppm)

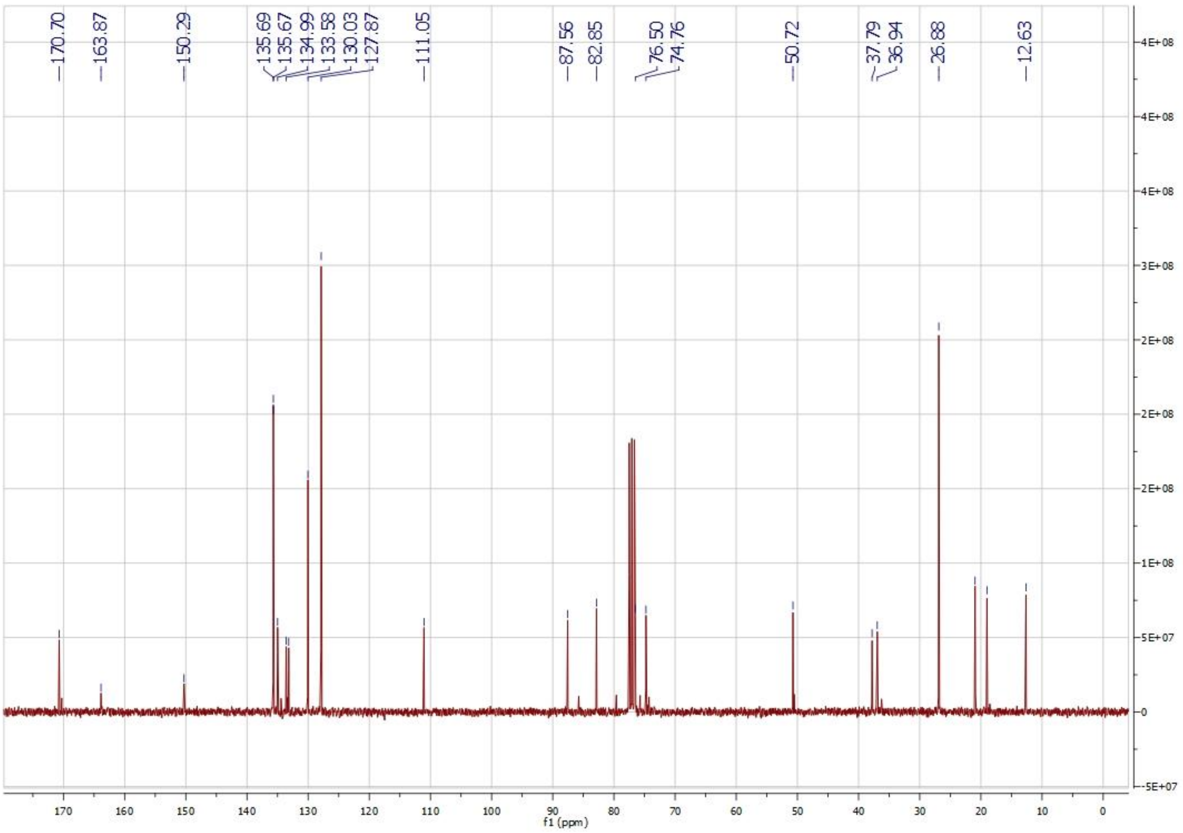
170.70
163.87
150.29
135.69 135.67 134.99 133.58 130.03 127.87
111.05
87.56
82.85
76.50 74.76
50.72
37.79 36.94
26.88
12.63
f1 (ppm)

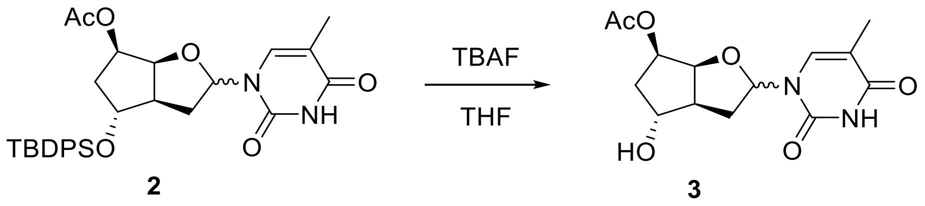

(3'S,5'R,7'R)-1-{5'-O-Acetyl-2',3'-dideoxy-3',5'-ethano-7'-hydroxy-α,β-D-ribofuranosyl} thymine (3) :

To a solution of the nucleoside **2** (924 mg, 1.68 mmol) in dry THF (10 mL) was added TBAF (1M in THF, 3.4 mL, 3.4 mmol) at rt. After stirring for 2 h at rt, the reaction mixture was diluted with satd $NaHCO_3$ (80 mL) and extracted with EtOAc (3 X 80 mL) and DCM (2 X 80 mL). The combined organic phases were dried over $MgSO_4$, filtered and evaporated. The crude product was purified by CC (5% MeOH in DCM) to yield an anomeric mixture of **3** (391 mg, 75%).

Data for **3**: R_f = 0.24 (7% MeOH in DCM);

^{1}H NMR (400 MHz, $CDCl_3$) δ 9.66 (*br*, 0.15H, H-N(3)), 9.63 (*br*, 0.85H, H-N(3)), 7.27 (d, *J* = 1.0 Hz, 0.15H, H-C(6)), 7.06 (d, *J* = 1.0 Hz, 0.85H, H-C(6)), 6.00 (t, *J* = 6.1 Hz, 0.85H, H-C(1')), 5.91 (dd, *J* = 8.8, 5.5 Hz, 0.15H, H-C(1')), 5.26 – 5.10 (m, 1H, H-C(5')), 4.92 (dd, *J* = 6.5, 5.3 Hz, 0.85H, H-C(4')), 4.65 (dd, *J* = 6.9, 5.7 Hz, 0.15H, H-C(4')), 4.19 – 4.03 (m, 1H, H-C(7')), 2.91 – 2.72 (m, 2H, H-C(3'), O*H*), 2.64 (ddd, *J* = 13.3, 9.8, 5.5 Hz, 0.15H, H-C(2')), 2.25 – 2.15 (m, 1.7H, H-C(2')), 2.05 (s, 0.45H, *Me*CO_2), 2.04 (s, 2.55H, *Me*CO_2), 2.03 – 1.89 (m, 2H, H-C(6')), 1.88 (d, *J* = 0.7 Hz, 0.45H, *Me*-C(5)), 1.85 (d, *J* = 0.6 Hz, 2.55H, *Me*-C(5)), 1.42 – 1.28 (m, 0.15H, H-C(2')).

^{13}C NMR (101 MHz, $CDCl_3$) δ 170.87 (Me*C*O_2), 164.26 (C(4)), 150.66 (C(2)), 135.54 (C(6)), 111.22 (C(5)), 87.97 (C(1')), 82.97 (C(4')), 75.08 (C(7')), 74.52 (C(5')), 50.07 (C(3')), 37.81 (C(2')), 37.23 (C(6')), 21.02 (*Me*CO_2), 12.67 (*Me*-C(5)).

ESI$^+$-HRMS *m/z* calcd for $C_{14}H_{19}O_6N_2$ ([M + H]$^+$) 311.1238, found 311.1234.

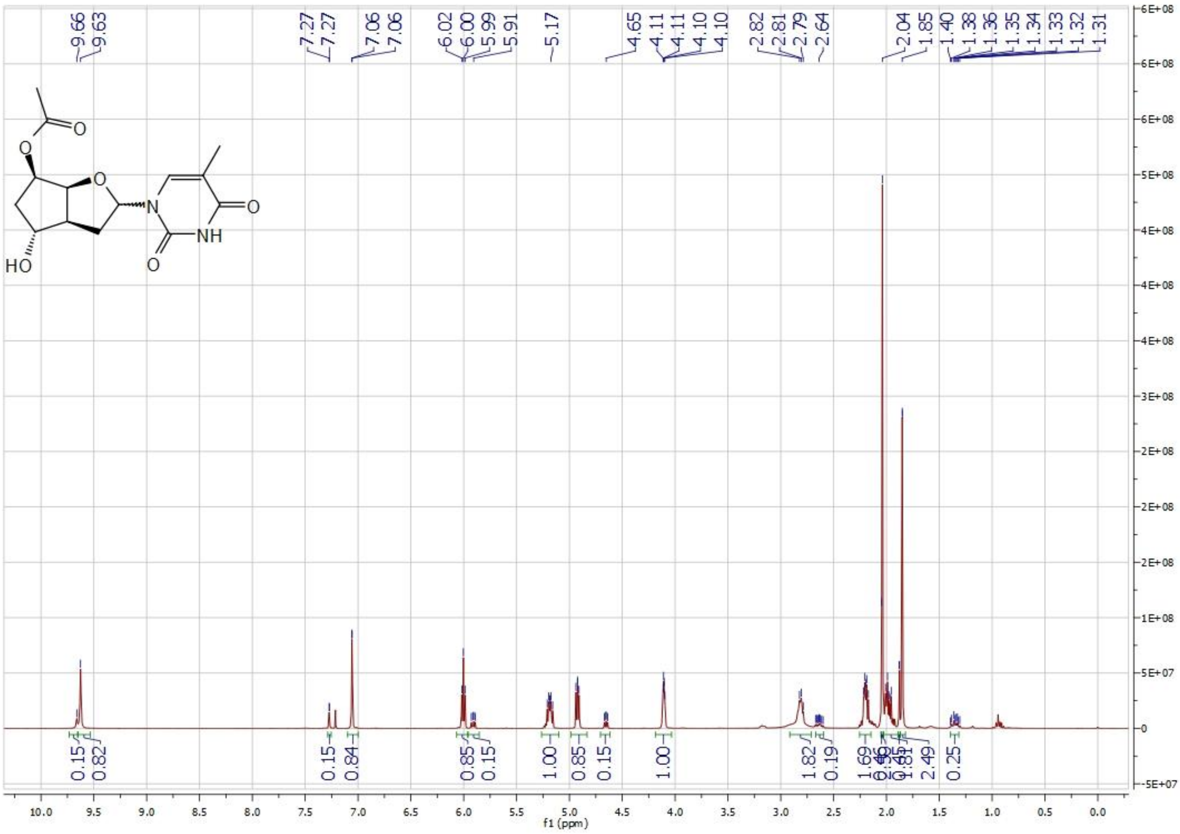
9.66 9.63
7.27 7.27 7.06 7.06
6.02 6.00 5.99 5.91
5.17
4.65 4.11 4.11 4.10 4.10
2.82 2.81 2.79 2.64
2.04 1.85 1.40 1.38 1.36 1.35 1.34 1.33 1.32 1.31
HO
O
NH
N
0.15 0.82 0.15 0.84 0.85 0.15 1.00 0.85 0.15 1.00 1.82 0.19 1.69 2.49 0.25
f1 (ppm)

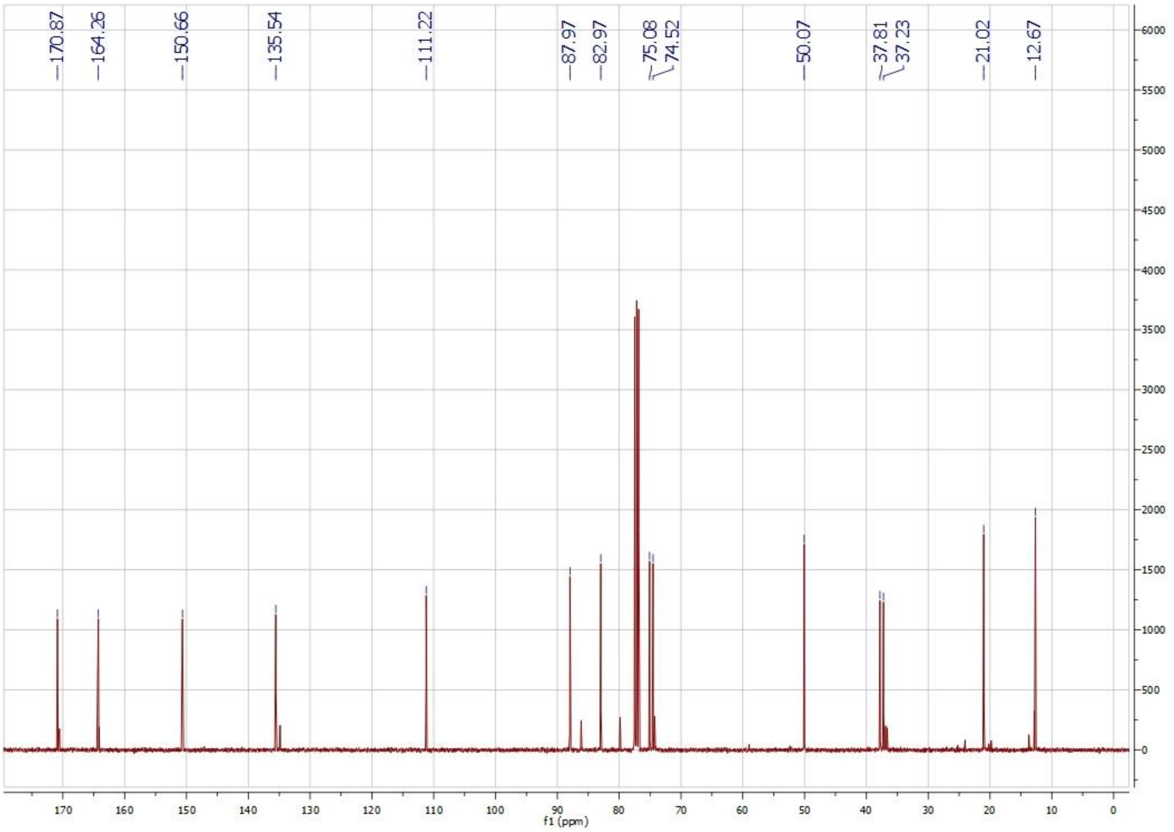
170.87 164.26 150.66 135.54 111.22 87.97 82.97 75.08 74.52 50.07 37.81 37.23 21.02 12.67
f1 (ppm)

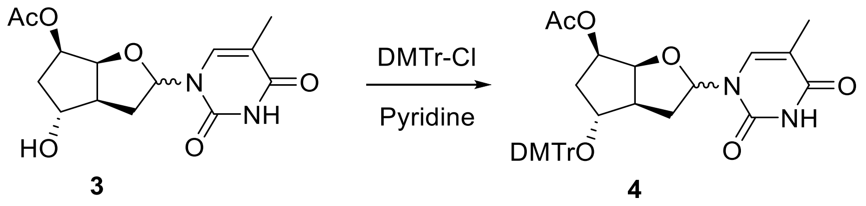

(3'S,5'R,7'R)-1-{5'-O-Acetyl-2',3'-dideoxy-3',5'-ethano-7'-O-[(4,4'-dimethoxytriphenyl)methyl]-α,β-D-ribofuranosyl} thymine (4) :

To a solution of the nucleoside **3** (364 mg, 1.17 mmol) in dry pyridine (7 mL) was added DMTr-Cl (1.19 g, 3.51 mmol) at rt. The solution was stirred for 1 day and then was diluted with satd $NaHCO_3$ (50 mL) and extracted with DCM (3 X 50 mL). The combined organic phases were dried over $MgSO_4$, filtered and evaporated. The crude product was purified by CC (EtOAc/hexane 2:1, +0.5 % Et_3N) to yield an anomeric mixture of **4** (690 mg, 96%) as a yellow foam.

Data for **4**: R_f = 0.70 (8% MeOH in DCM);

^{1}H NMR (300 MHz, $CDCl_3$) δ 9.17 (*br*, 0.85H, H-N(3)), 8.56 (*br*, 0.15H, H-N(3)), 7.38 – 7.32 (m, 2H, H-arom), 7.29 – 7.15 (m, 7H, H-arom), 6.82 (d, *J* = 1.1 Hz, 1H, H-C(6)), 6.76 (d, *J* = 8.9 Hz, 4H, H-arom), 5.86 (t, *J* = 6.0 Hz, 0.85H, H-C(1')), 5.71 (dd, *J* = 8.9, 5.4 Hz, 0.15H, H-C(1')), 5.25 (dd, *J* = 10.2, 5.6 Hz, 0.15H, H-C(5')), 5.21 – 5.11 (m, 0.85H, H-(C5')), 4.78 (dd, *J* = 6.7, 4.8 Hz, 0.85H, H-C(4')), 4.49 (dd, *J* = 7.1, 5.3 Hz, 0.15H, H-C(4')), 3.84 (*br*, 1H, H-C(7')), 3,72, 3.71 (2s, 6H, *Me*O), 2.34 – 2.23 (m, 1H, H-C(3')), 2.01, 1.99 (2s, 3H, *Me*CO_2), 1.82 (d, *J* = 0.5 Hz, 3H, *Me*-C(5)), 1.80 – 1.56 (m, 4H, H-C(2'), H-C(6')).

^{13}C NMR (75 MHz, $CDCl_3$) δ 170.69 (Me*C*O_2), 163.91 (C(4)), 158.82 (MeO-*C*-arom), 150.33 (C(2)), 145.34, 136.64, 136.58 (C-arom), 135.00 (C(6)), 130.25, 128.39, 128.07, 127.15, 113.41 (CH-arom), 111.04 (C(5)), 87.70 (*C*$(Ph)_3$), 87.31 (C(1')), 83.15 (C(4')), 77.16 (C(7')), 74.96 (C(5')), 55.37 (*Me*O-DMTr), 49.12 (C(3')), 37.55 (C(2')), 36.82 (C(6')), 21.07 (*Me*CO_2), 12.66 (*Me*-C(5)).

ESI^+-HRMS *m/z* calcd for $C_{35}H_{36}O_8N_2$ ($[M + H]^+$) 612.2466, found 612.2453.

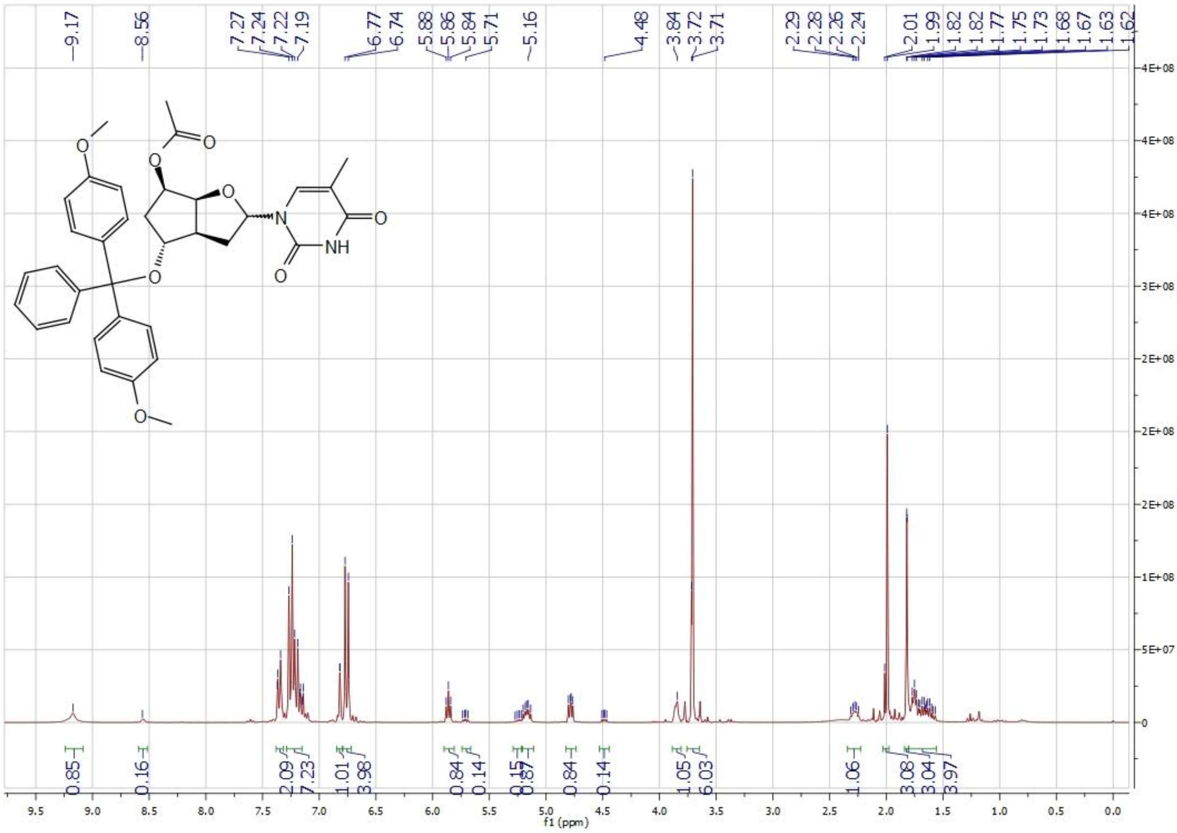
9.17
8.56
7.27 7.24 7.22 7.19
6.77 6.74
5.88 5.86 5.84 5.71
5.16
4.48
3.84 3.72 3.71
2.29 2.28 2.26 2.24
2.01 1.99 1.82 1.82 1.77 1.75 1.73 1.68 1.67 1.63 1.62
O
O
O
N
NH
O
O
O
O
4E+08
3E+08
2E+08
1E+08
5E+07
0
0.85 0.16 2.09 7.23 1.01 3.98 0.84 0.14 0.15 0.87 0.84 0.14 1.05 6.03 1.06 3.08 3.04 3.97
9.5 9.0 8.5 8.0 7.5 7.0 6.5 6.0 5.5 5.0 4.5 4.0 3.5 3.0 2.5 2.0 1.5 1.0 0.5 0.0
f1 (ppm)

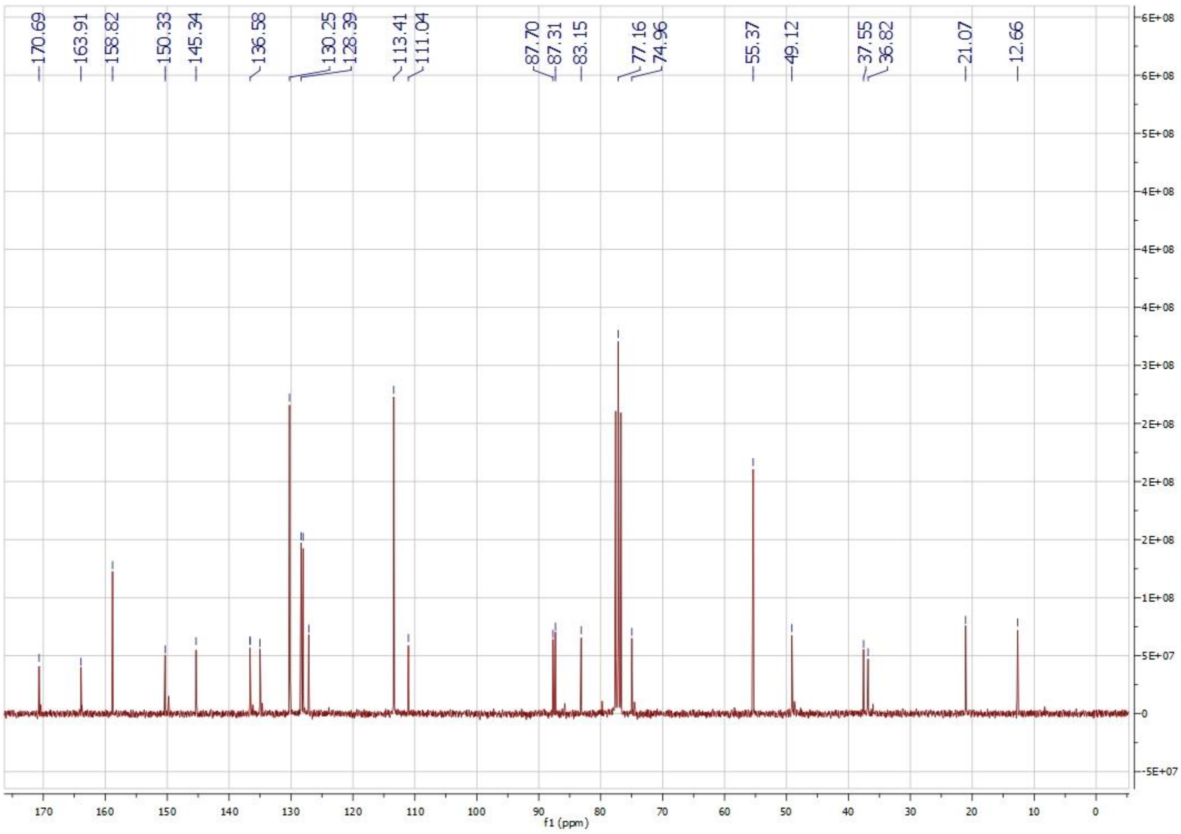
170.69
163.91
158.82
150.33
145.34
136.58
130.25 128.39
113.41 111.04
87.70 87.31
83.15
77.16 74.96
55.37
49.12
37.55 36.82
21.07
12.66
6E+08
5E+08
4E+08
3E+08
2E+08
1E+08
5E+07
0
-5E+07
170 160 150 140 130 120 110 100 90 80 70 60 50 40 30 20 10 0
f1 (ppm)

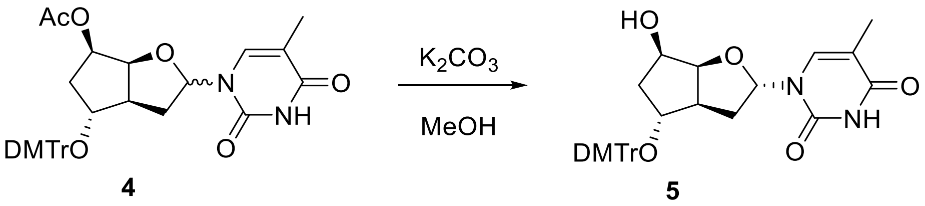

(3'S,5'R,7'R)-1-{2',3'-Dideoxy-3',5'-ethano-7'-O-[(4,4'-dimethoxytriphenyl)methyl]-α-D-ribofuranosyl} thymine (5) :

To a solution of the nucleoside **4** (690 mg, 1.12 mmol) in dry MeOH (10 mL) was added K_2CO_3 (467 mg, 3.36 mmol) at rt. The solution was stirred for 3 h and then diluted with satd NaCl (60 mL) and extracted with DCM (3 X 60 mL). The combined organic phases were dried over $MgSO_4$, filtered and evaporated. The crude product was purified by CC (3% isopropanol in Et_2O, +0.5 % Et_3N) to yield the α-anomer **5** (550 mg, 86%) as a white solid.

Data for **5**: R_f = 0.39 (5% MeOH in DCM);

^{1}H NMR (400 MHz, $CDCl_3$) δ 9.37 (*br*, 1H, H-N(3)), 7.39 – 7.31 (m, 2H, H-arom), 7.25 (d, *J* = 8.3 Hz, 4H, H-arom), 7.20 (t, *J* = 7.7 Hz, 2H, H-arom), 7.16 – 7.08 (m, 1H, H-arom), 6.78 (d, *J* = 1.1 Hz, 1H, H-C(6)), 6.74 (d, *J* = 8.8 Hz, 4H, H-arom), 5.91 (dd, *J* = 6.5, 4.9 Hz, 1H, H-C(1')), 4.57 (dd, *J* = 7.2, 4.4 Hz, 1H, H-C(4')), 4.35 – 4.18 (m, 1H, H-C(5')), 3.86 (d, *J* = 4.7 Hz, 1H, H-C(7')), 3.69 (s, 6H, *Me*O), 2.53 (*br*, 1H, O*H*), 2.22 (dd, *J* = 15.3, 6.3 Hz, 1H, H-C(3')), 1.85 – 1.69 (m, 5H, *Me*-C(5), H-C(2'), H-C(6')), 1.66 – 1.49 (m, 2H, H-C(2'), H-C(6')).

^{13}C NMR (101 MHz, $CDCl_3$) δ 163.98 (C(4)), 158.67 (MeO-*C*-arom), 150.47 (C(2)), 145.48 , 136.80, 136.75 (C-arom), 134.94 (C(6)), 130.19, 130.18, 128.35, 127.97, 127.01, 113.31 (CH-arom), 111.04 (C(5)), 87.82 (*C*(Ph)$_3$), 87.05 (C(1')), 85.74 (C(4')), 78.26 (C(7')), 73.33 (C(5')), 55.31 (*Me*O-DMTr), 48.81 (C(3')), 40.21 (C(6')), 37.68 (C(2')), 12.65 (*Me*-C(5)).

ESI$^+$-HRMS *m/z* calcd for $C_{33}H_{35}O_7N_2$ ([M + H]$^+$) 571.2439, found 571.2421.

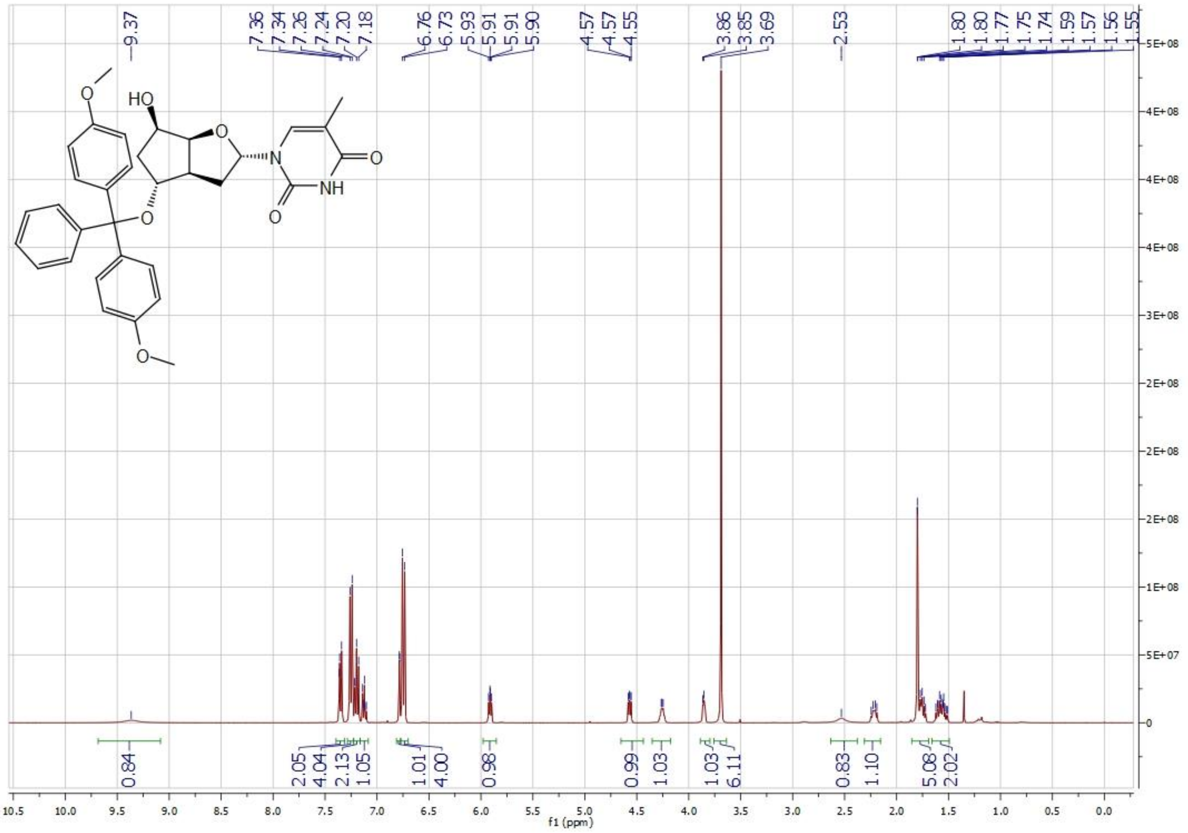

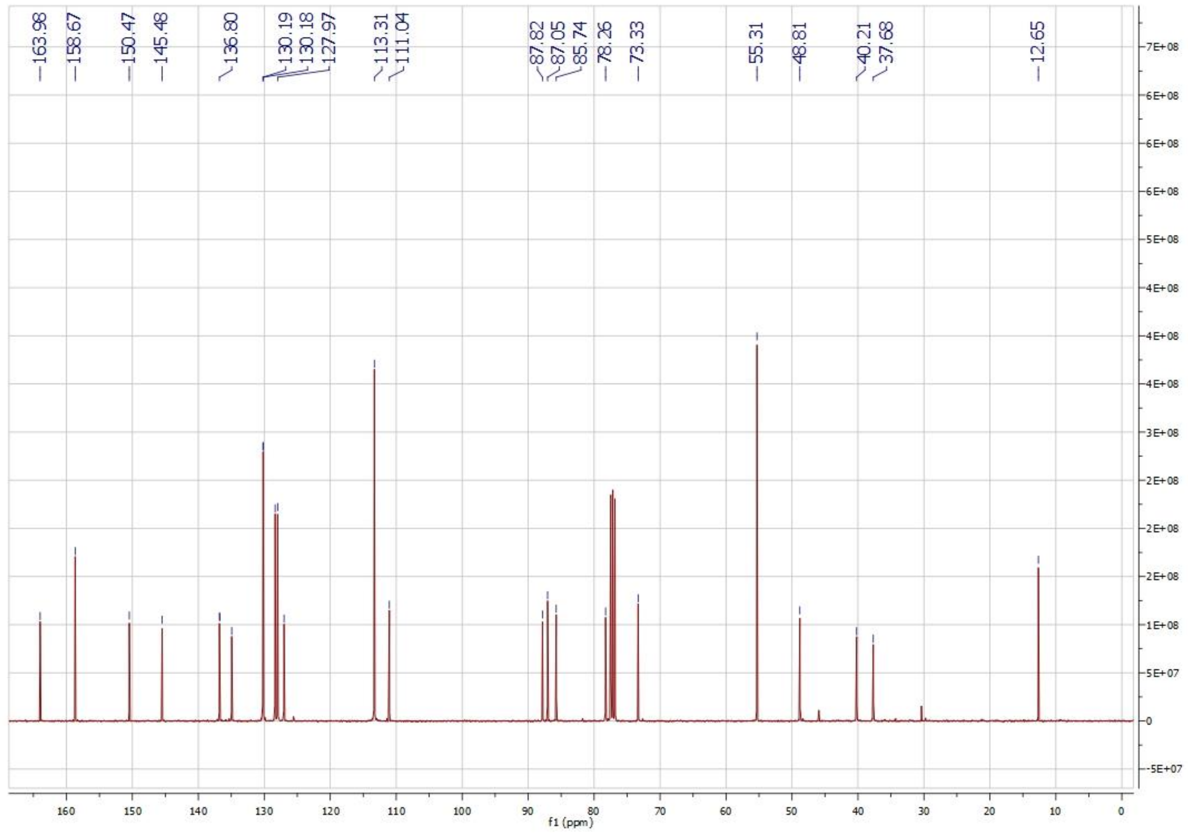

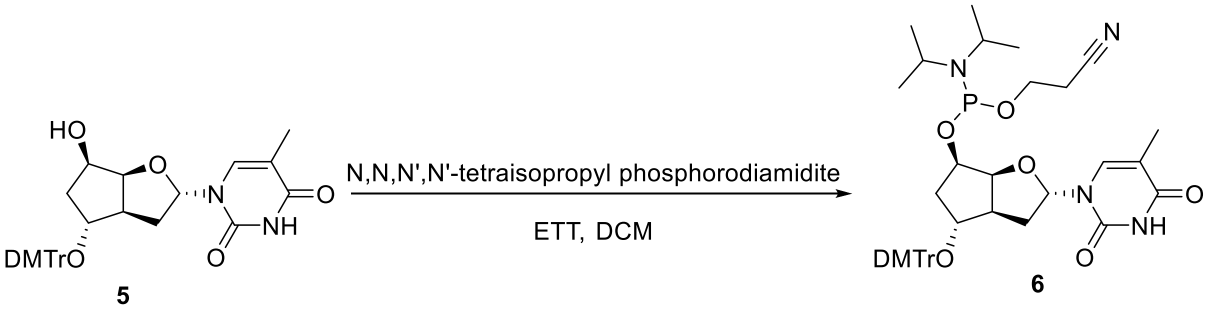

(3'S,5'R,7'R)-1-{5'-O-[(2-cyanoethoxy)-diisopropylaminophosphanyl]2',3'-Dideoxy-3',5'-ethano-7'-O-[(4,4'-dimethoxytriphenyl)methyl]-α-D-ribofuranosyl} thymine (6) :

To a solution of the nucleoside **5** (200 mg, 0.350 mmol) and 5-(Ethylthio)-1H-tetrazole (59 mg, 0.46 mmol) in dry DCM (7 mL) was added dropwise 2-Cyanoethyl N,N,N′,N′-tetraisopropylphosphordiamidite (0.17 mL, 0.53 mmol) at rt. After stirring for 1h, the reaction mixture was diluted with DCM (50 mL) and washed with satd $NaHCO_3$ (2 X 25 mL) and satd NaCl (25 mL). Aqueous phases were combined and extracted with DCM (30 mL). The combined organic phases were dried over $MgSO_4$, filtered and evaporated. The crude product was purified by CC (2% MeOH in DCM, +0.5 % Et_3N) to yield **6** (220 mg, mixture of two isomers, 81%) as a white solid.

Data for **6**: R_f = 0.44 (4% MeOH in DCM);

^{1}H NMR (300 MHz, $CDCl_3$) δ 9.03 (*br*, 1H, H-N(3)), 7.36 (d, *J* = 8.1 Hz, 2H, H-arom), 7.30 – 7.07 (m, 7H, H-arom), 6.84 (s, 1H, H-C(6)), 6.80 – 6.69 (m, 4H, H-arom), 5.95, 5.88 (2dd, *J* = 6.6, 4.8 Hz, 1H, H-C(1')), 4.70, 4.61 (2dd, *J* = 7.3, 4.3 Hz, 1H, H-C(4')), 4.41 – 4.20 (m, 1H, H-C(5')), 3.94 – 3.82 (m, 1H, H-C(7')), 3.81 – 3.62 (m, 8H, *Me*O, OC$H_2$$CH_2$CN), 3.59 – 3.40 (m, 2H, (Me_2C*H*)$_2$N), 2.61 – 2.46 (m, 2H, OCH_2CH_2CN), 2.28 (ddd, *J* = 14.1, 13.2, 7.3 Hz, 1H, H-C(3')), 1.91 – 1.73 (m, 5H, *Me*-C(5), H-C(6'), H-C(2')), 1.72 – 1.46 (m, 2H, H-C(6'), H-C(2')), 1.16 – 1.00 (m, 12H, (Me_2CH)$_2$N).

^{13}C NMR (75 MHz, $CDCl_3$) δ 164.01, 163.98 (C(4)), 158.70 (MeO-*C*-arom), 150.39, 150.17 (C(2)), 145.52, 136.84, 136.78 (C-arom), 135.44, 135.39 (C(6)), 130.21, 128.36, 128.32, 128.00, 127.03 (CH-arom), 118.02, 117.76 (O$CH_2$$CH_2$*C*N), 113.32 (CH-arom), 110.91, 110.59 (C(5)), 88.31, 88.06 (*C*(Ph)$_3$), 87.11, 87.06 (C(1')), 85.44, 85.39 ($J_{C,P}$ = 4.6, 3.1 Hz, C(4')), 78.25, 78.13 (C(7')), 74.70, 74.34 ($J_{C,P}$ = 13.5, 18.5 Hz, C(5')), 58.73, 58.47 ($J_{C,P}$ = 18.9, 20.1 Hz, (O*C*$H_2$$CH_2$CN)), 55.35, 55.32 (*Me*O-DMTr), 48.80, 48.64 (C(3')), 43.22, 43.06 ($J_{C,P}$ = 12.4, 11.0 Hz (Me_2C*H*)$_2$N), 39.68, 39.63 (C(6')), 38.06, 37.93 (C(2')), 24.81, 24.74, 24.71, 24.68, 24.65, 24.59 (6s, Me_2CH)$_2$N), 20.37, 20.35 ($J_{C,P}$ = 7.1, 6.8 Hz, OCH_2*C*H_2CN), 12.66 (*Me*-C(5)).

^{31}P NMR (122 MHz, $CDCl_3$) δ 148.18, 147.80.

ESI$^+$-HRMS *m/z* calcd for $C_{42}H_{52}O_8N_4P$ ([M + H]$^+$) 771.3517, found 771.3517.

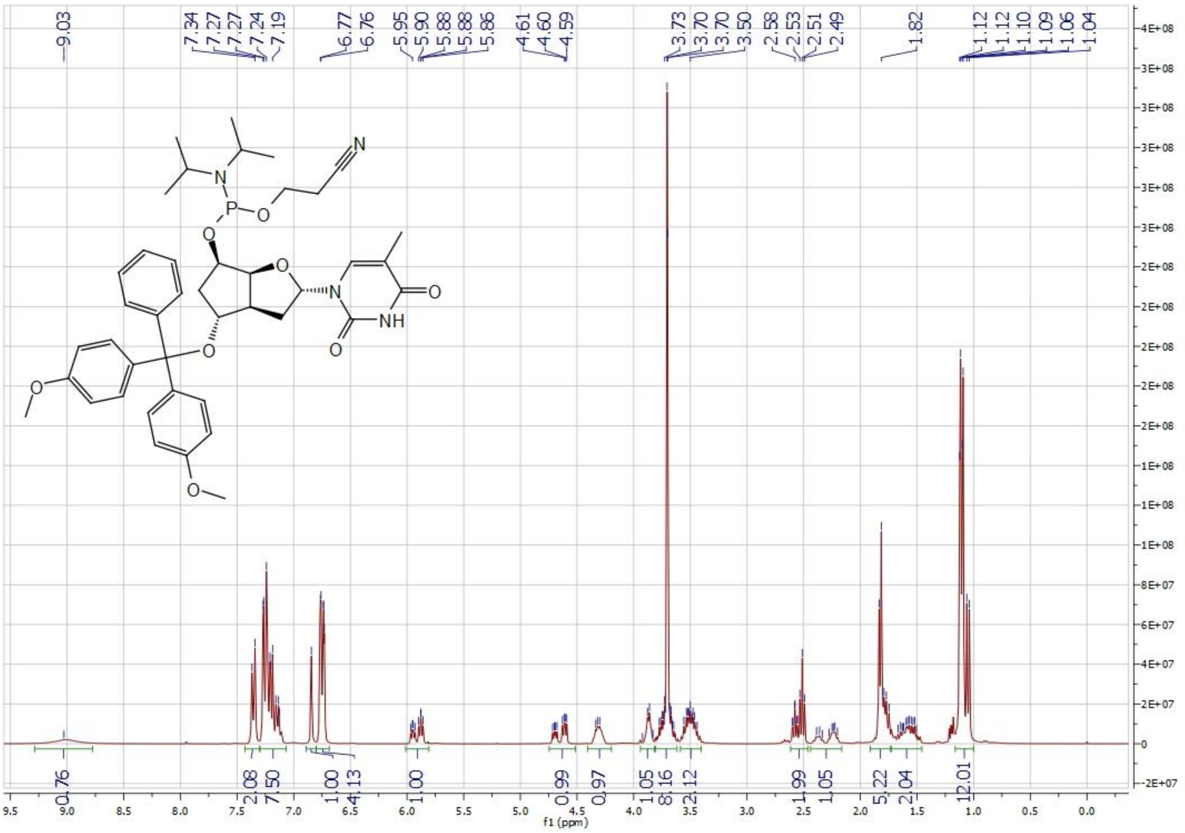
9.03
7.34 7.27 7.27 7.24 7.19
6.77 6.76
5.95 5.90 5.88 5.88 5.86
4.61 4.60 4.59
3.73 3.70 3.70 3.50
2.58 2.53 2.51 2.49
1.82
1.12 1.12 1.10 1.09 1.06 1.04
N
P
O
NH
0.76 2.08 7.50 1.00 4.13 1.00 0.99 0.97 1.05 8.16 2.12 1.99 1.05 5.22 2.04 12.01
f1 (ppm)

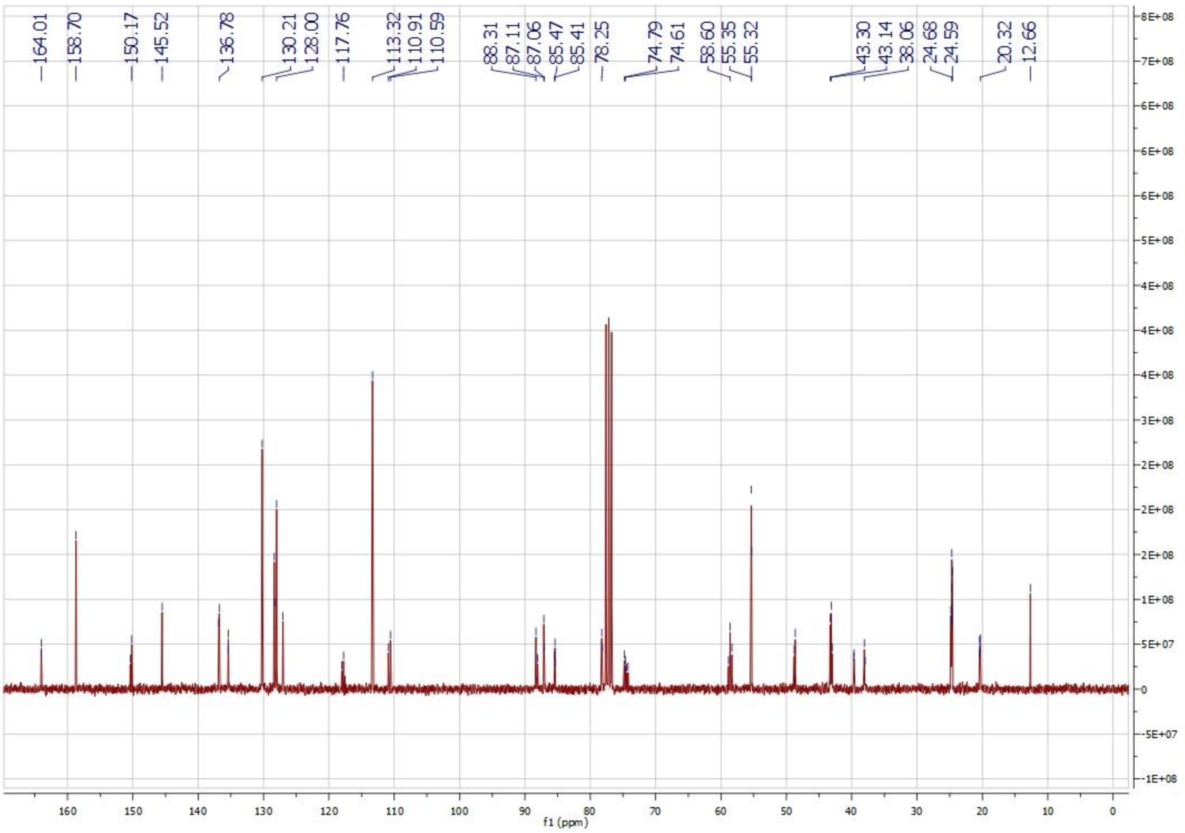
164.01 158.70 150.17 145.52 136.78 130.21 128.00 117.76 113.32 110.91 110.59
88.31 87.11 87.06 85.47 85.41 78.25 74.79 74.61 58.60 55.35 55.32
43.30 43.14 38.06 24.68 24.59 20.32 12.66
f1 (ppm)

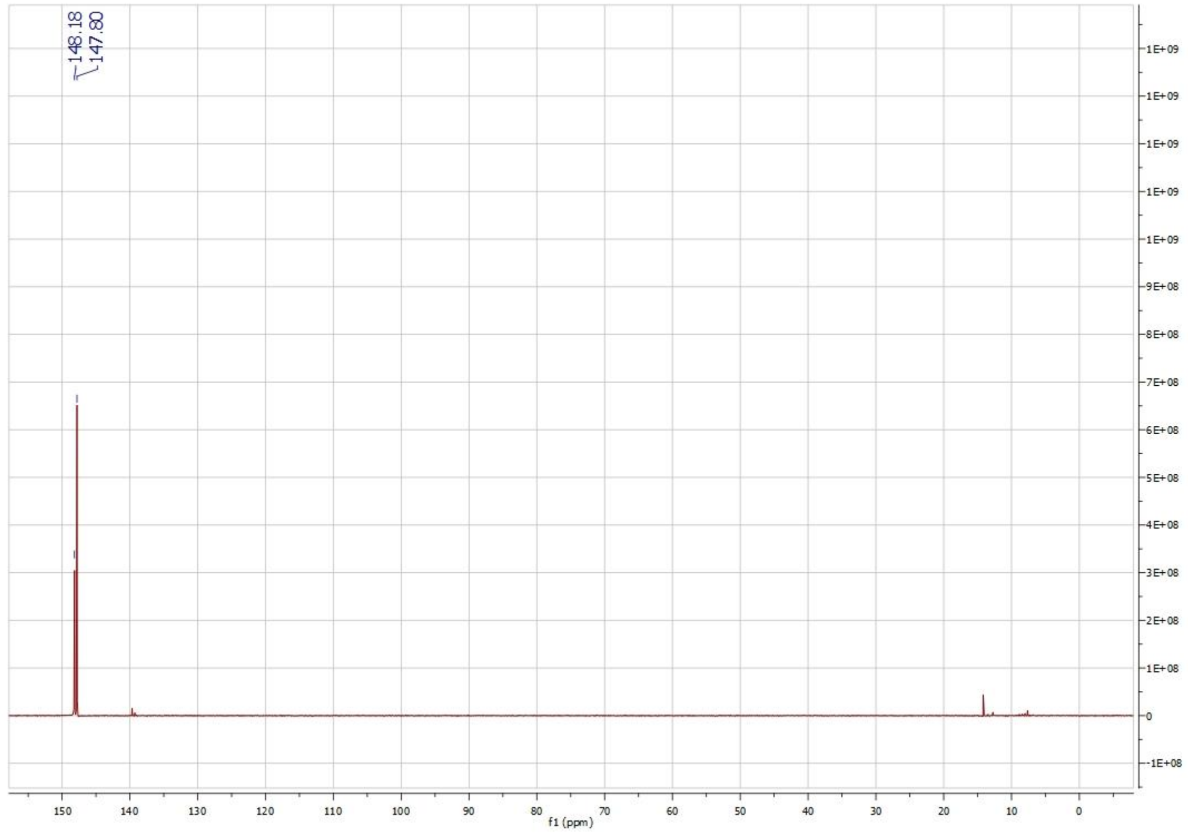

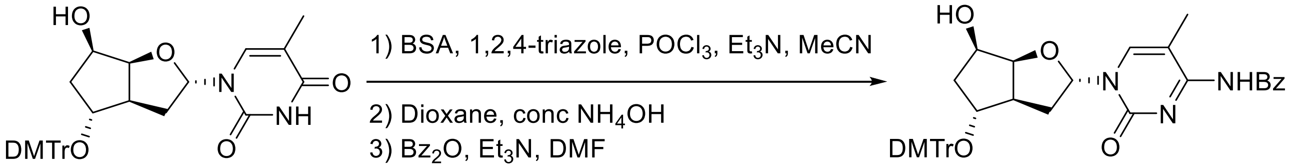

5 **7**

(3'S,5'R,7'R)-N4-Benzoyl-1-{2',3'-dideoxy-3',5'-ethano-7'-O-[(4,4'-dimethoxytriphenyl)methyl]-α-D-ribofuranosyl}-5-methylcytosine *(7)* :

To a solution of the nucleoside **5** (268 mg, 0.470 mmol) in dry MeCN (5 mL) was added dropwise BSA (0.28 mL, 1.13 mmol) at 0°, and then the solution was stirred overnight at rt. In another flask, a suspension of 1,2,4-triazole (1.14 g, 16.5 mmol) in dry MeCN (50 mL) was cool down to 0° C and $POCl_3$ (0.35 mL, 3.8 mmol) followed Et_3N (2.62 mL, 18.8 mmol) were added. The suspension was stirred for 30 min at 0° C, and then the previous prepared solution of the silylated compound **5** was added to the suspension and the mixture was further stirred for 7 h at rt. Reaction was quenched with addition satd $NaHCO_3$ (10 mL), MeCN removed under reduced pressure and the resulting mixture diluted with satd $NaHCO_3$ (30 mL) and extracted with DCM (3 X 30 mL). The combined organic phases were dried over $MgSO_4$, filtered and evaporated.

The crude product was then dissolved in a mixture of 1,4-dioxane (10 mL) and concd NH_4OH (10 mL). After stirring for 3 h at rt, the mixture was reduced to half of its volume in vacuo, diluted with satd $NaHCO_3$ (25 mL) and extracted with DCM (4 X 30 mL). The combined organic phases were dried over $MgSO_4$, filtered and evaporated.

The crude product was then dissolved in dry DMF (10 mL). Et_3N (80 µL, 0.56 mmol) followed by Bz_2O (266 mg, 1.18 mmol) were added at rt and the solution was stirred overnight. The resulting brownish solution was quenched by careful addition of satd $NaHCO_3$ (40 mL) and extracted with DCM (4 X 40 mL). The combined organic phases were dried over $MgSO_4$, filtered and evaporated. The crude product was purified by CC (EtOAc/hexane 1:1, +0.5 % Et_3N) to yield **7** (263 mg, 83%) as a white foam.

Data for **7**: R_f = 0.53 (EtOAc/hexane 3:1);

^{1}H NMR (300 MHz, $CDCl_3$) δ 13.11 (*br*, 1H, N*H*), 8.30 – 8.10 (m, 2H, H-arom), 7.47 – 7.29 (m, 5H, H-arom), 7.28 – 7.06 (m, 7H, H-arom), 7.00 (d, *J* = 0.8 Hz, 1H, H-C(6)), 6.74 (d, *J* = 8.6 Hz, 4H, H-arom), 5.89 (dd, *J* = 6.3, 4.6 Hz, 1H, H-C(1’)), 4.61 (dd, *J* = 7.2, 4.5 Hz, 1H, H-C(4’)), 4.33 – 4.20 (m, 1H, H-C(5’)), 3.87 (*br*, 1H, H-C(7’)), 3.69 (s, 6H, *Me*O), 2.32 – 2.13 (m, 2H, H-C(3’), O*H*), 1.99 (s, 3H, *Me*-C(5)), 1.87 – 1.73 (m, 2H, H-C(2’), H-C(6’)), 1.66 – 1.47 (m, 2H, H-C(2’), H-C(6’)).

^{13}C NMR (75 MHz, $CDCl_3$) δ 179.61 (*C*ONH), 159.76 (C(4)), 158.74 (MeO-*C*-arom), 147.87 (C(2)), 145.47 (C-arom), 137.17 (C(6)), 136.77, 136.68, 136.03 (C-arom), 132.55, 130.21, 129.98, 128.34, 128.21, 128.03, 127.07, 113.35 (CH-arom), 111.81 (C(5)), 88.74 (*C*(Ph)$_3$), 87.13 (C(1’)), 86.12 (C(4’)), 78.17 (C(7’)), 73.31 (C(5’)), 55.35 (*Me*O-DMTr), 48.63 (C(3’)), 40.35 (C(6’)), 38.06 (C(2’)), 13.78 (*Me*-C(5)).

ESI$^+$-HRMS *m/z* calcd for $C_{40}H_{40}O_7N_3$ ([M + H]$^+$) 674.2861, found 674.2877.

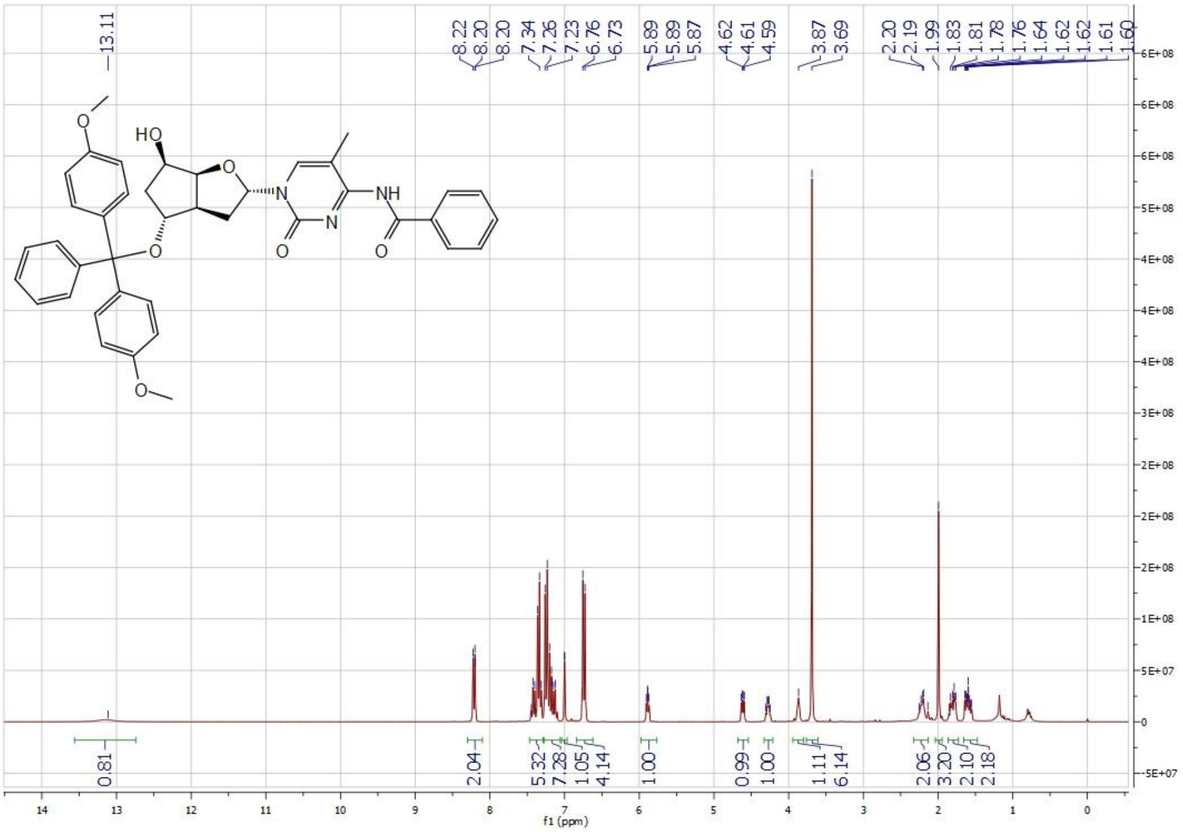

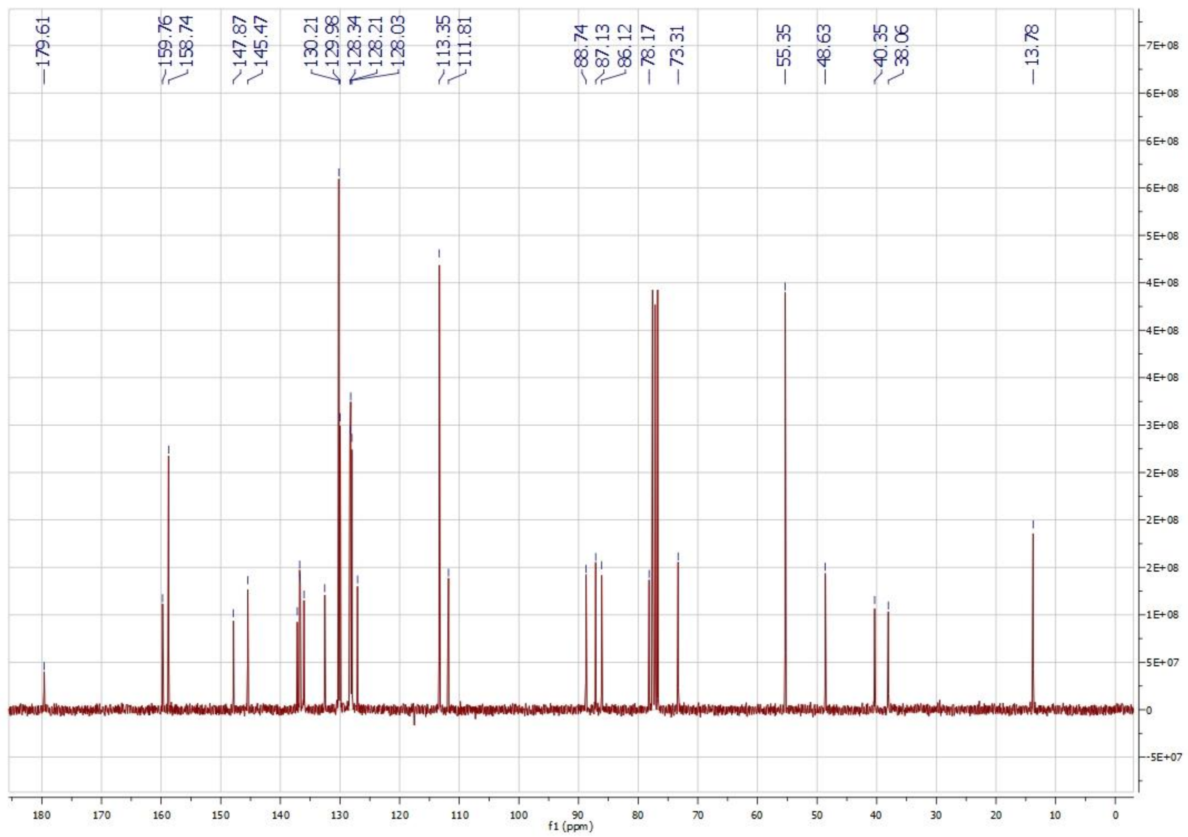

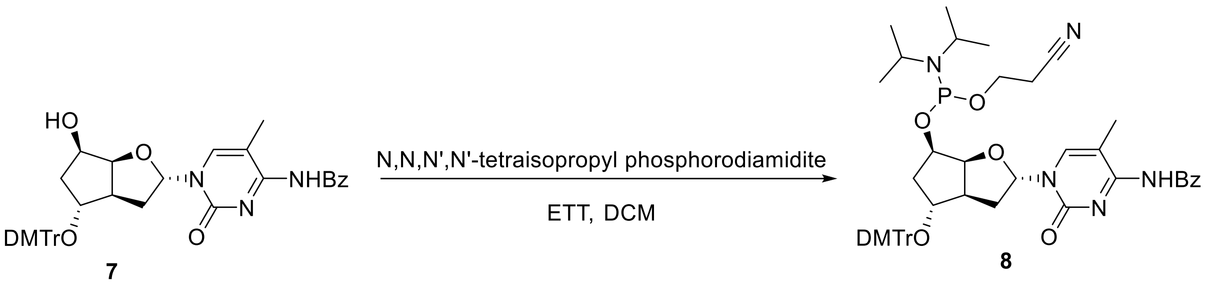

***(3'S,5'R,7'R)-N4-Benzoyl-1-{5'-O-[(2-cyanoethoxy)-diisopropylaminophosphanyl]2',3'-dideoxy-3',5'-ethano-7'-O-[(4,4'-dimethoxytriphenyl)methyl]-α-D-ribofuranosyl}-5-methylcytosine** (8)* :

To a solution of the nucleoside **7** (250 mg, 0.371 mmol) and 5-(Ethylthio)-1H-tetrazole (73 mg, 0.56 mmol) in dry DCM (8 mL) was added dropwise 2-Cyanoethyl N,N,N′,N′-tetraisopropylphosphordiamidite (0.20 mL, 0.63 mmol) at rt. After stirring for 30min, the reaction mixture was diluted with DCM (30 mL) and washed with satd $NaHCO_3$ (2 X 20 mL) and satd NaCl (20 mL). Aqueous phases were combined and extracted with DCM (20 mL). The combined organic phases were dried over $MgSO_4$, filtered and evaporated. The crude product was purified by CC (EtOAc/hexane 1:1, +0.5 % Et_3N) to yield **8** (260 mg, mixture of two isomers, 80%) as a white foam.

Data for **8**: R_f = 0.57 (EtOAc/hexane 1:1);

^{1}H NMR (300 MHz, $CDCl_3$) δ 13.26 (*br*, 1H, N*H*), 8.32 (d, *J* = 7.2 Hz, 2H, H-arom), 7.58 – 7.39 (m, 5H, H-arom), 7.38 – 7.14 (m, 8H, H-arom, H-C(6)), 6.88 – 6.77 (m, 4H, H-arom), 6.01, 5.96 (2dd, *J* = 6.3, 4.6 Hz, 1H, H-C(1')), 4.82, 4.74 (2dd, *J* = 7.3, 4.3 Hz, 1H, H-C(4')), 4.42 (td, *J* = 10.6, 6.0 Hz, 1H, H-C(5')), 3.97 (*br*, 1H, H-C(7')), 3.91 – 3.68 (m, 8H, *Me*O, OCH_2CH_2CN), 3.59 (dtd, *J* = 16.7, 6.7, 3.4 Hz, 2H, $(Me_2CH)_2N$)), 2.62 (dt, *J* = 15.5, 6.4 Hz, 2H, OCH_2CH_2CN), 2.49 – 2.23 (m, 1H, H-C(3')), 2.11, 2.09 (2d, *J* = 0.5 Hz, 3H, *Me*-C(5)), 2.00 – 1.82 (m, 2H, H-C(6'), H-C(2')), 1.82 – 1.55 (m, 2H, H-C(6'), H-C(2')), 1.17 (dd, *J* = 16.3, 6.8 Hz, 12H, $(Me_2CH)_2N$).

^{13}C NMR (101 MHz, $CDCl_3$) δ 179.60 (*C*ONH), 159.97 (C(4)), 158.76 (MeO-*C*-arom), 147.81, 147.70 (C(2)), 145.54 (C-arom), 137.34, 136.83 (C(6)), 136.77, 136.72, 136.65, 136.55 (C-arom), 132.45, 130.22, 130.20, 129.96, 128.34, 128.31, 128.18, 128.00, 127.04 (CH-arom), 117.89, 117.71 (OCH_2CH_2CN), 113.35 (CH-arom), 111.60, 111.36 (C(5)), 89.24, 89.01 (*C*$(Ph)_3$), 87.16, 87.12 (C(1')), 85.78, 85.62 ($J_{C,P}$ = 4.3, 3.2 Hz, C(4')), 78.20, 77.98 (C(7')), 74.68, 74.37($J_{C,P}$ = 13.4, 18.2 Hz, C(5')), 58.70, 58.44 ($J_{C,P}$ = 18.5, 20.0 Hz, (OCH_2CH_2CN)), 55.36, 55.33 (*Me*O-DMTr), 48.65, 48.44 (C(3')), 43.27, 43.14 ($J_{C,P}$ = 12.4, 12.3 Hz $(Me_2CH)_2N$), 39.87, 39.64 ($J_{C,P}$ = 3.4, 3.7 Hz (C(6')), 38.30, 38.22 (C(2')), 24.80, 24.72, 24.70, 24.67, 24.63 $(Me_2CH)_2N$), 20.39, 20.37 ($J_{C,P}$ = 7.2, 6.8 Hz, OCH_2CH_2CN), 13.72 (*Me*-C(5)).

^{31}P NMR (121 MHz, $CDCl_3$) δ 148.18, 147.96.

ESI^+-HRMS *m/z* calcd for $C_{49}H_{57}O_8N_5P$ ($[M + H]^+$) 874.3939, found 874.3946.

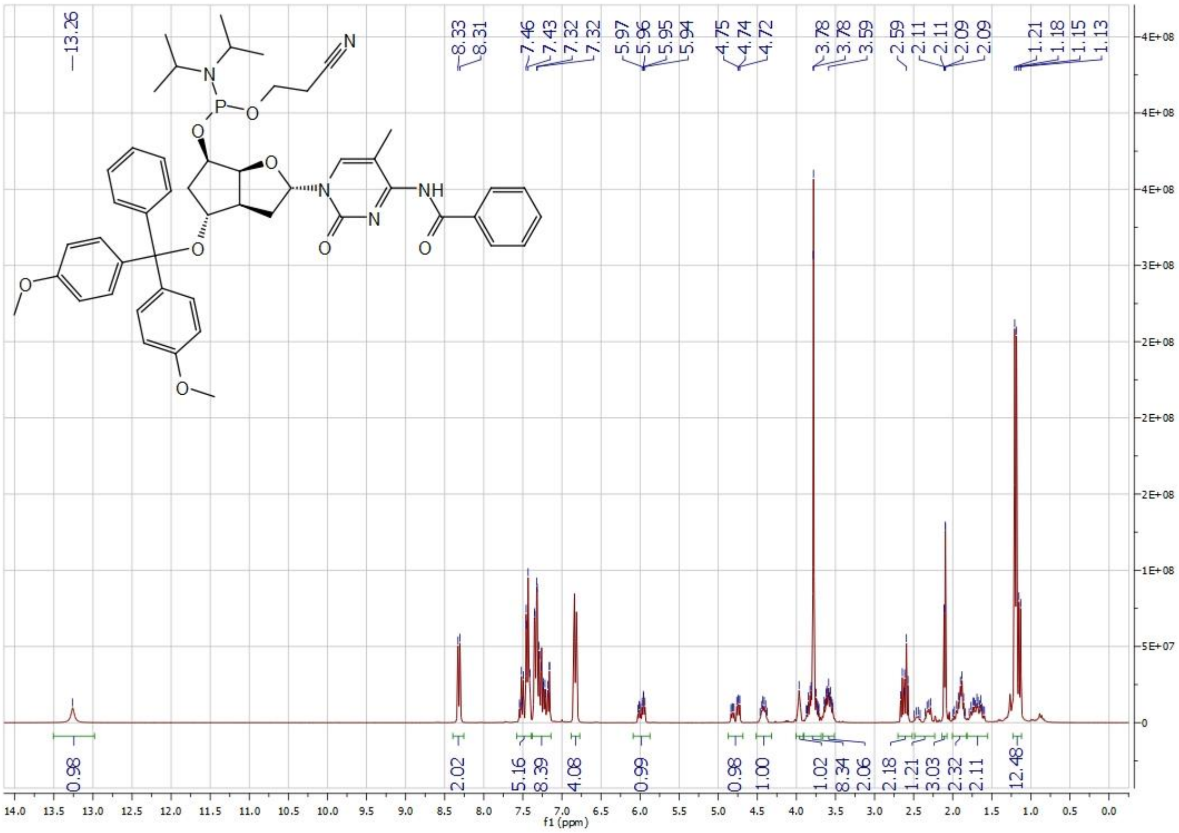
13.26
8.33
8.31
7.46
7.43
7.32
7.32
5.97
5.96
5.95
5.94
4.75
4.74
4.72
3.78
3.78
3.59
2.59
2.11
2.11
2.09
2.09
1.21
1.18
1.15
1.13
0.98
2.02
5.16
8.39
4.08
0.99
0.98
1.00
1.02
8.34
2.06
2.18
1.21
3.03
2.32
2.11
12.48
f1 (ppm)

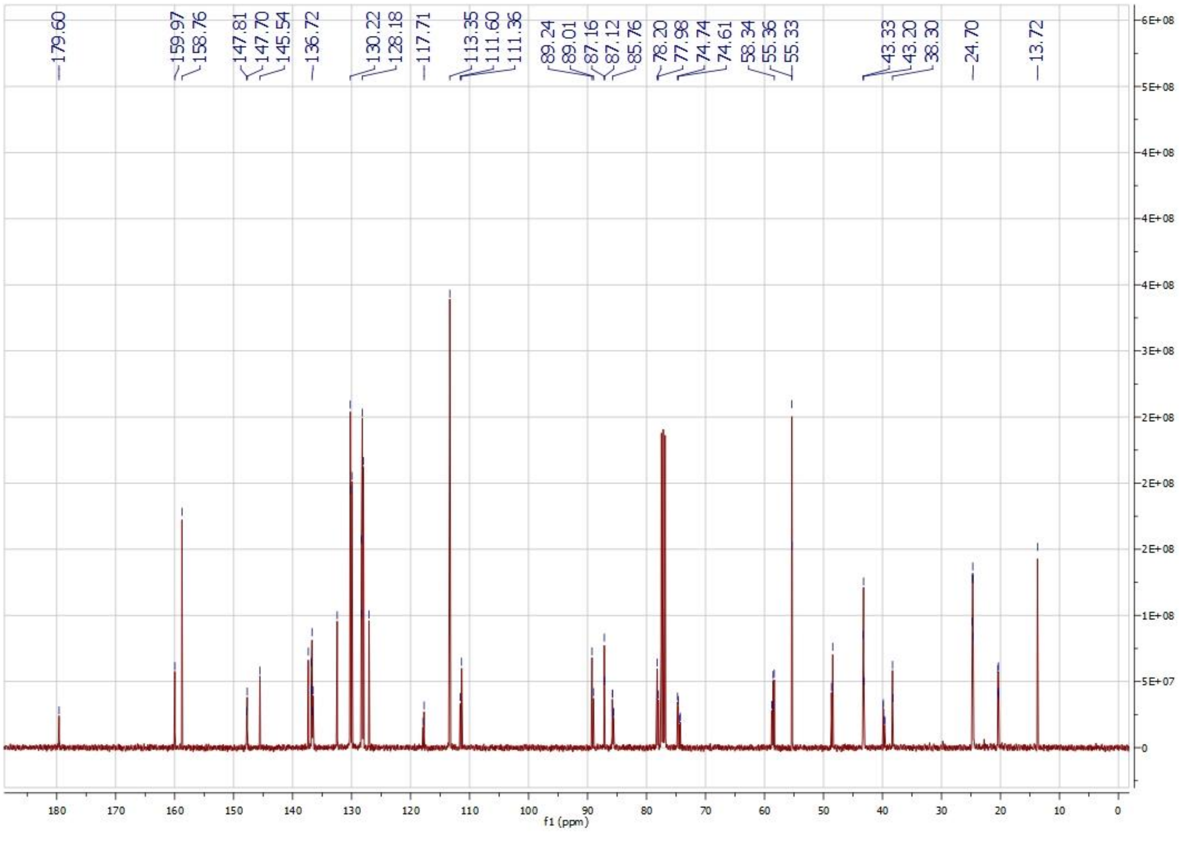
179.60
159.97
158.76
147.81
147.70
145.54
136.72
130.22
128.18
117.71
113.35
111.60
111.36
89.24
89.01
87.16
87.12
85.76
78.20
77.98
74.74
74.61
58.34
55.36
55.33
43.33
43.20
38.30
24.70
13.72
f1 (ppm)

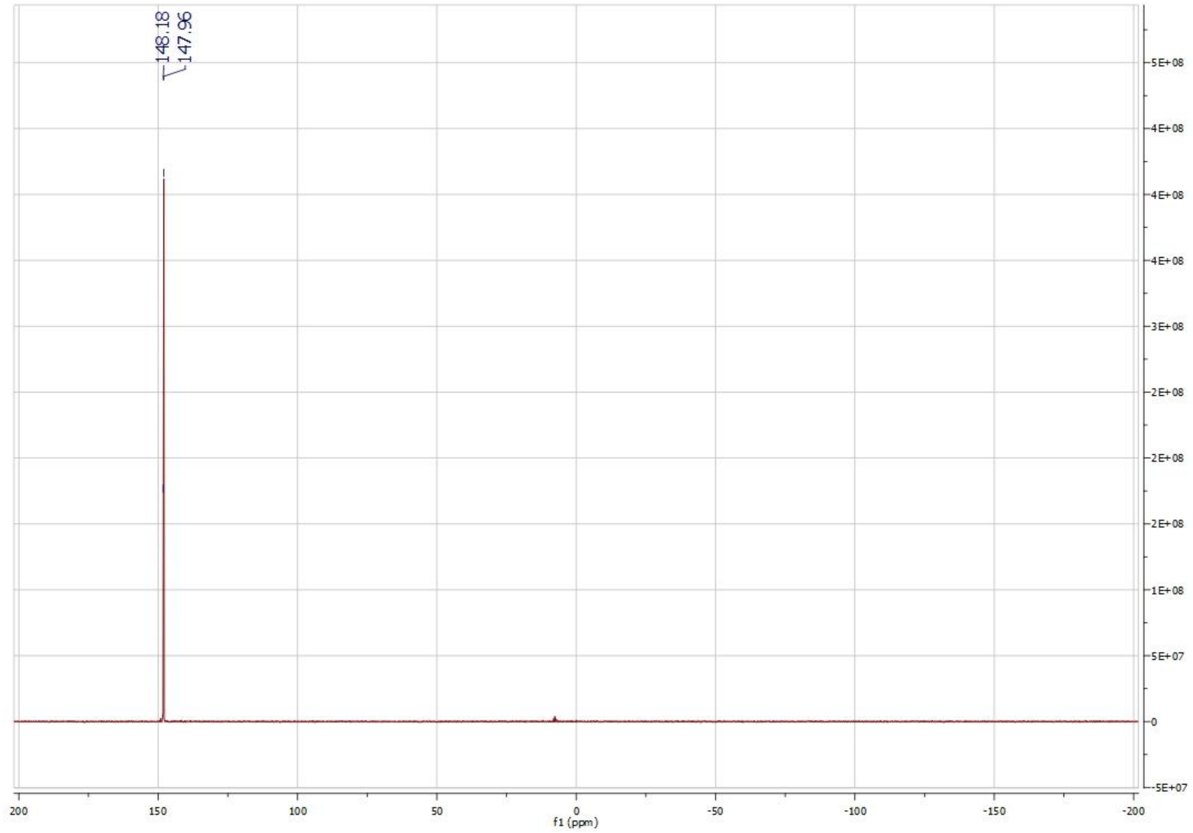

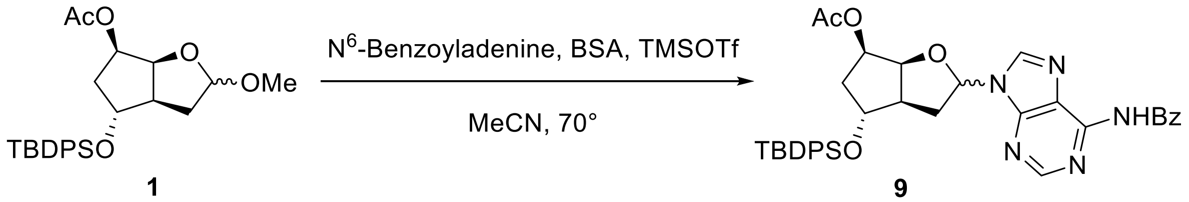

1 **9**

***(3'R,5'R,7'R)-N6-Benzoyl-9-{5'-O-acetyl-7'-[(tert-butyldiphenylsilyl)oxy]-2',3'-dideoxy-3',5'-ethano-α,β-D-ribofuranosyl} adenine** (9)* :

To a suspension of the sugar **1** (1.86 g, 4.10 mmol) and N^6-Benzoyladenine (1.96 g, 8.20 mmol) in dry MeCN (40 mL) was added BSA (4.00 mL, 16.4 mmol) at rt. After stirring for 25 min, the suspension became a clear solution and then was heated to 70°C. TMSOTf (1.48 mL, 8.20 mmol) was added dropwise and the solution was further stirred for 20 min at 70°C. The solution was then cooled down to rt, quenched with addition of satd $NaHCO_3$ (100 mL) and extracted with EtOAc (4 X 50 mL). The combined organic phases were dried over $MgSO_4$, filtered and evaporated. The crude product was purified by CC (2% MeOH in DCM) to yield a mixture of **9** (1.74 g, 64%) in an anomeric ratio α/β ≈ 4:1 as a white foam.

Data for **9**: R_f = 0.33 (EtOAc/hexane 4:1);

^{1}H NMR (400 MHz, $CDCl_3$) δ 9.33 (*br*, 1H, N*H*), 8.68 (d, *J* = 5.4 Hz, 0.8H, H-C(2)), 8.64 (d, *J* = 5.6 Hz, 0.2H, H-C(2)), 8.10 (d, *J* = 1.5 Hz, 0.2H. H-C(8)), 7.99 (d, *J* = 7.3 Hz, 2H, H-arom), 7.95 (s, 0.8H, H-C(8)), 7.63 (t, *J* = 8.7 Hz, 4H, H-arom), 7.55 (dd, *J* = 13.0, 6.4 Hz, 1H, H-arom), 7.50 – 7.34 (m, 8H, H-arom), 6.20 (dd, *J* = 6.3, 2.5 Hz, 0.8H, H-C(1')), 6.05 (t, *J* = 6.5 Hz, 0.2H, H-C(1')), 5.43 –

5.32 (m, 1H, H-C(5')), 5.03 – 4.97 (m, 0.8H, H-C(4')), 4.83 (t, *J* = 6.0 Hz, 0.2H, H-C(4')), 4.14 (*br*, 0.2H, H-C(7')), 4.08 (d, *J* = 3.7 Hz, 0.8H, H-C(7')), 3.02 (dd, *J* = 16.1, 6.6 Hz, 0.8H, H-C(3')), 2.83 (dd, *J* = 16.9, 7.7 Hz, 0.2H, H-C(3')), 2.59 – 2.39 (m, 1H, H-C(2')), 2.18 – 2.11 (m, 1H, H-C(6')), 2.07, 2.07 (2s, *Me*CO_2), 2.02, 2.01 (2s, 0.6H, *Me*CO_2), 2.01 – 1.92 (m, 1H, H-C(6')), 1.91 – 1.80 (m, 1H, H-C(3')), 1.07 (s, 9H, (C*H*$_3$)$_3$-C-Si).

^{13}C NMR (101 MHz, $CDCl_3$) δ 170.57, 170.49 (Me*C*O_2), 164.82 (*C*ONH), 152.50 (C(2)), 151.27 (C(4)), 149.56 (C(6)), 141.37, 141.06 (C(8)), 135.72, 135.68, 135.66 (CH-arom), 133.67, 133.57, 133.24, 133.22 (C-arom), 132.73, 130.03, 129.98, 128.80, 128.78, 127.92, 127.86, 127.85 (CH-arom), 123.61 (C(5)), 87.19, 86.17 (C(1')), 83.22, 80.96 (C(4'), 76.50, 76.04 (C(7')), 74.38 (C(5')), 51.07 (C(3')), 37.29, 37.15, 36.80, 36.60 (C(2'), C(6')), 26.89 ((*C*H$_3$)$_3$-C-Si), 20.97, 20.90 (*Me*CO_2), 19.01 ((CH$_3$)$_3$-*C*-Si).

ESI$^+$-HRMS *m/z* calcd for $C_{37}H_{40}O_5N_5Si$ ([M + H]$^+$) 662.2793, found 662.2787.

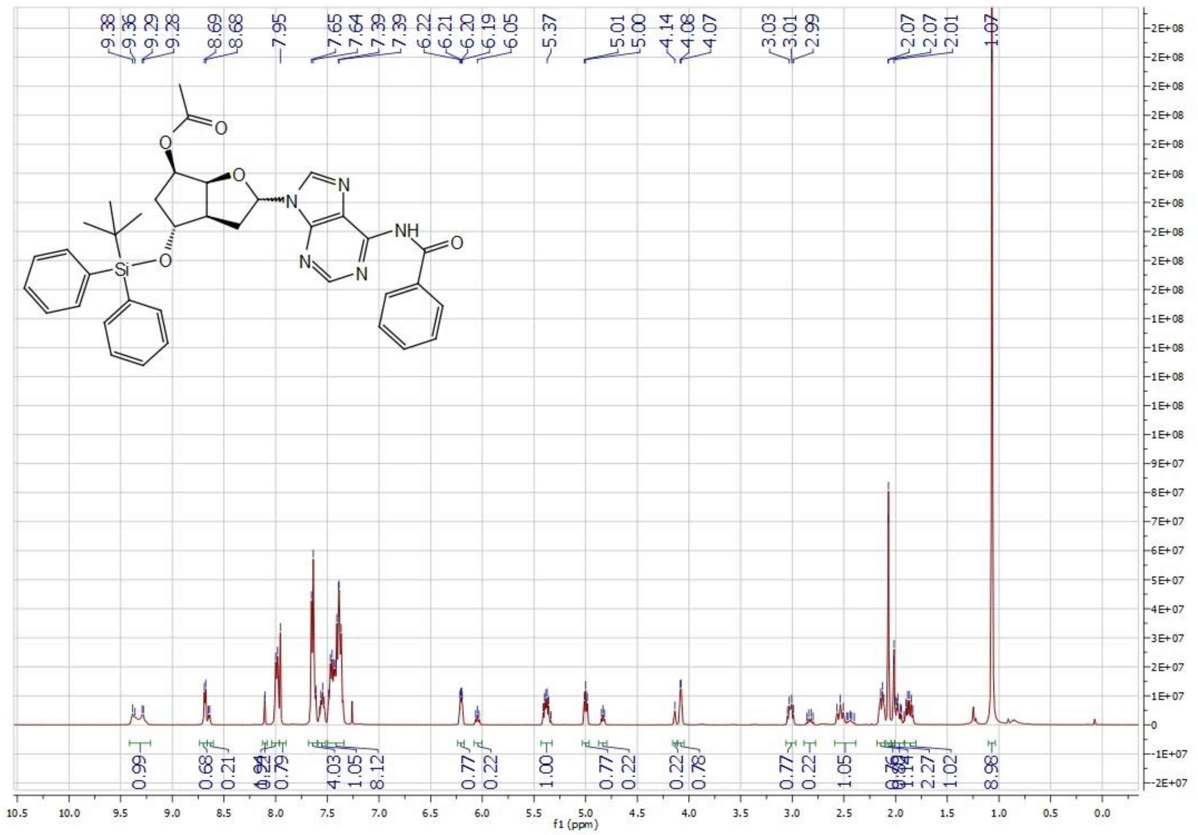

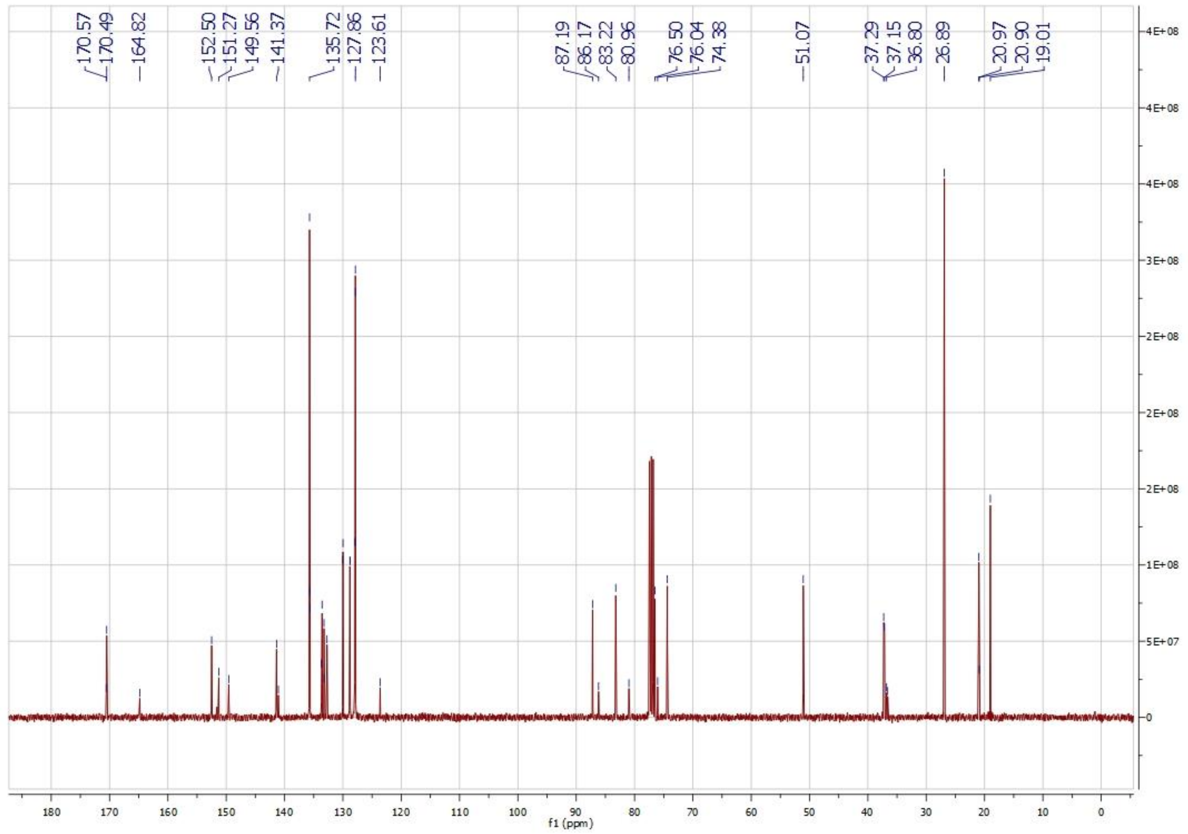

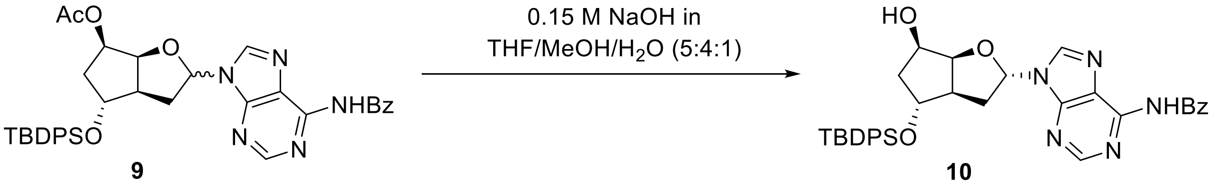

(3’R,5’R,7’R)-N6-Benzoyl-9-{7’-[(tert-butyldiphenylsilyl)oxy]-2’,3’-dideoxy-3’,5’-ethano-α-D-ribofuranosyl} adenine (10) :

The nucleoside **9** (1.74 g, 2.64 mmol) was dissolved in 0.15 M NaOH in THF/methanol/H_2O (5:4:1, 80 mL) at 0°C. The reaction was stirred for 20 min and quenched by addition of NH_4Cl (1.06 g). Solvents were then removed under reduced pressure and the product purified by CC (5% isopropanol in DCM) to yield **10-α** (836 mg, 51%) and **10-β** (287 mg, 18%) as white foams.

Data for **10-α**: R_f = 0.35 (5% MeOH in DCM);

^{1}H NMR (300 MHz, $CDCl_3$) δ 9.34 (s, 1H, N*H*), 8.71 (s, 1H, H-C(2)), 8.02 (d, *J* = 7.4 Hz, 2H, H-arom)), 7.92 (s, 1H, H-C(8)), 7.68 – 7.58 (m, 4H, H-arom), 7.58 – 7.31 (m, 9H, H-arom), 6.23 (dd, *J* = 6.7, 2.4 Hz, 1H, H-C(1’)), 4.74 (dd, *J* = 6.6, 4.9 Hz, 1H, H-C(4’)), 4.49 (dt, *J* = 12.5, 6.3 Hz, 1H, H-C(5’)), 4.10 (*br*, 1H, H-C(7’)), 3.07 (d, *J* = 6.7 Hz, 1H, O*H*), 2.92 (dd, *J* = 15.4, 7.3 Hz, 1H, H-C(3’)), 2.52 – 2.35 (m, 1H, H-C(2’)), 2.10 – 1.97 (m, 1H, H-C(6’)), 1.94 – 1.77 (m, 2H, H-C(2’), H-C(6’)), 1.06 (s, 9H, (CH_3)$_3$-C-Si).

^{13}C NMR (75 MHz, $CDCl_3$) δ 164.98 (*C*ONH), 152.65 (C(2)), 151.31 (C(4)), 149.69 (C(6)), 140.93 (C(8)), 135.74 (CH-arom), 133.82, 133.68, 133.39 (C-arom), 132.77, 130.02, 129.98, 128.76, 128.06, 127.87, 127.85 (CH-arom), 123.38 (C(5)), 87.16 (C(1')), 85.35 (C(4')), 77.40 (C(7')), 72.79 (C(5')), 50.63 (C(3')), 40.86 (C(6')), 37.25 (C(2')), 26.94 ((CH_3)$_3$-C-Si), 19.05 ((CH_3)$_3$-*C*-Si).

ESI$^+$-HRMS *m/z* calcd for $C_{35}H_{38}O_4N_5Si$ ([M + H]$^+$) 620.2688, found 620.2671.

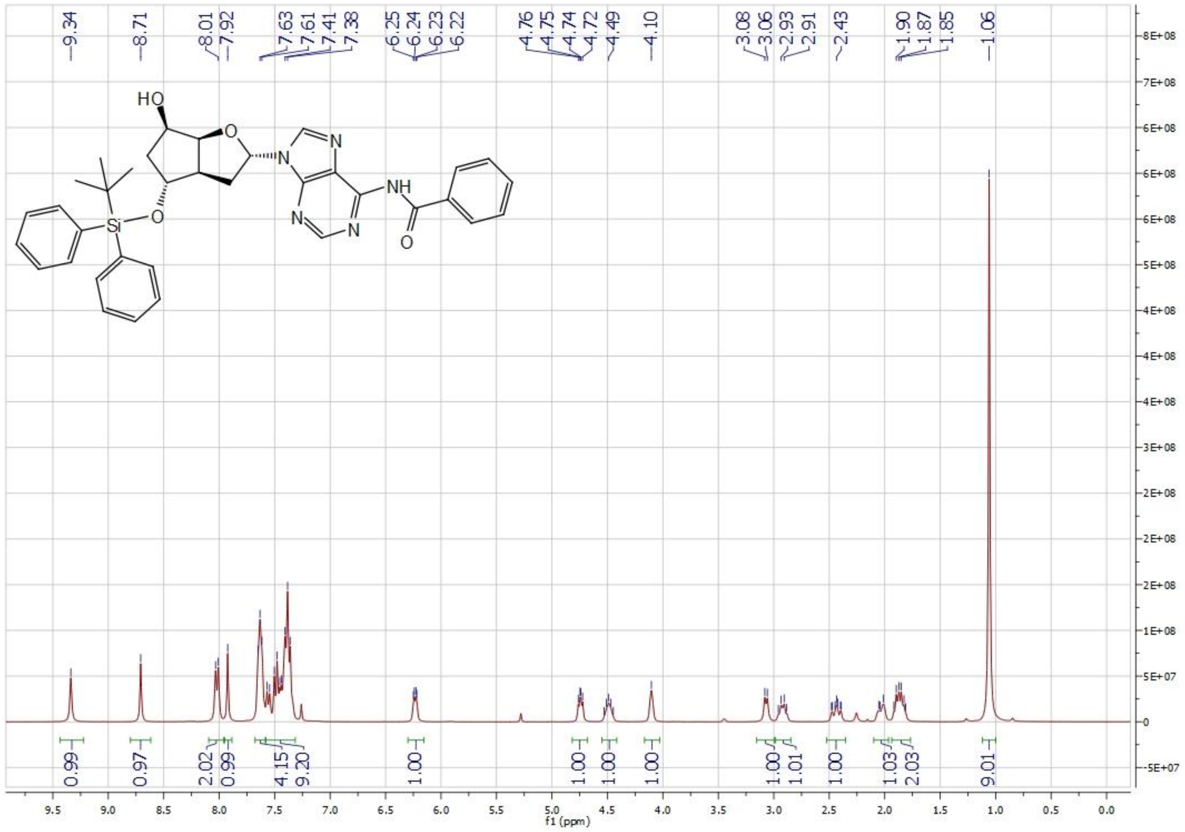

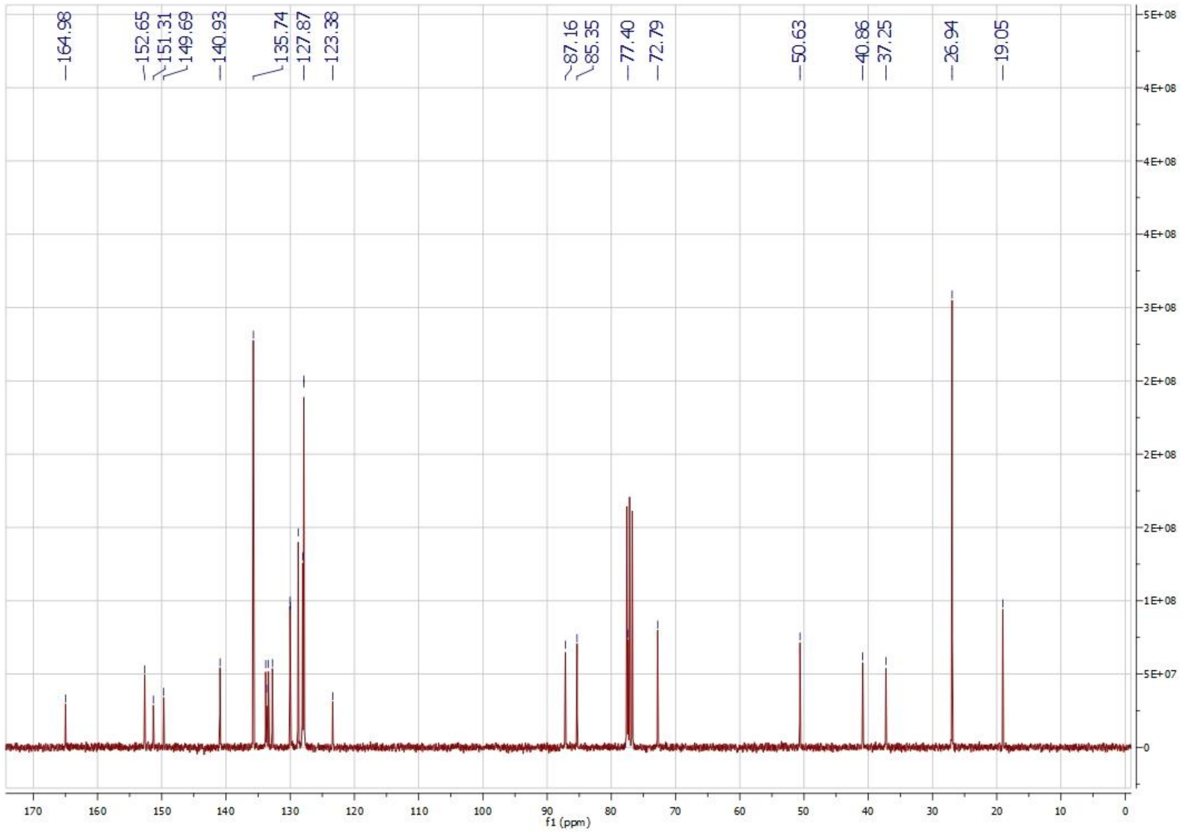

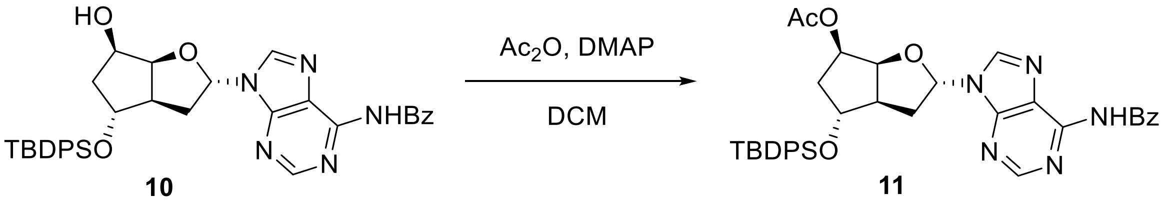

(3'R,5'R,7'R)-N6-Benzoyl-9-{5'-O-acetyl-7'-[(tert-butyldiphenylsilyl)oxy]-2',3'-dideoxy-3',5'-ethano-α-D-ribofuranosyl} adenine (11) :

To a solution of the nucleoside **10** (1.09 g, 1.75 mmol) and 4-dimethylaminopyridine (321 mg, 2.63 mmol) in dry DCM (50 mL) was added acetic anhydride (0.83 mL, 8.8 mmol) at rt. After stirring overnight, the reaction was quenched by addition of satd $NaHCO_3$ (50 mL). The phases were separated and aqueous phase further extracted with DCM (2 X 80 mL). The combined organic phases were dried over $MgSO_4$, filtered and evaporated. The crude product was purified by CC (2.5% MeOH in DCM) to yield **11** (1.04 g, 90%) as white foams.

Data for **11**: R_f = 0.33 (EtOAc/hexane 4:1);

^{1}H NMR (300 MHz, $CDCl_3$) δ 8.99 (*br*, 1H, N*H*), 8.73 (s, 1H, H-C(2)), 8.09 – 7.99 (m, 2H, H-arom), 7.98 (s, 1H, H-C(8)), 7.70 – 7.58 (m, 5H, H-arom), 7.57 – 7.48 (m, 2H, H-arom), 7.47 – 7.34 (m, 6H, H-arom), 6.22 (dd, *J* = 6.8, 3.2 Hz, 1H, H-C(1')), 5.45 – 5.35 (m, 1H, H-C(5')), 5.01 (dd, *J* = 6.7, 5.0 Hz, 1H, H-C(4')), 4.09 (d, *J* = 4.1 Hz, 1H, H-C(7')), 3.02 (dt, *J* = 9.5, 6.5 Hz, 1H, H-C(3')), 2.55 (ddd, *J* = 13.5, 10.0, 3.2 Hz, 1H, H-C(2')), 2.15 (dd, *J* = 13.2, 6.2 Hz, 1H, H-C(6')), 2.09 (s, 3H, *Me*CO_2),

2.01 (dt, J = 8.0, 3.5 Hz, 1H, H-C(2')), 1.88 (dt, J = 13.6, 5.3 Hz, 1H, H-C(6')), 1.08 (s, 9H, (CH_3)$_3$-C-Si).

^{13}C NMR (101 MHz, $CDCl_3$) δ 170.61 (Me*C*O$_2$), 164.75 (*C*ONH), 152.67 (C(2)), 151.37 (C(4)), 149.64 (C(6)), 141.41 (C(8)), 135.85 (CH-arom), 133.71, 133.38 (C-arom), 132.91, 130.15, 130.10, 128.99, 128.02, 127.99, 127.97 (CH-arom), 123.64 (C(5)), 87.37 (C(1')), 83.37 (C(4')), 76.63 (C(7')), 74.51 (C(5')), 51.19 (C(3')), 37.44 (C(2')), 37.32 (C(6')), 27.01 ((*C*H$_3$)$_3$-C-Si), 21.08 (*Me*CO$_2$), 19.14 ((CH$_3$)$_3$-*C*-Si).

ESI$^+$-HRMS *m/z* calcd for $C_{37}H_{40}O_5N_5Si$ ([M + H]$^+$) 662.2793, found 662.2787.

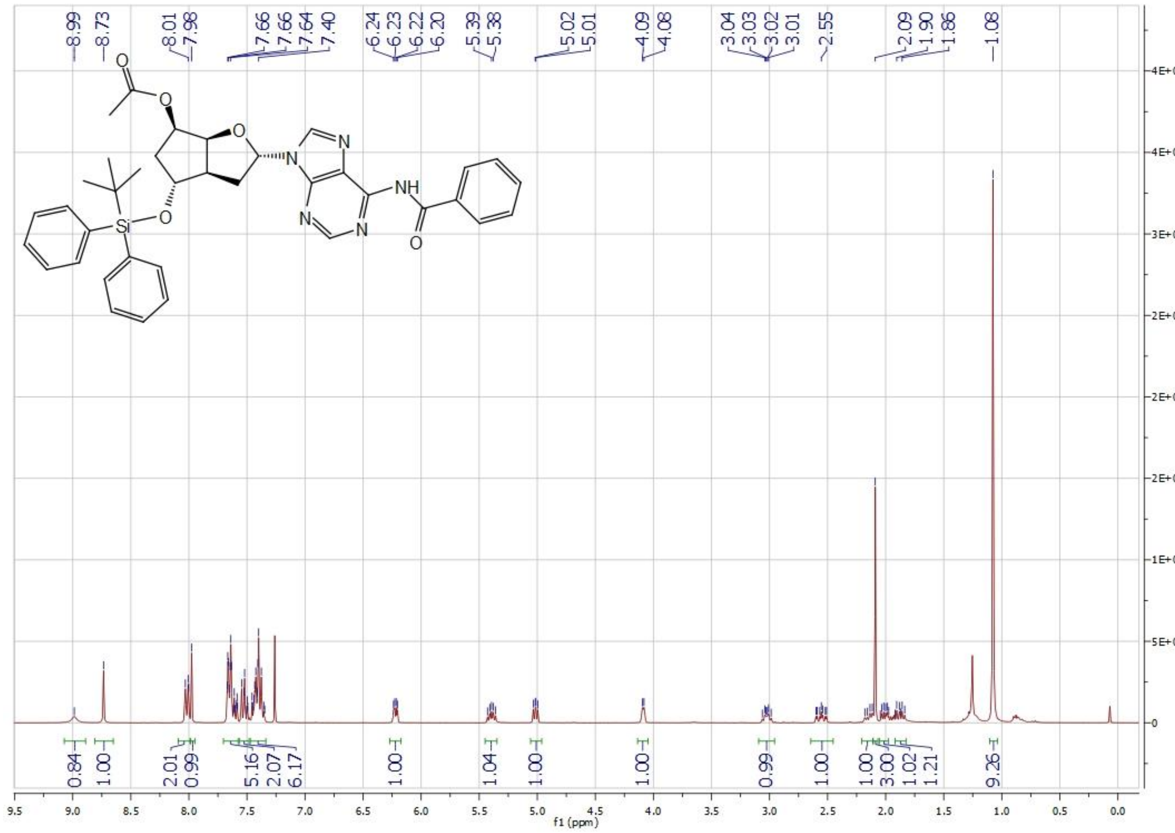

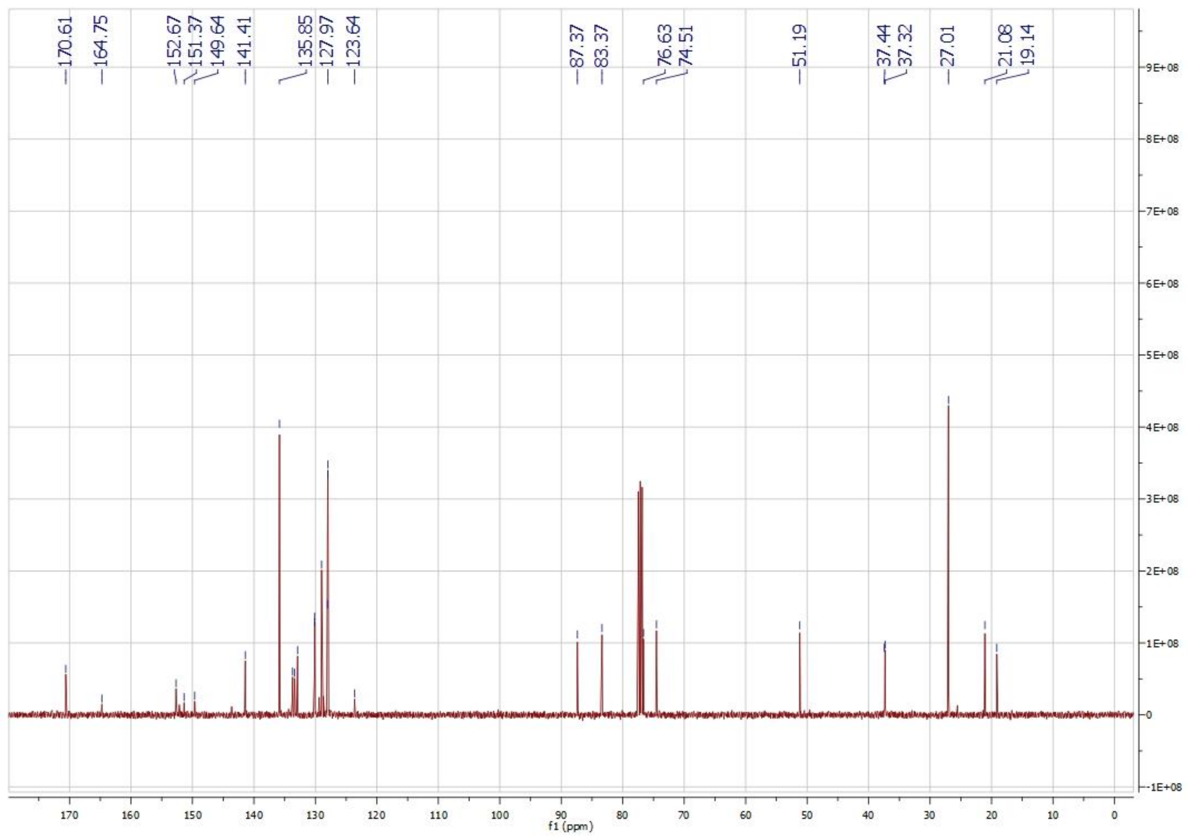

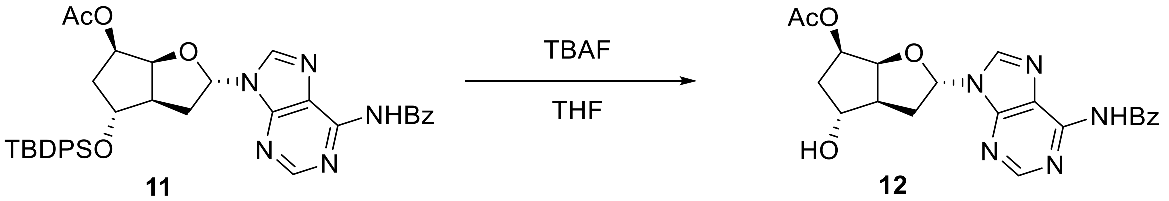

(3'S,5'R,7'R)-N6-Benzoyl-9-{5'-O-acetyl-2',3'-dideoxy-3',5'-ethano-7'-hydroxy-α-D-ribofuranosyl} adenine (12) :

To a solution of the nucleoside **11** (990 mg, 1.50 mmol) in dry THF (50 mL) was added TBAF (1M in THF, 3.0 mL, 3.0 mmol) at rt. After stirring for 3.5 hours at rt, the solution was diluted with EtOAc (30 mL) and THF was removed under reduced pressure. The mixture was then diluted with satd $NaHCO_3$ (50 mL) and extracted with DCM (4 X 50 mL). The combined organic phases were dried over $MgSO_4$, filtered and evaporated. The crude product was purified by CC (6% MeOH in DCM) to yield **12** (570 mg, 90%) as a white foam, containing traces of TBAF.

Data for **12**: R_f = 0.33 (10% MeOH in DCM);

^{1}H NMR (400 MHz, $CDCl_3$) δ 9.60 (*br*, 1H, N*H*), 8.67 (s, 1H, H-C(2)), 8.09 (s, 1H, H-C(8)), 7.96 (d, *J* = 7.4 Hz, 2H, H-arom), 7.51 (t, *J* = 7.4 Hz, 1H, H-arom), 7.42 (t, *J* = 7.5 Hz, 2H, H-arom), 6.33 (dd, *J* = 6.7, 3.1 Hz, 1H, H-C(1')), 5.25 (ddd, *J* = 9.7, 6.4, 5.3 Hz, 1H, H-C(5')), 4.92 (dd, *J* = 6.4, 5.4 Hz, 1H, H-C(1')), 4.14 (*br*, 2H, H-C(7'), O*H*), 3.06 (dd, *J* = 16.0, 6.6 Hz, 1H, H-C(3')), 2.87 (ddd, *J* = 13.2, 9.9, 3.0 Hz, 1H, H-C(2')), 2.26 – 2.17 (m, 1H, H-C2')), 2.10 – 1.98 (m, 5H, H-C(6'), *Me*CO_2).

^{13}C NMR (75 MHz, $CDCl_3$) δ 170.64 (Me*C*O_2), 165.27 (*C*ONH), 152.49 (C(2)), 151.26 (C(4)), 149.58 (C(6)), 141.64 (C(8)), 133.60 (C-arom), 132.82, 128.76, 128.06 (CH-arom), 123.30 (C(5)), 87.30 (C(1')), 83.17 (C(4')), 74.67 (C(7')), 74.20 (C(5')), 50.41 (C(3')), 37.43 (C(2')), 36.92 (C(6')), 20.96 (*Me*CO_2).

ESI$^+$-HRMS *m/z* calcd for $C_{21}H_{22}O_5N_5$ ([M + H]$^+$) 424.1615, found 424.1623.

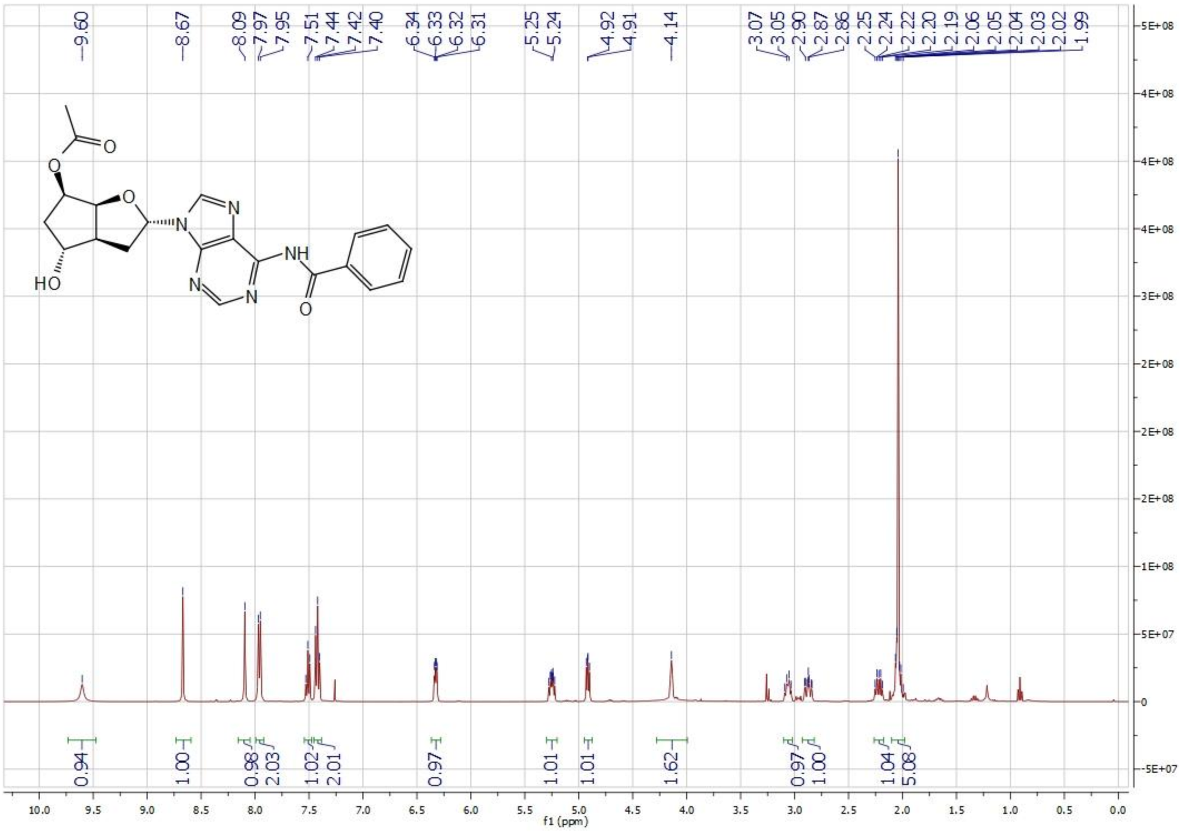

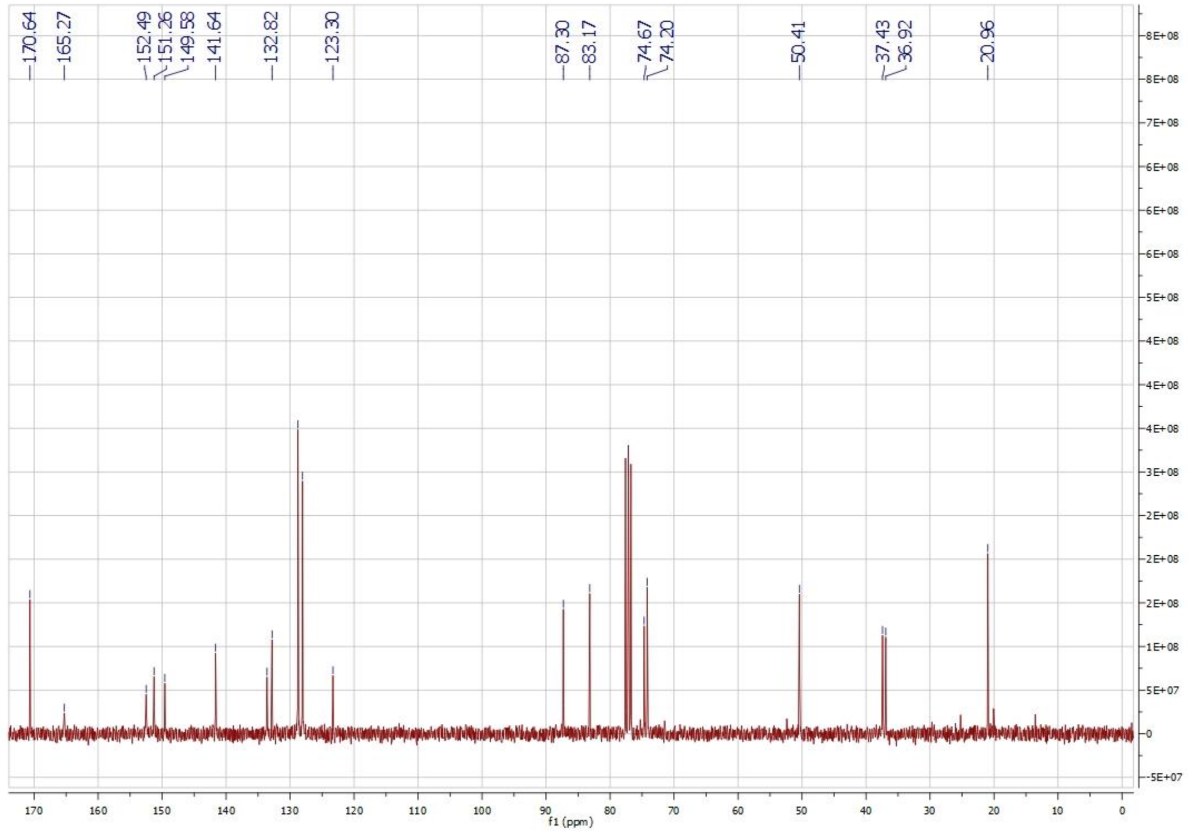

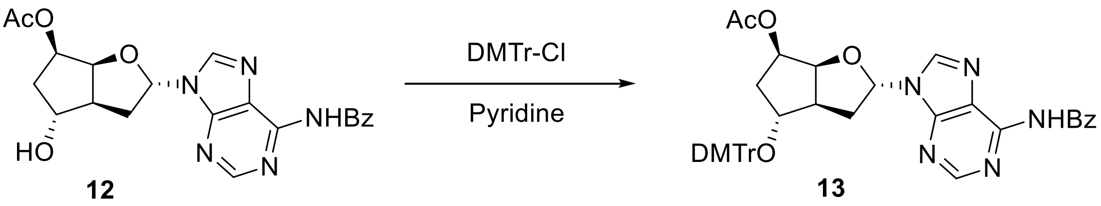

(3'S,5'R,7'R)-N6-Benzoyl-9-{5'-O-acetyl-2',3'-dideoxy-3',5'-ethano-7'- O-[(4,4'-dimethoxytriphenyl)methyl]-α-D-ribofuranosyl} adenine (13) :

To a solution of nucleoside **12** (570 mg, 1.35 mmol) in dry pyridine (16 mL) was added DMTr-Cl (1.37 g, 4.04 mmol) at rt. The solution was stirred for 1 day and then was diluted with satd $NaHCO_3$ (100 mL) and extracted with DCM (3 X 80 mL). The combined organic phases were dried over $MgSO_4$, filtered and evaporated. The crude product was purified by CC (2% MeOH in DCM, +0.5 % Et_3N) to yield **13** (876 mg, 89%) as a yellow foam.

Data for **13**: R_f = 0.81 (5% MeOH in DCM);

^{1}H NMR (300 MHz, $CDCl_3$) δ 9.42 (d, J = 14.6 Hz, 1H, N*H*), 8.73 (s, 1H, H-C(2)), 8.03 (d, J = 7.6 Hz, 2H, H-arom), 7.93 (s, 1H, H-C(8)), 7.66 – 7.55 (m, 1H, H-arom), 7.55 – 7.45 (m, 4H, H-arom), 7.45 – 7.22 (m, 7H, H-arom), 6.87 (d, J = 8.7 Hz, 4H, H-arom), 6.25 (dd, J = 6.6, 2.4 Hz, 1H, H-C(1')), 5.47 – 5.33 (m, 1H, H-C(5')), 4.89 (dd, J = 6.7, 4.9 Hz, 1H, H-C(4')), 4.02 (d, J = 2.5 Hz, 1H, H-C(7')), 3.79 (s, 6H, *Me*O), 2.58 (dd, J = 16.0, 6.9 Hz, 1H, H-C(3')), 2.38 (ddd, J = 12.7, 10.0, 2.4 Hz, 1H, H-C(2')), 2.11 (s, 3H, *Me*CO_2), 2.09 – 1.87 (m, 3H, H-C(2'), H-C(6')).

^{13}C NMR (75 MHz, $CDCl_3$) δ 170.40 (Me*C*O_2), 164.84 (*C*ONH), 158.66 (MeO-*C*-arom), 152.45 (C(2)), 151.22 (C(4)), 149.51 (C(6)), 145.23 (C-arom), 141.23 (C(8)), 136.51, 133.65 (C-arom), 132.68, 130.12, 128.75, 128.33, 127.95, 127.90, 127.03 (CH-arom), 123.55 (C(5)), 113.27 (CH-arom), 87.19 (*C*$(Ph)_3$), 87.12 (C(1')), 83.25 (C(4')), 77.16 (C(7')), 74.41 (C(5')), 55.23 (*Me*O-DMTr), 49.23 (C(3')), 37.61 (C(2')), 36.22 (C(6')), 20.98 (*Me*CO_2).

ESI$^+$-HRMS *m/z* calcd for $C_{42}H_{40}O_7N_5$ ($[M + H]^+$) 726.2922, found 726.2905.

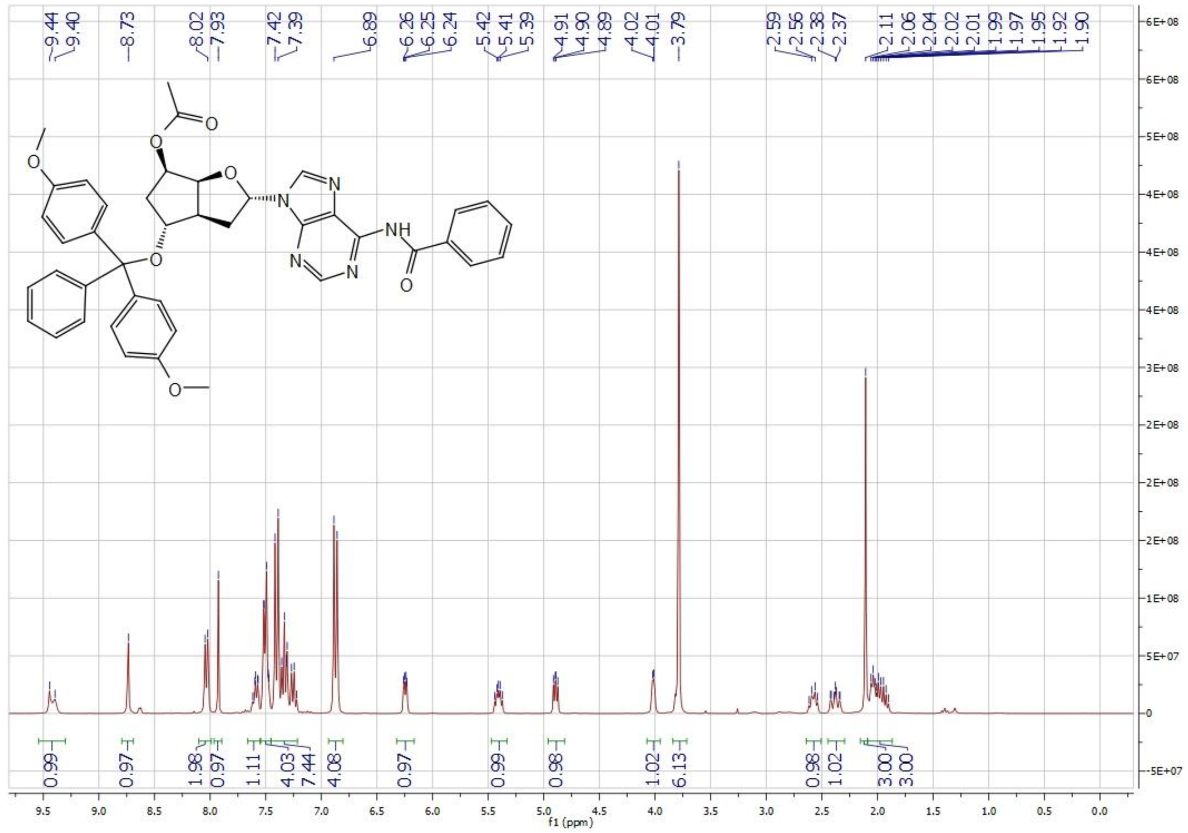

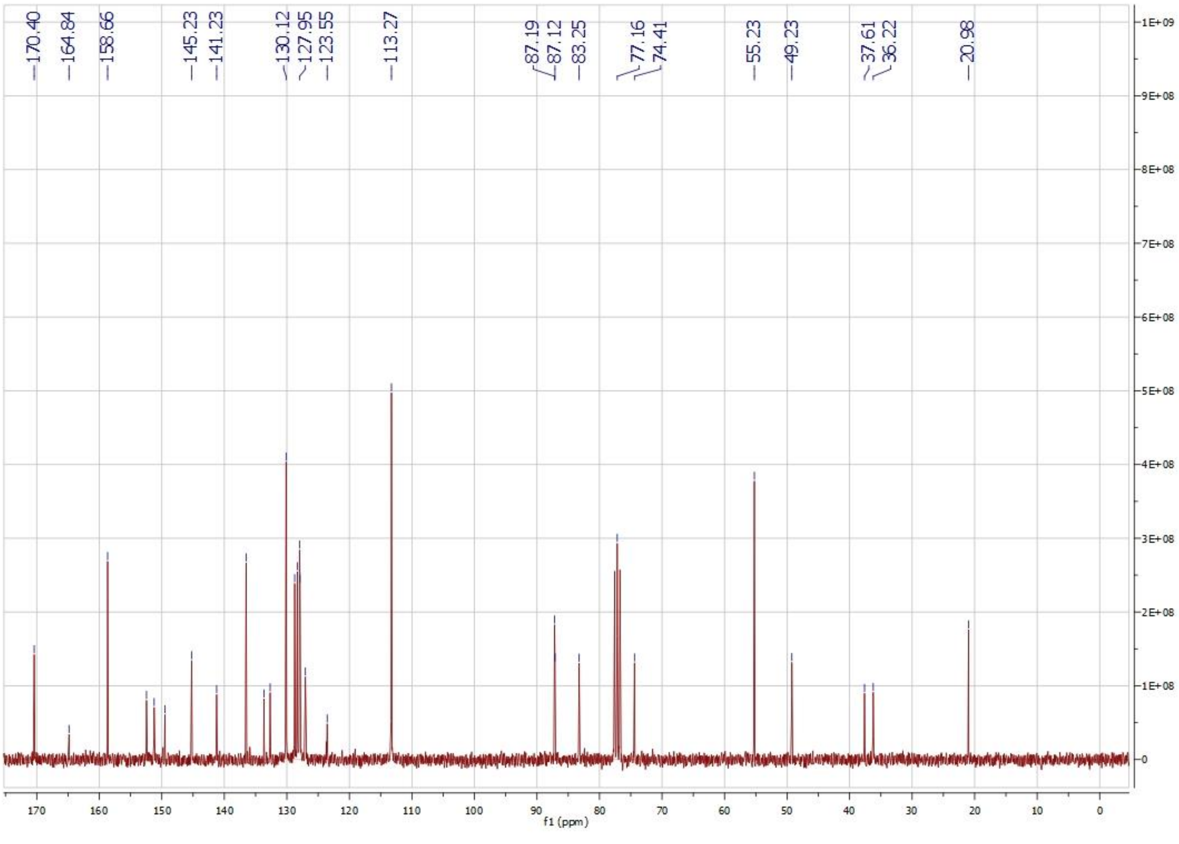

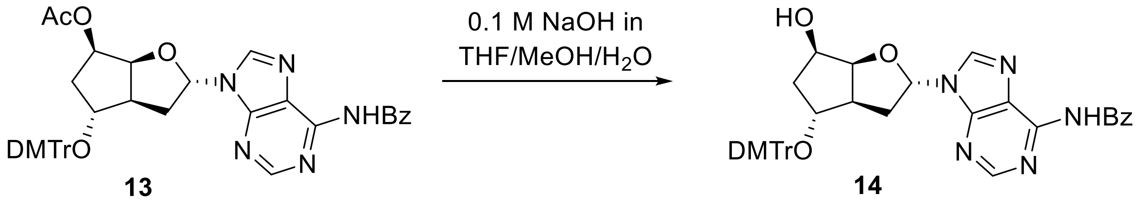

13 **14**

(3'S,5'R,7'R)-N6-benzoyl-9-{2',3'-dideoxy-3',5'-ethano-7'- O-[(4,4'-dimethoxytriphenyl)methyl]-α-D-ribofuranosyl} adenine (14) :

The nucleoside **13** (870 mg, 1.20 mmol) was dissolved in 0.1 M NaOH in THF/methanol/H_2O (5:4:1, 50 mL) at 0°C. The reaction was stirred for 30 min at 0°C and then quenched by addition of NH_4Cl (321 mg). The solution was diluted with satd $NaHCO_3$ (100 mL) and extracted with DCM (4 X 80 mL). The combined organic phases were dried over $MgSO_4$, filtered and evaporated. The crude product was purified by CC (3% MeOH in DCM, +0.5 % Et_3N) to yield **14** (777 mg, 94%) as white foams.

Data for **14**: R_f = 0.26 (5% MeOH in DCM);

^{1}H NMR (300 MHz, $CDCl_3$) δ 9.39 (s, 1H, N*H*), 8.61 (s, 1H, H-C(2)), 7.93 (d, *J* = 7.4 Hz, 2H, H-arom), 7.75 (s, 1H, H-C(8)), 7.46 (t, *J* = 7.3 Hz, 1H, H-arom), 7.40 – 7.31 (m, 4H, H-arom), 7.29 – 7.16 (m, 6H, H-arom), 7.11 (t, *J* = 7.2 Hz, 1H, H-arom), 6.73 (d, *J* = 8.7 Hz, 4H, H-arom), 6.12 (dd, *J* = 6.5, 1.9 Hz, 1H, H-C(1')), 4.53 (dd, *J* = 7.5, 4.5 Hz, 1H, H-C(4')), 4.32 (*br*, 1H, H-C(5')), 3.90 (t, *J* = 4.5 Hz, 1H, H-C(7')), 3.66, 3.65 (2s, 6H, *Me*O), 3.31 (*br*, 1H, O*H*), 2.36 (dd, *J* = 16.5, 8.1 Hz, 1H, H-C(3')), 2.04 (ddd, *J* = 12.0, 9.9, 2.0 Hz, 1H, H-C(2')), 1.92 – 1.69 (m, 3H, H-C(2'), H-C(6')).

^{13}C NMR (75 MHz, $CDCl_3$) δ 164.92 (*C*ONH), 158.64 (MeO-*C*-arom), 152.60 (C(2)), 151.28 (C(4)), 149.61 (C(6)), 145.44 (C-arom), 140.71 (C(8)), 136.77, 133.65 (C-arom), 132.72, 130.15, 130.12, 128.73, 128.39, 128.04, 127.96, 127.02 (CH-arom), 123.27 (C(5)), 113.28 (CH-arom), 87.11 (C(1')), 87.01 (*C*(Ph)$_3$), 85.60 (C(4')), 78.16 (C(7')), 72.72 (C(5')), 55.28 (*Me*O-DMTr), 48.89 (C(3')), 39.93 (C(6')), 37.55 (C(2')).

ESI$^+$-HRMS *m/z* calcd for $C_{40}H_{38}O_6N_5$ ([M + H]$^+$) 684.2817, found 684.2800.

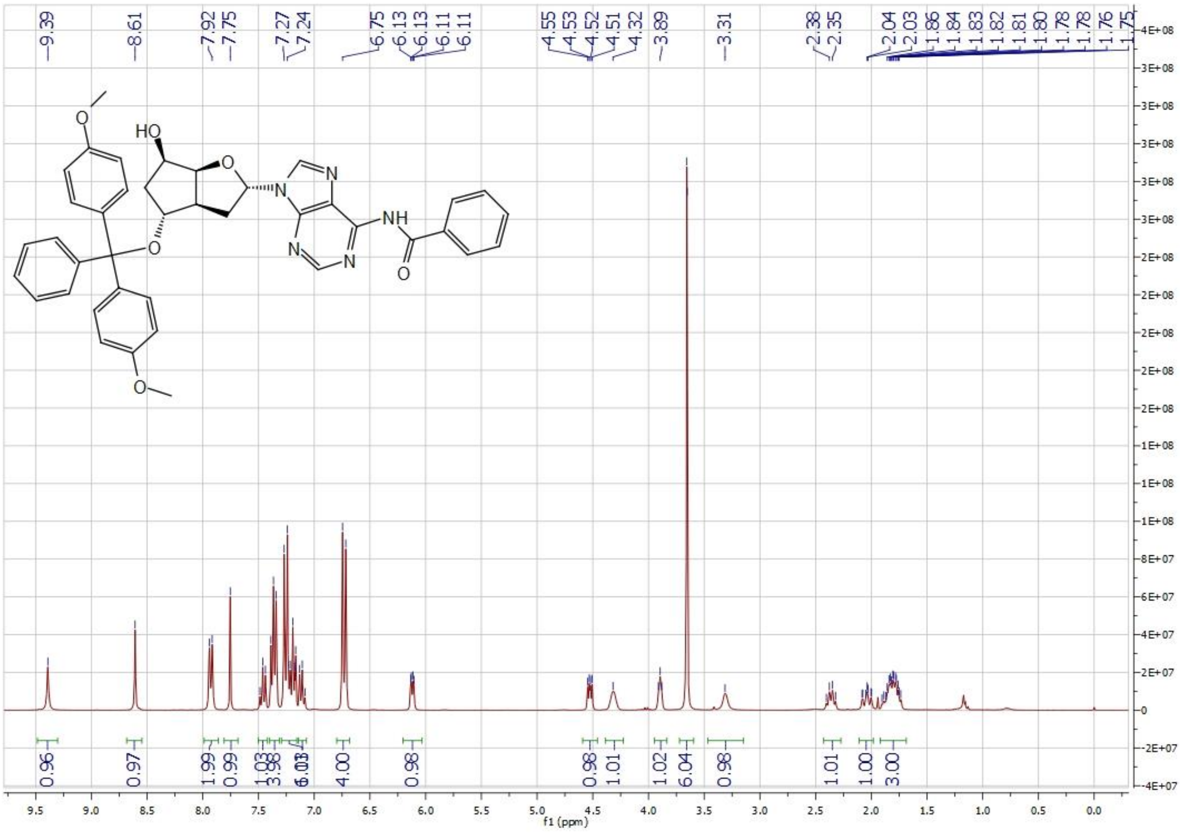

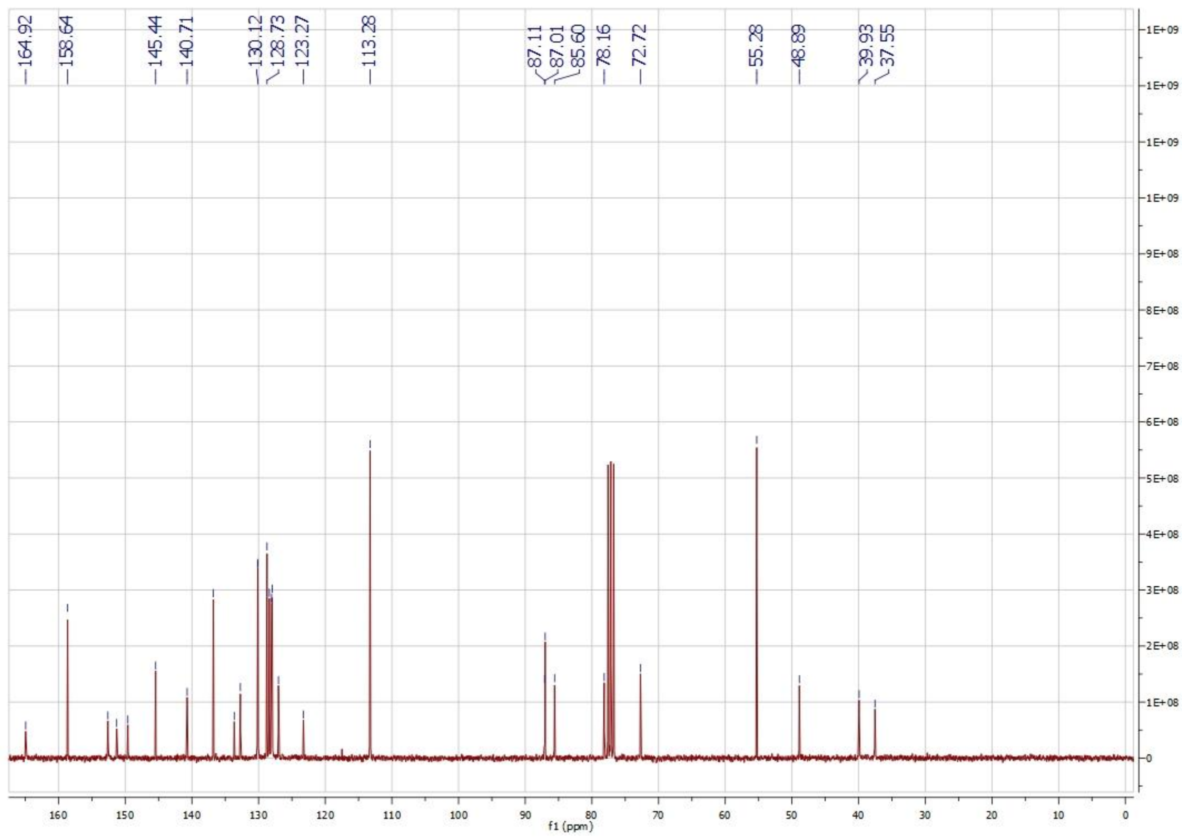

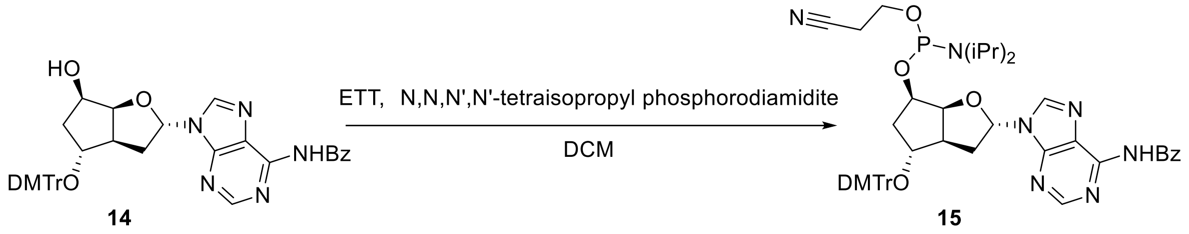

(3'S,5'R,7'R)-N6-Benzoyl-9-{5'-O-[(2-cyanoethoxy)-diisopropylaminophosphanyl]-2',3'-dideoxy-3',5'-ethano-7'-O-[(4,4'-dimethoxytriphenyl)methyl]-α-D-ribofuranosyl} adenine (15) :

To a solution of the nucleoside **14** (199 mg, 0.290 mmol) and 5-(Ethylthio)-1H-tetrazole (57 mg, 0.44 mmol) in dry DCM (7 mL) was added dropwise 2-Cyanoethyl N,N,N′,N′-tetraisopropylphosphordiamidite (0.16 mL, 0.49 mmol) at rt. After stirring for 60 min, the reaction mixture was diluted with satd $NaHCO_3$ (20 mL) and extracted with DCM (3 X 20 mL). The combined organic phases were dried over $MgSO_4$, filtered and evaporated. The crude product was purified by CC (EtOAc, +0.5 % Et_3N) to yield **15** (197 mg, mixture of two isomers, 77%) as a white foam.

Data for **15**: R_f = 0.75 (5% MeOH in DCM);

^{1}H NMR (300 MHz, $CDCl_3$) δ 8.98 (*br*, 1H, N*H*), 8.68, 8.67 (2s, 1H, C(2)), 7.94 (d, *J* = 7.6 Hz, 2H, H-arom), 7.90, 7.84 (2s, 1H, C(8)), 7.56 – 7.49 (m, 1H, H-arom), 7.48 – 7.34 (m, 4H, H-arom), 7.30 – 7.10 (m, 7H, H-arom), 6.80 – 6.69 (m, 4H, Harom), 6.21, 6.15 (2dd, *J* = 6.8, 2.2 Hz, 1H, H-C(1')), 4.69, 4.59 (2dd, *J* = 7.3, 4.5 Hz, 1H, H-C(4')), 4.44 (tt, *J* = 12.3, 6.3 Hz, 1H, H-C(5')), 3.90 (dd, *J* = 9.0, 3.8 Hz, 1H, H-C(5')), 3.82 – 3.63 (m, 8H, *Me*O, OC*H*$_2$CH$_2$CN), 3.59 – 3.43 (m, 2H, (Me$_2$C*H*)$_2$N),

2.61 – 2.49 (m, 2H, OCH_2CH_2CN), 2.47 – 2.07 (m, 2H, H-C(3’), H-C(2’)), 1.98 – 1.66 (m, 3H, H-C(2’), H-C(6’)), 1.15 – 1.03 (m, 12H, $(Me_2CH)_2N$).

^{13}C NMR (101 MHz, $CDCl_3$) δ 164.67 (*C*ONH), 158.77 (MeO-*C*-arom), 152.58 (C(2)), 151.34, 151.29 (C(4)), 149.46 (C(6)), 145.55, 145.54 (C-arom), 141.58, 141.50 (C(8)), 136.87, 136.85, 136.84, 133.85 (C-arom), 132.85, 130.26, 130.23, 130.20, 128.97, 128.47, 128.43, 128.02, 127.96, 127.08 (CH-arom), 123.62, 123.58 (C(5)), 117.91, 117.70 (OCH_2CH_2CN), 113.37 (CH-arom), 87.80, 87.67 (C(1’)), 87.20, 87.14 (*C*(Ph)$_3$), 85.29, 85.22 (($J_{C,P}$ = 4.2, 3.1 Hz, C(4’)), 78.16, 77.96 (C(7’)), 74.28, 73.98 ($J_{C,P}$ = 14.8, 18.4 Hz, C(5’)), 58.80, 58.61 ($J_{C,P}$ = 16.2, 17.3 Hz OCH_2CH_2CN), 55.37, 55.35 (*Me*O-DMTr), 49.02, 48.91 (C(3’)), 43.29, 43.16 ($J_{C,P}$ = 8.9, 9.0 Hz, $((Me_2CH)_2N)$), 39.09 (C(6’)), 37.99, 37.95 (C(2’)), 24.82, 24.77, 24.74, 24.70, 24.64 ($(Me_2CH)_2N$), 20.43, 20.42 ($J_{C,P}$ = 1.4, 1.9 Hz, OCH_2CH_2CN).

^{31}P NMR (121 MHz, $CDCl_3$) δ 148.14, 148.11.

ESI$^+$-HRMS *m/z* calcd for $C_{45}H_{56}O_7N_8P$ ($[M + H]^+$) 884.3895, found 884.3904.

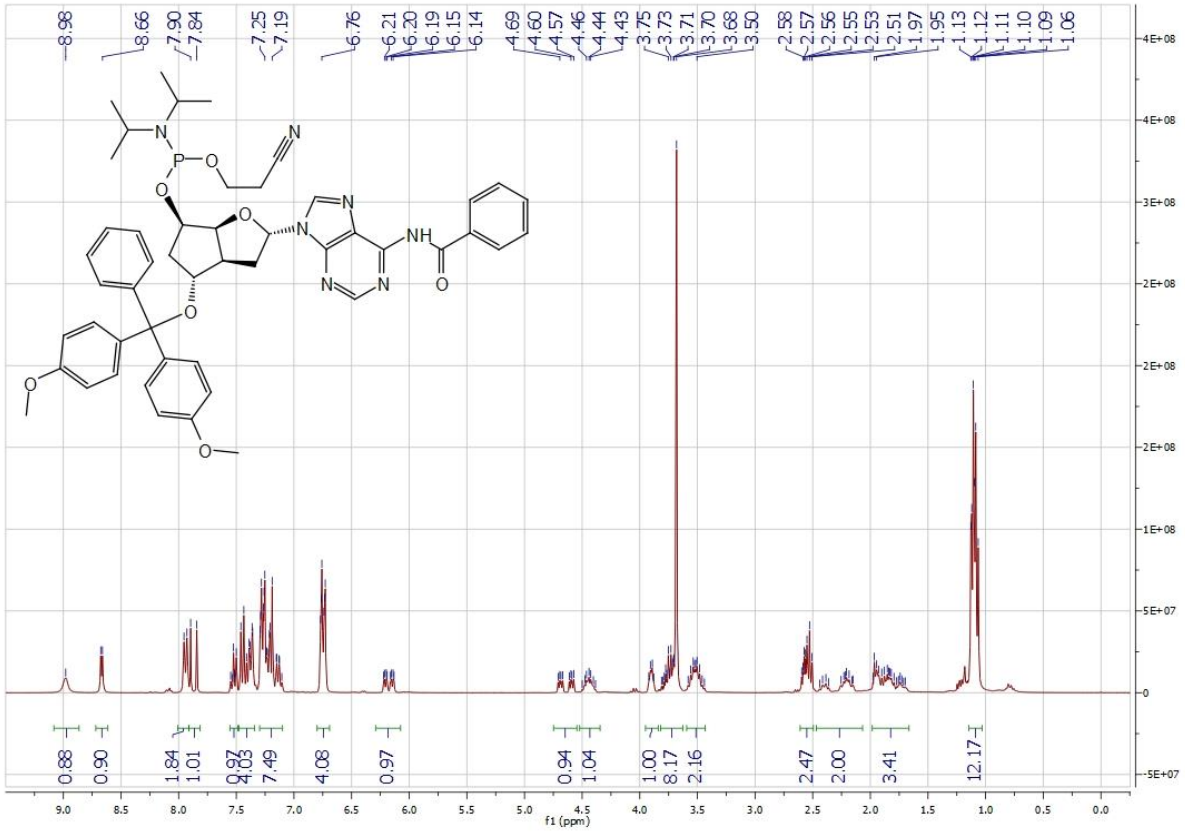

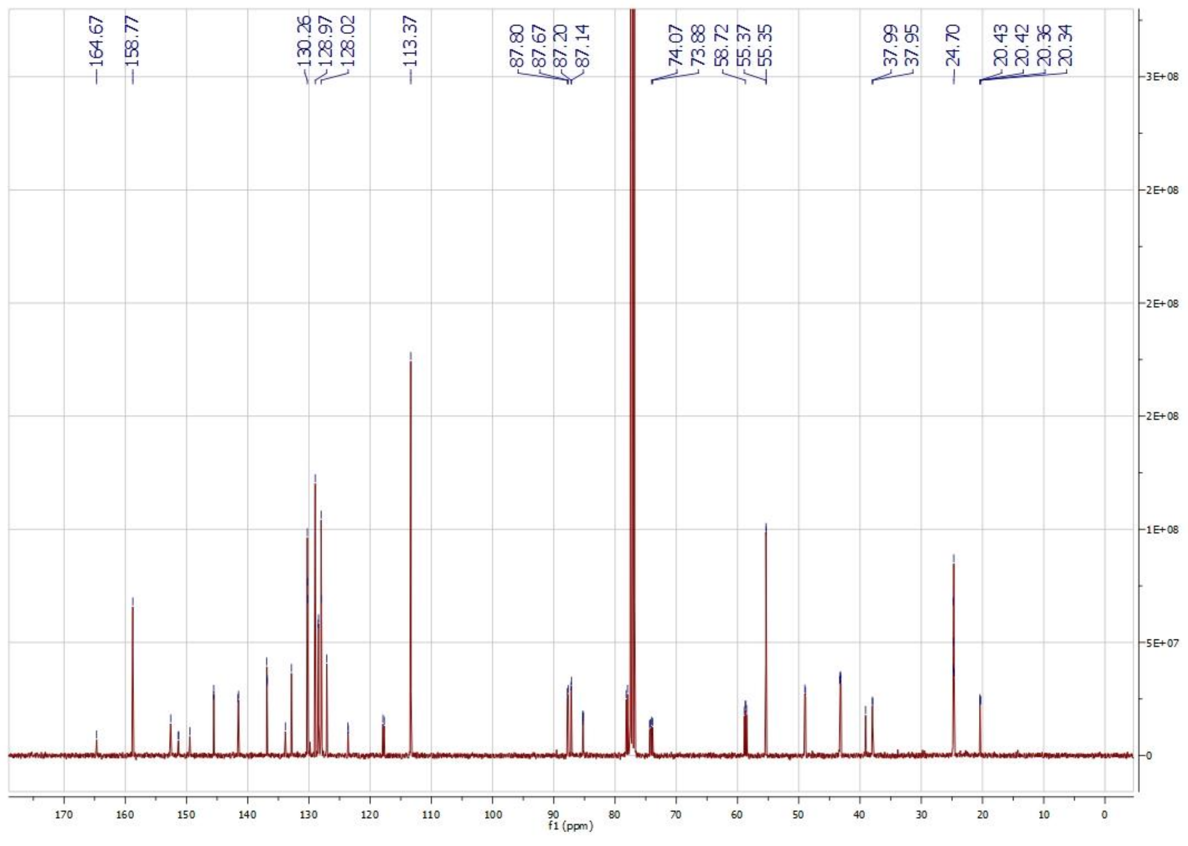
164.67
158.77
130.26
128.97
128.02
113.37
87.80
87.67
87.20
87.14
74.07
73.88
58.72
55.37
55.35
37.99
37.95
24.70
20.43
20.42
20.36
20.34
f1 (ppm)

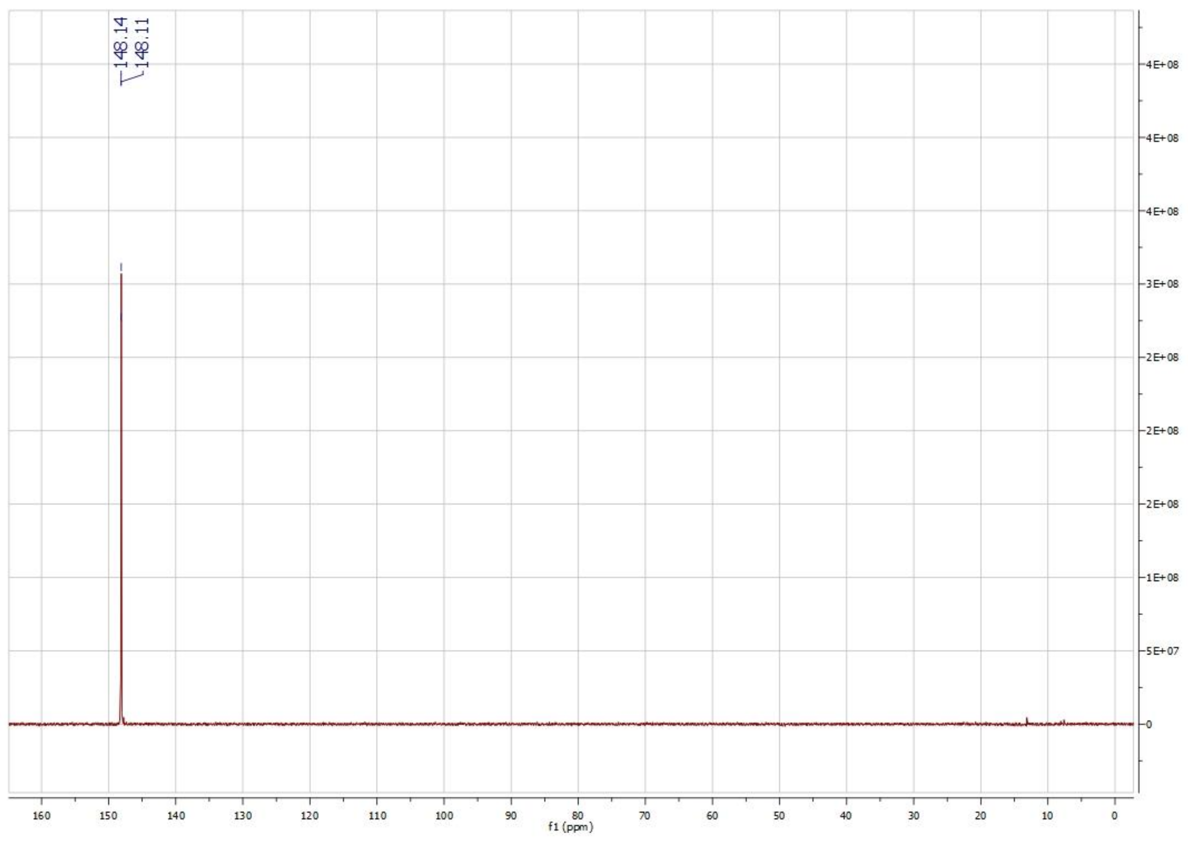
148.14
148.11
f1 (ppm)

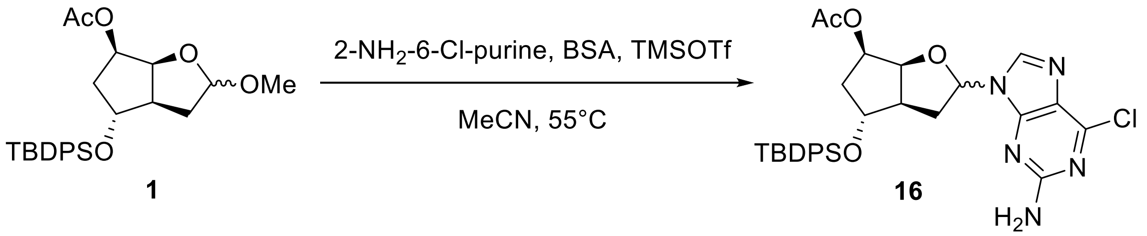

(3'R,5'R,7'R)-2-Amino-6-chloro-9-{5'-O-acetyl-7'-[(tert-butyldiphenylsilyl)oxy]-2',3'-dideoxy-3',5'-ethano-α,β-D-ribofuranosyl} purine (16) :

To a suspension of the sugar **1** (1.75 g, 3.85 mmol) and 2-amino-6-chloropurine (1.05 g, 6.17 mmol) in dry MeCN (20 mL) was added BSA (3.80 mL, 15.4 mmol) at rt. The suspension was heated to 55°C and stirred for 30 min. Then TMSOTf (1.05 mL, 5.78 mmol) was added dropwise and the solution was further stirred for 50 min at 55°C. The solution was cooled down to rt, quenched with addition of satd $NaHCO_3$ (10 mL), diluted EtOAc (50mL) and filtered through a short pad of SiO_2. The SiO_2 was washed with additional EtOAc. The mixture was then washed with satd $NaHCO_3$ (2 x 80 mL), aqueous phases were combined and extracted with EtOAc (3 X 50 mL). The combined organic phases were dried over $MgSO_4$, filtered and evaporated. The crude product was purified by CC (2.5% MeOH in DCM) to yield a mixture of **16** (1.77 g, 77%) in an anomeric ratio α/β ≈ 7:3 as a white foam.

Data for **16**: R_f = 0.54 (EtOAc/hexane 5:1);

^{1}H NMR (300 MHz, $CDCl_3$) δ 7.86 (s, 0.3H, H-C(8)), 7.69 (s, 0.7H, H-C(8)), 7.68 – 7.60 (m, 4H, H-arom), 7.47 – 7.34 (m, 6H, H-arom), 6.04 (dd, *J* = 6.9, 3.0 Hz, 0.7H, H-C(1')), 5.87 (dd, *J* = 8.0, 6.2 Hz, 0.3H, H-C(1')), 5.37 (dt, *J* = 14.2, 4.6 Hz, 1H, H-C(5')), 5.16 (*br*, 2H, N*H₂*), 4.91 (dd, *J* = 6.5, 5.1 Hz, 0.7H, H-C(4')), 4.79 (dd, *J* = 6.9, 5.2 Hz, 0.3H, H-C(4')), 4.13 (*br*, 0.3H, H-C(7')), 4.06 (d, *J* = 4.0 Hz, 0.7H, H-C(7')), 2.95 (dd, *J* = 16.3, 6.6 Hz, 0.7H, H-C(3')), 2.81 (dd, *J* = 17.0, 7.4 Hz, 0.3H, H-C(3')), 2.49 – 2.30 (m, 1H, H-C(2')), 2.14 (dd, *J* = 13.1, 6.7 Hz, 1H, H-C(6')), 2.08 (s, 2.1H, *Me*CO_2), 2.02 (s, 0.9H, *Me*CO_2), 2.02 – 1.91 (m, 1H, H-C(6')), 1.80 (td, *J* = 13.4, 6.8 Hz, 1H, H-C(2')), 1.07, 1.06 (2s, 9H, $(CH_3)_3$-C-Si).

^{13}C NMR (75 MHz, $CDCl_3$) δ 170.55, 170.44 (Me*C*O_2), 158.98, 158.91 (C(2)), 153.18, 152.95 (C(4)), 151.40, 151.34 (C(6)), 140.38, 140.14 (C(8)), 135.73, 135.70 (CH-arom), 133.78, 133.62, 133.24, 133.17 (C-arom), 130.03, 130.00, 127.88, 127.86 (CH-arom), 125.65, 125.57 (C(5)), 86.59, 85.74 (C(1')), 82.93, 80.99 (C(4')), 76.57, 76.14 (C(7')), 74.34, 74.32 (C(5')), 51.15, 51.10 (C(3')), 37.19, 36.99 (C(6')), 36.70, 36.25 (C(2')), 26.87 (($CH_3)_3$-C-Si), 20.95, 20.86 (*Me*CO_2), 19.00 (($CH_3)_3$-*C*-Si).

ESI^+-HRMS *m/z* calcd for $C_{30}H_{35}O_4N_5ClSi$ ($[M + H]^+$) 592.2141, found 592.2158.

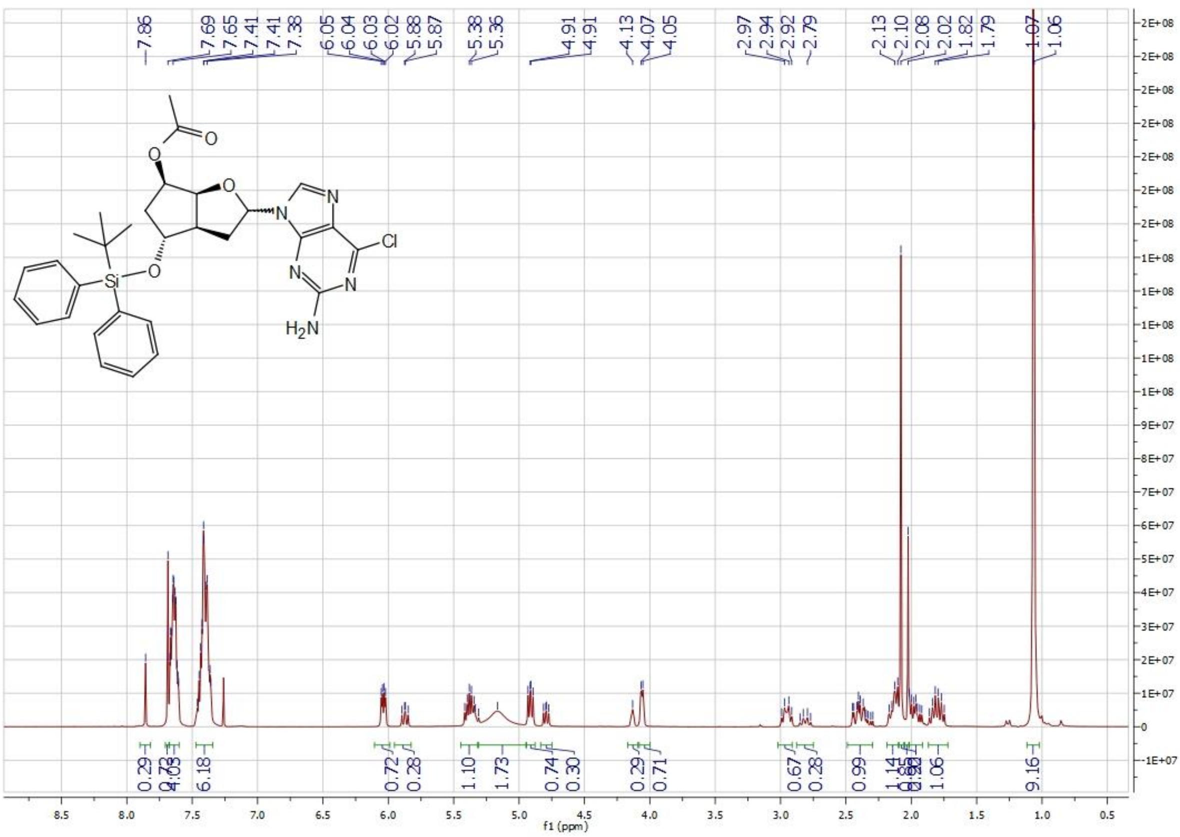
7.86
7.69
7.65
7.41
7.41
7.38
6.05
6.04
6.03
6.02
5.88
5.87
5.38
5.36
4.91
4.91
4.13
4.07
4.05
2.97
2.94
2.92
2.79
2.13
2.10
2.08
2.02
1.82
1.79
1.07
1.06
Cl
N
N
N
N
H2N
O
O
O
O
Si
0.29
4.03
6.18
0.72
0.28
1.10
1.73
0.74
0.30
0.71
0.67
0.28
0.99
1.14
1.06
9.16
f1 (ppm)

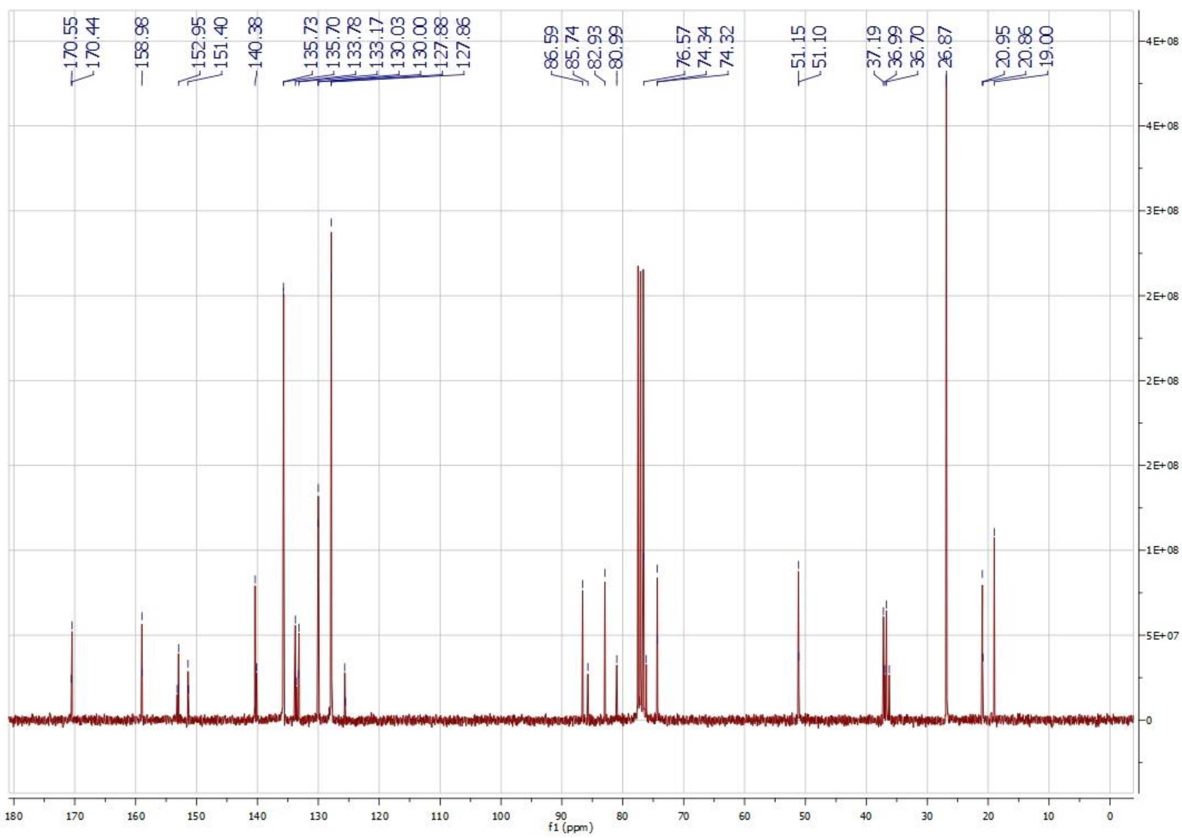
170.55
170.44
158.98
152.95
151.40
140.38
135.73
135.70
133.78
133.17
130.03
130.00
127.88
127.86
86.59
85.74
82.93
80.99
76.57
74.34
74.32
51.15
51.10
37.19
36.99
36.70
26.87
20.95
20.86
19.00
f1 (ppm)

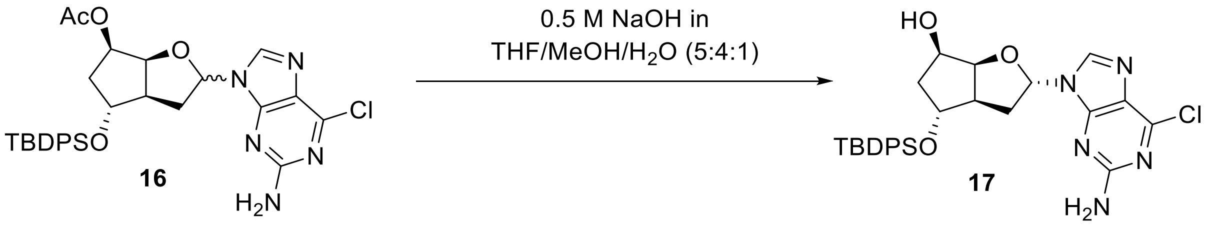

(3'R,5'R,7'R)- 2-Amino-6-chloro-9-{7'-[(tert-butyldiphenylsilyl)oxy]-2',3'-dideoxy-3',5'-ethano-α-D-ribofuranosyl} purine (17) :

The nucleoside **16** (1.78 g, 3.01 mmol) was dissolved in 0.5 M NaOH in THF/methanol/H_2O (5:4:1, 15 mL) at 0°C. The reaction was stirred for 20 min at 0°C and was quenched by addition of NH_4Cl (484 mg). The suspension was then diluted with satd $NaHCO_3$ (100 mL) and extracted with DCM (4 X 75 mL). The combined organic phases were dried over $MgSO_4$, filtered and evaporated. The crude product was purified by CC (3% MeOH in DCM) to yield **17-α** (992 mg, 60%) and **17-β** (428 mg, 25%) as white foams.

Data for **17-α**: R_f = 0.34 (5% MeOH in DCM);

^{1}H NMR (400 MHz, $CDCl_3$) δ 7.71 – 7.60 (m, 5H, H-arom, H-(C(8)), 7.49 – 7.34 (m, 6H, H-arom), 6.08 (dd, *J* = 6.9, 2.6 Hz, 1H, H-C(1')), 5.26 (s, 2H, N*H*2), 4.70 (dd, *J* = 7.5, 4.8 Hz, 1H, H-C(4')), 4.47 (dt, *J* = 10.0, 5.1 Hz, 1H, H-C(5')), 4.11 (t, *J* = 3.3 Hz, 1H, H-C(7')), 2.87 (dd, *J* = 16.5, 7.7 Hz, 1H, H-C(3')), 2.57 (*br*, 1H, O*H*), 2.27 (ddd, *J* = 14.0, 9.9, 2.6 Hz, 1H, H-C(2')), 2.10 – 2.01 (m, 1H, H-C(6')), 1.92 – 1.76 (m, 2H, H-C(2'), H-C(6')), 1.06 (s, 9H, (C*H*3)3-C-Si).

^{13}C NMR (75 MHz, $CDCl_3$) δ 159.09 (C(2)), 153.05 (C(4)), 151.46 (C(6)), 139.91 (C(8)), 135.71 (CH-arom), 133.96, 133.27 (C-arom), 130.00, 129.96, 127.86, 127.83 (CH-arom), 125.52 (C(5)), 86.46 (C(1')), 84.92 (C(4')), 77.40 (C(7')), 72.63 (C(5')), 50.55 (C(3')), 40.92 (C(6')), 36.78 (C(2')), 26.88 ((*C*H3)3-C-Si), 19.01 ((CH3)3-*C*-Si).

ESI$^+$-HRMS *m/z* calcd for $C_{28}H_{33}O_3N_5ClSi$ ([M + H]$^+$) 550.2036, found 550.2019.

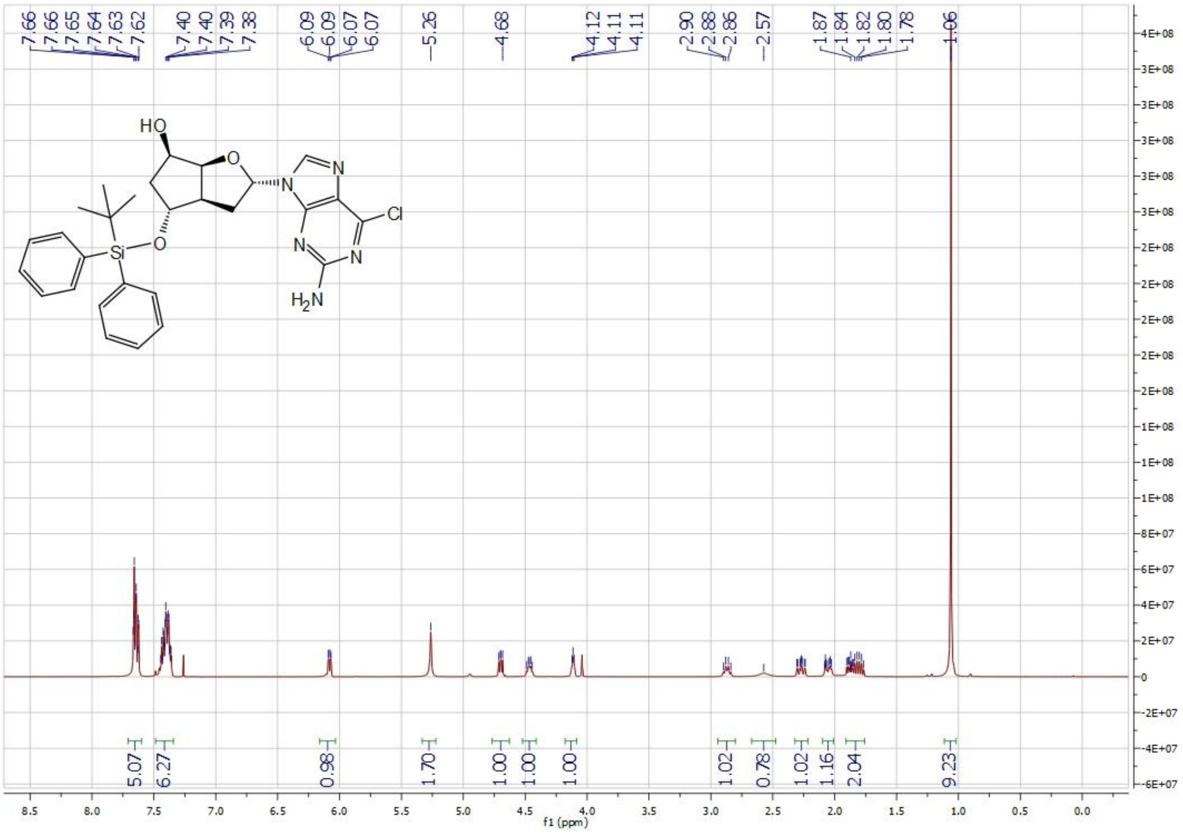
7.66 7.66 7.65 7.64 7.63 7.62
7.40 7.40 7.39 7.38
6.09 6.09 6.07 6.07
5.26
4.68
4.12 4.11 4.11
2.90 2.88 2.86
2.57
1.87 1.84 1.82 1.80 1.78
1.06
HO
O
N
N
N
N
Cl
H2N
Si
O
5.07 6.27 0.98 1.70 1.00 1.00 1.00 1.02 0.78 1.02 1.16 2.04 9.23
f1 (ppm)

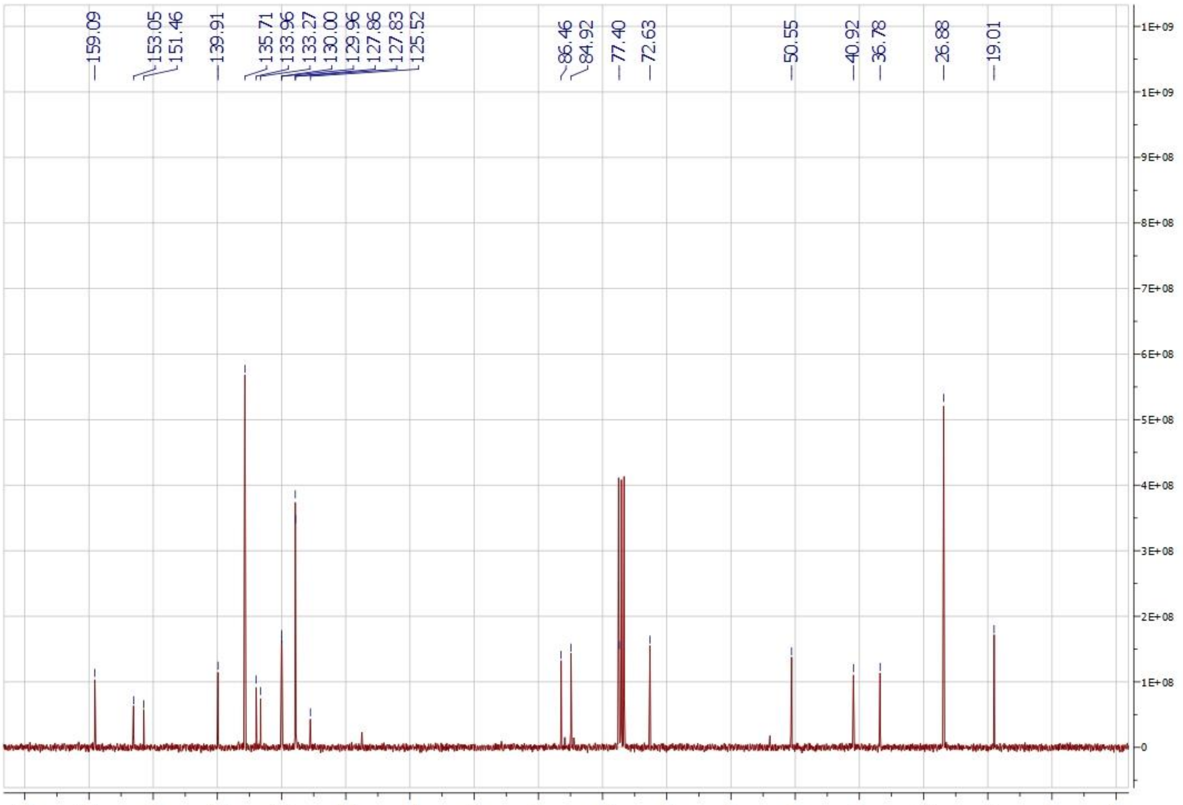
159.09
153.05 151.46
139.91
135.71 133.96 133.27 130.00 129.96 127.86 127.83 125.52
86.46 84.92
77.40
72.63
50.55
40.92
36.78
26.88
19.01
f1 (ppm)

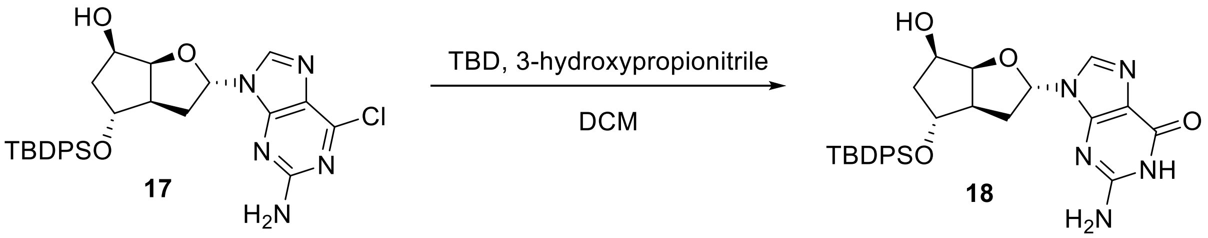

(3'R,5'R,7'R)-9-{7'-[(tert-butyldiphenylsilyl)oxy]-2',3'-dideoxy-3',5'-ethano-α-D-ribofuranosyl} guanine (18) :

To a solution of the nucleoside **17** (610 mg, 1.03 mmol) in dry DCM (15 mL) were added 3-hydroxypropionitrile (0.28 mL, 4.12 mmol) followed by 1,5,7-Triazabicyclo[4.4.0]dec-5-ene (287 mg, 2.06 mmol) at rt. After 4 hours of stirring at rt, a second portion of 3-hydroxypropionitrile (0.28 mL, 3.23 mmol) followed by 1,5,7-Triazabicyclo[4.4.0]dec-5-ene (287 mg, 2.06 mmol) were added. The reaction was further stirred for 2 days and then was directly purified by CC (10% MeOH in DCM) to yield **18** (500 mg, 87%) as white foam.

Data for **18**: R_f = 0.30 (10% MeOH in DCM);

^{1}H NMR (400 MHz, MeOD) δ 7.73 – 7.61 (m, 5H, H-arom, H-C(8)), 7.53 – 7.32 (m, 6H, H-arom), 6.06 (dd, *J* = 6.9, 3.7 Hz, 1H, H-C(1')), 4.74 (dd, *J* = 7.0, 4.6 Hz, 1H, H-C(4')), 4.46 – 4.36 (m, 1H, H-C(5')), 4.11 (*br*, 1H, H-C(7')), 2.91 (dd, *J* = 16.2, 6.6 Hz, 1H, H-C(3')), 2.31 (ddd, *J* = 13.8, 10.0, 3.7 Hz, 1H, H-C(2')), 1.98 – 1.78 (m, 3H, H-C(2'), H-C(3')), 1.07 (s, 9H, $(CH_3)_3$-C-Si).

^{13}C NMR (101 MHz, MeOD) δ 159.30 (C(2)), 155.14 (C(6)), 152.38 (C(4)), 137.28 (C(8)), 136.93, 136.88 (CH-arom), 135.13, 134.78 (C-arom), 131.07, 131.06, 128.91, 128.89 (CH-arom), 117.98 (C(5)), 87.72 (C(1')), 86.25 (C(4')), 79.21, (C(7')) 73.87 (C(5')), 52.13 (C(3')), 41.44 (C(6')), 38.35 (C(2')), 27.42 ((CH_3)$_3$-C-Si), 19.82 ((CH_3)$_3$-*C*-Si)).

ESI$^+$-HRMS *m/z* calcd for $C_{28}H_{34}O_4N_5Si$ ([M + H]$^+$) 532.2386, found 532.2367.

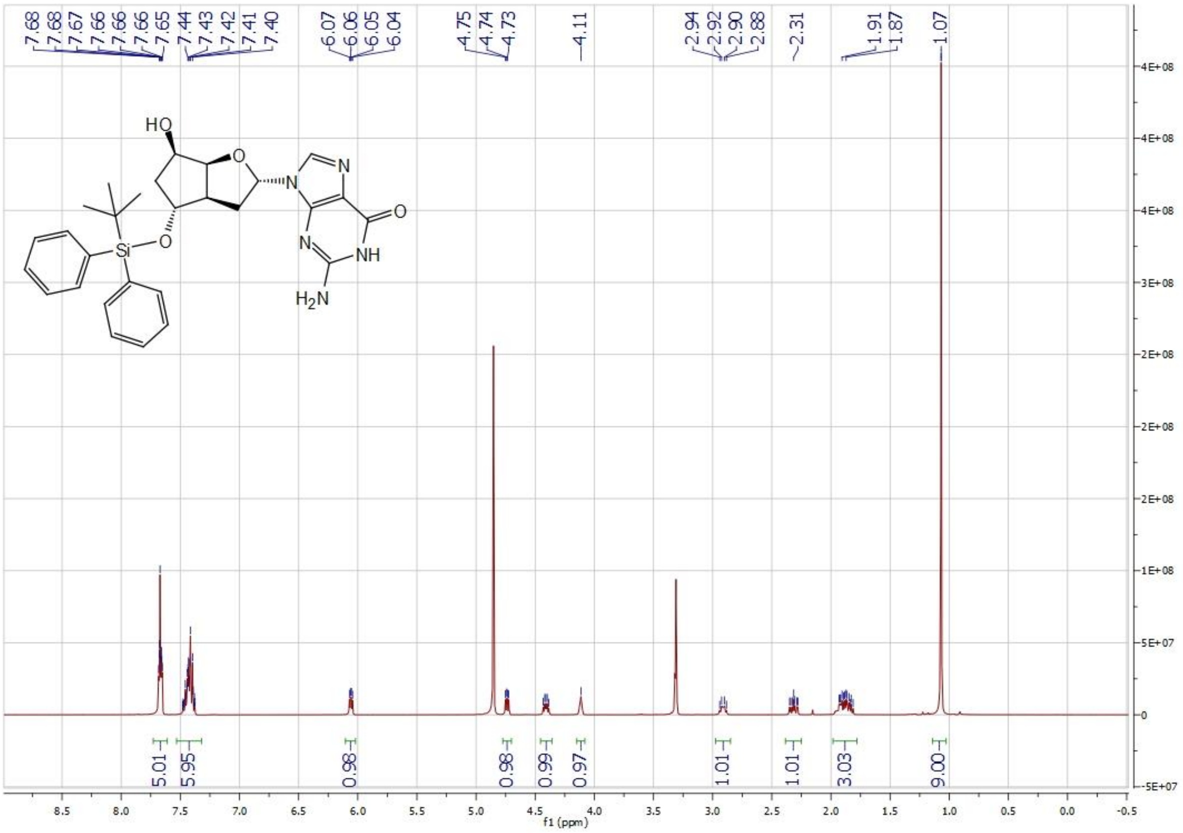
7.68 7.68 7.67 7.66 7.66 7.66 7.65 7.44 7.43 7.42 7.41 7.40
6.07 6.06 6.05 6.04
4.75 4.74 4.73
4.11
2.94 2.92 2.90 2.88
2.31
1.91 1.87
1.07
HO
O
N
N
O
NH
N
N
H_2N
Si
O
5.01 5.95 0.98 0.98 0.99 0.97 1.01 1.01 3.03 9.00
f1 (ppm)

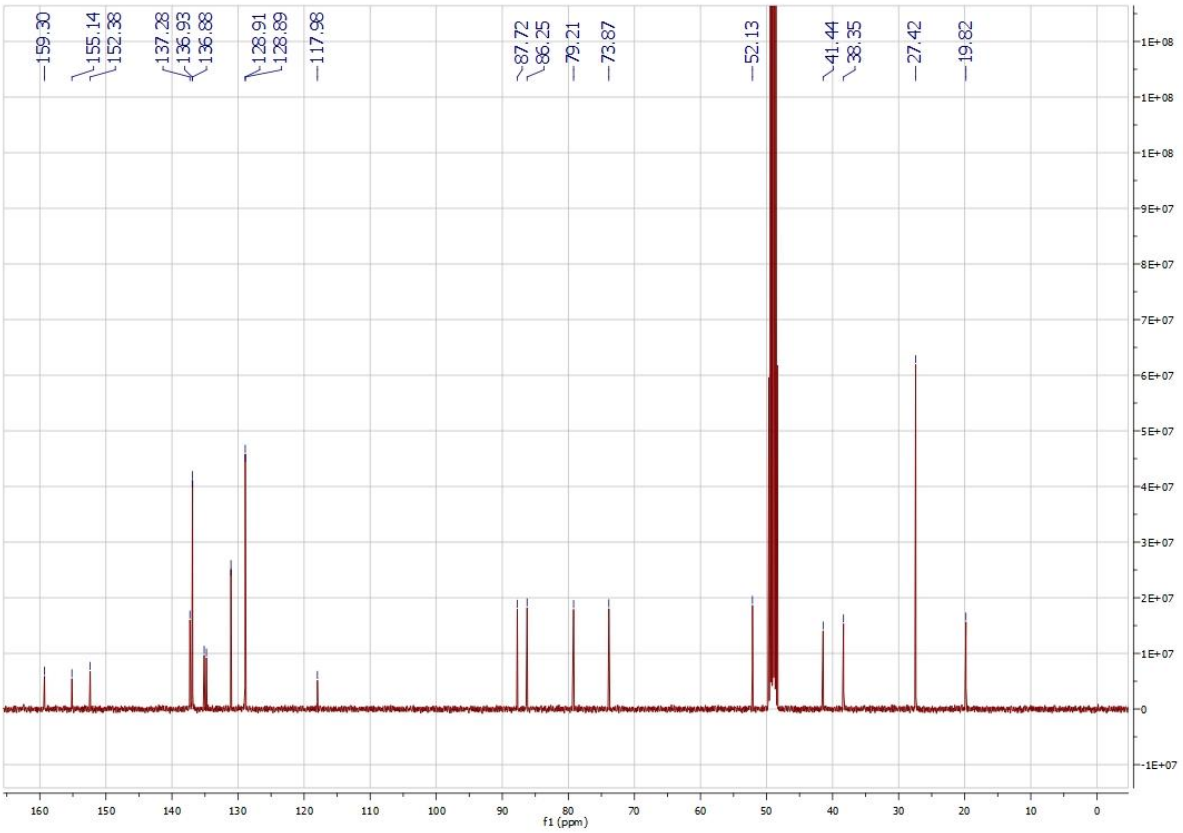
159.30
155.14 152.38
137.28 136.93 136.88
128.91 128.89
117.98
87.72 86.25
79.21
73.87
52.13
41.44 38.35
27.42
19.82
f1 (ppm)

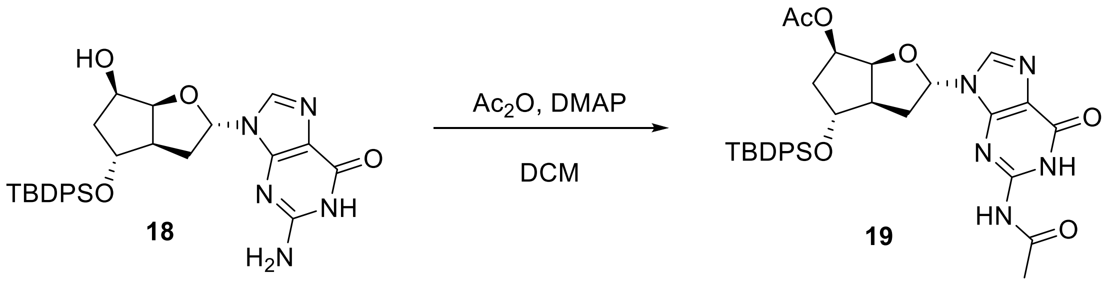

(3’R,5’R,7’R)- N2-Acetyl-9-{5’-O-acetyl-7’-[(tert-butyldiphenylsilyl)oxy]-2’,3’-dideoxy-3’,5’-ethano-α-D-ribofuranosyl} guanine (19) :

To a solution of nucleoside **18** (500 mg, 0.940 mmol) and 4-Dimethylaminopyridine (276 mg, 2.4 mmol) in dry DCM (15 mL) was added acetic anhydride (1.0 mL, 10.3 mmol) at rt. After stirring for 2 days, reaction was quenched by addition of satd $NaHCO_3$ (30 mL). The mixture was then extracted with DCM (3 X 30 mL). The combined organic phases were dried over $MgSO_4$, filtered and evaporated. The crude product was purified by CC (3.5% MeOH in DCM) to yield **19** (441 mg, 76%) as white foam.

Data for **19**: R_f = 0.62 (10% MeOH in DCM);

^{1}H NMR (300 MHz, $CDCl_3$) δ 12.11 (*br*, 1H, N*H*-C(4)), 9.94 (*br*, 1H, H-N(1)), 7.62 (d, *J* = 6.7 Hz, 5H, H-arom, H-C(8)), 7.46 – 7.31 (m, 6H, H-arom), 6.03 (dd, *J* = 6.7, 2.7 Hz, 1H, H-C(1’)), 5.31 (dt, *J* = 10.3, 5.2 Hz, 1H, H-(C5’)), 4.99 – 4.81 (m, 1H, H-C(4’)), 4.02 (d, *J* = 3.8 Hz, 1H, H-C(7’)), 2.88 (dd, *J* = 16.0, 6.6 Hz, 1H, H-C(3’)), 2.44 – 2.20 (m, 4H, *Me*CONH, H-C(2’)), 2.12 – 1.73 (m, 6H, *Me*CO_2, H-C(6’), H-C(2’)), 1.04 (s, 9H, $(CH_3)_3$-C-Si).

^{13}C NMR (75 MHz, $CDCl_3$) δ 172.73 (Me*C*ONH), 170.46 (Me*C*O_2), 155.87 (C(6)), 148.09 (C(4)), 147.47 (C(2)), 137.13 (C(8)), 135.74 (CH-arom), 133.62, 133.29 (C-arom), 130.13, 130.09, 127.96, 127.93 (CH-arom), 121.54 (C(5)), 86.47 (C(1’)), 82.81 (C(4’)), 76.60 (C(7’)), 74.37 (C(5’)), 51.23 (C(3’)), 37.04, 37.01, (C(2’), C(6’)) 26.92 (($CH_3)_3$-C-Si), 24.46 (*Me*CONH), 21.00 (*Me*CO_2), 19.05 ($(CH_3)_3$-*C*-Si).

ESI$^+$-HRMS *m/z* calcd for $C_{32}H_{38}O_6N_5Si$ ($[M + H]^+$) 616.2586, found 616.2580.

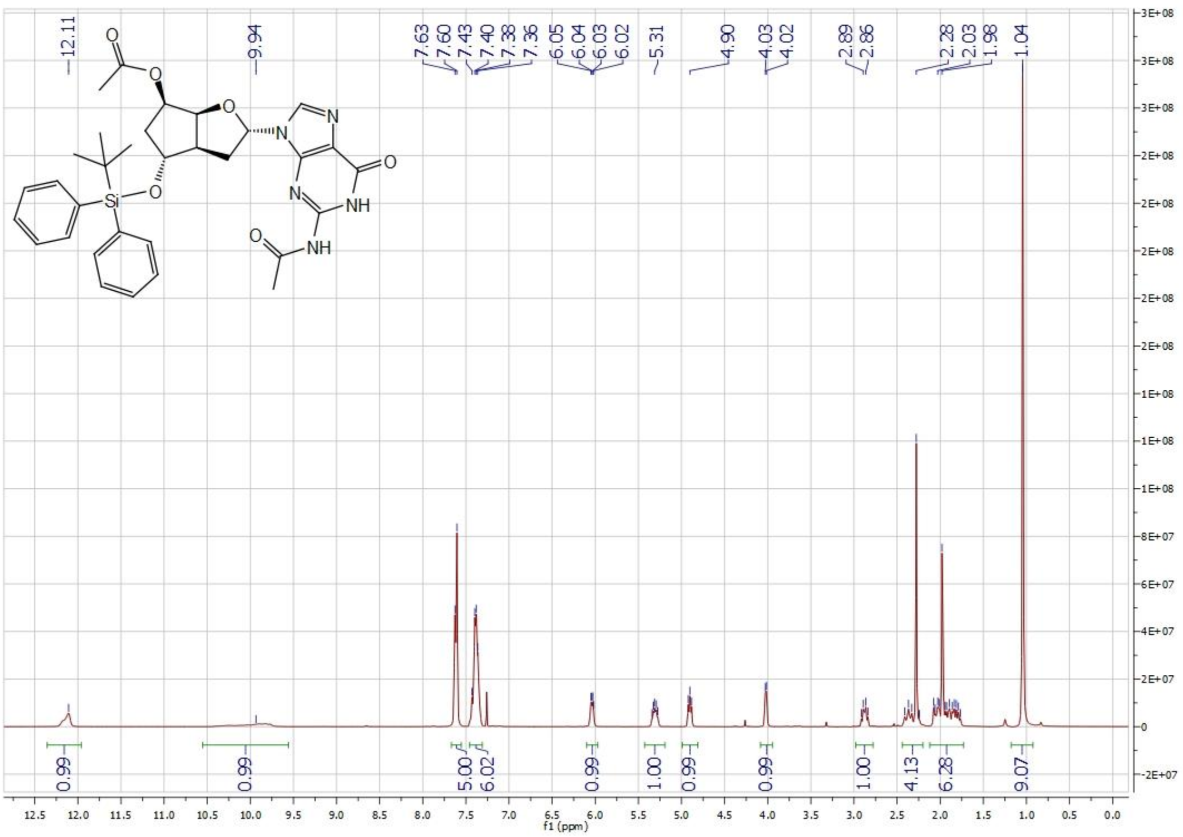
12.11
9.94
7.63
7.60
7.43
7.40
7.38
7.36
6.05
6.04
6.03
6.02
5.31
4.90
4.03
4.02
2.89
2.86
2.28
2.03
1.98
1.04
0.99
0.99
5.00
6.02
0.99
1.00
0.99
0.99
1.00
4.13
6.28
9.07
f1 (ppm)
O
Si
N
NH
NH

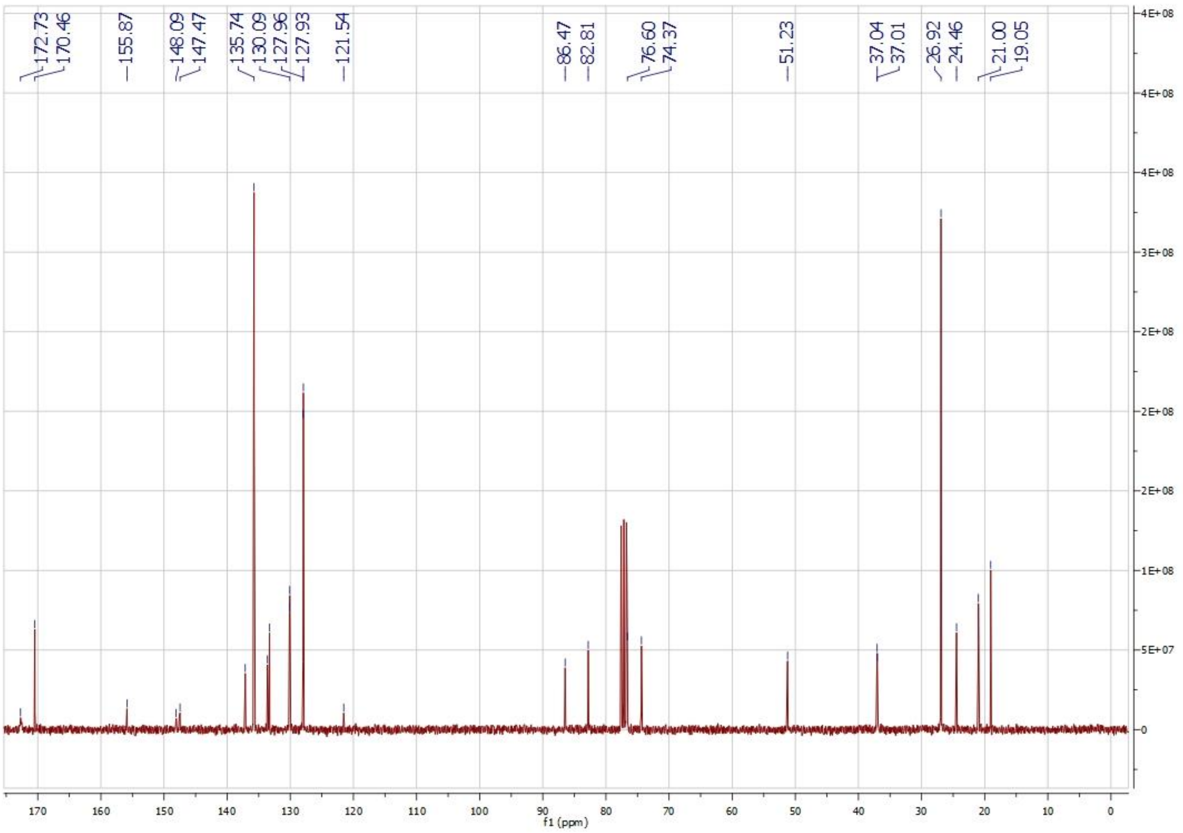
172.73
170.46
155.87
148.09
147.47
135.74
130.09
127.96
127.93
121.54
86.47
82.81
76.60
74.37
51.23
37.04
37.01
26.92
24.46
21.00
19.05
f1 (ppm)

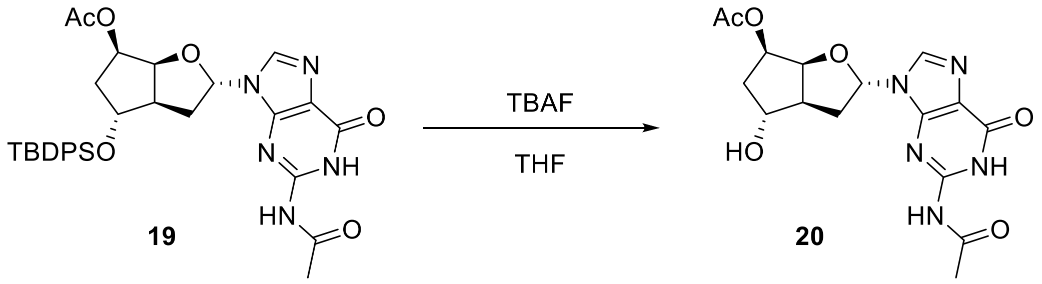

(3’S,5’R,7’R)- N2-Acetyl-9-{5’-O-acetyl-2’,3’-dideoxy-3’,5’-ethano-7’-hydroxy-α-D-ribofuranosyl} guanine (20) :

To a solution of nucleoside **19** (440 mg, 0.714 mmol) in dry THF (5 mL) was added TBAF (1M in THF, 1.1 mL, 1.1 mmol) at rt. The solution was stirred for 4 hours at rt and then was directly purified by CC (13% MeOH in DCM) to yield **20** (235 mg, 87%) as white foam. Crystals suitable for X-ray analysis were obtained by recrystallization in a mixture of H_2O/MeOH.

Data for **20**: R_f = 0.25 (13% MeOH in DCM);

^{1}H NMR (300 MHz, MeOD) δ 8.03 (s, 1H, H-C(8)), 6.28 (dd, *J* = 7.0, 3.8 Hz, 1H, H-C(1’)), 5.21 (ddd, *J* = 9.2, 6.8, 5.1 Hz, 1H, H-C(5’)), 4.98 (dd, *J* = 6.7, 5.0 Hz, 1H, H-(4’)), 4.13 – 4.05 (m, 1H, H-C(7’)), 3.17 – 3.05 (m, 1H, H-C(3’)), 2.86 (ddd, *J* = 13.8, 10.0, 3.8 Hz, 1H, H-C(2’)), 2.39 – 2.27 (m, 1H, H-C(2’)), 2.24 (s, 3H, *Me*CONH), 2.16 – 2.00 (m, 5H, *Me*CO_2, H-C(6’)).

^{13}C NMR (101 MHz, MeOD) δ 174.95 (Me*C*ONH), 172.32 (Me*C*O_2), 157.50 (C(6)), 149.96 (C(4)), 149.38 (C(2)), 139.66 (C(8)), 121.76 (C(5)), 88.23 (C(1’)), 84.23 (C(4’)), 75.83 (C(5’), C(7’)), 51.65 (C(3’)), 38.04, 37.93 (C(2’), C(6’)), 23.83 (*Me*CONH), 20.71 (*Me*CO_2).

ESI$^+$-HRMS *m/z* calcd for $C_{16}H_{20}O_6N_5$ ($[M + H]^+$) 378.1408, found 378.1419.

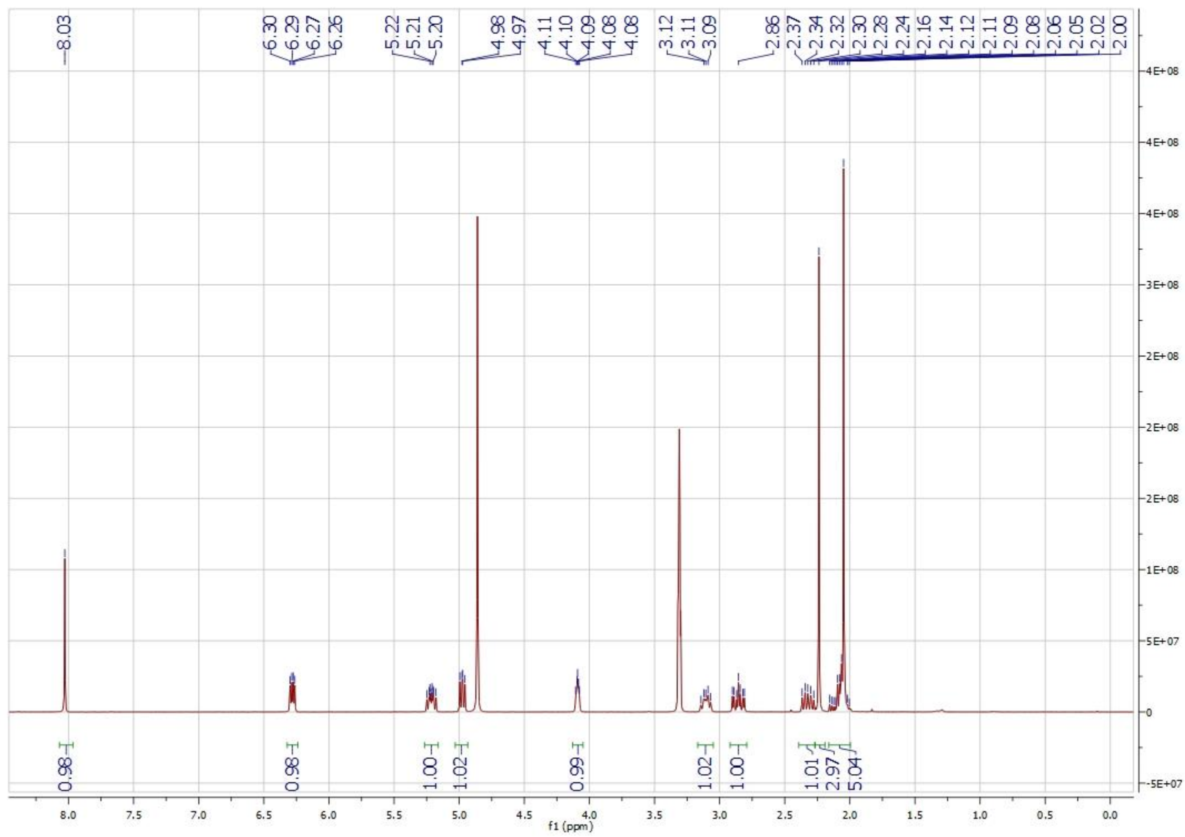
8.03
6.30
6.29
6.27
6.26
5.22
5.21
5.20
4.98
4.97
4.11
4.10
4.09
4.08
4.08
3.12
3.11
3.09
2.86
2.37
2.34
2.32
2.30
2.28
2.24
2.16
2.14
2.12
2.11
2.09
2.08
2.06
2.05
2.02
2.00
0.98
0.98
1.00
1.02
0.99
1.02
1.00
1.01
2.97
5.04
f1 (ppm)

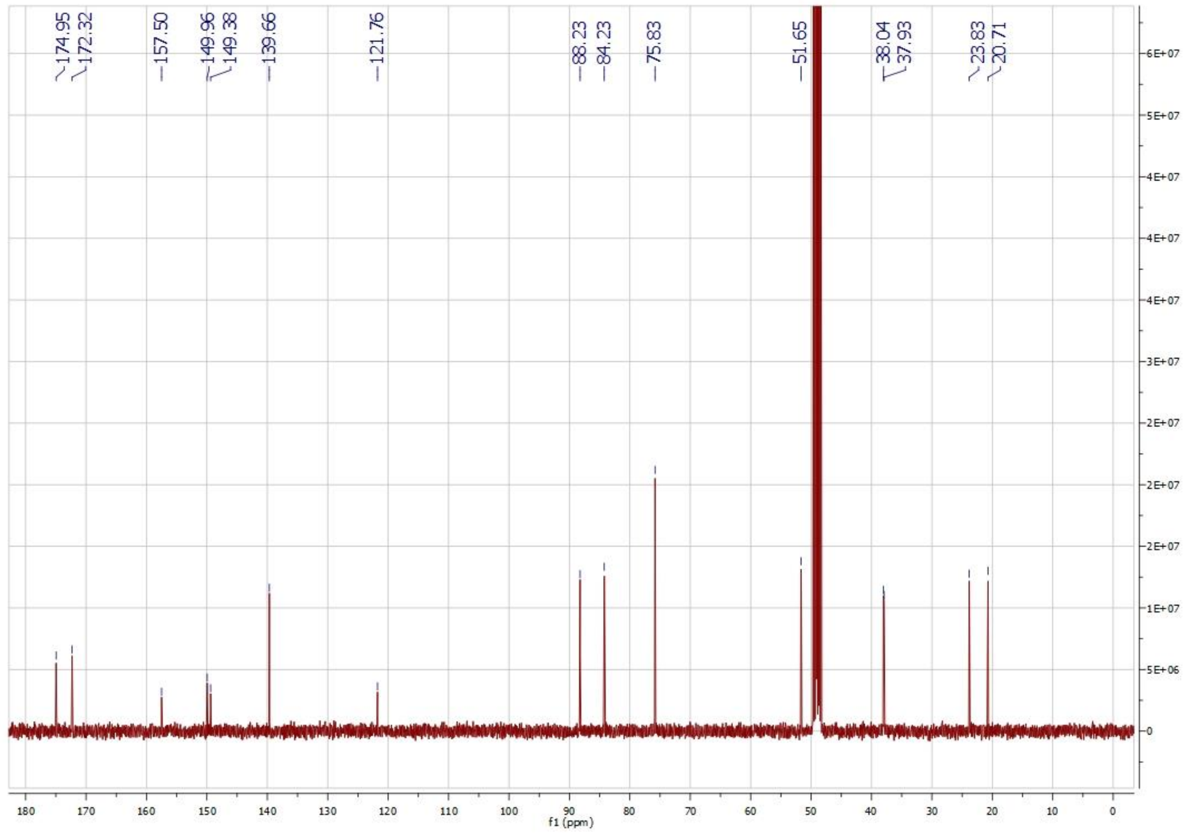
174.95
172.32
157.50
149.96
149.38
139.66
121.76
88.23
84.23
75.83
51.65
38.04
37.93
23.83
20.71
f1 (ppm)

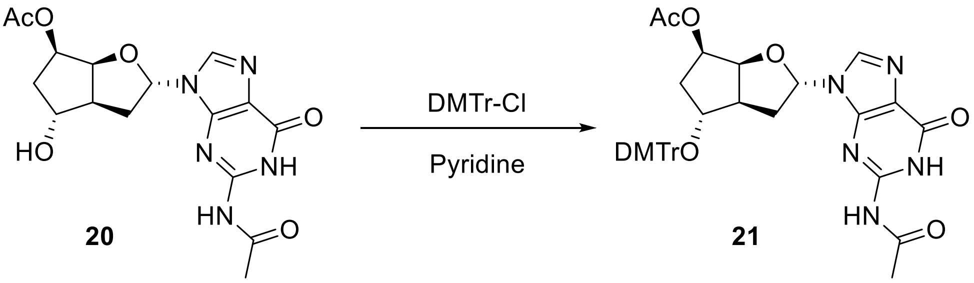

(3'S,5'R,7'R)- N2-Acetyl-9-{5'-O-acetyl-2',3'-dideoxy-3',5'-ethano-7'- O-[(4,4'-dimethoxytriphenyl)methyl]-α-D-ribofuranosyl} guanine (21) :

To a solution of the nucleoside **20** (186 mg, 0.492 mmol) in dry pyridine (10 mL) was added DMTr-Cl (501 mg, 1.48 mmol) at rt. The solution was stirred for 2 days and then was diluted with satd $NaHCO_3$ (40 mL) and extracted with DCM (3 X 30 mL). The combined organic phases were dried over $MgSO_4$, filtered and evaporated. The crude product was purified by CC (3% MeOH in DCM, +0.5 % Et_3N) to yield **21** (333 mg, 99%) as a yellow foam.

Data for **21**: R_f = 0.56 (10% MeOH in DCM);

^{1}H NMR (300 MHz, $CDCl_3$) δ 12.05 (*br*, 1H, N*H*-C(4)), 9.90 (*br*, 1H, H-N(1)), 7.40 (s, 1H, H-C(8)), 7.38 – 7.31 (m, 2H, H-arom), 7.28 – 7.08 (m, 7H, H-arom), 6.75 (dd, *J* = 9.0, 2.7 Hz, 4H, H-arom), 5.95 – 5.85 (m, 1H, H-C(1')), 5.30 – 5.10 (m, 1H, H-C(5')), 4.70 – 4.58 (m, 1H, H-C(4')), 3.81 (*br*, 1H, H-C(7')), 3.68, 3.68 (2s, 6H, *Me*O), 2.25 – 2.07 (m, 5H, *Me*CONH, H-C(3'), H-C(2')), 1.96 – 1.79 (m, 5H, $MeCO_2$, H-C(2'), H-C(6')), 1.74 – 1.59 (m, 1H, H-C(6')).

^{13}C NMR (75 MHz, $CDCl_3$) δ 172.65 (Me*C*ONH), 170.42 (Me*C*O_2), 158.73, 158.70 (MeO-*C*-arom), 155.86 (C(6)), 147.96 (C(4)), 147.43 (C(2)), 145.31 (C-arom), 137.17 (C(8)), 136.69, 136.44 (C-arom), 130.32, 130.21, 128.29, 128.05, 127.09 (CH-arom), 121.53 (C(5)), 113.38, 113.35 (CH-arom), 87.25 (*C*$(Ph)_3$), 86.73 (C(1')), 82.77 (C(4')), 77.19 (C(7')), 74.37 (C(5')), 55.38 (*Me*O-DMTr), 49.28 (C(3')), 37.25 (C(2')), 36.06 (C(6')), 24.40 (*Me*CONH), 21.01 (*Me*CO_2).

ESI$^+$-HRMS *m/z* calcd for $C_{37}H_{38}O_8N_5$ ($[M + H]^+$) 680.2715, found 680.2718

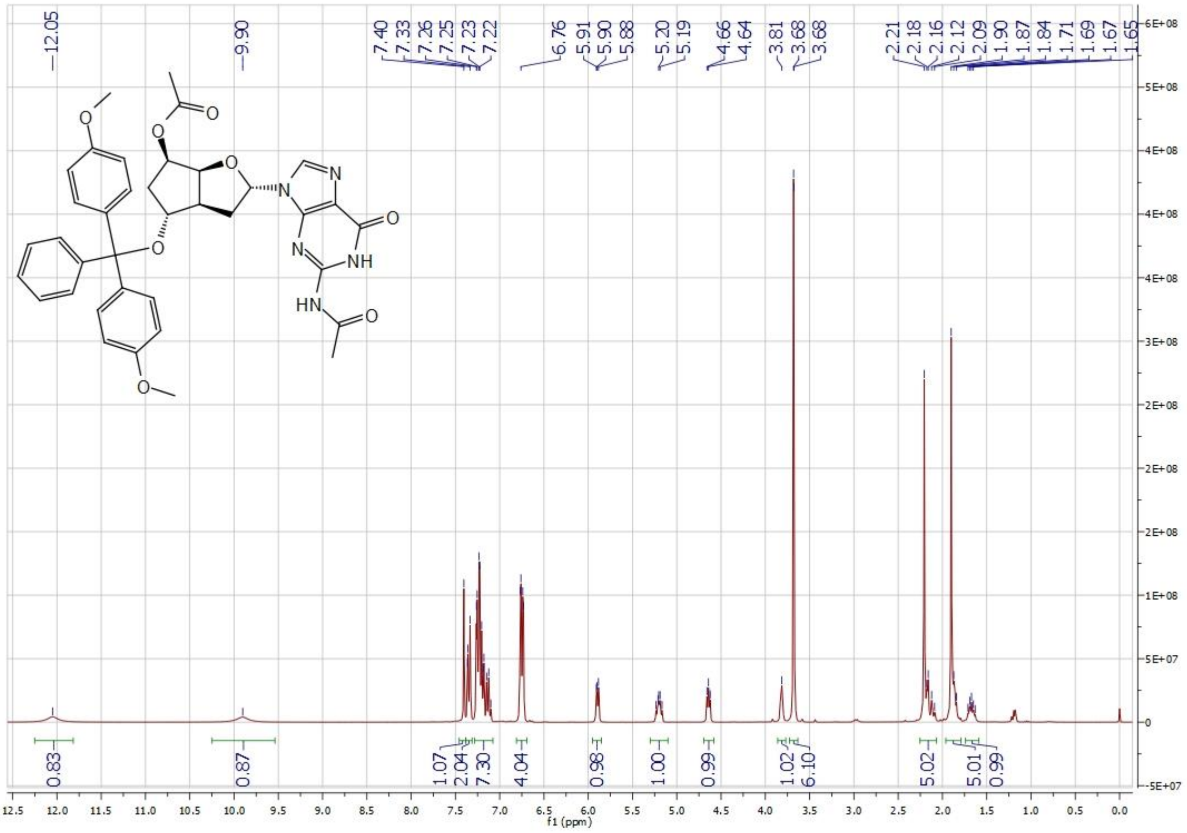

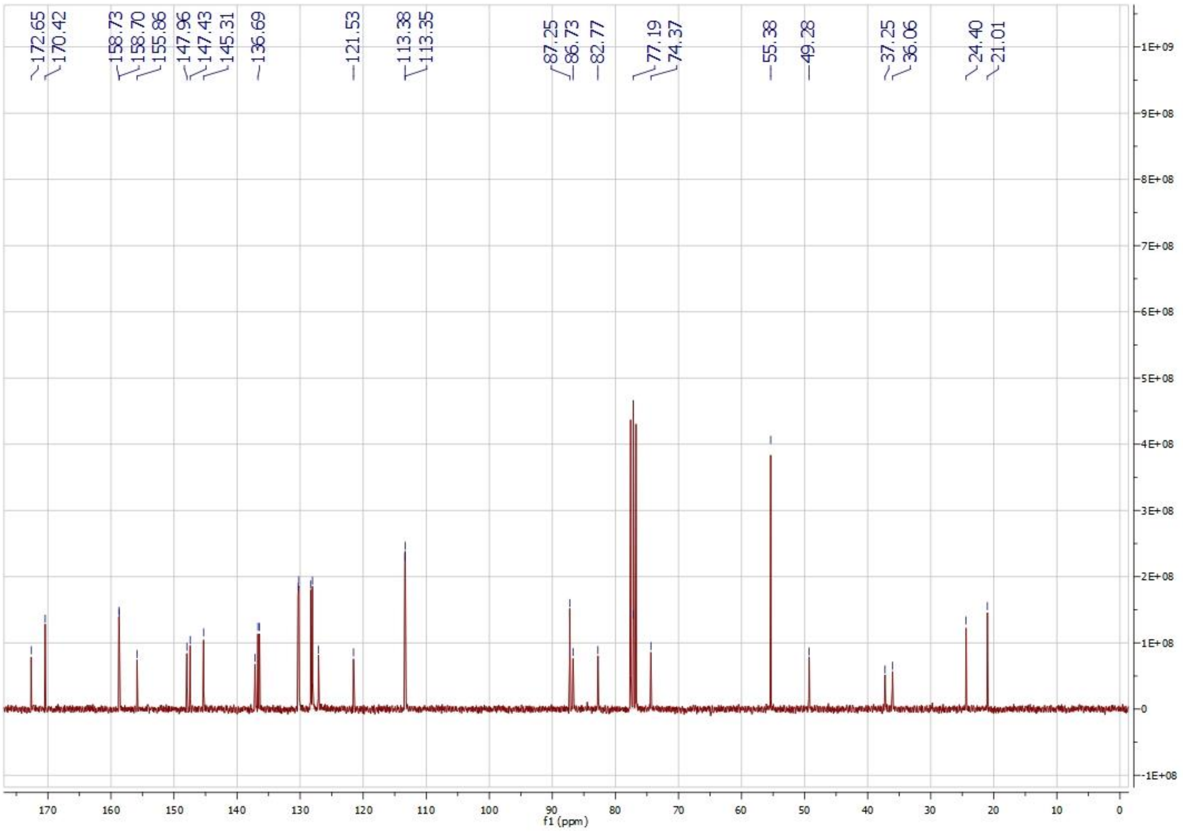

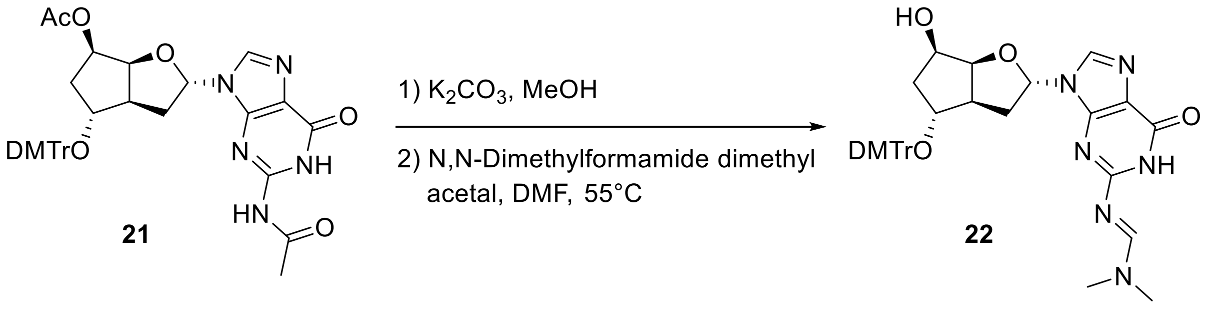

(3'S,5'R,7'R)-N2-(N,N-Dimethylformamidino)-9-{2',3'-dideoxy-3',5'-ethano-7'-O-[(4,4'-dimethoxytriphenyl)methyl]-α-D-ribofuranosyl} guanine (22) :

To a solution of the nucleoside **21** (333 mg, 0.490 mmol) in dry MeOH (10 mL) was added K_2CO_3 (305 mg, 2.20 mmol) at rt. The suspension was stirred for 7 h at rt, then NH_4Cl (78 mg, 1.46 mmol) was added and the resulting mixture was filtered through a short pad of SiO_2. The SiO_2 was washed with additional MeOH and then solvent was evaporated.

The crude product was dissolved in dry DMF (10 mL) and N,N-Dimethylformamide dimethyl acetal (0.33 mL, 2.5 mmol) was added. The solution was stirred for 2 hours at 55° C and then the solvents were removed under reduced pressure. The crude product was purified by CC (7% MeOH in DCM, +0.5 % Et_3N) to yield **22** (245 mg, 77%) as white foam containing traces of Et_3N.

Data for **22**: R_f = 0.32 (12% MeOH in DCM);

^{1}H NMR (300 MHz, $CDCl_3$) δ 9.75 (*br*, 1H, H-N(1)), 8.25 (s, 1H, NC*H*N(CH_3)$_2$), 7.37 (d, *J* = 7.3 Hz, 2H, H-arom), 7.29 – 7.08 (m, 8H, H-arom, H-C(8)), 6.74 (d, *J* = 8.1 Hz, 4H, H-arom), 6.03 (dd, *J* = 6.7, 2.8 Hz, 1H, H-C(1')), 4.57 (dd, *J* = 7.5, 4.6 Hz, 1H, H-C(4')), 4.37 – 4.26 (m, 1H, H-C(5')), 3.89 (t, *J* = 3.9 Hz, 1H, H-C(7')), 3.67, 3.67 (2s, 6H, *Me*O), 3.24 (*br*, 1H, O*H*), 2.94 (s, 3H, NCHN(CH_3)$_2$), 2.87 (s, 3H, NCHN(CH_3)$_2$), 2.35 (dd, *J* = 15.9, 7.6 Hz, 1H, H-C(3')), 1.94 – 1.68 (m, 4H, H-C(2'), H-C(6')).

^{13}C NMR (75 MHz, $CDCl_3$) δ 158.61 (MeO-*C*-arom), 158.28 (C(2)), 157.92 (N*C*HN(CH_3)$_2$), 156.69 (C(6)), 149.90 (C(4)), 145.52, 136.86, 136.77 (C-arom), 135.50 (C(8)), 130.15, 128.32, 127.92, 126.95 (CH-arom), 120.27 (C(5)), 113.24 (CH-arom), 86.92 (*C*(Ph)$_3$), 85.57 (C(1')), 85.12 (C(4')), 78.31 (C(7')), 72.69 (C(5')), 55.28 (*Me*O-DMTr), 49.28 (C(3')), 41.38 (NCHN(*C*H_3)$_2$), 39.77 (C(6')), 37.58 (C(2')), 35.04 (NCHN(*C*H_3)$_2$).

ESI$^+$-HRMS *m/z* calcd for $C_{36}H_{39}O_6N_6$ ([M + H]$^+$) 651.2926, found 651.2921.

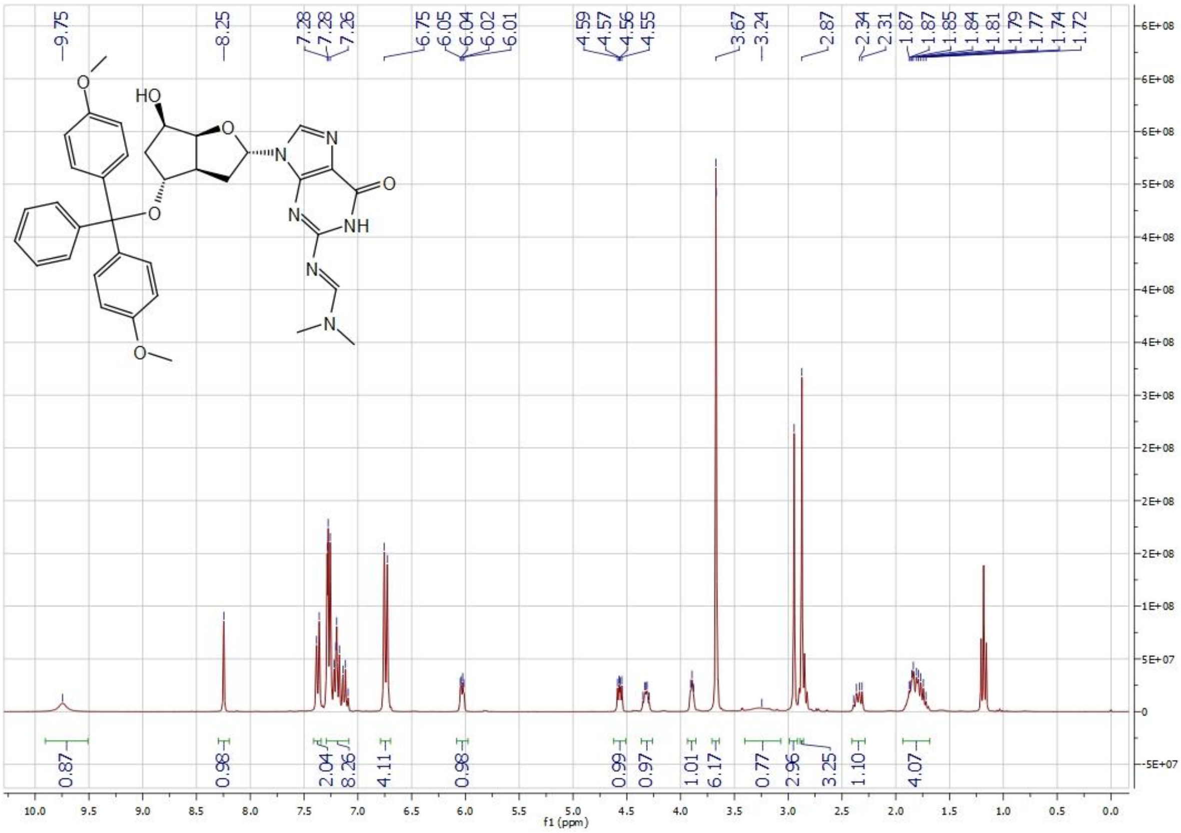

9.75
8.25
7.28
7.28
7.26
6.75
6.05
6.04
6.02
6.01
4.59
4.57
4.56
4.55
3.67
3.24
2.87
2.34
2.31
1.87
1.87
1.85
1.84
1.81
1.79
1.77
1.74
1.72
HO
O
N
N
O
N
NH
N
N
O
O
O
0.87
0.98
2.04
8.26
4.11
0.98
0.99
0.97
1.01
6.17
0.77
2.96
3.25
1.10
4.07
f1 (ppm)

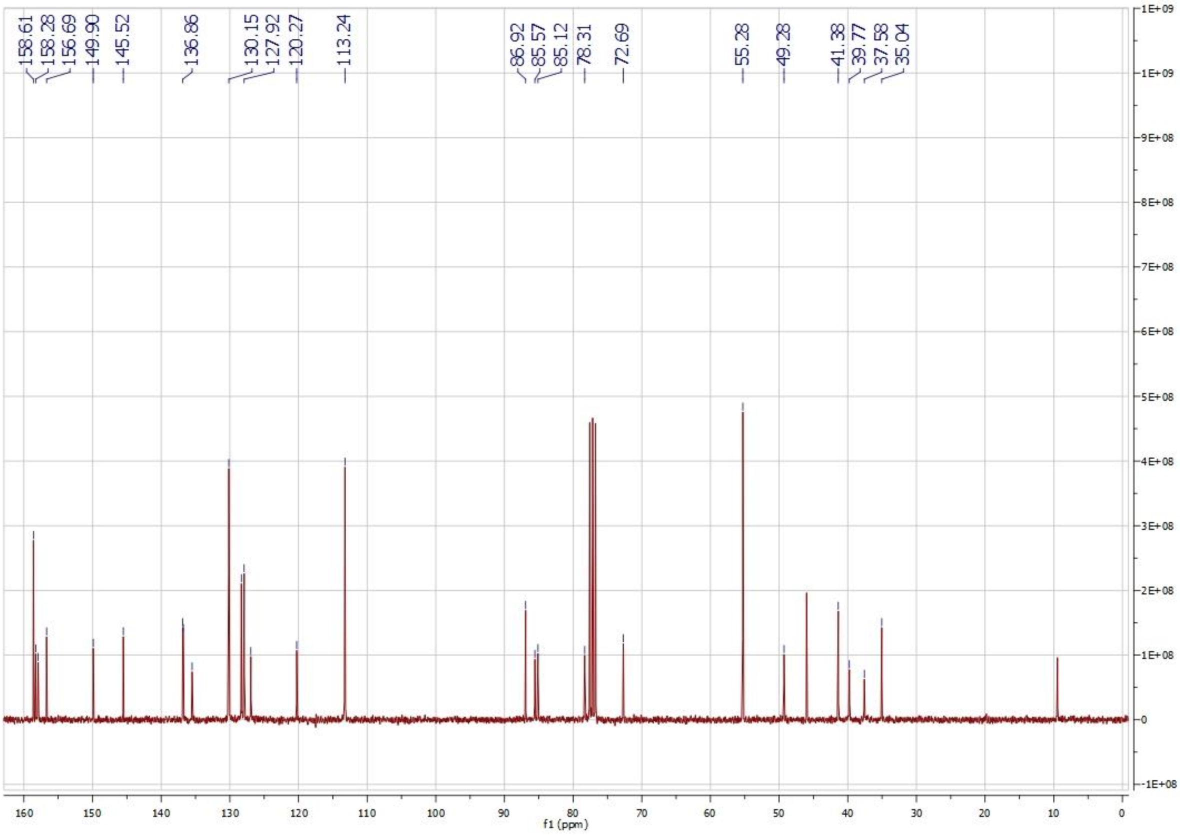

158.61
158.28
156.69
149.90
145.52
136.86
130.15
127.92
120.27
113.24
86.92
85.57
85.12
78.31
72.69
55.28
49.28
41.38
39.77
37.58
35.04
f1 (ppm)

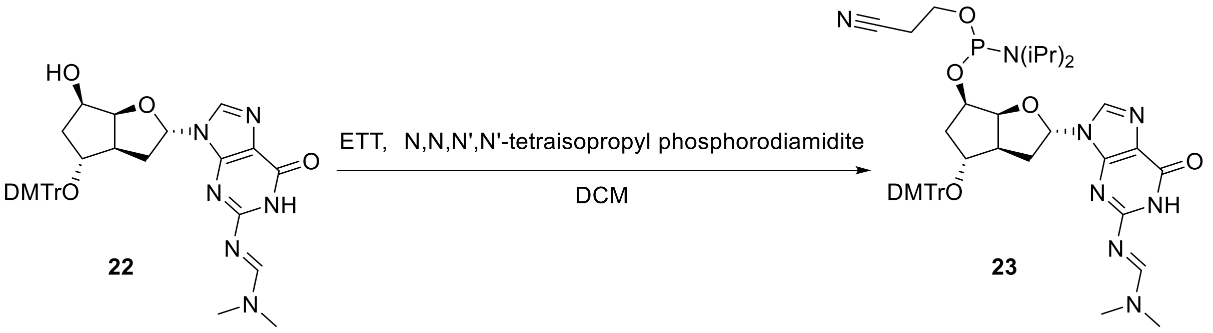

(3’S,5’R,7’R)-N2-(N,N-Dimethylformamidino)-9-{5’-O-[(2-cyanoethoxy)-diisopropylaminophosphanyl]-2’,3’-dideoxy-3’,5’-ethano-7’-O-[(4,4’-dimethoxytriphenyl)methyl]-α-D-ribofuranosyl} guanine (23) :

To a solution of the nucleoside **22** (245 mg, 0.377 mmol) and 5-(Ethylthio)-1H-tetrazole (74 mg, 0.57 mmol) in dry DCM (15 mL) was added dropwise 2-Cyanoethyl N,N,N′,N′-tetraisopropylphosphordiamidite (0.20 mL, 0.64 mmol) at rt. After stirring for 50 min, the reaction mixture was diluted with satd $NaHCO_3$ (25 mL) and extracted with DCM (3 X 25 mL). The combined organic phases were dried over $MgSO_4$, filtered and evaporated. The crude product was purified by CC (3% MeOH in DCM, +0.5 % Et_3N) to yield **23** (212 mg, mixture of two isomers, 67%) as a white foam.

Data for **23**: R_f = 0.42 (7% MeOH in DCM);

^{1}H NMR (300 MHz, $CDCl_3$) δ 9.35 (*br*, 1H, H-N(1)), 8.51, 8.49 (2s, 1H, NC*H*N(CH_3)$_2$), 7.41 – 7.10 (m, 10H, H-arom, H-C(8)), 6.83 – 6.70 (m, 4H, H-arom), 6.15 – 6.00 (m, 1H, H-C(1’)), 4.64 – 4.36 (m, 2H, H-C(4’), H-C(5’)), 3.90 – 3.82 (m, 1H, H-C(7’)), 3.80 – 3.62 (m, 8H, *Me*O, OC*H*$_2$CH$_2$CN), 3.59 – 3.43 (m, 2H, (Me$_2$C*H*)$_2$N), 3.04, 3.02 (2s, 6H, NCHN(C*H*$_3$)$_2$), 2.67 – 2.48 (m, 2H, OCH$_2$C*H*$_2$CN), 2.32 (ddd, *J* = 24.1, 15.1, 6.7 Hz, 1H, H-C(3’)), 2.02 – 1.63 (m, 4H, H-C(2’), H-C(6’)), 1.14 – 1.03 (m, 12H, (*Me*$_2$CH)$_2$N).

^{13}C NMR (101 MHz, $CDCl_3$) δ 158.76 (MeO-*C*-arom), 158.17, 158.12 (C(2)), 158.03 (N*C*HN(CH_3)$_2$), 156.66, 156.59 (C(6)), 149.85, 149.79 (C(4)), 145.51, 145.49 , 136.84, 136.77, 136.73, 136.71 (C-arom), 135.76, 135.59 (C(8)), 130.24, 130.20, 128.41, 128.33, 128.02, 127.10, 127.08 (CH-arom), 120.74, 120.70 (C(5)), 117.98, 117.72 (OCH$_2$CH$_2$*C*N), 113.34 (CH-arom), 87.16, 87.10 (*C*(Ph)$_3$), 86.00, 85.72 (C(1’)), 84.13, 84.10 ($J_{C,P}$ = 3.6, 2.5 Hz, C(4’)), 78.02, 77.67 (C(7’)), 74.15, 73.74 ($J_{C,P}$ = 15.3, 18.7 Hz, C(5’)), 58.90, 58.67 ($J_{C,P}$ = 18.7, 19.7 Hz O*C*H$_2$CH$_2$CN), 55.38, 55.36 (*Me*O-DMTr), 49.20, 49.09 (C(3’)), 43.20, 43.15 ($J_{C,P}$ = 12.4, 12.6 Hz, ((Me$_2$*C*H)$_2$N), 41.42, 41.38 (NCHN(*C*H$_3$)$_2$), 38.68, 38.65 (C(6’)), 37.97, 37.84 (C(2’)), 35.25 (NCHN(*C*H$_3$)$_2$), 24.83, 24.75, 24.68, 24.60, 24.53 ((*Me*$_2$CH)$_2$N), 20.35, 20.28 (OCH$_2$*C*H$_2$CN).

^{31}P NMR (121 MHz, $CDCl_3$) δ 148.21, 148.01.

ESI$^+$-HRMS *m/z* calcd for $C_{45}H_{56}O_7N_8P$ ([M + H]$^+$) 851.4004, found 851.4013.

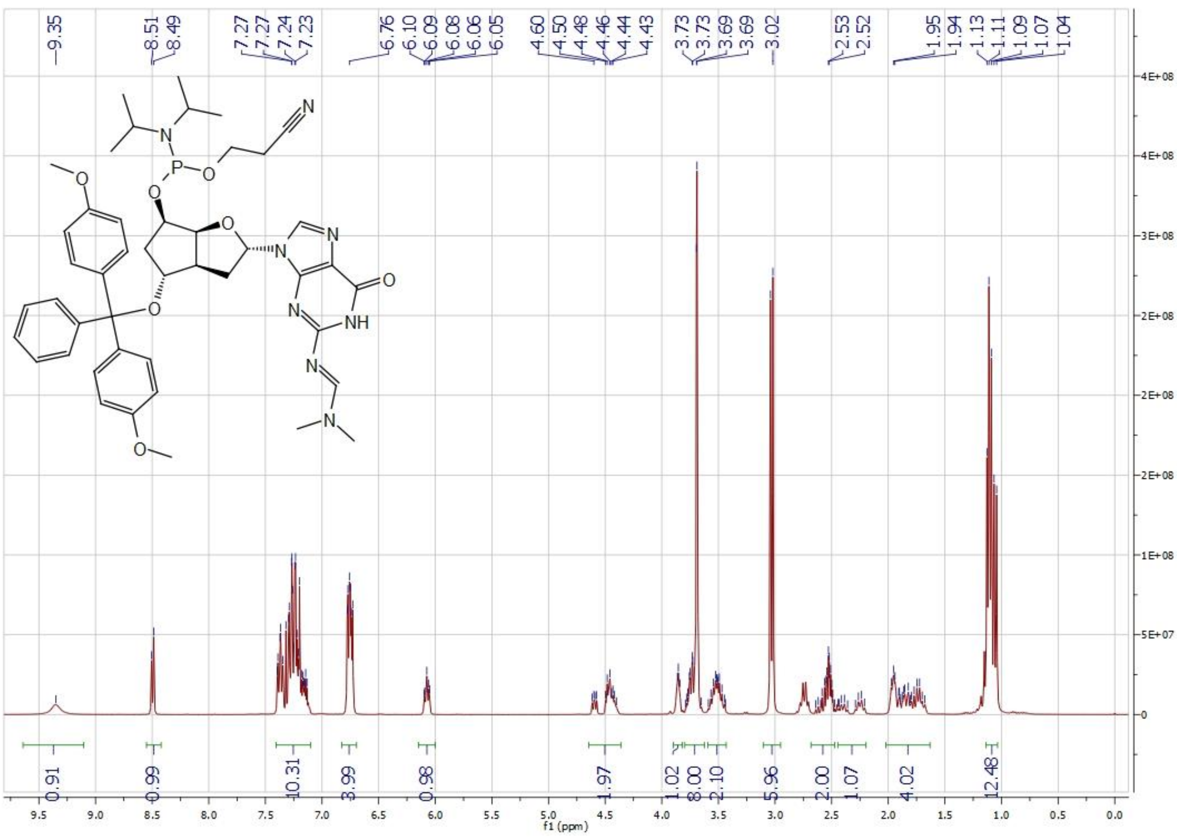
9.35
8.51
8.49
7.27
7.27
7.24
7.23
6.76
6.10
6.09
6.08
6.06
6.05
4.60
4.50
4.48
4.46
4.44
4.43
3.73
3.73
3.69
3.69
3.02
2.53
2.52
1.95
1.94
1.13
1.11
1.09
1.07
1.04
0.91
0.99
10.31
3.99
0.98
1.97
1.02
8.00
2.10
5.96
2.00
1.07
4.02
12.48
f1 (ppm)

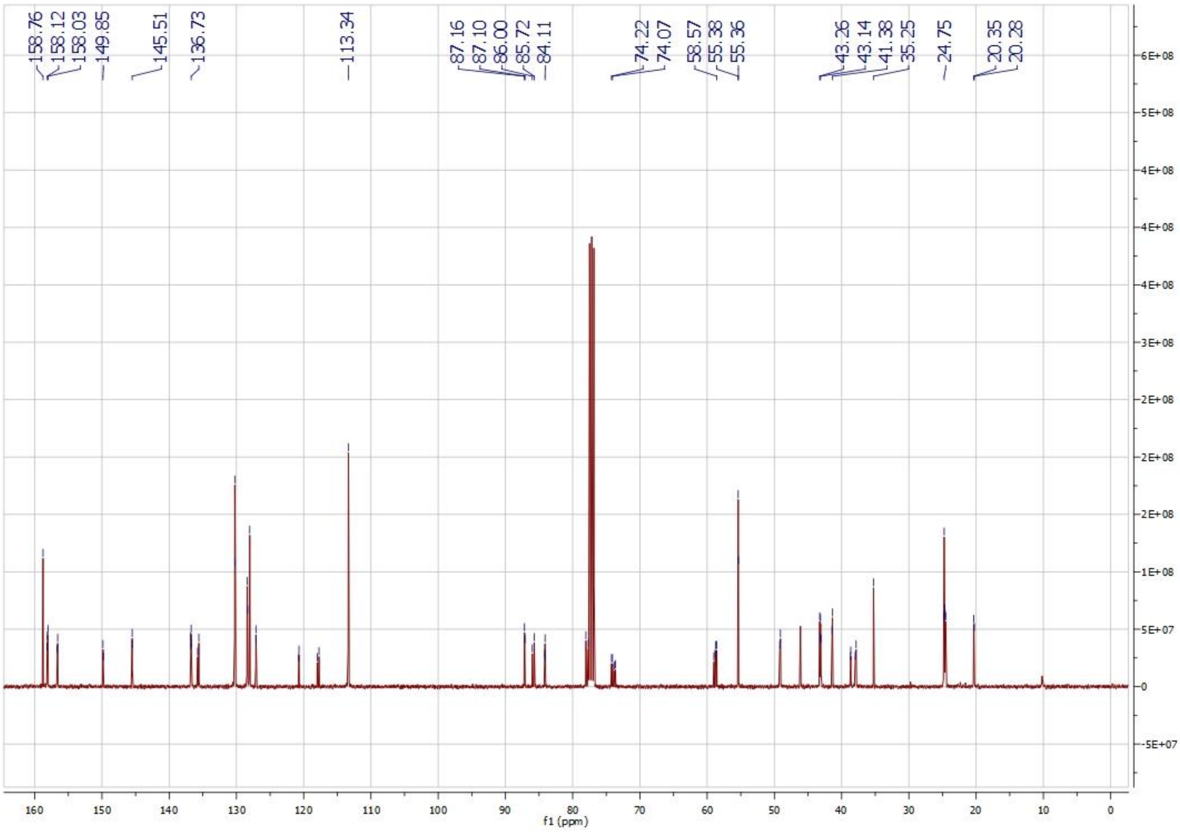
158.76
158.12
158.03
149.85
145.51
136.73
113.34
87.16
87.10
86.00
85.72
84.11
74.22
74.07
58.57
55.38
55.36
43.26
43.14
41.38
35.25
24.75
20.35
20.28
f1 (ppm)

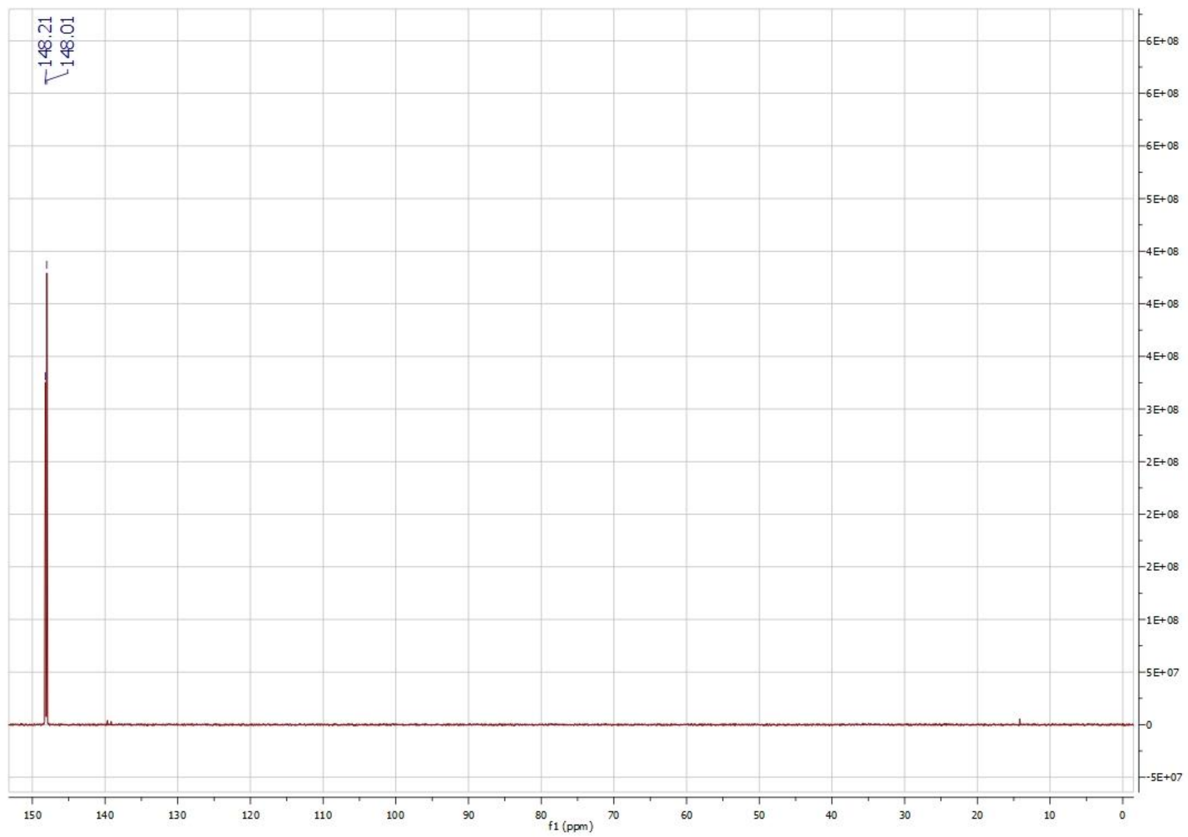

3.2. Synthesis of 5'-O-p-nitrobenzoyl-7',5'-α-bc-T

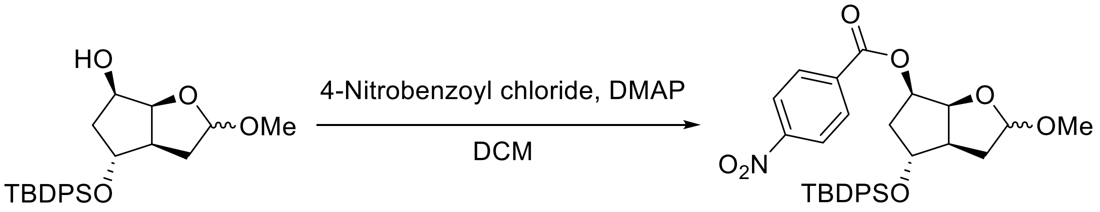

24 **25**

(3aR,4R,6R,6aS)-4-((Tert-butyldiphenylsilyl)oxy)-2-methoxyhexahydro-2H-cyclopenta[b]furan-6-yl (4-nitrobenzoate) (25) :

To a solution of the sugar **24** (195 mg, 0.437 mmol) and 4-Dimethylaminopyridine (70 mg, 0.568 mmol) in dry DCM (10 mL) was added 4-Nitrobenzoyl chloride (158 mg, 0.850 mmol) at rt. After stirring overnight, reaction was quenched by slow addition of satd $NaHCO_3$ (3 mL). The mixture was then diluted with satd $NaHCO_3$ (15 mL) and extracted with DCM (3 X 15 mL). The combined organic phases were dried over $MgSO_4$, filtered and evaporated. The crude product was purified by CC (EtOAc/hexanne 1:5) to yield a mixture of **25** (260 mg, 98%) in an anomeric ratio α/β ≈ 4:1 as a white solid.

Data for **25**: R_f = 0.62 (EtOAc/hexane 1:2);

^{1}H NMR (300 MHz, $CDCl_3$) δ 8.33 – 8.17 (m, 4H, H-arom), 7.72 – 7.61 (m, 4H, H-arom), 7.51 – 7.32 (m, 6H, H-arom), 5.65 – 5.47 (m, 1H, H-C(6)), 4.97 (dd, *J* = 9.2, 5.6 Hz, 1H, H-C(2)), 4.87 (t, *J* = 5.8 Hz, 1H, H-C(6a)), 4.18 (d, *J* = 5.0 Hz, 0.2H, H-C(4)), 3.98 (d, *J* = 3.5 Hz, 0.8H, H-C(4)), 3.21 (d, *J* = 15.1 Hz, 3H, *Me*O), 2.88 (dd, *J* = 16.6, 7.9

Hz, 0.8H, H-C(3a)), 2.75 – 2.62 (m, 0.2H, H-C(3a)), 2.49 – 2.34 (m, 0.2H, H-C(5)), 2.24 – 1.83 (m, 2.8H, H-(5), H-C(3)), 1.28 (ddd, *J* = 13.0, 7.9, 4.9 Hz, 1H, H-C(3)), 1.09 (s, 9H, (C*H*$_3$)$_3$-C-Si).

^{13}C NMR (75 MHz, $CDCl_3$) δ 164.46, 164.41 (*C*O_2R), 150.63 (O_2N-*C*-arom), 135.87, 135.82 (CH-arom), 134.07, 133.75, 133.69 (CH-arom), 130.98, 130.89, 129.98, 129.96, 129.91, 127.89, 127.87, 127.85, 123.59 (CH-arom), 106.49, 106.39 (C(2)), 83.21, 79.87 (C(6a)), 76.54 (C(4)), 76.09 (C(6)), 54.55, 54.47 (*Me*O), 51.69, 50.30 (C(3a), 38.07 (C(3)), 37.17, 36.65 (C(5)), 27.04, 26.99 90 ((*C*H$_3$)$_3$-C-Si), 19.14 ((CH$_3$)$_3$-*C*-Si).

ESI$^+$-HRMS *m/z* calcd for $C_{31}H_{35}O_7NaSi$ ([M + Na]$^+$) 584.2075, found 584.2085.

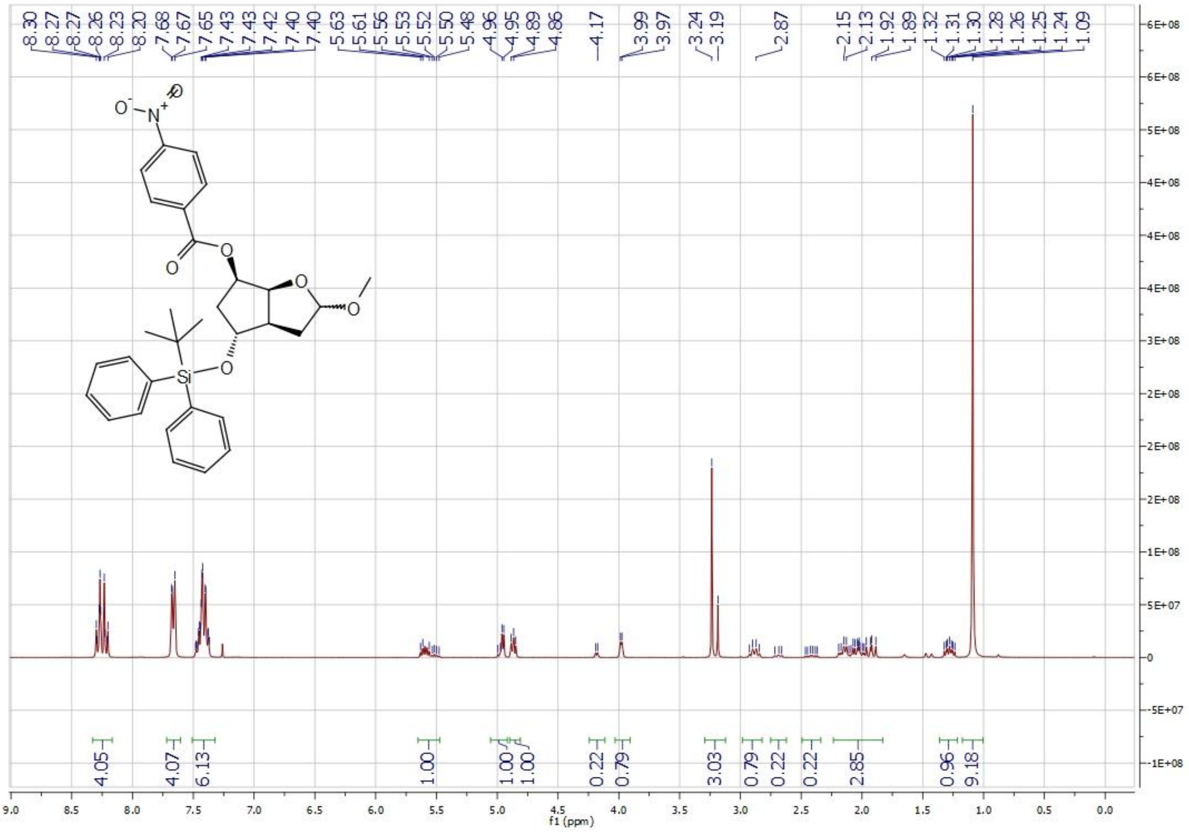

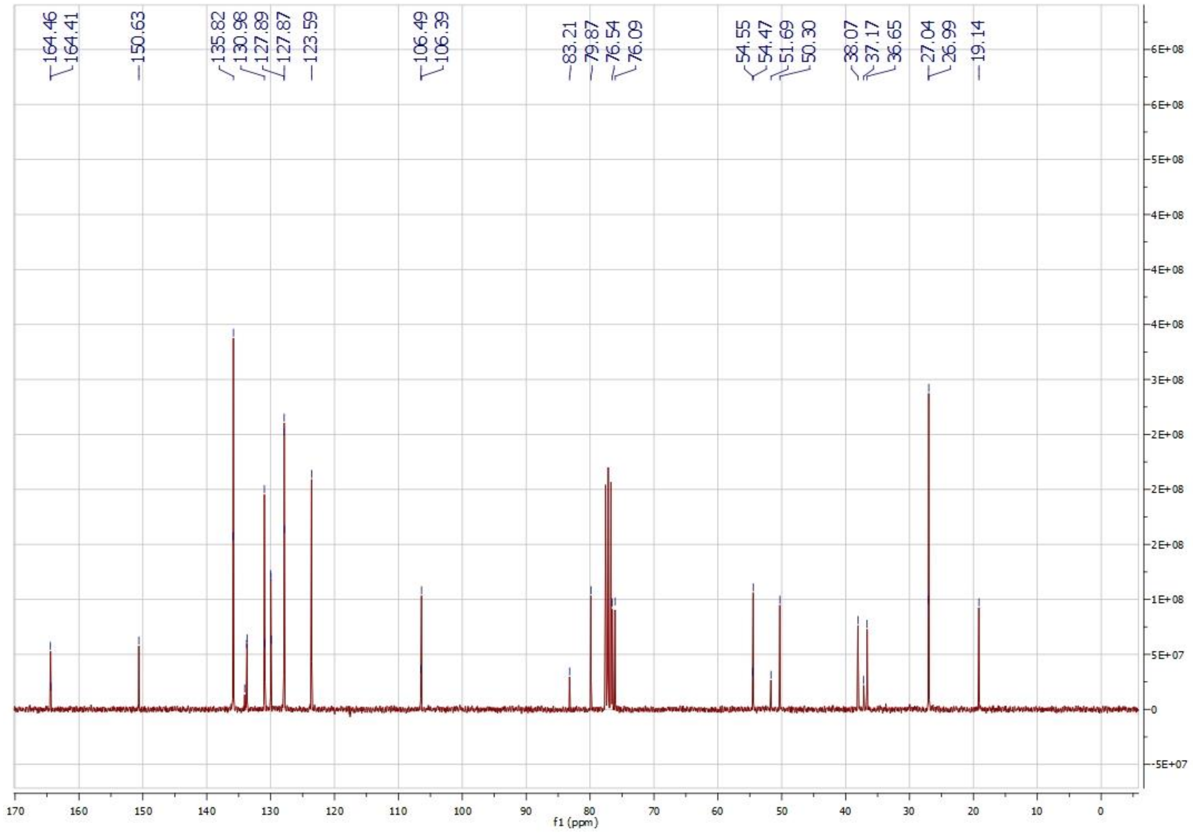

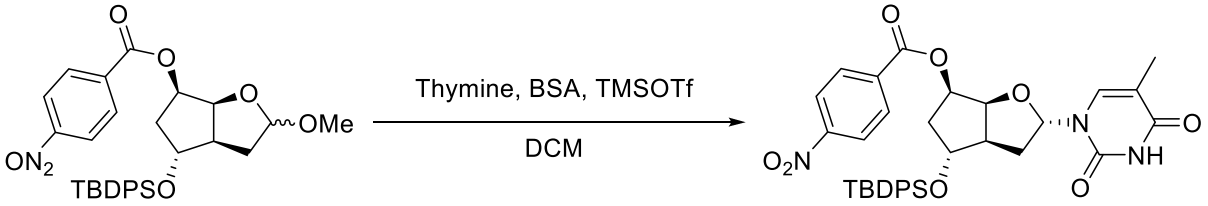

25 **26**

(3’R,5’R,7’R)-1-{7’-[(tert-butyldiphenylsilyl)oxy]-2’,3’-dideoxy-3’,5’-ethano-5’-O-(4-nitrobenzoate)-α,β-D-ribofuranosyl} thymine (26) :

To a solution of the sugar **25** (260 mg, 0.463 mmol) and thymine (84 mg, 0.695 mmol) in dry MeCN (3 mL) was added dropwise BSA (0.34 mL, 1.4 mmol) at rt. After stirring for 30 min at rt, the solution was cooled down to 0°C and TMSOTf (0.10 mL, 1.3 mmol) was added dropwise. After further stirring for 2 h at 0° C and for 18 h at rt, the reaction mixture was diluted with satd $NaHCO_3$ (30 mL) and extracted with DCM (4 X 40 mL). The combined organic phases were dried over $MgSO_4$, filtered and evaporated. The crude product was purified by CC (2% MeOH in DCM) to yield a mixture of **26** (240 mg, 79%) in an anomeric ratio α/β ≈ 88:12 as white foam.

Data for **26**: R_f = 0.56 (DCM + 3% MeOH);

^{1}H NMR (300 MHz, $CDCl_3$) δ 9.38 (*br*, 1H, (s, 1H, H-N(3)), 8.32 – 8.23 (m, 2H, H-arom), 8.22 – 8.11 (m, 2H, H-arom), 7.65 (dd, *J* = 7.7, 1.5 Hz, 4H, H-arom), 7.50 – 7.36 (m, 6H, H-arom), 6.95 (d, *J* = 0.9 Hz, 1H, H-C(6)), 5.96 (t, *J* = 6.3 Hz, 1H, H-C(1’)), 5.55 (dt, *J* = 9.9, 6.0 Hz, 1H, H-C(5’)), 5.13 (dd, *J* = 6.4, 5.4 Hz, 1H, H-C(4’)), 4.20 – 4.05 (m, 1H, H-C(7’)), 2.94 – 2.78 (m, 1H, H-C(3’)), 2.22 (dd, *J* = 13.3, 6.4 Hz, 1H, H-C(6’)), 2.09 – 1.73 (m, 6H, H-C(6’), H-C(2’), *Me*-C(5)), 1.09 (s, 9H, (CH_3)$_3$-C-Si).

^{13}C NMR (75 MHz, $CDCl_3$) δ 164.32, 163.79 (C(4), *C*O$_2$R), 150.65, 150.39 (O_2N-*C*-arom, C(2)), 135.70, 135.68 (CH-arom), 135.13 (C-arom), 134.83 (C(6)), 133.46, 133.10 (C-arom), 130.91, 130.73, 130.11, 127.93, 123.60 (CH-arom), 111.30 (C(5)), 87.26 (C(1')), 82.44 (C(4')), 76.40 (C(7')), 76.07 (C(5')), 50.76 (C(3')), 37.94 (C(6')), 36.68 (C(2')), 26.89 ((*C*H$_3$)$_3$-C-Si), 19.03 ((CH$_3$)$_3$-*C*-Si), 12.62 (*Me*-C(5)).

ESI$^+$-HRMS *m/z* calcd for $C_{35}H_{37}O_8N_3NaSi$ ([M + Na]$^+$) 678.2242, found 678.2254.

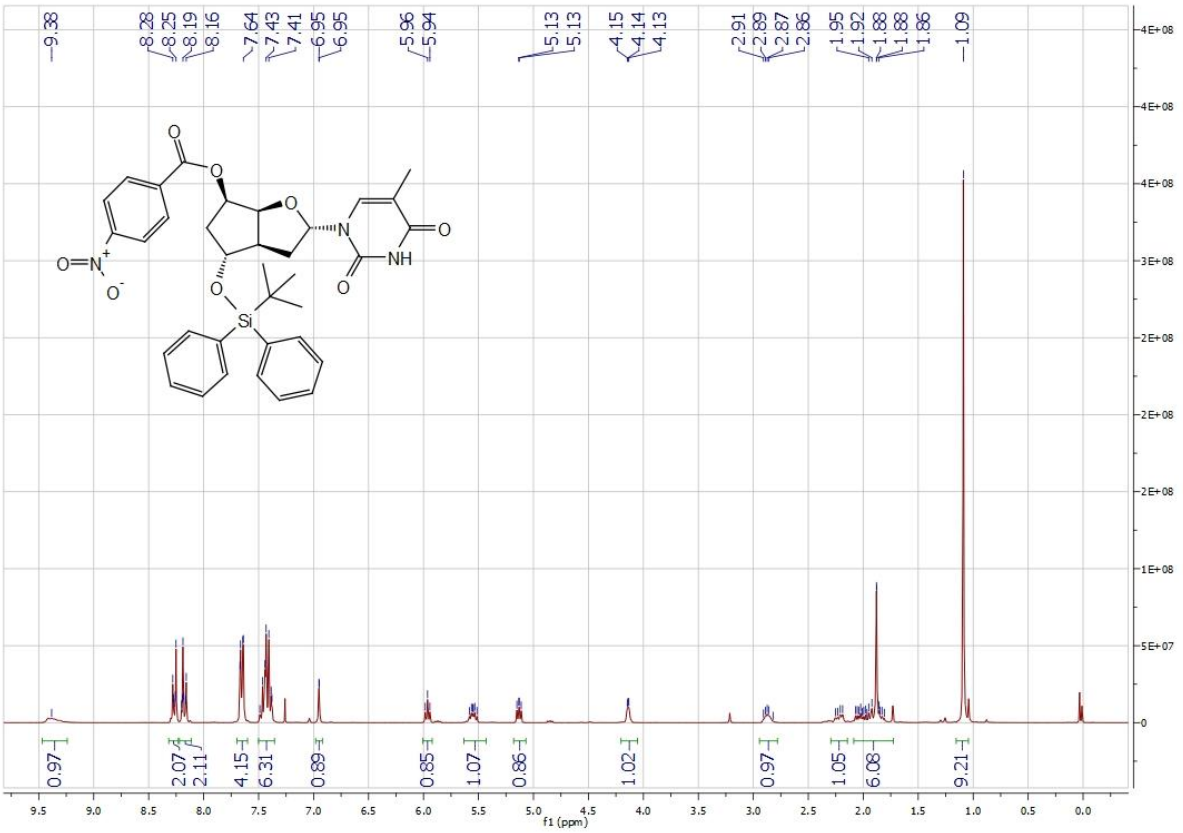

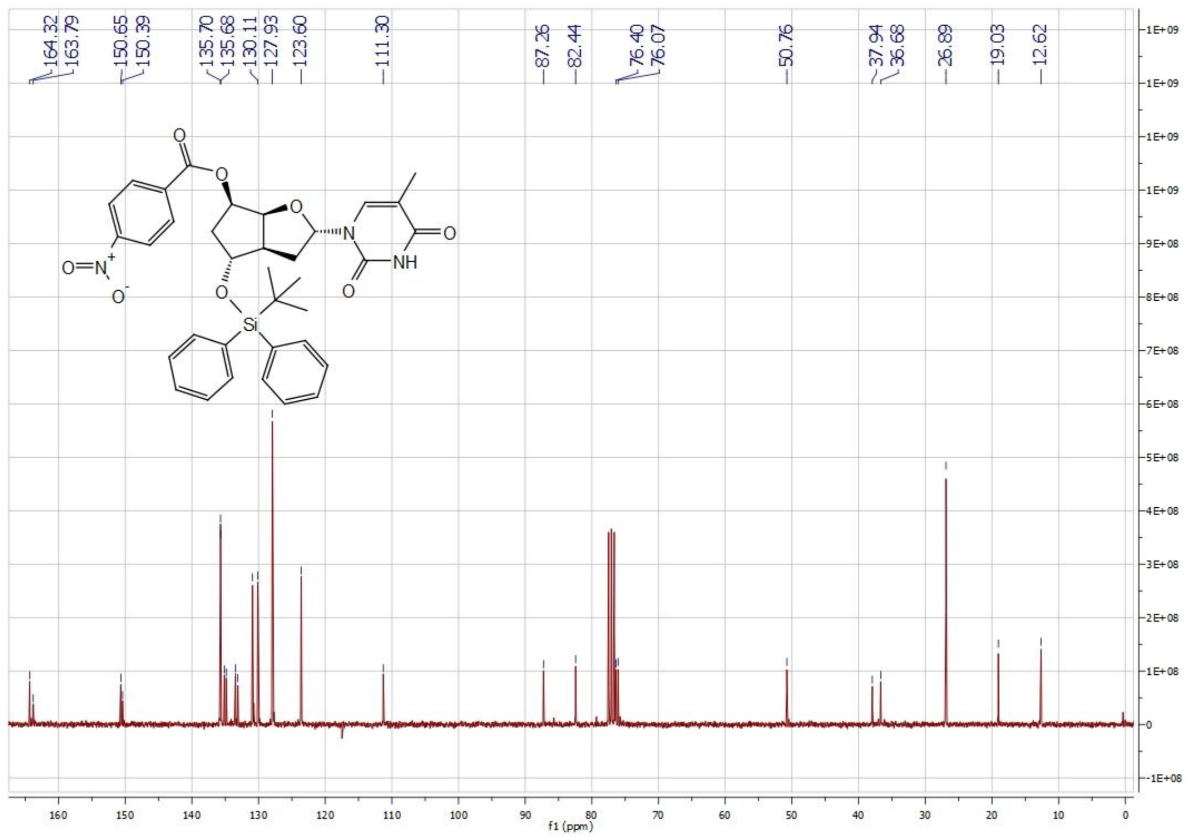

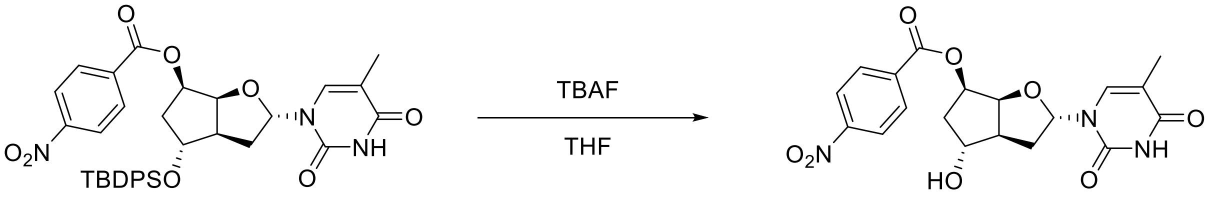

(3'R,5'R,7'R)-1-{2',3'-dideoxy-3',5'-ethano-7'-hydroxy-5'-O-(4-nitrobenzoate)-α,β-D-ribofuranosyl} thymine (27) :

To a solution of the nucleoside **26** (220 mg, 0.335 mmol) in dry THF (2 mL) was added TBAF (1M in THF, 0.84 mL, 0.84 mmol) at rt. After stirring for 4 h at rt, the reaction mixture was diluted with satd $NaHCO_3$ (20 mL) and extracted with EtOAc (3 X 20 mL) and DCM (2 X 80 mL). The combined organic phases were dried over $MgSO_4$, filtered and evaporated. The crude product was purified by CC (5% MeOH in DCM) to yield an anomeric mixture of **27** (101 mg, 72%). Crystals suitable for X-ray analysis were obtained by recrystallization in EtOAc.

Data for **27**: R_f = 0.50 (DCM +7% MeOH);

^{1}H NMR (300 MHz, $CDCl_3$) δ 8.96 (*br*, 1H, H-N(3)), 8.34 – 8.17 (m, 4H, H-arom), 7.07 (d, *J* = 1.1 Hz, 1H, H-C(6)), 6.11 (t, *J* = 6.3 Hz, 1H, H-C(1')), 5.57 – 5.45 (m, 1H, H-C(5')), 5.15 (dd, *J* = 6.6, 5.4 Hz, 1H, H-C(4')), 4.38 – 4.23 (m, 1H, H-C(7')), 2.96 (dd, *J* = 13.5, 6.9 Hz, 1H, H-C(3')), 2.26 (ddd, *J* = 13.1, 10.3, 5.4 Hz, 4H, H-C(2'), H-C(6')), 1.91 (d, *J* = 0.9 Hz, 3H, *Me*-C(5)).

ESI$^+$-HRMS *m/z* calcd for $C_{19}H_{19}O_8N_3Na$ ([M + Na]$^+$) 440.1064, found 440.1072.

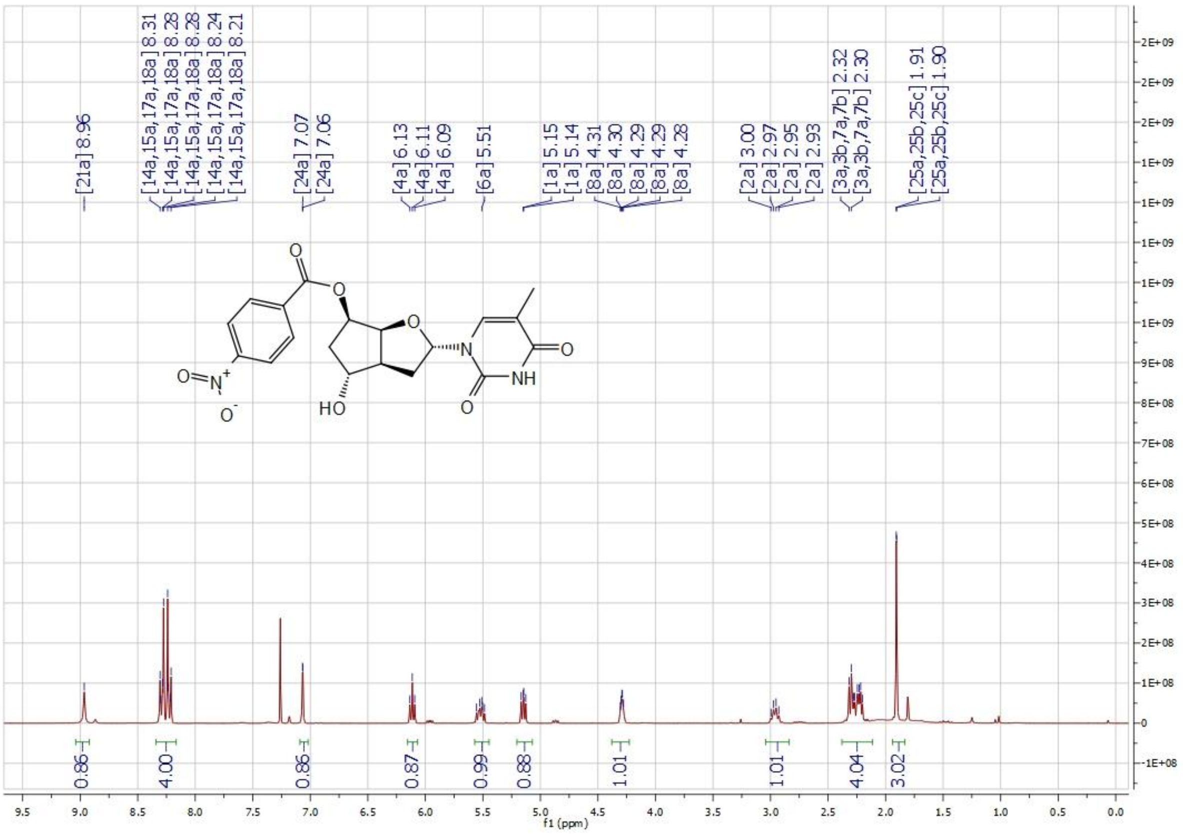

4. NOESY spectra

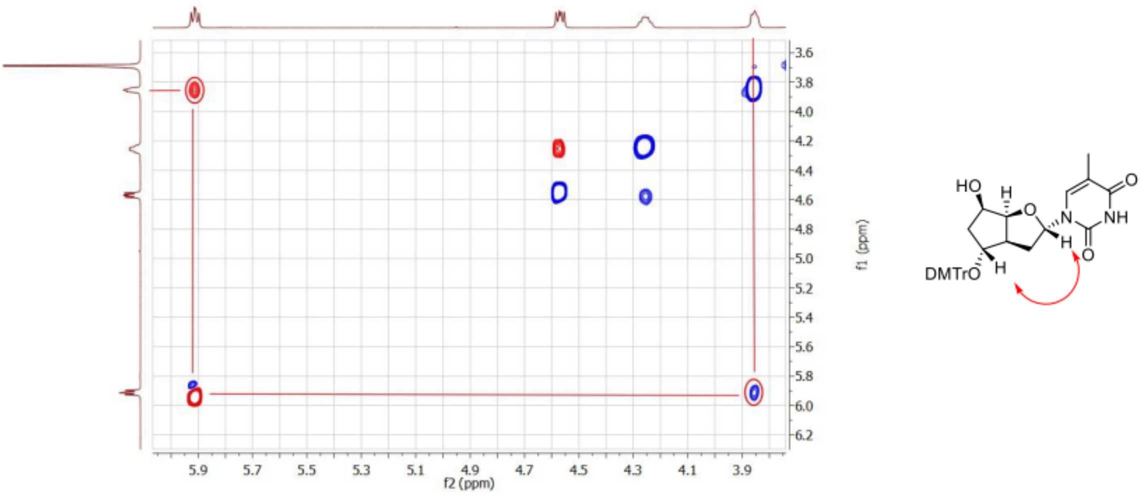

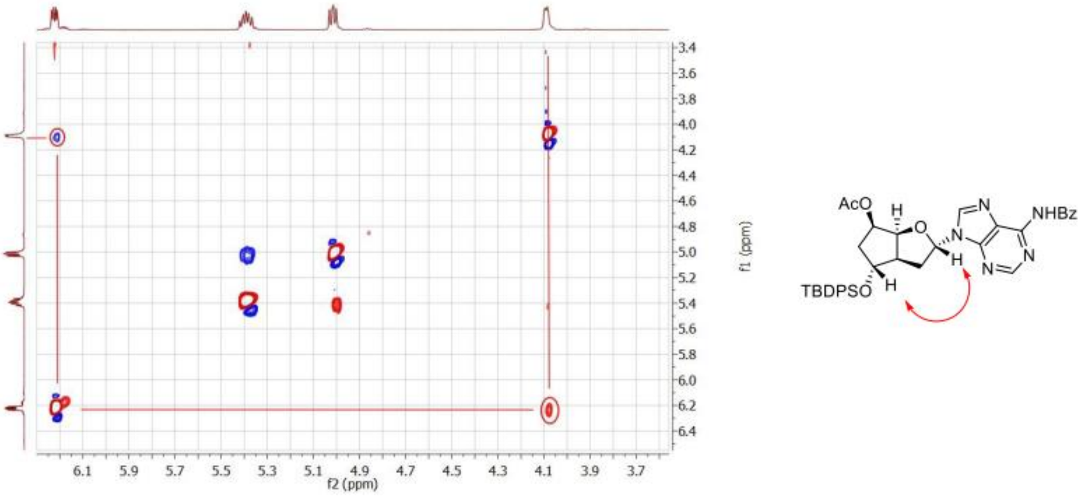

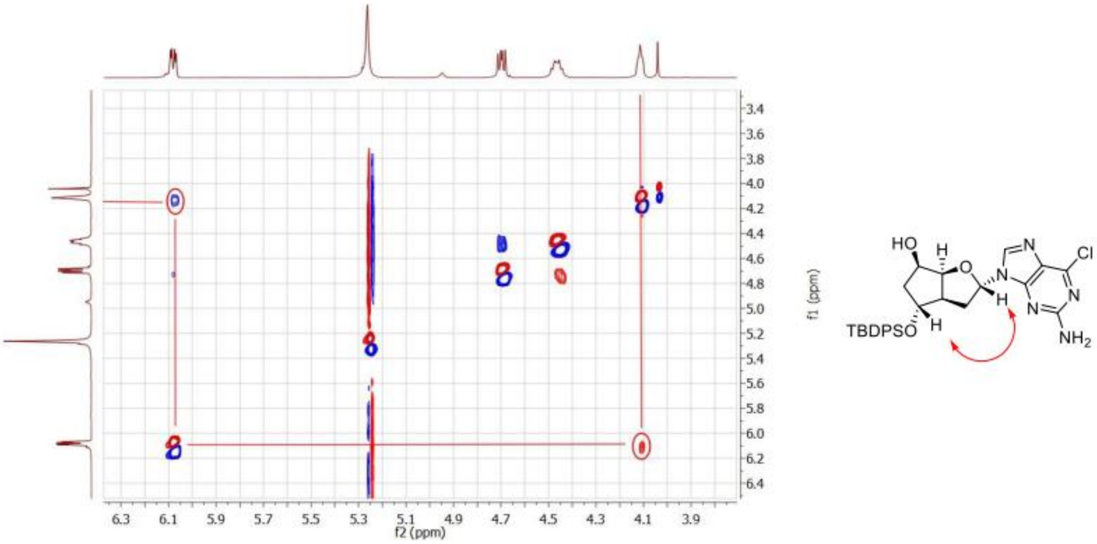

5. HMBC spectra

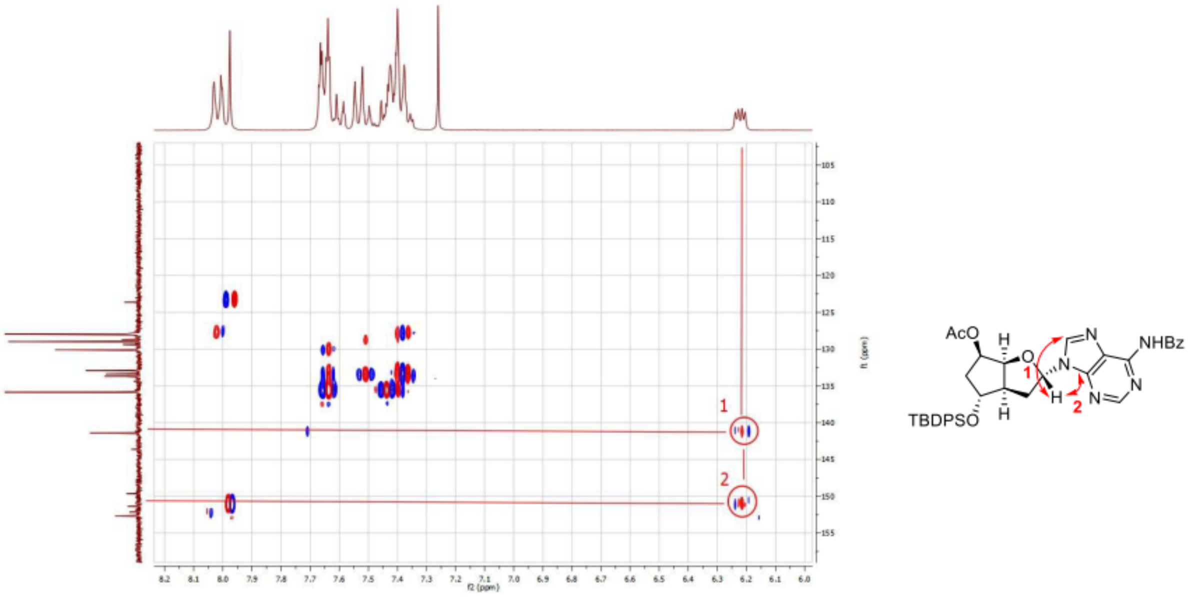

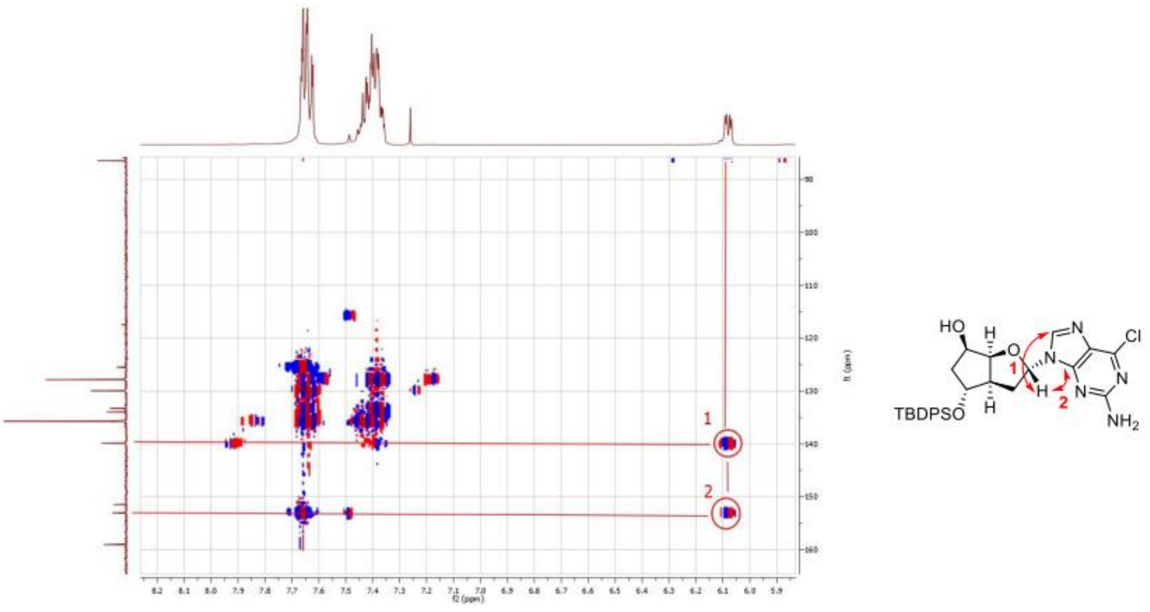

6. Anomer separations

In general, the presence of anomers was quantified by H[1]-NMR. The orientation of the anomeric center was confirmed by 2D-NMR (see above), and by X-ray structure (see below) for the Thymine and Guanine nucleosides. The full characterization of the β-anomers was reported previously[1]. The anomers were separated at different steps during the synthetic pathway by standard column chromatography techniques. We report here the liquid chromatography analyses of the separation steps:

Thymidine building block:

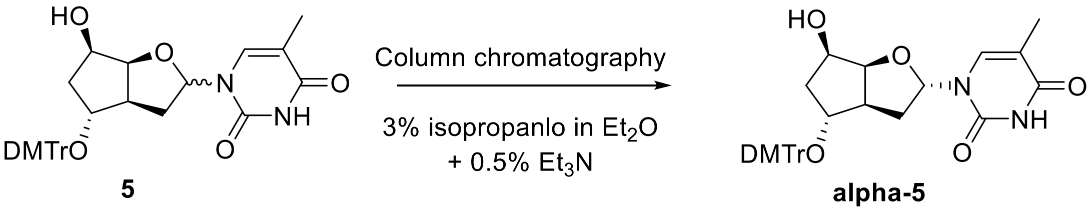

Crude product:

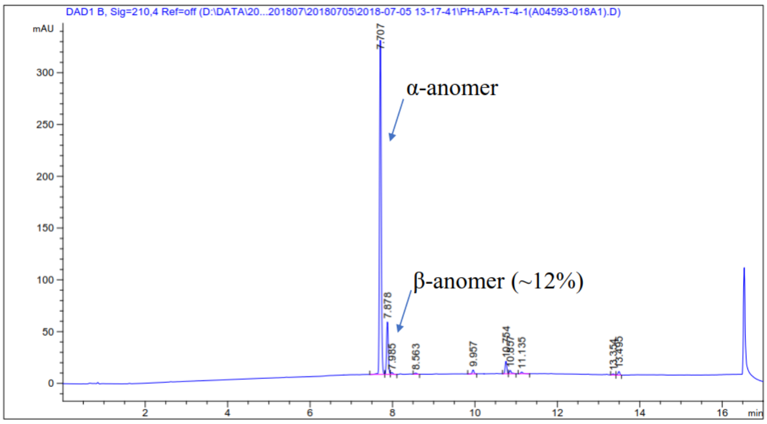

Purified product (containing <1% of β-anomer):

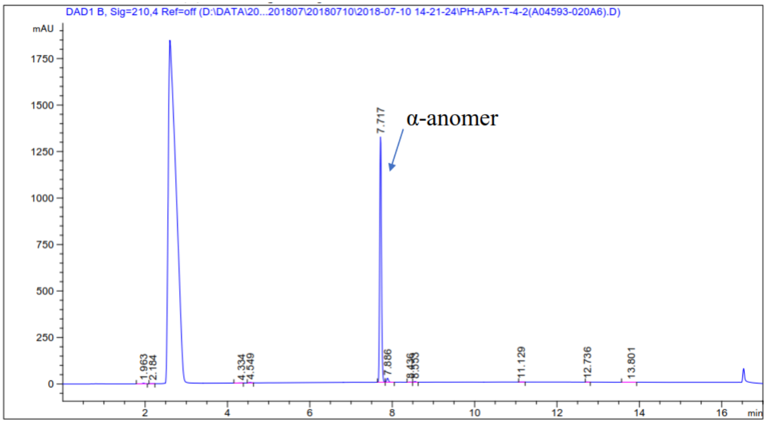

**Column**	Ascentis Express C18, 4.6×100 mm, 2.7 μm
**Mobile phase A**	Water
**Mobile phase B**	ACN
**Column Temp**	40℃
**Flow Rate:**	1.2mL/min
**Diluent/Blank**	ACN
**Run Time**	19.1min (post time 5 min)

**Time(min)**	0.0	5	9	14	16	19	19.1
**%Mobile phase A**	95	80	50	40	0	0	95
**%Mobile phase B**	5	20	50	60	100	100	5

Adenosine building block:

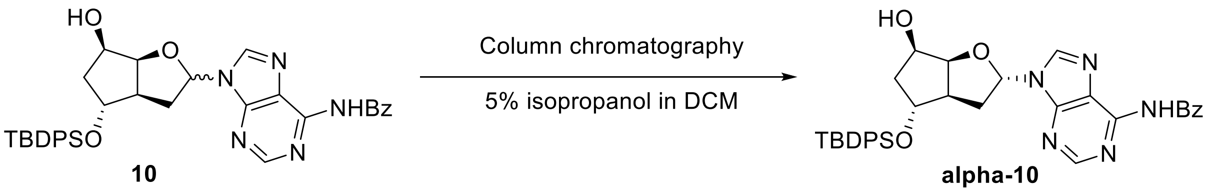

Crude product:

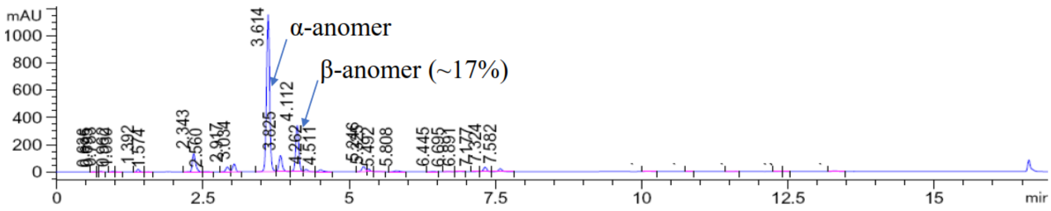

Purified product (containing <1% of β-anomer):

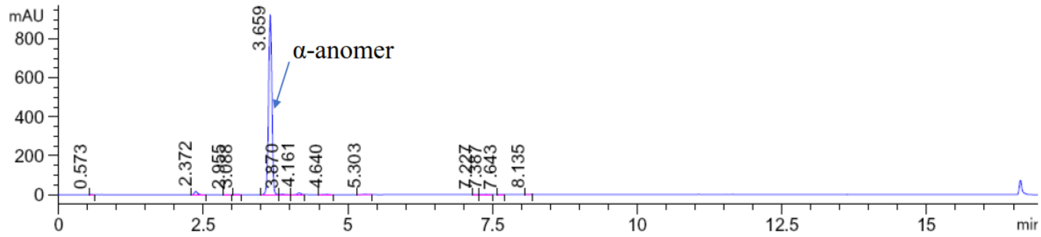

**Column**	Ascentis Express C18, 4.6×100 mm, 2.7 μm
**Mobile phase A**	Water
**Mobile phase B**	ACN
**Column Temp**	40℃
**Flow Rate:**	1.2mL/min
**Diluent/Blank**	ACN
**Run Time**	17.0min(post time 3 min)

**Time(min)**	0.0	12	15	20	20.1	22
**%Mobile phase A**	40	25	0	0	40	40
**%Mobile phase B**	60	75	100	100	60	60

Guanosine building block:

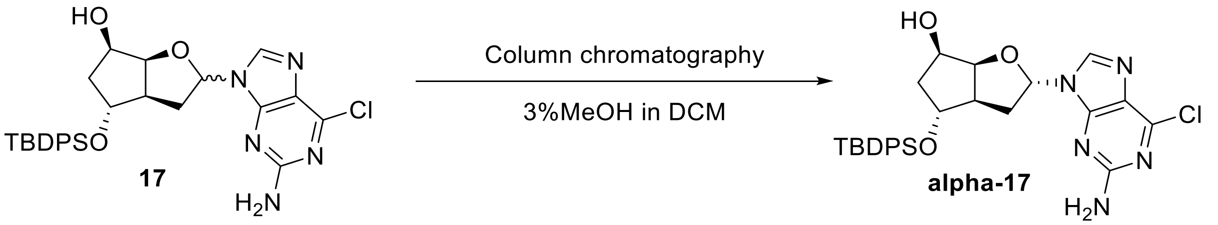

Crude product:

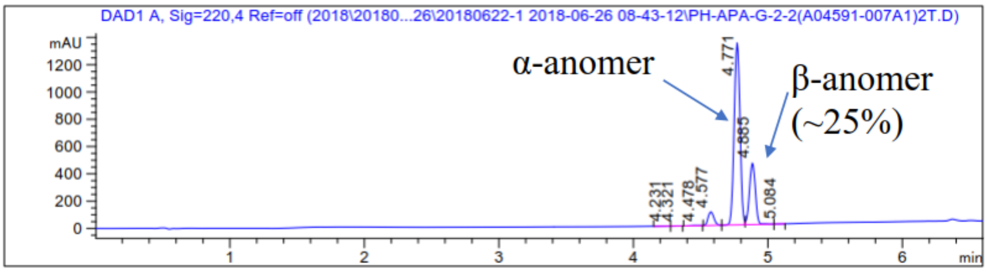

Purified product (containing <1% of β-anomer):

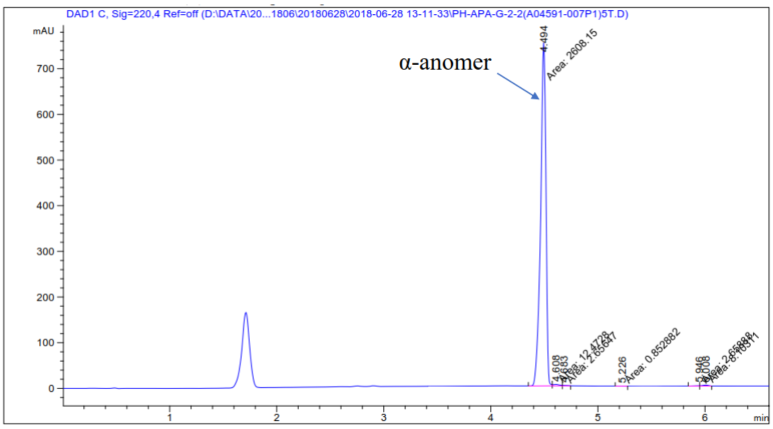

**Column**	Agilent XDB C18, 4.6×50 mm, 1.8 µm
**Mobile phase A**	0.05%TFA in Water
**Mobile phase B**	0.05%TFA in ACN
**Column Temp**	40℃
**Flow Rate:**	1.2mL/min
**Diluent/Blank**	ACN
**Run Time**	6.6min(post time 4 min)

**Time(min)**	0.0	4.5	6.5	6.6
**%Mobile phase A**	90	0	0	90
**%Mobile phase B**	10	100	100	10

5-Methylcytidine building block:

The methylcytosine synthetic pathway use the previously purified thymine nucleoside. Thus, the anomeric separation is already performed.

7. Crystal-structure determination

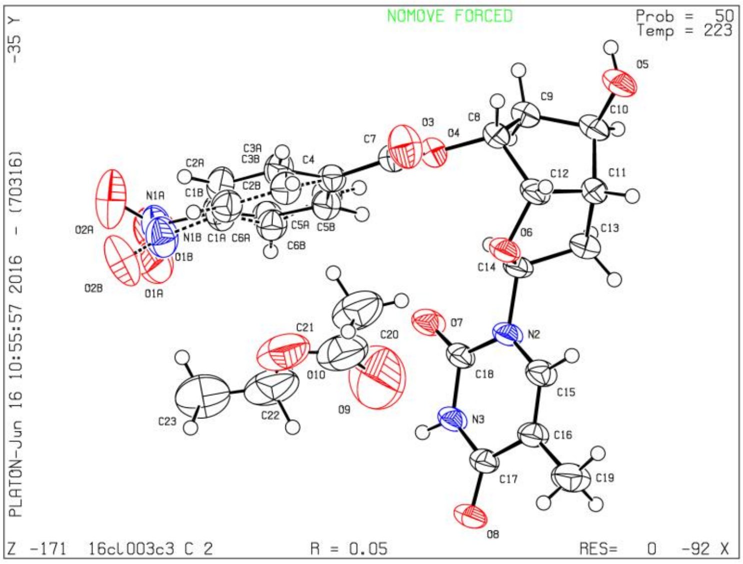

Crystal-Structure Determination 27. A colorless transparent crystal of $[C_{19}H_{19}N_3O_8]_2[0.5(C_4H_8O_2)]$ was mounted in air and used for X-ray structure determination at ambient conditions. All measurements were made on a *Oxford Diffraction SuperNova* area-detector diffractometer[2] using mirror optics monochromated Mo *K*α radiation (λ = 0.71073 Å) and Al filtered.[3] The unit cell constants and an orientation matrix for data collection were obtained from a least-squares refinement of the setting angles of reflections in the range 1.7° < θ < 28.07°. A total of 440 frames were collected using ω scans, with 15+15 seconds exposure time, a rotation angle of 1° per frame, a crystal-detector distance of 65.1 mm, at T = 223(2) K.

Data reduction was performed using the *CrysAlisPro*[2] program. The intensities were corrected for Lorentz and polarization effects, and an absorption correction based on the multi-scan method using SCALE3 ABSPACK in *CrysAlisPro*[2] was applied. Data collection and refinement parameters are given in *Table S3.*

The structure was solved by direct methods using *SHELX*[4], which revealed the positions of all non-hydrogen atoms of the title compound. The non-hydrogen atoms were refined anisotropically. All H-atoms were placed in geometrically calculated positions and refined using a riding model where each H-atom was assigned a fixed isotropic displacement parameter with a value equal to 1.2Ueq of its parent atom.

Refinement of the structure was carried out on F^2 using full-matrix least-squares procedures, which minimized the function $\Sigma w(F_o^2 - F_c^2)^2$. The weighting scheme was based on counting statistics and included a factor to downweight the intense reflections. All calculations were performed using the *SHELXL-2014/*[4] program.

The compound crystallizes in the monoclinic space group *C 2*, with a monoclinic angle very close to 90 degrees, which implies an easy pseudo-merohedral twinning, that cannot be easily deconvoluted. Moreover, the p-NO_2-

benzoate group of the main molecule is disordered over two conformation and the co-crystallized acetate solvent is disordered about a twofold axis. For all these reasons, the structure determination and refinement was somewhat complicated, some short intermolecular contacts are calculated, and the absolute configuration cannot be determined (Flack parameter being unrealistic), but it is assigned according to the reaction sequence.

Table S3. Crystal data and structure refinement for **27**.

Identification code	shelx	
Empirical formula	C21 H23 N3 O9	
Formula weight	461.42	
Temperature	223(2) K	
Wavelength	0.71073 Å	
Crystal system	Monoclinic	
Space group	C 2	
Unit cell dimensions	a = 22.6224(5) Å	a= 90°.
	b = 7.9610(2) Å	b= 90.172(2)°.
	c = 12.0447(3) Å	g = 90°.
Volume	2169.20(9) Å^3	
Z	4	
Density (calculated)	1.413 Mg/m^3	
Absorption coefficient	0.112 mm^{-1}	
F(000)	968	
Crystal size	0.344 x 0.265 x 0.072 mm^3	
Theta range for data collection	1.691 to 28.071°.	
Index ranges	-28<=h<=28, -9<=k<=10, -15<=l<=15	
Reflections collected	7292	
Independent reflections	4360 [R(int) = 0.0206]	
Completeness to theta = 25.242°	100.0 %	
Absorption correction	Gaussian	
Max. and min. transmission	0.994 and 0.979	
Refinement method	Full-matrix least-squares on F^2	
Data / restraints / parameters	4360 / 319 / 405	
Goodness-of-fit on F2	1.027	
Final R indices [I>2sigma(I)]	R1 = 0.0541, wR2 = 0.1280	
R indices (all data)	R1 = 0.0694, wR2 = 0.1414	
Absolute structure parameter	1.7(6)	
Extinction coefficient	n/a	
Largest diff. peak and hole	0.329 and -0.208 e.Å^{-3}	

Table S4. Atomic coordinates (x 10^4) and equivalent isotropic displacement parameters (Å^{2}x 10^3) For **27**. U(eq) is defined as one third of the trace of the orthogonalized U^{ij} tensor.

	x	y	z	U(eq)
N(1A)	6568(4)	7740(12)	7165(12)	63(3)
O(1A)	6771(4)	6672(13)	6538(7)	88(2)
O(2A)	6834(2)	8999(8)	7479(7)	95(3)
C(1A)	5965(8)	7459(16)	7590(30)	47(2)
C(2A)	5663(4)	8781(13)	8052(7)	52(2)
C(3A)	5111(4)	8450(16)	8494(8)	47(2)
C(5A)	5207(8)	5540(20)	8058(15)	43(3)
C(6A)	5779(6)	5838(18)	7668(15)	48(3)

N(1B)	6504(11)	7640(30)	6980(30)	62(4)
O(1B)	6869(8)	6540(30)	7103(19)	87(5)
O(2B)	6605(7)	8893(19)	6427(15)	85(5)
C(1B)	5937(19)	7420(40)	7560(70)	50(3)
C(2B)	5555(9)	8750(30)	7690(18)	48(3)
C(3B)	5009(10)	8380(40)	8180(20)	46(4)
C(5B)	5230(20)	5480(50)	8220(40)	44(4)
C(6B)	5718(16)	5820(40)	7540(40)	46(3)
C(4)	4879(2)	6850(5)	8512(3)	39(1)
C(7)	4300(2)	6494(6)	9058(3)	43(1)
O(3)	3934(1)	7537(5)	9269(3)	65(1)
O(4)	4253(1)	4863(4)	9297(2)	43(1)
O(8)	3721(1)	2259(4)	3030(2)	48(1)
O(6)	3436(1)	4051(4)	7795(2)	43(1)
O(7)	4672(1)	1956(5)	6341(2)	52(1)
O(5)	2871(1)	2398(6)	11100(2)	65(1)
N(3)	4175(1)	2061(5)	4700(2)	39(1)
N(2)	3668(1)	2262(5)	6353(2)	41(1)
C(18)	4206(2)	2085(6)	5841(3)	40(1)
C(17)	3675(2)	2257(5)	4047(3)	38(1)
C(14)	3644(2)	2433(6)	7566(3)	37(1)
C(8)	3714(2)	4268(6)	9792(3)	40(1)
C(16)	3125(2)	2448(6)	4644(3)	41(1)
C(11)	2979(2)	2225(7)	9129(3)	45(1)
C(10)	3241(2)	1695(7)	10254(3)	48(1)
C(12)	3214(2)	4031(6)	8910(3)	43(1)
C(15)	3153(2)	2468(7)	5753(3)	45(1)
C(9)	3843(2)	2544(7)	10274(3)	47(1)
C(19)	2562(2)	2619(9)	3992(4)	65(2)
C(13)	3219(2)	1221(6)	8146(3)	48(1)
O(9)	4258(9)	5580(20)	5153(16)	239(8)
O(10)	4918(6)	7481(15)	5297(8)	109(4)
C(20)	4027(7)	8000(20)	6227(12)	105(5)
C(21)	4376(7)	6940(20)	5470(20)	111(5)
C(22)	5253(6)	6380(20)	4668(13)	110(5)
C(23)	5790(8)	7320(40)	4290(20)	135(7)

Table S5. Bond lengths [Å] and angles [°] for **27**.

N(1A)-O(1A)	1.227(12)
N(1A)-O(2A)	1.228(13)
N(1A)-C(1A)	1.476(9)
C(1A)-C(6A)	1.361(10)
C(1A)-C(2A)	1.373(13)
C(2A)-C(3A)	1.384(9)
C(2A)-H(2A)	0.9400
C(3A)-C(4)	1.378(13)
C(3A)-H(3A)	0.9400
C(5A)-C(4)	1.394(17)
C(5A)-C(6A)	1.396(12)

C(5A)-H(5A)	0.9400
C(6A)-H(6A)	0.9400
N(1B)-O(1B)	1.21(2)
N(1B)-O(2B)	1.22(2)
N(1B)-C(1B)	1.474(19)
C(1B)-C(6B)	1.37(2)
C(1B)-C(2B)	1.38(2)
C(2B)-C(3B)	1.400(19)
C(2B)-H(2B)	0.9400
C(3B)-C(4)	1.32(3)
C(3B)-H(3B)	0.9400
C(5B)-C(4)	1.40(4)
C(5B)-C(6B)	1.40(2)
C(5B)-H(5B)	0.9400
C(6B)-H(6B)	0.9400
C(4)-C(7)	1.493(5)
C(7)-O(3)	1.200(5)
C(7)-O(4)	1.335(6)
O(4)-C(8)	1.440(5)
O(8)-C(17)	1.230(4)
O(6)-C(14)	1.399(5)
O(6)-C(12)	1.435(4)
O(7)-C(18)	1.217(4)
O(5)-C(10)	1.434(5)
O(5)-H(5)	0.8300
N(3)-C(18)	1.376(4)
N(3)-C(17)	1.384(5)
N(3)-H(3)	0.81(8)
N(2)-C(18)	1.373(4)
N(2)-C(15)	1.378(5)
N(2)-C(14)	1.468(4)
C(17)-C(16)	1.447(5)
C(14)-C(13)	1.533(6)
C(14)-H(14)	0.9900
C(8)-C(9)	1.518(7)
C(8)-C(12)	1.560(5)
C(8)-H(8)	0.9900
C(16)-C(15)	1.337(5)
C(16)-C(19)	1.500(5)
C(11)-C(13)	1.529(6)
C(11)-C(10)	1.537(5)
C(11)-C(12)	1.555(7)
C(11)-H(11)	0.9900
C(10)-C(9)	1.521(6)
C(10)-H(10)	0.9900
C(12)-H(12)	0.9900
C(15)-H(15)	0.9400
C(9)-H(9A)	0.9800
C(9)-H(9B)	0.9800
C(19)-H(19A)	0.9700
C(19)-H(19B)	0.9700
C(19)-H(19C)	0.9700
C(13)-H(13A)	0.9800

C(13)-H(13B)	0.9800
O(9)-C(21)	1.175(12)
O(10)-C(21)	1.317(12)
O(10)-C(22)	1.386(11)
C(20)-C(21)	1.472(12)
C(20)-H(20A)	0.9700
C(20)-H(20B)	0.9700
C(20)-H(20C)	0.9700
C(22)-C(23)	1.496(12)
C(22)-H(22A)	0.9800
C(22)-H(22B)	0.9800
C(23)-H(23A)	0.9700
C(23)-H(23B)	0.9700
C(23)-H(23C)	0.9700
O(1A)-N(1A)-O(2A)	124.9(9)
O(1A)-N(1A)-C(1A)	117.1(11)
O(2A)-N(1A)-C(1A)	118.0(11)
C(6A)-C(1A)-C(2A)	123.0(9)
C(6A)-C(1A)-N(1A)	117.0(9)
C(2A)-C(1A)-N(1A)	119.1(10)
C(1A)-C(2A)-C(3A)	117.4(8)
C(1A)-C(2A)-H(2A)	121.3
C(3A)-C(2A)-H(2A)	121.3
C(4)-C(3A)-C(2A)	121.8(9)
C(4)-C(3A)-H(3A)	119.1
C(2A)-C(3A)-H(3A)	119.1
C(4)-C(5A)-C(6A)	120.0(12)
C(4)-C(5A)-H(5A)	120.0
C(6A)-C(5A)-H(5A)	120.0
C(1A)-C(6A)-C(5A)	118.1(11)
C(1A)-C(6A)-H(6A)	121.0
C(5A)-C(6A)-H(6A)	121.0
O(1B)-N(1B)-O(2B)	122(2)
O(1B)-N(1B)-C(1B)	116(2)
O(2B)-N(1B)-C(1B)	121(2)
C(6B)-C(1B)-C(2B)	119(3)
C(6B)-C(1B)-N(1B)	115(2)
C(2B)-C(1B)-N(1B)	121(2)
C(1B)-C(2B)-C(3B)	116(2)
C(1B)-C(2B)-H(2B)	121.8
C(3B)-C(2B)-H(2B)	121.8
C(4)-C(3B)-C(2B)	121(2)
C(4)-C(3B)-H(3B)	119.3
C(2B)-C(3B)-H(3B)	119.3
C(4)-C(5B)-C(6B)	117(3)
C(4)-C(5B)-H(5B)	121.7
C(6B)-C(5B)-H(5B)	121.7
C(1B)-C(6B)-C(5B)	117(3)
C(1B)-C(6B)-H(6B)	121.5
C(5B)-C(6B)-H(6B)	121.5
C(3A)-C(4)-C(5A)	118.8(7)
C(3B)-C(4)-C(5B)	121.1(17)

C(3B)-C(4)-C(7)	120.5(11)
C(3A)-C(4)-C(7)	121.1(5)
C(5A)-C(4)-C(7)	120.0(7)
C(5B)-C(4)-C(7)	117.6(14)
O(3)-C(7)-O(4)	124.8(4)
O(3)-C(7)-C(4)	124.6(4)
O(4)-C(7)-C(4)	110.5(4)
C(7)-O(4)-C(8)	118.6(3)
C(14)-O(6)-C(12)	107.0(3)
C(10)-O(5)-H(5)	109.5
C(18)-N(3)-C(17)	127.3(3)
C(18)-N(3)-H(3)	116(6)
C(17)-N(3)-H(3)	117(6)
C(18)-N(2)-C(15)	121.6(3)
C(18)-N(2)-C(14)	119.5(3)
C(15)-N(2)-C(14)	118.5(3)
O(7)-C(18)-N(2)	123.6(3)
O(7)-C(18)-N(3)	122.3(3)
N(2)-C(18)-N(3)	114.1(3)
O(8)-C(17)-N(3)	119.7(3)
O(8)-C(17)-C(16)	124.8(3)
N(3)-C(17)-C(16)	115.5(3)
O(6)-C(14)-N(2)	107.2(3)
O(6)-C(14)-C(13)	106.1(3)
N(2)-C(14)-C(13)	114.8(3)
O(6)-C(14)-H(14)	109.5
N(2)-C(14)-H(14)	109.5
C(13)-C(14)-H(14)	109.5
O(4)-C(8)-C(9)	107.1(3)
O(4)-C(8)-C(12)	111.8(3)
C(9)-C(8)-C(12)	106.8(3)
O(4)-C(8)-H(8)	110.4
C(9)-C(8)-H(8)	110.4
C(12)-C(8)-H(8)	110.4
C(15)-C(16)-C(17)	117.3(3)
C(15)-C(16)-C(19)	124.1(3)
C(17)-C(16)-C(19)	118.6(3)
C(13)-C(11)-C(10)	113.7(4)
C(13)-C(11)-C(12)	103.3(3)
C(10)-C(11)-C(12)	105.8(3)
C(13)-C(11)-H(11)	111.2
C(10)-C(11)-H(11)	111.2
C(12)-C(11)-H(11)	111.2
O(5)-C(10)-C(9)	109.8(4)
O(5)-C(10)-C(11)	107.2(4)
C(9)-C(10)-C(11)	103.6(3)
O(5)-C(10)-H(10)	111.9
C(9)-C(10)-H(10)	111.9
C(11)-C(10)-H(10)	111.9
O(6)-C(12)-C(11)	106.8(3)
O(6)-C(12)-C(8)	112.4(3)
C(11)-C(12)-C(8)	104.1(3)
O(6)-C(12)-H(12)	111.1

C(11)-C(12)-H(12)	111.1
C(8)-C(12)-H(12)	111.1
C(16)-C(15)-N(2)	124.1(3)
C(16)-C(15)-H(15)	117.9
N(2)-C(15)-H(15)	117.9
C(8)-C(9)-C(10)	103.0(4)
C(8)-C(9)-H(9A)	111.2
C(10)-C(9)-H(9A)	111.2
C(8)-C(9)-H(9B)	111.2
C(10)-C(9)-H(9B)	111.2
H(9A)-C(9)-H(9B)	109.1
C(16)-C(19)-H(19A)	109.5
C(16)-C(19)-H(19B)	109.5
H(19A)-C(19)-H(19B)	109.5
C(16)-C(19)-H(19C)	109.5
H(19A)-C(19)-H(19C)	109.5
H(19B)-C(19)-H(19C)	109.5
C(11)-C(13)-C(14)	104.4(4)
C(11)-C(13)-H(13A)	110.9
C(14)-C(13)-H(13A)	110.9
C(11)-C(13)-H(13B)	110.9
C(14)-C(13)-H(13B)	110.9
H(13A)-C(13)-H(13B)	108.9
C(21)-O(10)-C(22)	113.0(11)
C(21)-C(20)-H(20A)	109.5
C(21)-C(20)-H(20B)	109.5
H(20A)-C(20)-H(20B)	109.5
C(21)-C(20)-H(20C)	109.5
H(20A)-C(20)-H(20C)	109.5
H(20B)-C(20)-H(20C)	109.5
O(9)-C(21)-O(10)	117.4(14)
O(9)-C(21)-C(20)	127.4(15)
O(10)-C(21)-C(20)	114.3(12)
O(10)-C(22)-C(23)	107.1(17)
O(10)-C(22)-H(22A)	110.3
C(23)-C(22)-H(22A)	110.3
O(10)-C(22)-H(22B)	110.3
C(23)-C(22)-H(22B)	110.3
H(22A)-C(22)-H(22B)	108.5
C(22)-C(23)-H(23A)	109.5
C(22)-C(23)-H(23B)	109.5
H(23A)-C(23)-H(23B)	109.5
C(22)-C(23)-H(23C)	109.5
H(23A)-C(23)-H(23C)	109.5
H(23B)-C(23)-H(23C)	109.5

Symmetry transformations used to generate equivalent atoms:

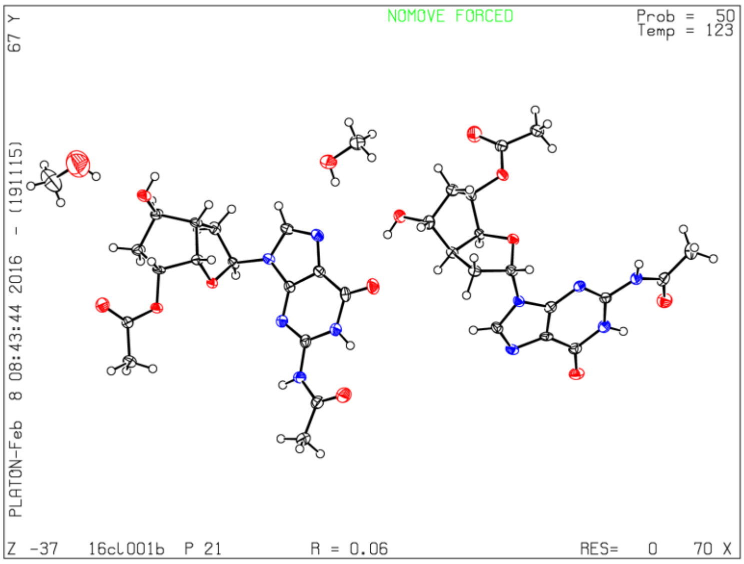

Crystal-Structure Determination of 20. –A colorless transparent crystal of [$C_{16}H_{19}N_5O_6$].(CH_4O) was mounted in air and used for X-ray structure determination at ambient conditions. All measurements were made on a *Oxford Diffraction SuperNova* area-detector diffractometer[2] using mirror optics monochromated Mo *Kα* radiation (λ = 0.71073 Å) and Al filtered.[3] The unit cell constants and an orientation matrix for data collection were obtained from a least-squares refinement of the setting angles of reflections in the range 1.5° < θ < 27.2°. A total of 970 frames were collected using ω scans, with 45+45 seconds exposure time, a rotation angle of 1° per frame, a crystal-detector distance of 65.1 mm, at T = 123(2) K.

Data reduction was performed using the *CrysAlisPro*[2] program. The intensities were corrected for Lorentz and polarization effects, and an absorption correction based on the multi-scan method using SCALE3 ABSPACK in *CrysAlisPro*[2] was applied. Data collection and refinement parameters are given in *Table S6.*

The structure was solved by direct methods using *SHELXS-97*[4], which revealed the positions of all non-hydrogen atoms of the title compound. The non-hydrogen atoms were refined anisotropically. All H-atoms were placed in geometrically calculated positions and refined using a riding model where each H-atom was assigned a fixed isotropic displacement parameter with a value equal to 1.2 Ueq of its parent atom.

Refinement of the structure was carried out on F^2 using full-matrix least-squares procedures, which minimized the function $\Sigma w(F_o^2 - F_c^2)^2$. The weighting scheme was based on counting statistics and included a factor to downweight the intense reflections. All calculations were performed using the *SHELXL-97*[3] program.

Table S6. Crystal data and structure refinement for **20**.

Empirical formula	C17 H23 N5 O7
Formula weight	409.40
Temperature	123(2) K
Wavelength	0.71073 Å
Crystal system	Monoclinic
Space group	P 21

Unit cell dimensions	a = 4.83370(10) Å	a= 90°.
	b = 25.1367(4) Å	b= 92.4850(10)°.
	c = 15.2979(2) Å	g = 90°.
Volume	1857.00(5) $Å^3$	
Z	4	
Density (calculated)	1.464 Mg/m^3	
Absorption coefficient	0.115 mm^{-1}	
F(000)	864	
Crystal size	0.4678 x 0.1255 x 0.0405 mm^3	
Theta range for data collection	1.559 to 27.205°.	
Index ranges	-6<=h<=6, -31<=k<=32, -19<=l<=19	
Reflections collected	25075	
Independent reflections	7480 [R(int) = 0.0452]	
Completeness to theta = 25.000°	100.0 %	
Absorption correction	Gaussian	
Max. and min. transmission	0.996 and 0.97	
Refinement method	Full-matrix least-squares on F^2	
Data / restraints / parameters	7480 / 1 / 533	
Goodness-of-fit on F2	1.058	
Final R indices [I>2sigma(I)]	R1 = 0.0579, wR2 = 0.1456	
R indices (all data)	R1 = 0.0657, wR2 = 0.1518	
Absolute structure parameter	0.7(4)	
Extinction coefficient	n/a	
Largest diff. peak and hole	1.403 and -0.492 e.$Å^{-3}$	

Table S7. Atomic coordinates (x 10^4) and equivalent isotropic displacement parameters ($Å^2$x 10^3) For **20**. U(eq) is defined as one third of the trace of the orthogonalized Uij tensor.

	x	y	z	U(eq)
O(104)	8096(7)	6256(1)	2370(2)	19(1)
O(202)	-2251(7)	2928(1)	8632(2)	20(1)
O(204)	-2777(7)	3802(1)	7746(2)	19(1)
O(103)	6024(8)	5715(2)	4998(2)	28(1)
O(105)	828(7)	5549(2)	-1052(2)	27(1)
O(203)	132(8)	4159(2)	10461(2)	26(1)
O(102)	7720(7)	7065(1)	3416(2)	22(1)
O(201)	-1750(8)	2445(2)	9852(2)	30(1)
O(106)	4154(8)	7076(2)	-1835(2)	31(1)
O(205)	4163(8)	4664(2)	4581(2)	32(1)
O(206)	1467(8)	3129(2)	3681(2)	35(1)
O(1S)	5410(9)	5863(2)	7197(3)	39(1)
O(101)	5629(10)	7527(2)	4445(3)	42(1)
N(101)	6745(8)	5569(2)	1391(2)	18(1)
N(201)	-1522(8)	4540(2)	6901(3)	18(1)
N(202)	1787(9)	5042(2)	6319(3)	22(1)
N(102)	3166(8)	5125(2)	746(3)	20(1)
N(204)	1147(9)	3974(2)	4680(3)	24(1)
N(103)	7416(8)	6262(2)	316(2)	18(1)
N(203)	-2062(8)	3853(2)	5791(2)	20(1)
N(104)	4170(9)	6185(2)	-878(3)	23(1)
N(105)	7480(9)	6871(2)	-796(3)	24(1)

N(205)	-1788(9)	3227(2)	4700(3)	24(1)
C(212)	-919(9)	4303(2)	6127(3)	17(1)
C(113)	2769(10)	5729(2)	-606(3)	21(1)
C(111)	6117(9)	5817(2)	610(3)	19(1)
C(202)	-2254(10)	2472(2)	9071(3)	18(1)
C(210)	182(10)	4982(2)	6973(3)	20(1)
C(114)	6338(10)	6416(2)	-438(3)	20(1)
C(109)	8871(10)	5756(2)	2037(3)	20(1)
C(110)	4886(10)	5154(2)	1427(3)	20(1)
C(112)	3922(10)	5550(2)	218(3)	20(1)
C(211)	1149(10)	4615(2)	5781(3)	21(1)
C(209)	-3544(9)	4323(2)	7509(3)	18(1)
C(213)	2319(10)	4449(2)	4985(3)	22(1)
C(103)	6929(10)	6565(2)	3800(3)	20(1)
C(115)	6316(11)	7191(2)	-1435(3)	26(1)
C(208)	-3580(10)	4630(2)	8359(3)	21(1)
C(102)	6812(10)	7516(2)	3769(3)	21(1)
C(207)	-721(9)	3829(2)	8454(3)	17(1)
C(105)	8178(10)	5725(2)	4395(3)	22(1)
C(214)	-902(10)	3702(2)	5082(3)	22(1)
C(204)	-3378(10)	3641(2)	9722(3)	22(1)
C(206)	-1262(10)	4364(2)	8933(3)	18(1)
C(205)	-2214(10)	4200(2)	9844(3)	20(1)
C(107)	6256(9)	6166(2)	3072(3)	15(1)
C(104)	9206(10)	6295(2)	4358(3)	23(1)
C(106)	7025(10)	5612(2)	3455(3)	18(1)
C(108)	9194(10)	5398(2)	2835(3)	20(1)
C(201)	-2957(12)	2012(2)	8486(3)	27(1)
C(203)	-1312(10)	3395(2)	9119(3)	18(1)
C(101)	7465(12)	7991(2)	3232(3)	27(1)
C(216)	-1818(13)	2423(2)	3825(4)	34(1)
C(215)	-545(11)	2950(2)	4057(3)	26(1)
C(1S)	7413(13)	6233(3)	6919(4)	36(1)
C(116)	7913(13)	7697(2)	-1592(4)	34(1)
O(2S)	-1259(18)	4106(4)	12221(5)	102(2)
C(2S)	-3709(18)	3827(4)	12461(6)	69(2)

Table S8. Bond lengths [Å] and angles [°] for **20**.

O(104)-C(109)	1.413(6)
O(104)-C(107)	1.441(5)
O(202)-C(202)	1.329(6)
O(202)-C(203)	1.452(5)
O(204)-C(209)	1.405(6)
O(204)-C(207)	1.439(5)
O(103)-C(105)	1.421(6)
O(103)-H(123)	0.8400
O(105)-C(113)	1.222(6)
O(203)-C(205)	1.447(6)
O(203)-H(223)	0.8400

O(102)-C(102)	1.337(6)
O(102)-C(103)	1.445(6)
O(201)-C(202)	1.211(6)
O(106)-C(115)	1.222(6)
O(205)-C(213)	1.230(6)
O(206)-C(215)	1.236(7)
O(1S)-C(1S)	1.422(7)
O(1S)-H(1S)	0.8400
O(101)-C(102)	1.204(6)
N(101)-C(111)	1.369(6)
N(101)-C(110)	1.380(6)
N(101)-C(109)	1.472(6)
N(201)-C(212)	1.369(6)
N(201)-C(210)	1.385(6)
N(201)-C(209)	1.481(6)
N(202)-C(210)	1.301(6)
N(202)-C(211)	1.380(7)
N(102)-C(110)	1.307(6)
N(102)-C(112)	1.396(6)
N(204)-C(214)	1.371(6)
N(204)-C(213)	1.393(7)
N(204)-H(204)	0.8800
N(103)-C(114)	1.303(6)
N(103)-C(111)	1.369(6)
N(203)-C(214)	1.300(6)
N(203)-C(212)	1.351(6)
N(104)-C(114)	1.352(6)
N(104)-C(113)	1.403(7)
N(104)-H(104)	0.8800
N(105)-C(115)	1.370(7)
N(105)-C(114)	1.393(6)
N(105)-H(125)	0.8800
N(205)-C(215)	1.365(6)
N(205)-C(214)	1.388(7)
N(205)-H(225)	0.8800
C(212)-C(211)	1.393(7)
C(113)-C(112)	1.429(7)
C(111)-C(112)	1.372(7)
C(202)-C(201)	1.493(7)
C(210)-H(210)	0.9500
C(109)-C(108)	1.520(7)
C(109)-H(109)	1.0000
C(110)-H(110)	0.9500
C(211)-C(213)	1.426(7)
C(209)-C(208)	1.513(7)
C(209)-H(209)	1.0000
C(103)-C(104)	1.524(7)
C(103)-C(107)	1.524(7)
C(103)-H(103)	1.0000
C(115)-C(116)	1.511(8)
C(208)-C(206)	1.545(7)
C(208)-H(20A)	0.9900
C(208)-H(20B)	0.9900

C(102)-C(101)	1.490(7)
C(207)-C(203)	1.527(7)
C(207)-C(206)	1.560(6)
C(207)-H(207)	1.0000
C(105)-C(104)	1.517(8)
C(105)-C(106)	1.546(6)
C(105)-H(105)	1.0000
C(204)-C(203)	1.520(6)
C(204)-C(205)	1.522(7)
C(204)-H(20C)	0.9900
C(204)-H(20D)	0.9900
C(206)-C(205)	1.542(6)
C(206)-H(206)	1.0000
C(205)-H(205)	1.0000
C(107)-C(106)	1.551(7)
C(107)-H(107)	1.0000
C(104)-H(10A)	0.9900
C(104)-H(10B)	0.9900
C(106)-C(108)	1.541(6)
C(106)-H(106)	1.0000
C(108)-H(10C)	0.9900
C(108)-H(10D)	0.9900
C(201)-H(20E)	0.9800
C(201)-H(20F)	0.9800
C(201)-H(20G)	0.9800
C(203)-H(203)	1.0000
C(101)-H(10E)	0.9800
C(101)-H(10F)	0.9800
C(101)-H(10G)	0.9800
C(216)-C(215)	1.498(8)
C(216)-H(21A)	0.9800
C(216)-H(21B)	0.9800
C(216)-H(21C)	0.9800
C(1S)-H(1S1)	0.9800
C(1S)-H(1S2)	0.9800
C(1S)-H(1S3)	0.9800
C(116)-H(11A)	0.9800
C(116)-H(11B)	0.9800
C(116)-H(11C)	0.9800
O(2S)-C(2S)	1.437(11)
O(2S)-H(2S)	0.8400
C(2S)-H(2S1)	0.9800
C(2S)-H(2S2)	0.9800
C(2S)-H(2S3)	0.9800
C(109)-O(104)-C(107)	108.1(3)
C(202)-O(202)-C(203)	116.4(4)
C(209)-O(204)-C(207)	108.4(3)
C(105)-O(103)-H(123)	109.5
C(205)-O(203)-H(223)	109.5
C(102)-O(102)-C(103)	118.4(4)
C(1S)-O(1S)-H(1S)	109.5
C(111)-N(101)-C(110)	105.0(4)

C(111)-N(101)-C(109)	124.2(4)
C(110)-N(101)-C(109)	130.6(4)
C(212)-N(201)-C(210)	105.6(4)
C(212)-N(201)-C(209)	123.7(4)
C(210)-N(201)-C(209)	130.7(4)
C(210)-N(202)-C(211)	104.1(4)
C(110)-N(102)-C(112)	104.2(4)
C(214)-N(204)-C(213)	124.7(4)
C(214)-N(204)-H(204)	117.7
C(213)-N(204)-H(204)	117.7
C(114)-N(103)-C(111)	111.2(4)
C(214)-N(203)-C(212)	112.2(4)
C(114)-N(104)-C(113)	125.2(4)
C(114)-N(104)-H(104)	117.4
C(113)-N(104)-H(104)	117.4
C(115)-N(105)-C(114)	127.4(4)
C(115)-N(105)-H(125)	116.3
C(114)-N(105)-H(125)	116.3
C(215)-N(205)-C(214)	127.2(4)
C(215)-N(205)-H(225)	116.4
C(214)-N(205)-H(225)	116.4
N(203)-C(212)-N(201)	126.5(4)
N(203)-C(212)-C(211)	128.0(4)
N(201)-C(212)-C(211)	105.5(4)
O(105)-C(113)-N(104)	120.3(5)
O(105)-C(113)-C(112)	129.5(5)
N(104)-C(113)-C(112)	110.1(4)
N(101)-C(111)-N(103)	124.8(4)
N(101)-C(111)-C(112)	107.3(4)
N(103)-C(111)-C(112)	127.8(4)
O(201)-C(202)-O(202)	122.9(5)
O(201)-C(202)-C(201)	125.4(5)
O(202)-C(202)-C(201)	111.7(4)
N(202)-C(210)-N(201)	113.9(4)
N(202)-C(210)-H(210)	123.1
N(201)-C(210)-H(210)	123.1
N(103)-C(114)-N(104)	125.8(4)
N(103)-C(114)-N(105)	116.2(4)
N(104)-C(114)-N(105)	117.9(4)
O(104)-C(109)-N(101)	109.8(4)
O(104)-C(109)-C(108)	104.9(4)
N(101)-C(109)-C(108)	113.2(4)
O(104)-C(109)-H(109)	109.6
N(101)-C(109)-H(109)	109.6
C(108)-C(109)-H(109)	109.6
N(102)-C(110)-N(101)	113.7(4)
N(102)-C(110)-H(110)	123.1
N(101)-C(110)-H(110)	123.1
C(111)-C(112)-N(102)	109.8(4)
C(111)-C(112)-C(113)	119.8(5)
N(102)-C(112)-C(113)	130.5(4)
N(202)-C(211)-C(212)	110.9(4)
N(202)-C(211)-C(213)	130.5(4)

C(212)-C(211)-C(213)	118.6(5)
O(204)-C(209)-N(201)	109.4(3)
O(204)-C(209)-C(208)	105.4(4)
N(201)-C(209)-C(208)	112.6(4)
O(204)-C(209)-H(209)	109.8
N(201)-C(209)-H(209)	109.8
C(208)-C(209)-H(209)	109.8
O(205)-C(213)-N(204)	120.0(5)
O(205)-C(213)-C(211)	128.6(5)
N(204)-C(213)-C(211)	111.4(4)
O(102)-C(103)-C(104)	114.8(4)
O(102)-C(103)-C(107)	109.1(4)
C(104)-C(103)-C(107)	104.0(4)
O(102)-C(103)-H(103)	109.6
C(104)-C(103)-H(103)	109.6
C(107)-C(103)-H(103)	109.6
O(106)-C(115)-N(105)	122.2(5)
O(106)-C(115)-C(116)	123.5(5)
N(105)-C(115)-C(116)	114.3(5)
C(209)-C(208)-C(206)	103.5(4)
C(209)-C(208)-H(20A)	111.1
C(206)-C(208)-H(20A)	111.1
C(209)-C(208)-H(20B)	111.1
C(206)-C(208)-H(20B)	111.1
H(20A)-C(208)-H(20B)	109.0
O(101)-C(102)-O(102)	122.9(5)
O(101)-C(102)-C(101)	125.1(5)
O(102)-C(102)-C(101)	112.0(4)
O(204)-C(207)-C(203)	108.9(4)
O(204)-C(207)-C(206)	105.7(4)
C(203)-C(207)-C(206)	105.2(3)
O(204)-C(207)-H(207)	112.2
C(203)-C(207)-H(207)	112.2
C(206)-C(207)-H(207)	112.2
O(103)-C(105)-C(104)	107.0(4)
O(103)-C(105)-C(106)	110.8(4)
C(104)-C(105)-C(106)	104.2(4)
O(103)-C(105)-H(105)	111.5
C(104)-C(105)-H(105)	111.5
C(106)-C(105)-H(105)	111.5
N(203)-C(214)-N(204)	125.0(5)
N(203)-C(214)-N(205)	117.8(4)
N(204)-C(214)-N(205)	117.2(4)
C(203)-C(204)-C(205)	101.6(4)
C(203)-C(204)-H(20C)	111.5
C(205)-C(204)-H(20C)	111.5
C(203)-C(204)-H(20D)	111.5
C(205)-C(204)-H(20D)	111.5
H(20C)-C(204)-H(20D)	109.3
C(205)-C(206)-C(208)	113.1(4)
C(205)-C(206)-C(207)	104.9(4)
C(208)-C(206)-C(207)	103.7(4)
C(205)-C(206)-H(206)	111.6

C(208)-C(206)-H(206)	111.6
C(207)-C(206)-H(206)	111.6
O(203)-C(205)-C(204)	106.8(4)
O(203)-C(205)-C(206)	110.6(4)
C(204)-C(205)-C(206)	105.1(4)
O(203)-C(205)-H(205)	111.3
C(204)-C(205)-H(205)	111.3
C(206)-C(205)-H(205)	111.3
O(104)-C(107)-C(103)	108.8(4)
O(104)-C(107)-C(106)	106.0(4)
C(103)-C(107)-C(106)	106.0(4)
O(104)-C(107)-H(107)	111.9
C(103)-C(107)-H(107)	111.9
C(106)-C(107)-H(107)	111.9
C(105)-C(104)-C(103)	102.3(4)
C(105)-C(104)-H(10A)	111.3
C(103)-C(104)-H(10A)	111.3
C(105)-C(104)-H(10B)	111.3
C(103)-C(104)-H(10B)	111.3
H(10A)-C(104)-H(10B)	109.2
C(108)-C(106)-C(105)	114.2(4)
C(108)-C(106)-C(107)	103.9(4)
C(105)-C(106)-C(107)	104.9(4)
C(108)-C(106)-H(106)	111.2
C(105)-C(106)-H(106)	111.2
C(107)-C(106)-H(106)	111.2
C(109)-C(108)-C(106)	103.8(4)
C(109)-C(108)-H(10C)	111.0
C(106)-C(108)-H(10C)	111.0
C(109)-C(108)-H(10D)	111.0
C(106)-C(108)-H(10D)	111.0
H(10C)-C(108)-H(10D)	109.0
C(202)-C(201)-H(20E)	109.5
C(202)-C(201)-H(20F)	109.5
H(20E)-C(201)-H(20F)	109.5
C(202)-C(201)-H(20G)	109.5
H(20E)-C(201)-H(20G)	109.5
H(20F)-C(201)-H(20G)	109.5
O(202)-C(203)-C(204)	116.2(4)
O(202)-C(203)-C(207)	107.4(3)
C(204)-C(203)-C(207)	105.1(4)
O(202)-C(203)-H(203)	109.3
C(204)-C(203)-H(203)	109.3
C(207)-C(203)-H(203)	109.3
C(102)-C(101)-H(10E)	109.5
C(102)-C(101)-H(10F)	109.5
H(10E)-C(101)-H(10F)	109.5
C(102)-C(101)-H(10G)	109.5
H(10E)-C(101)-H(10G)	109.5
H(10F)-C(101)-H(10G)	109.5
C(215)-C(216)-H(21A)	109.5
C(215)-C(216)-H(21B)	109.5
H(21A)-C(216)-H(21B)	109.5

C(215)-C(216)-H(21C)	109.5
H(21A)-C(216)-H(21C)	109.5
H(21B)-C(216)-H(21C)	109.5
O(206)-C(215)-N(205)	121.9(5)
O(206)-C(215)-C(216)	122.4(5)
N(205)-C(215)-C(216)	115.6(5)
O(1S)-C(1S)-H(1S1)	109.5
O(1S)-C(1S)-H(1S2)	109.5
H(1S1)-C(1S)-H(1S2)	109.5
O(1S)-C(1S)-H(1S3)	109.5
H(1S1)-C(1S)-H(1S3)	109.5
H(1S2)-C(1S)-H(1S3)	109.5
C(115)-C(116)-H(11A)	109.5
C(115)-C(116)-H(11B)	109.5
H(11A)-C(116)-H(11B)	109.5
C(115)-C(116)-H(11C)	109.5
H(11A)-C(116)-H(11C)	109.5
H(11B)-C(116)-H(11C)	109.5
C(2S)-O(2S)-H(2S)	109.5
O(2S)-C(2S)-H(2S1)	109.5
O(2S)-C(2S)-H(2S2)	109.5
H(2S1)-C(2S)-H(2S2)	109.5
O(2S)-C(2S)-H(2S3)	109.5
H(2S1)-C(2S)-H(2S3)	109.5
H(2S2)-C(2S)-H(2S3)	109.5

Symmetry transformations used to generate equivalent atoms:

Table S9. Anisotropic displacement parameters ($Å^2$x 10^3) for **20**. The anisotropic displacement factor exponent takes the form: $-2\pi^2[h^2a^{*2}U^{11} + ... + 2 h k a^* b^* U^{12}]$

	U^{11}	U^{22}	U^{33}	U^{23}	U^{13}	U^{12}
O(104)	21(2)	14(2)	21(2)	-1(1)	4(1)	0(1)
O(202)	23(2)	18(2)	20(2)	-1(1)	-4(1)	-4(1)
O(204)	22(2)	17(2)	17(2)	0(1)	-2(1)	-5(1)
O(103)	28(2)	30(2)	25(2)	-1(2)	6(2)	-12(2)
O(105)	24(2)	29(2)	27(2)	-7(2)	-2(2)	-4(2)
O(203)	26(2)	25(2)	28(2)	-1(2)	-4(1)	-8(2)
O(102)	25(2)	16(2)	26(2)	-1(1)	9(1)	-4(2)
O(201)	39(2)	25(2)	26(2)	4(2)	-2(2)	5(2)
O(106)	33(2)	32(2)	29(2)	1(2)	-7(2)	1(2)
O(205)	33(2)	31(2)	32(2)	3(2)	14(2)	-3(2)
O(206)	36(2)	37(2)	32(2)	-5(2)	9(2)	-1(2)
O(1S)	40(2)	39(2)	38(2)	-6(2)	-2(2)	-12(2)
O(101)	68(3)	22(2)	39(2)	-2(2)	24(2)	11(2)
N(101)	20(2)	15(2)	20(2)	1(2)	2(2)	0(2)
N(201)	16(2)	15(2)	22(2)	-1(2)	2(2)	-1(2)
N(202)	22(2)	16(2)	26(2)	3(2)	-1(2)	1(2)
N(102)	18(2)	16(2)	27(2)	-5(2)	5(2)	-1(2)
N(204)	29(2)	23(2)	22(2)	-1(2)	7(2)	-2(2)

N(103)	18(2)	20(2)	17(2)	-1(2)	-1(1)	0(2)
N(203)	19(2)	21(2)	19(2)	1(2)	-1(2)	1(2)
N(104)	24(2)	25(2)	19(2)	2(2)	-5(2)	-1(2)
N(105)	25(2)	27(2)	18(2)	1(2)	-3(2)	-6(2)
N(205)	25(2)	23(2)	23(2)	-4(2)	4(2)	-5(2)
C(212)	17(2)	15(2)	20(2)	2(2)	1(2)	3(2)
C(113)	21(2)	21(3)	22(2)	-5(2)	2(2)	0(2)
C(111)	17(2)	20(3)	19(2)	-2(2)	2(2)	4(2)
C(202)	19(2)	15(2)	20(2)	2(2)	-1(2)	4(2)
C(210)	18(2)	16(2)	27(2)	-2(2)	0(2)	1(2)
C(114)	21(2)	19(3)	20(2)	-3(2)	3(2)	-2(2)
C(109)	20(2)	16(2)	24(2)	-2(2)	4(2)	0(2)
C(110)	20(2)	16(2)	25(2)	-1(2)	8(2)	-2(2)
C(112)	22(2)	14(2)	23(2)	-8(2)	3(2)	1(2)
C(211)	22(2)	18(3)	24(2)	1(2)	2(2)	-3(2)
C(209)	14(2)	15(2)	24(2)	2(2)	4(2)	-1(2)
C(213)	22(2)	23(3)	22(2)	9(2)	2(2)	3(2)
C(103)	19(2)	16(2)	25(2)	1(2)	6(2)	-4(2)
C(115)	30(3)	30(3)	17(2)	0(2)	0(2)	4(2)
C(208)	14(2)	23(3)	26(2)	0(2)	6(2)	-1(2)
C(102)	24(2)	20(3)	20(2)	1(2)	-6(2)	1(2)
C(207)	12(2)	19(2)	20(2)	-4(2)	2(2)	-1(2)
C(105)	20(2)	26(3)	22(2)	4(2)	0(2)	1(2)
C(214)	24(3)	23(3)	18(2)	5(2)	-1(2)	1(2)
C(204)	18(2)	26(3)	24(2)	-6(2)	2(2)	-6(2)
C(206)	15(2)	17(2)	23(2)	-5(2)	3(2)	-4(2)
C(205)	17(2)	23(3)	21(2)	-6(2)	4(2)	-3(2)
C(107)	12(2)	15(2)	19(2)	1(2)	2(2)	-1(2)
C(104)	18(2)	27(3)	23(2)	-1(2)	-1(2)	-8(2)
C(106)	14(2)	17(2)	22(2)	1(2)	-1(2)	0(2)
C(108)	17(2)	19(2)	23(2)	-1(2)	-1(2)	0(2)
C(201)	35(3)	17(3)	28(3)	-1(2)	1(2)	0(2)
C(203)	15(2)	16(3)	22(2)	-7(2)	0(2)	-6(2)
C(101)	33(3)	20(3)	28(3)	0(2)	-3(2)	-4(2)
C(216)	50(4)	28(3)	24(2)	-5(2)	3(2)	-2(3)
C(215)	32(3)	25(3)	20(2)	0(2)	0(2)	2(2)
C(1S)	42(3)	31(3)	36(3)	-2(3)	6(2)	2(3)
C(116)	39(3)	35(3)	28(3)	8(2)	2(2)	0(3)
O(2S)	102(6)	106(6)	98(5)	13(5)	5(5)	-24(5)
C(2S)	64(5)	52(5)	91(6)	23(5)	-10(4)	-14(4)

Table S10. Anisotropic displacement parameters ($Å^2 x 10^3$) for **20**. The anisotropic displacement factor exponent takes the form: $-2\pi^2[h^2a^{*2}U^{11} + ... + 2 h k a^* b^* U^{12}]$

	U^{11}	U^{22}	U^{33}	U^{23}	U^{13}	U^{12}
N(1A)	43(4)	71(4)	76(6)	20(4)	5(4)	-5(3)
O(1A)	59(4)	118(6)	86(5)	-15(5)	27(4)	-10(4)
O(2A)	49(3)	67(4)	169(7)	15(4)	7(4)	-10(3)
C(1A)	36(3)	56(3)	48(3)	13(3)	1(3)	-1(3)
C(2A)	43(4)	50(3)	61(5)	6(4)	-5(3)	-10(3)
C(3A)	43(4)	40(4)	56(5)	-5(4)	1(4)	-2(3)

C(5A)	45(4)	41(4)	44(6)	0(4)	1(4)	-4(3)
C(6A)	37(4)	59(4)	48(5)	-2(3)	4(4)	2(3)
N(1B)	46(6)	65(6)	76(7)	14(6)	2(6)	1(5)
O(1B)	59(8)	94(10)	106(12)	21(10)	9(9)	27(7)
O(2B)	76(8)	74(8)	106(10)	30(7)	43(7)	4(7)
C(1B)	39(5)	56(5)	54(5)	9(5)	1(5)	-1(5)
C(2B)	44(6)	49(5)	50(7)	6(6)	-7(6)	0(5)
C(3B)	42(7)	43(7)	51(9)	3(7)	-1(7)	3(6)
C(5B)	43(6)	44(7)	45(9)	9(6)	-1(7)	1(6)
C(6B)	38(6)	53(5)	47(7)	-2(5)	-1(6)	1(5)
C(4)	37(2)	43(2)	36(2)	0(2)	0(2)	1(2)
C(7)	43(2)	48(3)	38(2)	0(2)	-1(2)	-1(2)
O(3)	52(2)	56(2)	87(2)	1(2)	20(2)	8(2)
O(4)	40(1)	50(2)	40(1)	3(1)	5(1)	-7(1)
O(8)	56(2)	64(2)	24(1)	2(1)	7(1)	-2(2)
O(6)	44(2)	59(2)	26(1)	-1(1)	8(1)	0(1)
O(7)	33(1)	92(3)	32(1)	-2(2)	5(1)	6(2)
O(5)	56(2)	108(3)	32(1)	-1(2)	15(1)	-12(2)
N(3)	34(2)	57(2)	27(1)	-2(2)	8(1)	2(2)
N(2)	33(1)	67(2)	24(1)	-1(2)	5(1)	2(2)
C(18)	37(2)	54(3)	28(2)	-1(2)	8(2)	0(2)
C(17)	43(2)	42(2)	27(2)	-2(2)	5(1)	1(2)
C(14)	34(2)	56(3)	22(2)	-2(2)	3(1)	4(2)
C(8)	37(2)	53(3)	31(2)	-3(2)	7(2)	-6(2)
C(16)	39(2)	56(3)	29(2)	-3(2)	2(1)	4(2)
C(11)	36(2)	69(3)	32(2)	-5(2)	7(1)	-12(2)
C(10)	50(2)	66(3)	28(2)	2(2)	11(2)	-5(2)
C(12)	37(2)	62(3)	30(2)	-2(2)	10(2)	2(2)
C(15)	32(2)	69(3)	33(2)	0(2)	5(1)	6(2)
C(9)	47(2)	62(3)	30(2)	5(2)	1(2)	-10(2)
C(19)	45(2)	112(5)	38(2)	1(3)	-1(2)	11(3)
C(13)	54(2)	60(3)	30(2)	-4(2)	7(2)	-8(2)
O(9)	369(19)	151(12)	196(14)	-41(11)	48(15)	-148(13)
O(10)	153(9)	98(6)	76(7)	2(5)	-45(7)	-10(7)
C(20)	128(12)	99(11)	87(10)	5(8)	-46(9)	-5(9)
C(21)	177(12)	83(8)	73(8)	-7(7)	-30(10)	-29(9)
C(22)	140(11)	115(11)	75(8)	12(7)	-57(8)	3(8)
C(23)	122(11)	190(20)	93(14)	56(12)	-62(10)	-3(12)

8. References

1. Evéquoz, D.; Leumann, C. J., Probing the Backbone Topology of DNA: Synthesis and Properties of 7',5'-Bicyclo-DNA. *Chemistry (Weinheim an der Bergstrasse, Germany)* **2017,** *23* (33), 7953-7968.
2. Oxford Diffraction (2010). CrysAlisPro(Version 1.171.34.44). Oxford Diffraction Ltd., Yarnton, Oxfordshire, UK.

3. Macchi, P.; Burgi, H.-B.; Chimpri, A. S.; Hauser, J.; Gal, Z., Low-energy contamination of Mo microsource X-ray radiation: analysis and solution of the problem. *Journal of Applied Crystallography* **2011,** *44* (4), 763-771.
4. Sheldrick, G., A short history of SHELX. *Acta Crystallographica Section A* **2008,** *64* (1), 112-122.
